# Diastereodivergent and Enantioselective Organocatalytic Synthesis of Spiro-Fused Coumarins via [4+2] Cycloadditions

**DOI:** 10.1021/acs.orglett.5c05358

**Published:** 2026-01-29

**Authors:** Raquel Hidalgo-León, José Trujillo-Sierra, José Miguel Sansano, Fernando P. Cossío, Abel de Cozar, María de Gracia Retamosa

**Affiliations:** † Departamento de Química Orgánica, Centro de Innovación en Química Avanzada (ORFEO-CINQA) and Institute of Organic Synthesis, 16718 Universidad de Alicante, Ctra. Alicante-San Vicente s/n, 03080 Alicante, Spain; ‡ Departamento de Química Orgánica I and Centro de Innovación en Química Avanzada (ORFEO−CINQA), University of the Basque Country (UPV/EHU), P° Manuel Lardizabal 3, 20018 Donostia/San Sebastián, Spain; § Donostia International Physics Center (DIPC), P° Manuel Lardizabal 4, 20018 Donostia/San Sebastián, Spain; ∥ IKERBASQUE, Basque Foundation for Science, E-48013 Bilbao, Spain

## Abstract

A highly enantioselective, organocatalytic, diastereodivergent synthesis of spiro-fused coumarin derivatives via trienamine-mediated Diels–Alder reactions with cyclic 2,5-dienones is reported. Using a cinchonidine-based primary amine and either *p*-methylbenzoic acid or triethylamine enables reversible diastereoselectivity, providing complementary enantioenriched diastereoisomers. The influence of electron-withdrawing groups at C3 of coumarins is delineated, revealing distinct reactivity patterns. Computational studies provide insight into the mechanism and additive-controlled diastereodivergence.

## Graphical abstract

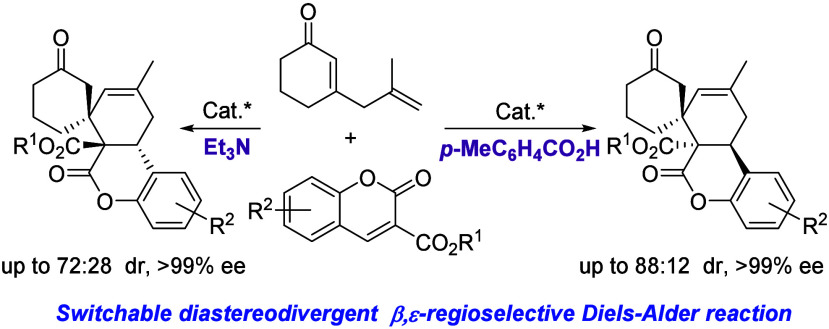

Coumarins constitute a significant class of oxygenated heterocycles commonly found in numerous natural products and biologically active compounds. Various coumarin derivatives demonstrate a broad spectrum of bioactivities, including anticancer, anti-inflammatory, antibacterial, antineurodegenerative, and antitubercular properties.^1^ Due to their versatile application in pharmaceuticals, modifications to the coumarin scaffold could significantly improve their pharmacological properties, leading to new advances in drug discovery. Numerous synthetic methodologies for the development of various coumarins and their derivatives have been reported.^2^ Coumarins bearing a suitable electron-withdrawing group at position C3 have been employed by several research groups as starting material to build more complex coumarin derivatives via 1,4-conjugated addition,^3^ [2+*n*][Bibr ref4] and [3+*n*][Bibr ref5] annulation reactions, cyclopropanations,^6^ and cycloadditions.^7^ However, spiro-fused coumarins have rarely been synthesized, and only a few examples have been described.^5e,5f,6g^ On the other hand, our research group has recently developed an enantioselective synthesis of spirocyclic compounds by Diels–Alder and aldol/cyclization reactions via trienamine employing δ-substituted 2,5-dienones (Scheme 1a).^8^ In this context, continuing with the elaboration of complex chiral organic entities and considering that coumarins are found in several molecules with biological activity, we envisioned that the use of coumarins with a suitable electron-withdrawing group at position C3 would increase the reactivity of the coumarin double bond, allowing the formation of sterically hindered spiro-fused coumarins via a Diels–Alder reaction (Scheme 1b).

**Scheme 1:**
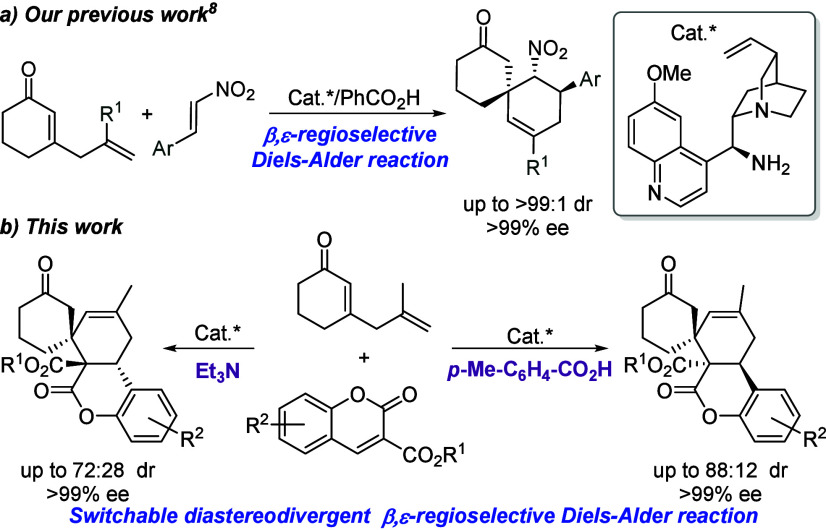
Synthesis of Spirocyclic Adducts from 2,5-Dienones

Based on the above considerations, the reaction of 2,5-dienone 1 and ethyl 3-coumarincarboxylate 2a was evaluated. The reaction was carried out using different organocatalysts with BA as additive in toluene at room temperature (Table 1, entries 1–6). The proline derivative **I** afforded the desired spiro-fused coumarins in good conversion as a mixture of diastereoisomers, with moderate enantiomeric excesses for both diastereoisomers (Table 1, entry 1). In contrast, the bifunctional amine-sulfonamide II and amine-thioureas **III** and **IV** gave rise to the desired adducts with high conversions and enantiomeric excesses but low diasterediastereoselectivities (Table 1, entries 2–4).Moreover, whereas quinine derivative **V** provided spirocyclic adduct **3a** with high diastereoselectivity, quinidine derivative **VI** led to the formation of a mixture of spirocycles **3a** and **3a′** with almost no diastereoselectivity (Table 1, entries 5 and 6). Further optimization was carried out with the best organocatalyst **V** using different amounts of the catalyst and reagents, and a variety of solvents (see the Supporting Information for details). Although these changes had a minimal impact on the reaction, the choice of additive emerged as a critical factor influencing the reaction outcome (Table 1, entries 7–10). While benzoic acid derivatives led to spirocyclic adducts with similar diastereomeric ratios (Table 1, entry 5 vs entries 7 and 8), the use of phenol and triethylamine resulted in lower diastereoselectivities (Table 1, entries 9 and 10, respectively). Notably, when both triethylamine and phenol were used as additives (Table 1, entry 10), **3a′** was obtained as the major diastereoisomer with a large enantiomeric excess. Finally, employing *p*-methylbenzoic acid (*p*-Me-BA) as the additive, the reaction was scaled up to 0.2 mmol, affording the desired cycloadduct **3a** with a high diastereomeric ratio and excellent enantiomeric excess (Table 1, entry 11).

**Table 1:**
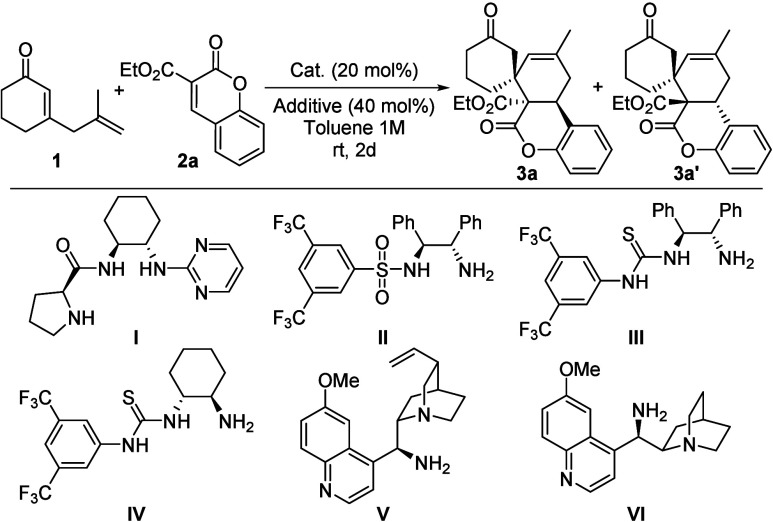
Catalyst and Additive Screening for the β,ε-Regioselective [4+2] Cycloaddition^a^

entry	catalyst	additive	conversion (%)^b^	**3a**:**3a′** ^c^	ee of **3a** (%)^d^	ee of **3a′** (%)^d^
1	**I**	BA	76	38:62	–73	–67
2	**II**	BA^e^	80	48:52	96	90
3	**III**	BA^f^	93	47:53	97	92
4	**IV**	BA^f^	>99	62:38	91	64
5	**V**	BA	95	80:20	96	80
6	**VI**	BA	>99	56:44	–95	–71
7	**V**	SA	95	85:15	91	17
8	**V**	*p*-Me-BA	97	82:18	>99	80
9	**V**	PhOH	55	38:62	80	90
10	**V**	Et_3_N^g^	30	36:64	83	90
11^h^	**V**	*p*-Me-BA	>99	82:18	>99	80

a Reactions performed with ketone **1** (0.2 mmol), ethyl 3-coumarincarboxylate **2a** (0.1 mmol), a catalyst (20 mol %), and BA (40 mol %) in toluene (100 μL, 1 M) at rt. Abbreviations: BA, benzoic acid; *p*-Me-BA, *p*-methylbenzoic acid.

b Conversions measured by ^1^H NMR after 2 days.

c Diastereomeric ratios measured by ^1^H NMR.

d Enantiomeric excesses measured by HPLC. Negative values indicate the opposite enantiomer.

e With 20% BA.

f With 10% BA.

g With 20% Et_3_N.

h Performed with ketone **1** (0.4 mmol), ethyl 3-coumarincarboxylate **2a** (0.2 mmol), a catalyst (20 mol %), and *p*-Me-BA (40 mol %) in toluene (200 μL) at rt.

Having determined the best reaction conditions, we explored the general nature of this process. The results of a series of experiments are summarized in Scheme 2. First, a wide variety of aryl-substituted ethyl 3-coumarincarboxylates **2b**–**n** bearing electron-donating and -withdrawing groups were tested. The reaction proceeded smoothly with coumarins containing electron-donating groups, affording cycloadducts **3b–g** in moderate to high yields with high diastereoselectivities and excellent enantiomeric excesses. Coumarins bearing electron-withdrawing groups on the aromatic ring were also tolerated. Whereas electron-withdrawing substituents at positions 1 and 3 afforded cycloadducts **3h** and **3i** in moderate yields and diastereoselectivities with excellent enantiomeric excesses, substituents at positions 2 and 4 afforded cycloadducts **3j–n** in good yields and diastereoselectivities with excellent enantiomeric excesses. More sterically hindered coumarin **2o** proved to be amenable for this reaction, and expected product **3o** could be obtained in 75% yield and 96% ee. On the other hand, isopropyl 3-coumarincarboxylate **2p** was also tolerated, leading to the desired spirocycle **3p** in moderate yield, high diastereomeric ratio, and excellent enantiomeric excess. To demonstrate the synthetic value of this methodology, the model reaction was performed at a 1 mmol scale for the synthesis of **3a** (73%, 82:18 dr, 97% ee) (see the Supporting Information). Adduct **3a** was crystallized, and its absolute 6a*R*,7*S*,10a*S* configuration was unequivocally confirmed by XRD analysis, assuming the same absolute configuration for other products **3**.

**Scheme 2:**
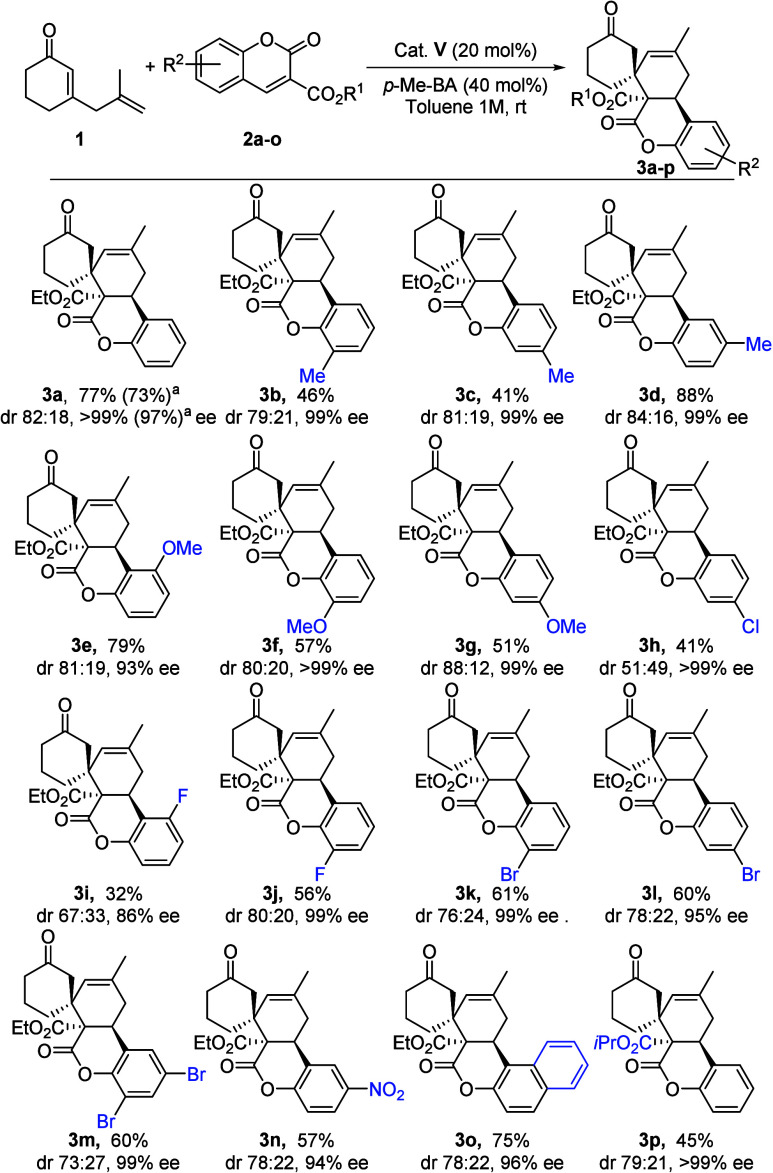
Substrate Scope of **3** ^b^

a At 1 mmol scale.
b Yields of isolated diastereoisomer **3**.

As previously mentioned, the use of triethylamine as an additive resulted in **3a′** being the major diastereoisomer. To further investigate the diastereodivergence of the process, alternative reaction conditions were evaluated using different bases as additives (Table 2 and the Supporting Information). Initially, the reaction was evaluated using quinine **V** and quinidine **VI** derivatives as catalysts and triethylamine as the additive, obtaining in both cases the desired spirocycle **3a′** as the major diastereoisomer after reaction for 2 days (Table 2, entries 1 and 2, respectively). Although quinidine derivative **VI** produced a higher diastereomeric ratio compared to that of quinine derivative **V**, its lower conversion after 2 days did not allow the determination of enantioselectivity. Notably, quinine derivative **V** afforded a large enantiomeric excess of **3a′** (Table 2, entry 1). Then, other organic and inorganic bases were evaluated (Table 2, entries 3–5). Similar results in terms of conversion and diastereoselectivity were obtained with DIPEA and Na_2_CO_3_ as with Et_3_N, but in both cases, a lower enantioselectivity was obtained for **3a′** (Table 2, entries 3 and 5 vs entry 1). DBU resulted in higher conversion and diastereoselectivity, but the Michael adduct was obtained as the major product (Table 2, entry 4). On the other hand, an increase in the amount of ketone **1** to 4 equiv allowed the conversion to increase to 73% after reaction for 2 days and >99% ee after reaction for 7 days, providing the desired spirocycle with good diastereomeric ratio and excellent enantiomeric excess (Table 2, entries 6 and 7, respectively). It is noteworthy that the reaction could be carried out without solvent with a slight decrease in enantioselectivity (Table 2, entry 8). Finally, the reaction was scaled to 0.2 mmol, affording the desired cycloadduct **3a′** with a good diastereomeric ratio and excellent enantiomeric excess (Table 2, entry 9).

**Table 2:**
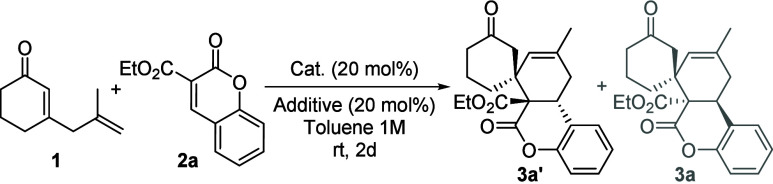
Optimization of the Reaction for the Synthesis of **3a′** ^a^

entry	catalyst	additive	conversion (%)^b^	**3a′**:**3a** ^c^	ee of **3a′** (%)^d^
1	**V**	Et_3_N	30	64:36	90
2	**VI**	Et_3_N	17	73:27	nd
3	**V**	DIPEA	30	64:36	85
4	**V**	DBU	72^e^	79:21	nd
5	**V**	Na_2_CO_3_	31	61:39	76
6^f^	**V**	Et_3_N	73	65:35	95
7^f^	**V**	Et_3_N	>99^g^	61:39	95
8^f^,^h^	**V**	Et_3_N	>99^g^	62:38	90
9^i^	**V**	Et_3_N	>99^g^	61:39	99

a Reactions performed with ketone **1** (0.2 mmol), ethyl 3-coumarincarboxylate **2a** (0.1 mmol), a catalyst (20 mol %), and an additive (40 mol %) in toluene (100 μL, 1 M) at rt.

b Conversions measured by ^1^H NMR after 2 days.

c Diastereomeric ratios measured by ^1^H NMR of the crude reaction.

d Enantiomeric excesses measured by HPLC.

e Mixture of 37% of the Michael product and 35% of a mixture of **3a′** and **3a**.

f Performed using 0.4 mmol of ketone **1**.

g Measured after 7 days.

h Performed without a solvent.

i Performed with ketone **1** (0.8 mmol), ethyl 3-coumarincarboxylate **2a** (0.2 mmol), catalyst **V** (20 mol %), and Et_3_N (20 mol %) in toluene (200 μL, 1 M) at rt.

Having determined the best reaction conditions to synthesize **3a′**, we investigated the general nature of this process (Scheme 3). A selection of aryl-substituted ethyl 3-coumarincarboxylate **2a**–**e** and **2h** bearing electron-donating and -withdrawing groups was successfully evaluated, affording spirocyclic adducts **3a–e′** and **3h′**, respectively, in moderate yields, good diastereoselectivities, and high to excellent enantioselectivities.

**Scheme 3:**
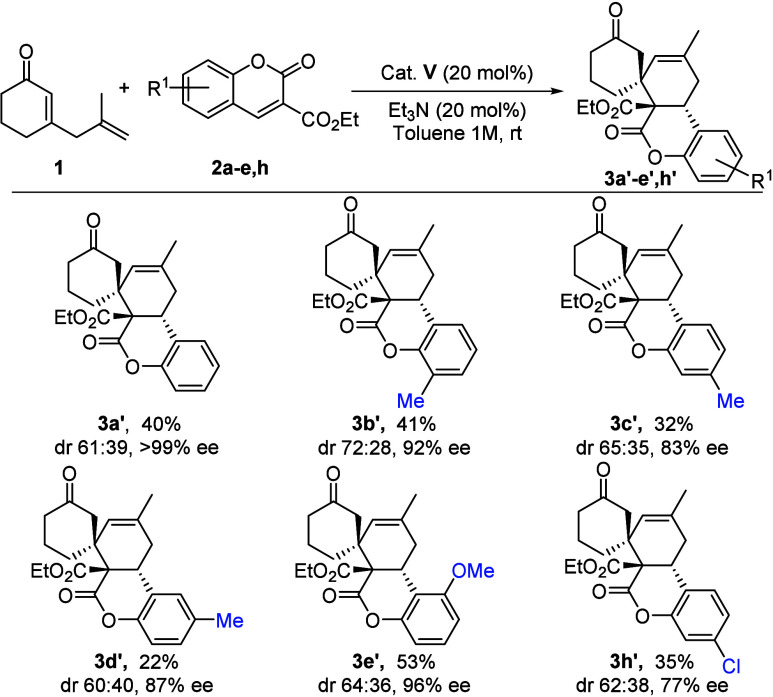
Substrate Scope of **3′** ^a^

a Yields of isolated diastereoisomer **3′**.

In order to understand the insights into the mechanism and its diastereodivergence depending on the additive, DFT calculations at the B3LYP-D3BJ­(SCRF=PCM, toluene)/6-31G­(δ) level of theory were performed (see the Supporting Information for further details). Our calculations indicate that, in an independent manner of the use of an acid additive, the reaction follows the catalytic cycle depicted in Scheme 4. Initially, **1a** interacts with the catalyst to generate α,β-unsaturated imine intermediate **INT1** that can evolve toward two different trienamines, namely **INT2** and **INT2′**. These latter trienamines can react with π-deficient alkene **2a**, giving rise to final spirocycles **3a** and **3a′**. Remarkably, in both scenarios, the reaction follows a stepwise mechanism in which the first C–C bond formation step (**TS1**) determines the geometry of the final spirocycle.

**Scheme 4:**
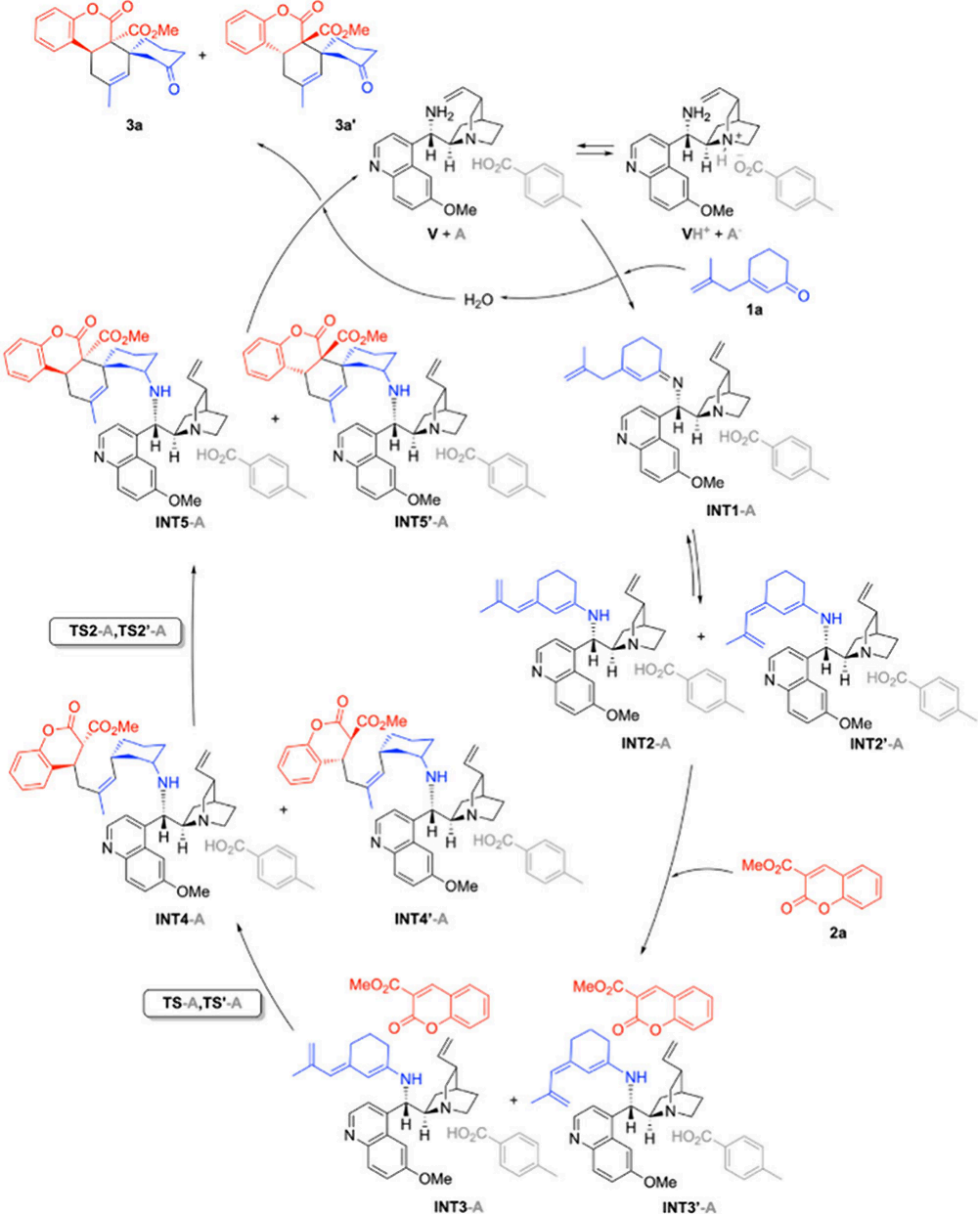
Proposed Catalytic Cycle Associated with the Formation of Spiro [4+2] Cycloadducts **3a** and **3a′** from Ketone **1a** and Alkene **2a** Catalyzed by **V** ^a^

a The presence of the acidic additive (*p*-Me-BA) is indicated as **A**.

In absence of an acid additive, our calculations indicate that unsaturated imine **INT1** can generate trienamines **INT2** and **INT2′**, in which the central double bond of the trienamine adopts a *trans* or *cis* configuration, where the former is ca. 4 kcal mol^–1^ more stable (see the Supporting Information). These trienamines can interact with electro-deficient alkene **2a** to generate reactive complexes **INT3** and **INT3′**. Analogously to the trienamines mentioned above, reactive complex **INT3**, related to the *trans* configuration of the central double bond of the trienamine moiety, is the most stable one (Figure 1). In line with that trend, the most stable transition structures are related to that *trans* configuration. Our calculations indicate that the diastereoselectivity arises from the *endo* or *exo* approach of **2a** to trienamine **INT3**. In fact, transition structure **TS1** *-exo*, related to the formation of **3a′**, is 1.4 kcal mol^–1^ more stable than its *endo* analogue, **TS1**, related to **3a** formation. We hypothesize that this effect can be ascribed to the free rotation of the N–C­(sp^3^) bond of the quinine moiety that could easily adapt to both approaches, thus being capable of allocating both the bicyclic moiety of catalyst **V** and the methoxycarbonyl group of coumarin **2a**. After the initial C–C bond formation, transient zwitterionic intermediate **INT4**-*exo* can form a ring closure transition structure (namely **TS2**-*exo* in Figure 1) to generate spirocyclic intermediate **INT5′**.

**Figure 1:**
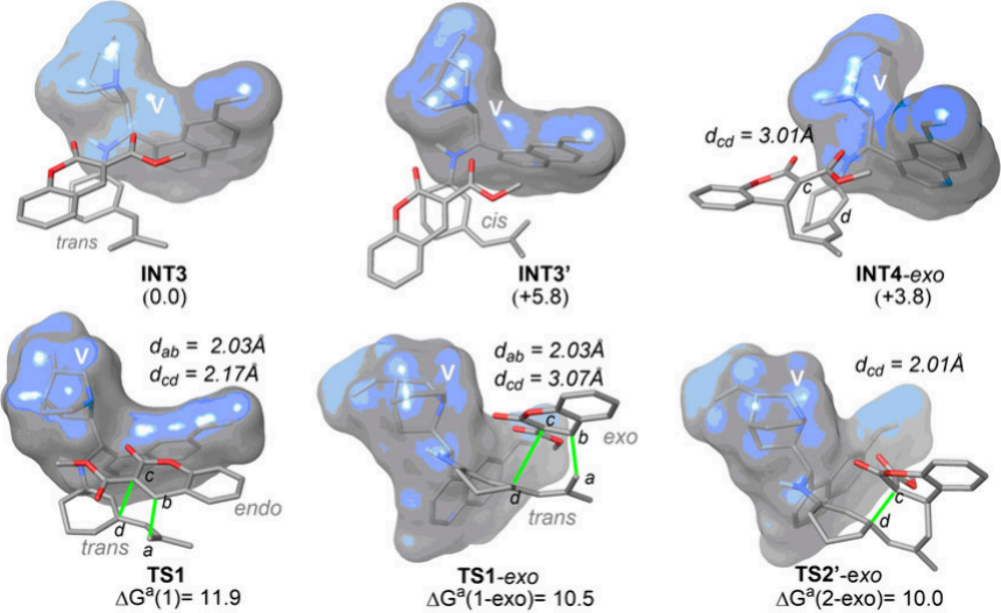
Main geometrical features of the stationary points associated with the formation of spiro [4+2] cycloadducts **3a** and **3a′** from ketone **1a** and alkene **2a** catalyzed by **V** computed at the B3LYP-D3BJ­(SCRF=PCM,toluene)/6-31G­(d) level of theory. Relative Gibbs energies and distances are given in kilocalories per mole and angstroms, respectively. A schematic representation of the stationary points is included in the Supporting Information.

The computed energetic difference in the rate-determining step of the reaction is related to a theoretical **3a**:**3a′** ratio of 10:90, with **3a′** being the major diastereoisomer. Moreover, the theoretical TOF value, related to the effective “activation” energy of the catalytic cycle δG (see the Supporting Information), is 1.2 h^–1^.

With respect to the acid additive (Figure 2), the presence of *p*-methyl benzoic acid strongly affects the geometry of the stationary points and the energetic profile of the catalytic cycle. This additive engages in H-bonding interactions with the nitrogen atom of the quinuclidine moiety, increasing the rigidity of the system. Consequently, the energetic difference between **INT2-A** and **INT2′-A** is reduced to only 1.6 kcal mol^–1^ (contrary to the ca. 4 kcal mol^–1^ energetic difference obtained for the absence of an additive case). Interaction with **2a** leads to the formation of reactive complexes **INT3-A** and **INT3′-A**, which are also close in energy (difference of ca. 2 kcal mol^–1^). Since both reactive complexes are energetically accessible, in this scenario transition structures associated with the two configurations of the trienamine moiety were explored. It is noteworthy that these H-bonding interactions provide a framework that enhances the reactivity of alkene **2a** via HOMO increasing activation and determines the **2a** approach. That dual activation is reflected in the lower computed Gibbs activation barrier as compared with the absence of the additive case (10.5 and 9.4 kcal mol^–1^ for **TS1-** *exo* and **TS1-A**, respectively).

**Figure 2:**
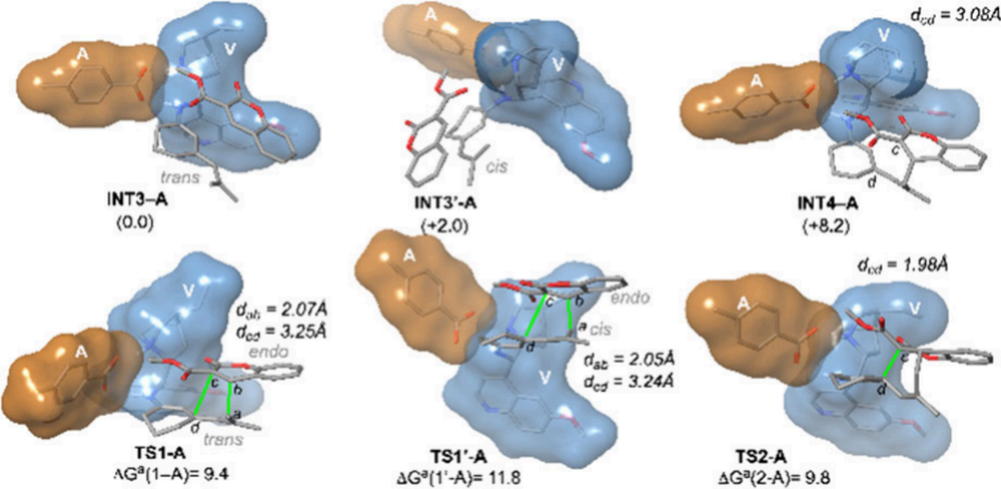
Main geometrical features of the stationary points associated with the formation of spiro [4+2] cycloadducts **3a** and **3a′** from ketone **1a** and alkene **2a** catalyzed by **V** in the presence of an acidic additive (*p*-Me-BA, denoted as **A**). See the legend of Figure 1 for further details.

In this case, least energetic transition structures **TS1-A** and **TS1′-A** correspond to the *endo* approach of **2a** to *trans* and *cis* trienamines, respectively. In fact, all attempts to isolate transition structures related to **2a**-*exo* were unsuccessful. Remarkably, in contrast to the previous case, the presence of the acid additive favors the formation of spirocycle **3a**. Analogously, once transient zwitterionic intermediate **INT4-A** is formed, it can evolve toward spirocyclic cycloadduct **INT5-A**. However, in this case, the computed Gibbs activation barrier of this second step is slightly higher than that obtained for the initial C–C bond formation.

Since formation of the final cycloadducts is related to the existence of different energetically accessible reactive complexes, we have considered a Kurtin–Hammet kinetic scenario for the estimation of the theoretical diastereoselectivity (see the Supporting Information). Within that framework, the computed energetic difference would lead to a higher theoretical diastereoselectivity toward **3a** formation than that observed for **3a′** in the absence of acid (**3a**:**3a′** ratio of 97:3), in qualitative agreement with the experimental evidence. Additionally, the computed TOF is 0.1 h^–1^, thus indicating enhancement of the catalytic performance due to the presence of the acid additive.

Apart from 3-coumarincarboxylates **2**, we also evaluated enantioselective β,ε-regioselective [4+2] cycloadditions with other coumarins bearing different electron-withdrawing groups at position C3 (Scheme 5). While 3-benzoylcoumarin **4** gave rise to the desired adduct in good yield and moderate diastereomeric ratio as a racemic mixture of major diastereoisomer **5**, 3-nitrocoumarin **6** gave rise to spirocyclic adduct **7′** in good diastereomeric ratio and moderate yield and enantioselectivity. Notably, the major diastereoisomer obtained from 3-nitrocoumarin **6** shares the same relative configuration as the minor diastereoisomer observed in reactions with 3-coumarin carboxylates. Due to the moderate enantiomeric excess obtained for **7′**, the absolute configuration has to be determined by electronic circular dichroism (ECD) (see the Supporting Information).

**Scheme 5:**
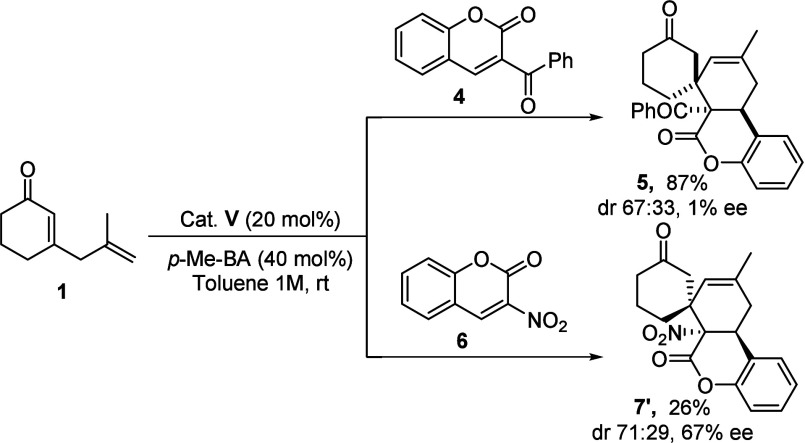
Cycloadditions between 2,5-Dienone **1** and Coumarins **4** and **6**

In conclusion, this work describes a synthetic diastereodivergent strategy to obtain chiral spiro-fused coumarin compounds with three stereogenic centers via trienamine catalysis in a Diels–Alder reaction. By using a cinchonidine-based primary amine as the catalyst and *p*-methylbenzoic acid or triethylamine as cocatalysts, the diastereodivergent construction of a wide variety of spiro-fused coumarins has been achieved. The addition of benzoic acid or triethylamine as a cocatalyst could effectively switch the diastereoselectivity. Additionally, the importance of an ester group at position C3 of the coumarins has been demonstrated by using other electron-withdrawing groups, which show different behaviors. Computational studies have provided insight into the mechanism and diastereodivergence of the reaction, depending on the additive used.

## Supplementary Material



## Data Availability

The data underlying this study are available in the published article and its Supporting Information.

## Notes

Experimental details, characterization data, DFT calculations, and NMR spectra (PDF)
