# Supplementary material for: Diastereodivergent and Enantioselective Organocatalytic Synthesis of Spiro-Fused Coumarins via [4+2] Cycloadditions
Source: Org Lett. 2026 Jan 29;28(6):2069–75. doi: 10.1021/acs.orglett.5c05358 (PMC12910717; doi:10.1021/acs.orglett.5c05358)
Supplement: Supplementary file 1 [file ol5c05358_si_001.pdf]

Diastereodivergent and Enantioselective Organocatalytic Synthesis of Spiro-Fused Coumarins via [4+2] Cycloadditions

Raquel Hidalgo-León,† José Trujillo-Sierra,† José Miguel Sansano,† Fernando P. Cossío,|| Abel de Cozar,,||,§ and María de Gracia Retamosa*,†*

† Departamento de Química Orgánica, Centro de Innovación en Química Avanzada (ORFEO-CINQA) and Institute of Organic Synthesis. Universidad de Alicante. Ctra. Alicante-San Vicente s/n, 03080-Alicante, Spain.

|| Departamento de Química Orgánica I and Centro de Innovación en Química Avanzada (ORFEO-CINQA), University of the Basque Country (UPV/EHU), Pº Manuel Lardizabal 3, 20018 Donostia/San Sebastián, Spain; Donostia International Physics Center (DIPC), Pº Manuel Lardizabal 4, 20018 Donostia/San Sebastián, Spain

§ IKERBASQUE, Basque Foundation for Science, E-48013, Bilbao, Spain.

Table of Contents

1 General remarks. S1

2. Optimization of the reaction conditions. S2

 2.1 Optimization of the reaction conditions to obtain **3a**. S2

 2.2 Optimization of the reaction conditions to obtain **3a’**. S4

3 Synthesis of coumarins **2a-p**. S6

4 General procedures for the synthesis of spirocycles. S7

5 Spirocyclic characterization. S8

 5.1 Spirocyclic characterization **3a-3p**, **5** and **7** in the standard conditions. S8

 5.2 Spirocyclic characterization of **3a’-3e’** and **3h’**. S20

6 NMR Spectra. S24

 6.1 NMR Spectra of **3a-3p**, **5** and **7**. S24

 6.2 NMR Spectra of **3a’-3e’** and **3h’**. S45

7 X-Ray Diffraction Analysis. 51

 7.1 X-Ray Diffraction Analysis of **3a** (CCDC 2395019). S51

 7.2 X-Ray Diffraction Analysis of **3h’** (CCDC 2395020). S52

8 Electronic Circular Dicrhoism of **7**. S54

9. Computational details. S55

1 General remarks.

Unless otherwise noted, reagents and substrates were purchased from commercial suppliers. Substituted ketone **1a** was prepared according to literature.[1,2] Catalysts **I**[3], **II**[4], **III**[5], **IV**[6], **V**, **VI**[7], **VII**[8], **VIII**[9] and **IX**[10] were prepared following literature procedures. Coumarin derivatives synthesis are described in section 3.

Analytical TLC was performed Schleicher & Schuell F1400/LS 254 silica gel plates, and the spots were visualized under UV light (λ = 254 nm). Flash column cromatography was carried out on column silica gel 60 Å (particle size 40-60 µm).

Melting points were determined with a Reichert Thermovar hot plate apparatus and are uncorrected. Optical rotations were measured on a JASCO P-1030 or JASCO DIP-1000 polarimeter with a thermally jacketed 5 cm cell at approximately 25 °C and concentrations (c) are given in g/100 mL.

The structurally most important peaks of the IR spectra (recorded using a Nicolet 510 P-FT) are listed, and wavenumbers are given in cm^{-1}.

NMR spectra were obtained using a Bruker AC-300 or AC-400 and were recorded at 300 or 400 MHz for ^{1}H NMR and 75 or 101 MHz for ^{13}C NMR, using $CDCl_3$ as solvent and TMS as internal standard (0.00 ppm) unless otherwise stated. The following abbreviations are used to describe peak patterns where appropriate: s = singlet, d = doublet, t = triplet, q = quartet, dd = doublet of doublets, m = multiplet or unresolved and br s = broad signal. All coupling constants (*J*) are given in Hertz (Hz) and chemical shifts in ppm. ^{13}C NMR spectra were referenced to $CDC1_3$ at 77.16 ppm.

Low-resolution electron impact (EI) mass spectra were obtained using a Agilent 5977B/MSD by injection or DIP; fragment ions in m/z are given with relative intensities (%) in parentheses. High-resolution mass spectra (HRMS) were measured on an instrument using a quadrupole time-of-flight mass spectrometer (QTOF) and also through the electron impact mode (EI) at 70 eV using a Finnigan VG Platform or a Finnigan MAT 95S.

Enantioselectivities were measured by HPLC JASCO using chiral stationary phases. In these experiments the racemic mixtures were analysed to establish the enantiomeric parameters of each enantiomer. In all cases, the enantiomeric excess of each compound is measured after it´s purification by colum chromatography. X-ray crystal structure was determined using a Bruker CCD-Apex.

---

[1] Hénon, H.; Mauduit, M.; Alexakis, A. *Angew. Chem. Int. Ed*. **2008**, *47*, 9122-9124.
[2] a) Woods, G. F.; Tucker, I. W. *J. Am. Chem. Soc*. **1948**, *70*, 2174. b) Woods, G. F.; Griswold, P. H.; Armbrecht, B. H.; Blumenthal, D. I.; Plapinger, R. *J. Am. Chem. Soc*. **1949**, *71*, 2028.
[3] P. Vizcaíno-Milla; J. M. Sansano; C. Nájera; B. Fiser; E. Gómez-Bengoa. *Eur. J. Org. Chem*. **2015**, 2614-2621.
[4] Yu, F.; Sun, X.; Jin, Z.; Wenm S.; Liang, X.; Ye, J. *Chem. Commun*. **2010**, *46*, 4589-4591.
[5] Yu, F.; Jin, Z.; Huang, H.; Ye, T.; Liang, X.; Ye, J. *Organic & Biomolecular Chemistry*. **2010**, *8* (20), 4767-4774.
[6] Acaso-Alegre, C.; Herrera, R. P.; Mangas-Sánchez, J. *Angew. Chem. Int. Ed*. **2022**, *61*, e202209159.
[7] T. Peňaška; V. Palchykov; E. Rakovský; G. Addová; R. Sebesta. *Eur. J. Org. Chem*. **2021**, 1693-1703.
[8] Castelló, L. M.; Nájera, C.; Sansano, J, M.; Larrañaga, O.; Cózar, A.; Cossío, F. P. *Adv. Synth. Catal*. **2014**. *356*, 3186-3870.
[9] Puglisi, A.; Benglia, M.; Annunziata, R.; Rossi, D. *Tetrahedron Asymmetry*. **2008**, *19*, 2258-2264.
[10] Vizcaíno-Milla, P.; Sansano, J. M.; Nájera, C.; Fiser, B.; Gómez-Bengoa, E. *Eur. J. Org. Chem*. **2015**, 2614-2621.

2. Optimization of the reaction conditions.

2.1 Optimization of the reaction conditions to obtain 3a.

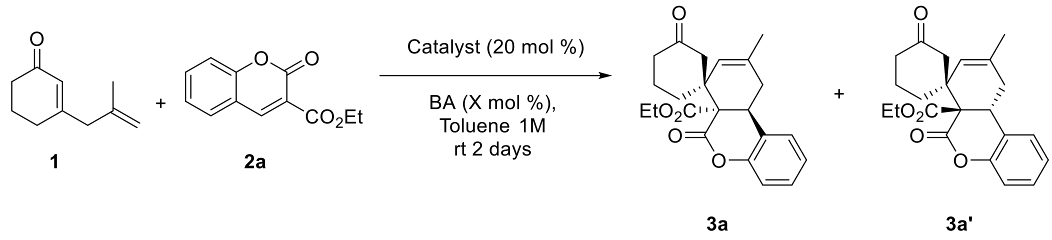

Scheme S1. General reaction to perform the optimization of **3a**.

To perform the catalyst screening, a reaction mixture of 3-(2-methylallyl)cyclohex-2-en-1-one **1** (0.2 mmol), ethyl 3-coumarincarboxylate **2a** (0.1 mmol), different organocatalysts (0.02 mmol) with benzoic acid (% mmol specified in each case) was stirred at room temperature, and the conversion was determined by ^{1}H-NMR after 2 days. . TLC plates were stained with vanillin. The reactions were heated in a sand bath when needed.

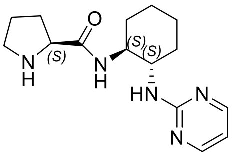

I
BA (40 mol %)
76% conversion
3a : **3a'**
38 (-73% *ee*) : 62 (-67% *ee*)

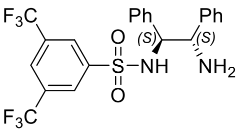

II
BA (10 mol %)
80% conversion
3a : **3a'**
48 (96% *ee*) : 52 (90% *ee*)

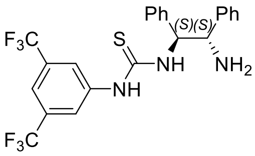

III
BA (10 mol%)
93% conversion
3a : **3a'**
47 (97% *ee*) : 53 (92% *ee*)

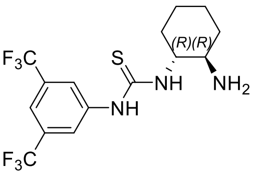

IV
BA (10 mol %)
full conversion
3a : **3a'**
62 (91% *ee*) : 38 (64% *ee*)

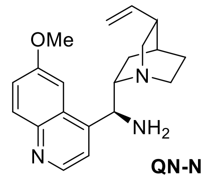

QN-NH$_2$

V
BA (40 mol %)
95% conversion
3a : **3a'**
80 (96% *ee*) : 20 (80% *ee*)

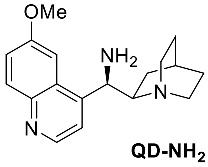

QD-NH$_2$

VI
BA (40 mol %)
full conversion
3a : **3a'**
56 (-95% *ee*) : 44 (-71% *ee*)

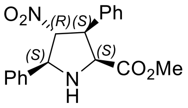

VII
BA (10 mol %)
45% conversion
3a : **3a'**
61 (-35% *ee*) : 39 (-23% *ee*)

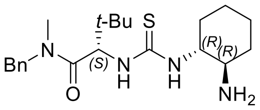

VIII
BA (10 mol %)
full conversion
3a : **3a'**
43 (-62% *ee*) : 57 (-63% *ee*)

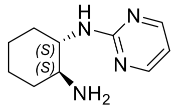

IX
BA (10 mol %)
60% conversion
3a : **3a'**
32 (racemic) : 68 (32% *ee*)

Scheme S2. Catalyst screening.

Table S1. Reaction between ethyl 3-coumarincarboxylate **2a** and 3-(2-methylallyl)cyclohex-2-en-1-one **1** testing a variety of solvents and additives. [a]

Entry	Solvent	Additive	Conv. (%)[b]	Dr 3a : 3aa'	*ee* 3aa : 3a' (%)[c]
**1**	Chloroform	BA	91	70 : 30 [d]	92% *ee* : 78% *ee*
**2**	Toluene	BA	95	80 : 20	96 % *ee* : 80% *ee*
**3**	DCE	BA	92	72 : 28	92% *ee* : 75% *ee*
**4**	TFT	BA	97	73 : 27	95% *ee* : 80% *ee*
**5**	THF	BA	95	74 : 26	91% *ee* : 87% *ee*
**6**	H_2O	BA	96	65 : 35	90% *ee* : 94% *ee*
**7**	Toluene	*m*-OMe-BA	92	79 : 21	80% *ee* : 50% *ee*
**8**	Toluene	SA	95	85 : 15	91% *ee* : 17% *ee*
**9**	Toluene	*p* -Cl-BA	92	77 : 23	96 % *ee* : 49% *ee*
**10**	Toluene	*p*-Me-BA	97	82 : 18	>99% *ee* : 80% *ee*
**11**	Toluene	Et_3N[d]	30	36 : 64	83% *ee* : 90% *ee*
**12**	Toluene	Fenol	55	38 : 62	80% *ee* : 90% *ee*

[a] The reactions were conducted using ketone **1** (0.2 mmol), ethyl 3-coumarincarboxylate **2a** (0.1 mmol) in the presence of catalyst **V** (20 mol %), additives (40 mol %) and solvent (1M). [b] Conversions were measured by ^{1}H-NMR of crude reaction after 2 days. [c] Enantiomeric excesses were measured by HPLC. [d] In this case, additive was in 20 mol %.

Table S2. Reaction between ketone **1** and ethyl 3-coumarincarboxylate **2a** catalyzed by QN-NH_2 **V** evaluating different concentrations and equivalents of ketone **1a** and organocatalyst **V**.[a],[b]

Entry	Concentration [M]	Equiv. ketone	% Catalyst	Conv. (%)	Dr 3a : 3a'	*ee* 3a : 3a' (%)[c]
**1**	1	2	20	97	82 :18 [d]	>99 % *ee* : 80% *ee*
**2**	2	2	20	>99	79 : 21	96% *ee* : 69% *ee*
**3**	0.5	2	20	91	77 : 23	96% *ee* : 62% *ee*
**4**	1	2.5	20	>99	75 : 25	95% *ee* : 68% *ee*
**5**	1	1.5	20	97	75 :25	96% *ee* :72% *ee*
**6[d]**	1	1.5	20	97	50 : 50	91% *ee* : 94% *ee*
**7**	1	2	15	77	75 :25	97% *ee* : 78% *ee*

[a] The reactions were conducted using ketone **1**, ethyl 3-coumarincarboxylate **2a** (0.1 mmol) in the presence of organocatalyst **V** and *p*-Me-BA (40 mol %) using toluene as solvent. [b] Reactions were monitorized by ^{1}H-NMR and allowed to stir at room temperature. [c] Enantiomeric excesses measured by HPLC. [d] Reaction carried out at 40 °C.

2.2 Optimization of the reaction conditions to obtain 3a'.

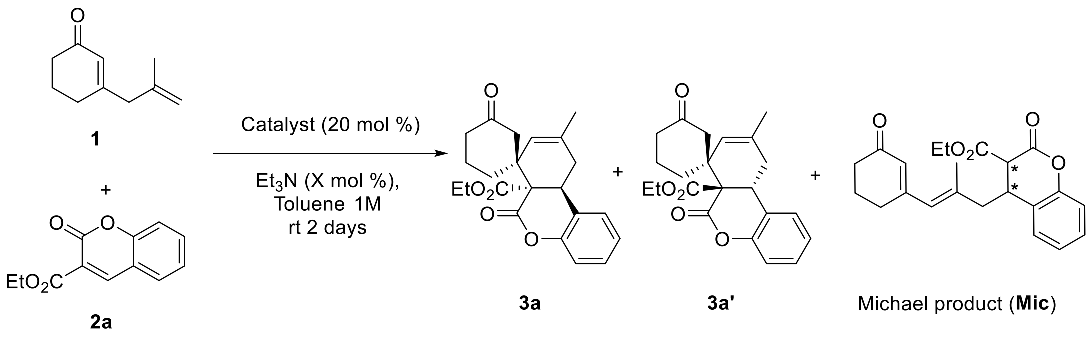

Scheme S3. General reaction to perform the optimization of **3a'**.

To perform optimization, a reaction mixture of 3-(2-methylallyl)cyclohex-2-en-1-one **1** (0.2 mmol), ethyl 3-coumarincarboxylate **2a** (0.1 mmol), different organocatalysts (0.02 mmol) with different additives (mol % specified in each case) was stirred at room temperature, and the conversion was determined by ^{1}H-NMR after 2 days. TLC plates were stained with vanillin. In the following experiments of Table S3, three products are observed in all cases in different proportions **3a**, **3a'** and the Michael-type product (abbreviated as **Mic** in Table S3).

Table S3. Reaction between ethyl 3-coumarincarboxylate **2a** and 3-(2-methylallyl)cyclohex-2-en-1-one **1** testing a different additives and other parameters to optimize **3a'** as the major diastereoisomer. [a]

Entry	Additive	Days	% Additive	Conv. (%)[b]	Mic : 3a' : 3a	Dr 3a' : 3a	*ee* 3a'[c]
**1**	Et_3N	2	20	30	11 : 57 : 32	64 : 36	90% *ee*
**2**	Et_3N[d],[e]	2	20	88	10 : 42 : 48	47 : 53	Nd
**3**	Et_3N[e],[f]	2	20	40	16 : 61 : 23	73 : 27	71 % *ee*
**4**	Et_3N[f][g]	7	20	23	0 : >99 : 0	>99 : 1	93% *ee*
**5**	Fenol	2	40	55	10 : 56 : 34	62 : 38	90% *ee*
**6**	Et_3N[e]	2	20	54	10 : 52 : 39	57: 43	Nd
**7**	Et_3N	7	40	42	15 : 51 : 33	61 : 39	Nd
**8**	Fenol[e]	2	40	>99	5 : 40 : 39	50 : 50	Nd
**9**	Fenol	2	80	66	10 : 56 : 34	62 : 38	Nd
**10**	Et_3N[g]	2	20	73	10 : 58 : 32	65 : 35	95% *ee*
**11**	Et_3N[g]	7	20	>99	5 : 58: 37	61 : 39	95% *ee*
**12**	Et_3N[g],[h]	7	20	>99	11 : 55 : 34	62 : 38	90% *ee*
**13**	$(iPr)_2NEt$	2	20	30	9 : 59 : 32	64 : 36	85% *ee*
**14**	H_2O	2	30	60	12 : 50 : 38	57 : 43	nd
**15**	DBU	2	20	72	52 : 38 : 10	79 : 21	nd
**16**	DBU	5	20	89	43 : 39 : 18	69 : 31	nd
**17**	DBU[i]	7	20	95	15 : 43 : 43	50 : 50	nd
**18**	DABCO	2	20	23	18 : 50 : 32	61 : 39	nd
**19**	Na_2CO_3	2	20	31	12 : 54 : 34	61 : 39	76% *ee*

[a] The reactions were conducted using ketone **1** (0.2 mmol), ethyl 3-coumarincarboxylate **2a** (0.1 mmol) in the presence of catalyst **V** (20 mol %), additives (X mol %) and solvent (1M). [b] Conversions were measured by ^{1}H-NMR of crude reaction. [c] Enantiomeric excesses were measured by HPLC. [d] Reaction performed with catalyst **II** (20 mol%]. [e] Reaction performed at 40° C. [f] Reaction performed with catalyst **VI** (20 mol%). [g] Reaction performed using 0.4 mmol of ketone **1**. [h] In this case, no solvent. [i] The first 5 days were at room temperature and then 2 more days at 60° C.

After the results of table S3, the best results were observed in entry 12 when using 4 equivalents ketone **1** with Et_3N at room temperature and without solvent. However, due to the difficulty of solubilizing certain coumarins, it is chosen to keep the solvent in the medium, being entry 11 the final conditions.

3 Synthesis of coumarins 2a-p.

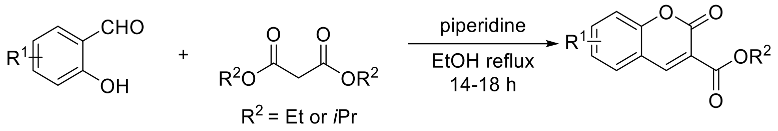

Scheme S4. General reaction to obtain coumarins **2a-p**.

To synthesize ethyl 3-coumarincarboxylate derivatives, in a round bottom flask salicylaldehides derivatives (3.2 mmol, 1 eq) and diethyl malonate (4.9 mmol, 1.5 eq) were dissolved in EtOH (10 mL, 0.32M) as it is described in literature.[11]Then piperidine (0.5 mmol) was added, and the reaction was stirring and heating with a reflux system in an oil bath for 14-18 hours until the conpsumption of the starting materials. Purification of the final coumarins was carried out by recrystallization in EtOH. To obtain isopropyl 2-oxo-2H-chromene-3-carboxylate derivatives the procedure is the same but using diisopropyl malonate in the same equivalents. The experimental data of these substrates are detailed in the cited reference above.

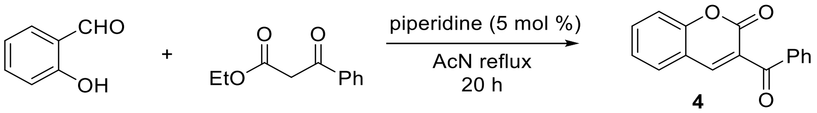

4

Scheme S5. General reaction to obtain coumarin **4**.

As previously described in literature [12], 3-benzoyl-chromen-2-one was synthesized using salicylaldehyde (3.2 mmol, 1.1 eq) and ethyl benzoylacetate (2.9 mmol, 1 eq) dissolved in AcN (10 mL, 0.29M). Then piperidine (0.2 mmol) was added to the mixture and it was stirring and heating with reflux system in an oil bath for 20 hours. After that time, full conversion was observed, and the crude was concentrated under pressure. The final product **4** was purified washing it with small amounts of cold EtOH. The experimental data of this compound is detailed in the cited reference above.

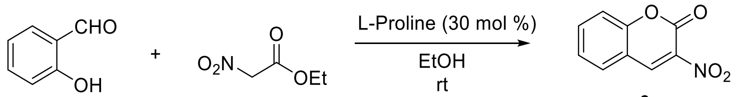

6

Scheme S6. General reaction to obtain coumarin **6**.

To synthesize 3-nitro-2H-chromen-2-one, salicylaldehyde (3 mmol, 1 eq) and ethyl nitroacetate (3 mmol, 1eq) were dissolved in EtOH (9 mL, 0.33M) in a round bottom flask. Then a catalytic amount of L-Pronline (30% mol) was added and the mixture was stirring for 4 hours. After that time, the crude was concentrated under reduced pressure, and it was extracted with $CHCl_3$ x 3. The combination of organic phases was washed with brine, and it was dried over anhydrous $MgSO_4$, filtered and concentrated under reduced pressure. The resulting residue was purified by flash chromatography (Hex/EtOAc 4:1) obtaining the final product 6 as a yellow solid.[13]

[11] Ding, J.; Liu, J.; Zhang, Z.; Cuo, J.; Cheng, M.; Wan, Y.; Wang, R.; Fang, Y.; Guan, J.; Cheng, M.; Wan, Y.; Wang, R.; Fang, Y.; Guan, Z.; Jin, Y.; Xie, S. S. *Bioorganic Chemistry*. **2020**, *101*, 104023.
[12] Röser, K.; Scheucher, A.; Mairhofer, C.; Bechmann, M.; Waser, M. *Org. Biomol. Chem*. **2022**, *20*, 3273-3276.
[13] Sharma, R. K.; Singh, V.; Tiwari, N.; Butcher, R. J.; Katiyar, D. *Bioorganic Chemistry*. **2020**, *98*, 10370.

4 General procedures for the synthesis of spirocycles.

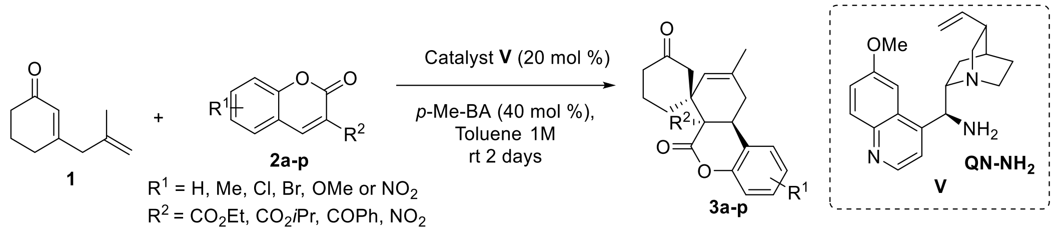

Scheme S7. General procedure to obtain spirocycles **3a-3p**.

Procedure A: A reaction mixture of dienophile **2a-p** (0.2 mmol, 1 eq), ketone **1** (63 µL, 0.4 mmol, 2 eq), organocatalyst **V** (13 mg, 0.04 mmol) and *p*-methyl benzoic acid (11 mg, 0.08 mmol) in toluene (200 µL, 1M) was stirred at room temperature monitored by TLC until the reaction finishes. After this time, the solvent was eliminated under reduced pressure. The crude was washed with saturated $NaHCO_3$, and the organic phase was dried over anhydrous $MgSO_4$ and concentrated under reduced pressure. The resulting residue was purified by flash chromatography in Hex/EtOAc obtaining the desired spiroadducts **3a-p** as white solids. Structural assignments were made with additional information from gCOSY and gHSQC experiments.

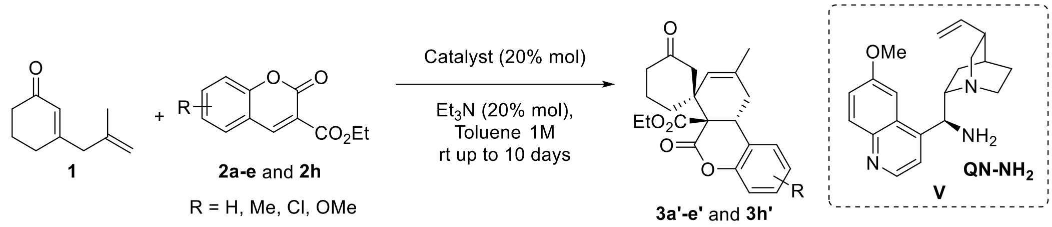

Scheme S8. General procedure to obtain spirocycles **3a'-3e' and 3h'**.

Procedure B: A reaction mixture of dienophile **2a-e and 2h** (0.2 mmol, 1 eq), ketone **1** (126 µL, 0.8 mmol, 4 eq), organocatalyst QN-NH_2 **V** (13 mg, 0.04 mmol) and triethyl amine (11 mg, 0.04 mmol) in toluene (200 µL, 1M) was stirred at room temperature monitored by TLC until the reaction ends. After this time, the solvent was eliminated under reduced pressure. The resulting residue was purified by flash chromatography in Hex/EtOAc obtaining the desired spiroadducts **3a'-e'** and **3h'** as white solids.

Scaled up of 3a.

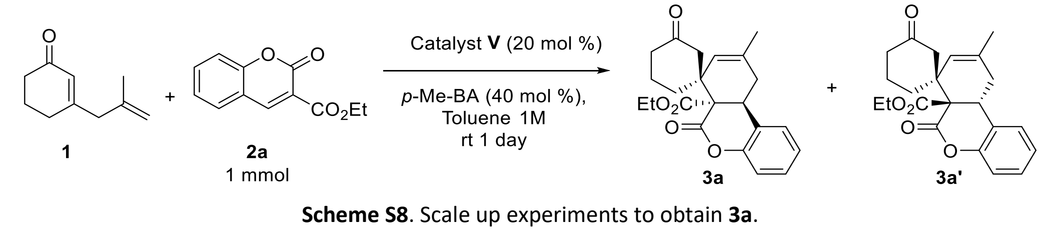

Scheme S8. Scale up experiments to obtain **3a**.

To evaluate the scaling of the reaction, a mixture of dienophile **2a** (220 mg, 1 mmol, 1 eq), ketone **1** (315 µL,2 mmol, 2 eq), organocatalyst **V** (65 mg, 0.2 mmol) and *p*-methyl benzoic acid (55 mg, 0.4 mmol) in toluene (1 mL, 1M) was stirred 1 day. After this time, the reaction has finished and the solvent was eliminated under reduced pressure. The crude was washed with saturated $NaHCO_3$, and the organic phase was dried over anhydrous $MgSO_4$ and concentrated under reduced pressure. The resulting residue was purified by flash chromatography in Hex/EtOAc obtaining the desired spiroadducts **3a** as white solid. The reaction was reproduced several times, affording yields in the range of 70–75%, with an average yield of 73% (235 mg). In all cases, the diastereoselectivity was 82:18, and the enantiomeric excess remained at 97%, matching the values obtained at the 0.2 mmol scale.

5 Spirocyclic characterization.

5.1 Spirocyclic characterization 3a-3p, 5 and 7 in the standard conditions.

Ethyl (6a*R*,7*S*,10a*S*)-9-methyl-3',6-dioxo-10,10a-dihydrospiro[benzo[c]chromene-7,1'-cyclohexane]-6a(6H)-carboxylate, **3a**.

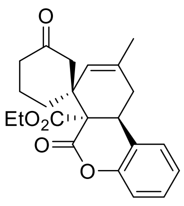

Following the general procedure **A**, starting from ethyl 3-coumarincarboxylate **2a** (44 mg, 0.2 mmol) and 3-(2-methylallyl)cyclohex-2-en-1-one **1** (63 µL, 0.4 mmol), QN-NH_2 **V** (13 mg, 0.02 mmol), 4-methyl benzoic acid (11 mg, 0.04 mmol) and toluene (200 µL, 1M), compound **3a** was obtained as a white solid (56 mg, 77%; reaction ran for 2 days). The diastereoselectivity of the reaction was 82:18. Performed at 1 mmol scale, spirocycle **3a** was isolated (235 mg, 73%, the reaction ran for 1 day), and the crude maintained the same diastereoselectivity. **Mp**: 128-133 °C. **IR** (neat) ν_{max}: 2956, 1776, 1734, 1705, 1460, 1227, 1126, 1028, 760 cm^{-1}. **^{1}H NMR** (300 MHz, $CDCl_3$) δ 7.20 (dtd, *J* = 7.7, 4.2, 1.7 Hz, 2H), 7.10 – 7.02 (m, 1H), 6.96 (dd, *J* = 8.4, 1.2 Hz, 1H), 5.40 (h, *J* = 1.5 Hz, 1H), 4.08 – 3.92 (m, 2H), 3.85 (dd, *J* = 11.1, 7.4 Hz, 1H), 3.74 (dt, *J* = 14.5, 2.3 Hz, 1H), 2.54 – 2.37 (m, 2H), 2.34 (dd, *J* = 14.5, 0.8 Hz, 1H), 2.30 – 2.17 (m, 1H), 2.02 – 1.74 (m, 5H), 1.64 – 1.56 (m, 3H), 0.92 (t, *J* = 7.1 Hz, 3H). **^{13}C NMR** (75 MHz, $CDCl_3$). δ 211.1, 167.8, 164.3, 150.0, 130.7, 128.6, 128.1, 127.6, 125.2, 124.6, 116.6, 62.1, 60.5, 48.6, 44.2, 40.9, 36.3, 35.6, 34.9, 22.9, 20.6, 13.8. **HRMS** (ESI) m/z: [M^+] Calcd for $C_{22}H_{24}O_5$ 368.1624; found 368.1606. The enantiomeric excess was determined by HPLC using a Chiralpak IG column [*n*-hexanes/2-propanol (90:10)]; flow rate 1 mL/min, λ = 210 nm; τ_{minor} = 61.3 min, τ_{major} = 85.3 min (99% ee); **$[\alpha]_D^{25}$** = -92.4 (c 0.8, $CHCl_3$).

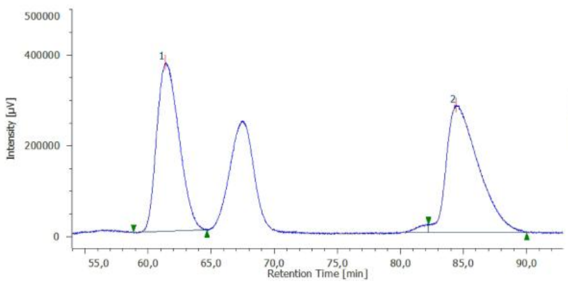

tR [min]	Area [µV·sec]	Height [µV]	Area%	Height%
61,333	45224898	372050	47,814	57,001
84,427	49359743	280655	52,186	42,999

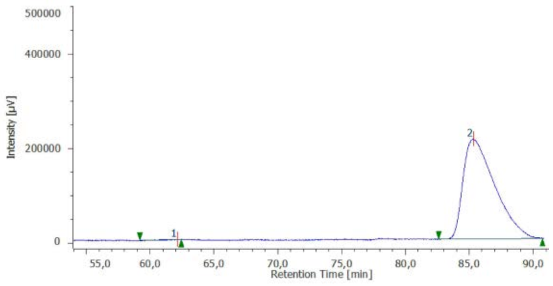

tR [min]	Area [µV·sec]	Height [µV]	Area%	Height%
62,133	13550	804	0,037	0,380
85,320	36291148	210544	99,963	99,620

Ethyl (6a*R*,7*S*,10a*S*)-4,9-dimethyl-3',6-dioxo-10,10a-dihydrospiro[benzo[c]chromene-7,1'-cyclohexane]-6a(6H)-carboxylate, **3b**.

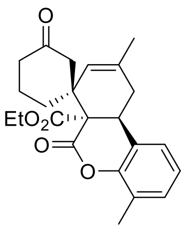

Following the general procedure **A**, starting from ethyl 8-methyl-2-oxo-2H-chromene-3-carboxylate **2b** (46 mg, 0.2 mmol) and 3-(2-methylallyl)cyclohex-2-en-1-one **1** (63 µL, 0.4 mmol), QN-NH_2 **V** (13 mg, 0.02 mmol), 4-methyl benzoic acid (11 mg, 0.04 mmol) and toluene (200 µL, 1M), compound **3b** was obtained as a white solid (35 mg, 46%; reaction ran for 2 days). The diastereoselectivity of the reaction was 79:21. **Mp**: 161-166 °C. **IR** (neat) ν_{max}: 2956, 1776, 1697, 1471, 1437, 1259, 1238, 1192, 1104, 1086, 785 cm^{-1}. **^{1}H NMR** (400 MHz, $CDCl_3$) δ 7.10 – 7.02 (m, 2H), 6.97 (t, *J* = 7.4 Hz, 1H), 5.44 (h, *J* = 1.6 Hz, 1H), 4.09 (dq, *J* = 10.7, 7.1 Hz, 1H), 3.96 (dq, *J* = 10.7, 7.1 Hz, 1H), 3.83 (dd, *J* = 11.1, 7.4 Hz, 1H), 3.81 – 3.75 (m, 1H), 2.49 (ddd, *J* = 7.4, 1.9, 0.9 Hz, 1H), 2.46 – 2.38 (m, 2H), 2.35 (dd, *J* = 14.5, 0.9 Hz, 1H), 2.24 (s, 4H), 2.02 – 1.89 (m, 2H), 1.88 – 1.81 (m, 2H), 1.63 (d, *J* = 1.4 Hz, 3H), 0.94

(t, J = 7.1 Hz, 3H). **^{13}C NMR** (101 MHz, $CDCl_3$) δ 211.3, 168.0, 164.5, 148.3, 130.8, 130.1, 127.9, 126.2, 125.3, 125.2, 124.3, 62.0, 60.5, 48.7, 44.3, 41.0, 36.6, 35.7, 34.9, 23.0, 20.7, 15.6, 13.8. **HRMS** (ESI) m/z: [M^+] Calcd for $C_{23}H_{26}O_5$ 382.1780; found 382.1768. The enantiomeric excess was determined by HPLC using a Chiralpak IG column [*n*-hexanes/IPA (95:5)]; flow rate 1 mL/min, λ = 212 nm; τ_{major} = 81.5 min, τ_{minor} = 94.1 min (99% ee); **[α]$_D^{25}$** = -233.5 (c 1.0, $CHCl_3$).

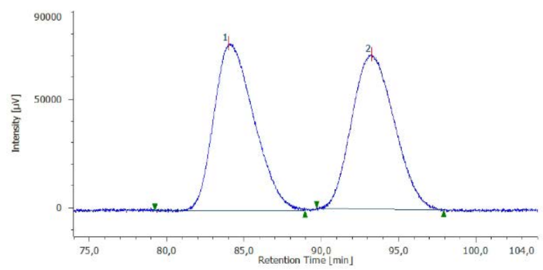

tR [min]	Area [μV·sec]	Height [μV]	Area%	Height%
84,013	13960589	77264	50,556	51,978
93,240	13653746	71384	49,444	48,022

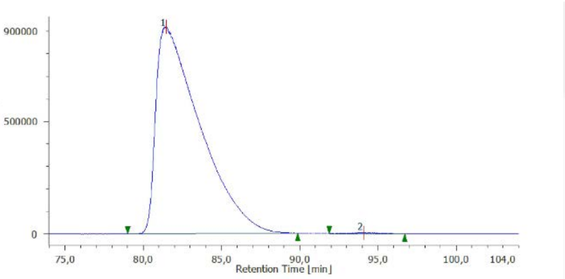

tR [min]	Area [μV·sec]	Height [μV]	Area%	Height%
81,480	180179796	917996	99,687	99,546
94,067	566058	4190	0,313	0,454

Ethyl (6a*R*,7*S*,10a*S*)-3,9-dimethyl-3',6-dioxo-10,10a-dihydrospiro[benzo[c]chromene-7,1'-cyclohexane]-6a(6H)-carboxylate, **3c**.

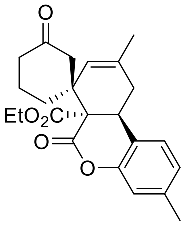

Following the general procedure **A**, starting from ethyl 7-methyl-2-oxo-2H-chromene-3-carboxylate **2c** (46 mg, 0.2 mmol) and 3-(2-methylallyl)cyclohex-2-en-1-one **1** (63 µL, 0.4 mmol), QN-NH_2 **V** (13 mg, 0.02 mmol), 4-methyl benzoic acid (11 mg, 0.04 mmol) and toluene (200 µL, 1M), compound **3c** was obtained as a white solid (31 mg, 41%; reaction ran for 2 days). The diastereoselectivity of the reaction was 81: 19. **Mp**: 148-154 °C. **IR** (neat) ν_{max}: 2958, 1784, 1732, 1705, 1624, 1433, 1221, 1124, 1047, 1032, 825 cm^{-1}. **^{1}H NMR** (400 MHz, $CDCl_3$) δ 7.09 (d, J = 7.7 Hz, 1H), 6.89 (ddd, J = 7.6, 1.7, 0.8 Hz, 1H), 6.80 (dd, J = 1.7, 0.8 Hz, 1H), 5.43 (h, J = 1.6 Hz, 1H), 4.04 (qd, J = 7.1, 3.8 Hz, 2H), 3.82 (dd, J = 11.1, 7.5 Hz, 1H), 3.79 – 3.75 (m, 1H), 2.50 – 2.46 (m, 1H), 2.46 – 2.40 (m, 2H), 2.36 (dd, J = 14.5, 0.9 Hz, 1H), 2.31 (d, J = 0.8 Hz, 3H), 2.29 – 2.20 (m, 1H), 2.00 – 1.91 (m, 1H), 1.91 – 1.79 (m, 3H), 1.63 (q, J = 1.1 Hz, 3H), 1.00 (t, J = 7.1 Hz, 3H). **^{13}C NMR** (101 MHz, $CDCl_3$) δ 211.2, 168.0, 164.6, 150.0, 139.0, 130.9, 127.3, 125.4, 125.3, 125.2, 117.1, 62.1, 60.7, 48.7, 44.3, 41.0, 36.1, 35.7, 35.2, 23.0, 21.3, 20.7, 14.0. **HRMS** (ESI) m/z: [M^+] Calcd for $C_{23}H_{26}O_5$ 382.1780; found 382.1771. The enantiomeric excess was determined by HPLC using a Chiralpak IG column [*n*-hexanes/IPA (60:40)]; flow rate 1 mL/min, λ = 221 nm; τ_{minor} = 12.5 min, τ_{major} = 15.5 min (99% ee); **[α]$_D^{25}$** = -205.4 (c 1.0, $CHCl_3$).

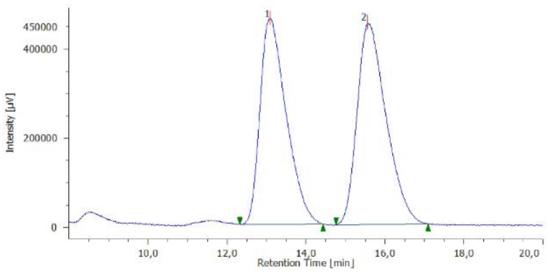

tR [min]	Area [μV·sec]	Height [μV]	Area%	Height%
13,093	21302176	462507	48,051	50,559
15,547	23030200	452278	51,949	49,441

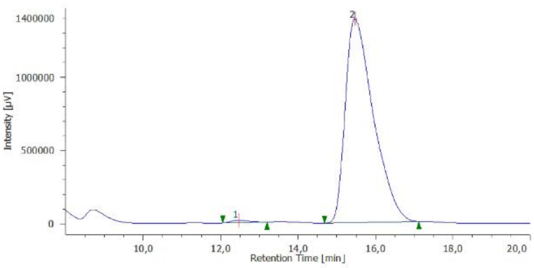

tR [min]	Area [μV·sec]	Height [μV]	Area%	Height%
12,467	536252	16495	0,758	1,172
15,480	70213857	1391483	99,242	98,828

Ethyl (6a*R*,7*S*,10a*S*)-2,9-dimethyl-3',6-dioxo-10,10a-dihydrospiro[benzo[c]chromene-7,1'-cyclohexane]-6a(6H)-carboxylate, **3d**.

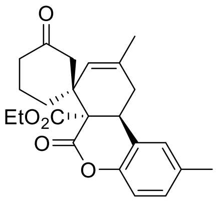

Following the general procedure **A**, starting from ethyl 6-methyl-2-oxo-2H-chromene-3-carboxylate **2d** (46 mg, 0.2 mmol) and 3-(2-methylallyl)cyclohex-2-en-1-one **1** (63 µL, 0.4 mmol), QN-NH_2 **V** (13 mg, 0.02 mmol), 4-methyl benzoic acid (11 mg, 0.04 mmol) and toluene (200 µL, 1M), compound **3d** was obtained as a white solid (67 mg, 88%; reaction ran for 2 days). The diastereoselectivity of the reaction was 84:16. **Mp**: 174-178 °C. **IR** (neat) ν_{max}: 2922, 2854, 1774, 1699, 1496, 1248, 1196, 1142, 814 cm^{-1}. **^{1}H NMR** (400 MHz, $CDCl_3$) δ 7.02 (ddt, *J* = 5.0, 2.8, 2.1 Hz, 2H), 6.88 (d, *J* = 8.8 Hz, 1H), 5.44 (hept, *J* = 1.7 Hz, 1H), 4.10 – 3.99 (m, 2H), 3.84 – 3.80 (m, 1H), 3.80 – 3.76 (m, 1H), 2.49 (ddd, *J* = 7.4, 1.9, 0.9 Hz, 1H), 2.46 – 2.40 (m, 1H), 2.36 (dd, *J* = 14.5, 0.9 Hz, 1H), 2.31 (d, *J* = 0.8 Hz, 3H), 2.29 – 2.20 (m, 1H), 2.00 – 1.88 (m, 3H), 1.88 – 1.80 (m, 2H), 1.63 (q, *J* = 1.0 Hz, 3H), 0.99 (t, *J* = 7.1 Hz, 3H). **^{13}C NMR** (101 MHz, $CDCl_3$) δ 211.2, 168.0, 164.6, 148.0, 134.3, 130.9, 129.2, 128.1, 127.9, 125.4, 116.5, 62.2, 60.6, 48.7, 44.3, 41.0, 36.5, 35.7, 35.1, 23.0, 20.9, 20.7, 14.0. **HRMS** (ESI) m/z: [M^+] Calcd for $C_{23}H_{26}O_5$ 382.1780; found 382.1768. The enantiomeric excess was determined by HPLC using a Chiralpak IG column [*n*-hexanes/IPA (60:40)]; flow rate 1 mL/min, λ = 218 nm; τ_{minor} = 12.2min, τ_{major} = 17.6 min (99% ee); **$[\alpha]_D^{25}$** = -196.9 (c 0.4, $CHCl_3$).

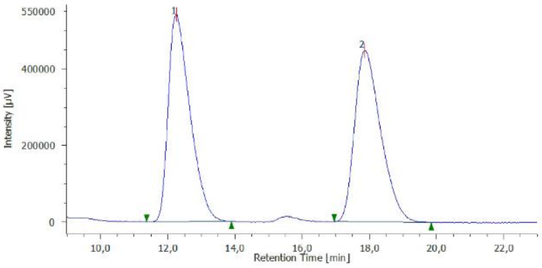

tR [min]	Area [µV·sec]	Height [µV]	Area%	Height%
12,267	24330616	540387	50,190	54,746
17,867	24146144	446696	49,810	45,254

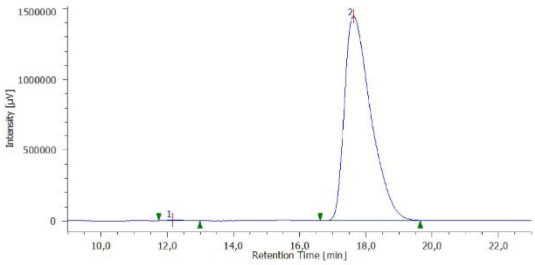

tR [min]	Area [µV·sec]	Height [µV]	Area%	Height%
12,160	242605	6554	0,303	0,452
17,627	79703390	1441946	99,697	99,548

Ethyl (6a*R*,7*S*,10a*S*)-1-methoxy-9-methyl-3',6-dioxo-10,10a-dihydrospiro[benzo[c]chromene-7,1'-cyclohexane]-6a(6H)-carboxylate, **3e**.

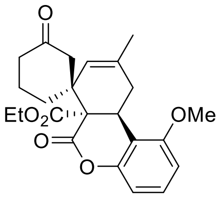

Following the general procedure **A**, starting from ethyl 5-methoxy-2-oxo-2H-chromene-3-carboxylate **2e** (50 mg, 0.2 mmol) and 3-(2-methylallyl)cyclohex-2-en-1-one **1** (63 µL, 0.4 mmol), QN-NH_2 **V** (13 mg, 0.02 mmol), 4-methyl benzoic acid (11 mg, 0.04 mmol) and toluene (200 µL, 1M), compound **3e** was obtained as a white solid (63 mg, 79%; reaction ran for 2 days). The diastereoselectivity of the reaction was 81:19. **Mp**:157-163 ºC. **IR** (neat) ν_{max}: 2959, 1767, 1711, 1468, 1279, 1252, 1211, 1095, 1075, 978, 791 cm^{-1}. **^{1}H NMR** (300 MHz, $CDCl_3$) δ 7.15 (t, *J* = 8.3 Hz, 1H), 6.63 (ddd, *J* = 8.3, 5.5, 0.9 Hz, 2H), 5.41 (q, *J* = 1.7 Hz, 1H), 4.28 (dd, *J* = 11.0, 7.4 Hz, 1H), 4.04 (qd, *J* = 7.1, 1.8 Hz, 2H), 3.87 (s, 3H), 3.76 (dt, *J* = 14.5, 2.4 Hz, 1H), 2.59 – 2.48 (m, 1H), 2.46 – 2.34 (m, 2H), 2.32 – 2.18 (m, 1H), 2.12 – 2.02 (m, 1H), 1.95 (dd, *J* = 6.9, 3.7 Hz, 1H), 1.90 – 1.72 (m, 3H), 1.63 (d, *J* = 1.4 Hz, 3H), 0.98 (t, *J* = 7.1 Hz, 3H). **^{13}C NMR** (101 MHz, $CDCl_3$). δ 211.4, 168.1, 164.5, 156.6, 151.0, 131.1, 128.7, 124.9, 117.3, 109.2, 106.5, 62.1, 60.3, 56.1, 48.8, 44.2, 41.0, 35.7, 33.4, 30.3, 23.1, 20.7, 14.0. **HRMS** (ESI) m/z: [M^+] Calcd for $C_{23}H_{26}O_6$ 398.1729; found 398.1714. The enantiomeric excess was determined by HPLC using a Chiralpak IG column [*n*-hexanes/IPA (60:40)]; flow rate 1 mL/min, λ = 219 nm; τ_{minor} = 13.1 min, τ_{major} = 16.1 min (93% ee); **$[\alpha]_D^{25}$** = -135.4 (c 0.7, $CHCl_3$).

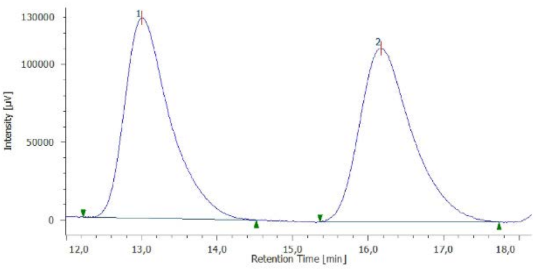

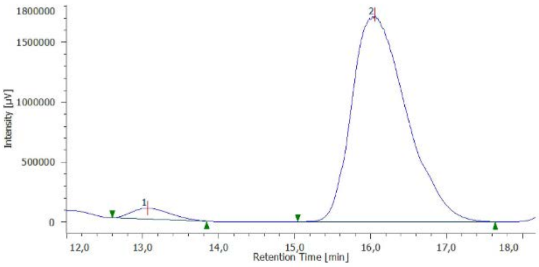

tR [min]	Area [µV·sec]	Height [µV]	Area%	Height%
13,000	5363032	128326	49,816	53,543
16,173	5402712	111342	50,184	46,457

tR [min]	Area [µV·sec]	Height [µV]	Area%	Height%
13,067	3169131	90097	3,562	4,972
16,053	85807251	1721997	96,438	95,028

Ethyl (6a*R*,7*S*,10a*S*)-4-methoxy-9-methyl-3',6-dioxo-10,10a-dihydrospiro[benzo[c]chromene-7,1'-cyclohexane]-6a(6H)-carboxylate, **3f**.

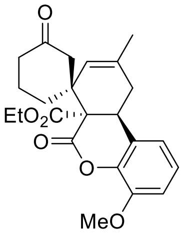

Following the general procedure **A**, starting from ethyl 8-methoxy-2-oxo-2*H*-1-benzopyran-3-carboxylate **2f** (50 mg, 0.2 mmol) and 3-(2-methylallyl)cyclohex-2-en-1-one **1** (63 µL, 0.4 mmol), QN-NH_2 **V** (13 mg, 0.02 mmol), 4-methyl benzoic acid (11 mg, 0.04 mmol) and toluene (200 µL, 1M), compound **3f** was obtained as a white solid (46 mg, 57%; reaction ran for 1 day). The diastereoselectivity of the reaction was 80:20. **Mp**: 150-155 °C. **IR** (neat) ν_{max}: 1780, 1699, 1485, 1228, 1136, 1099, 631, 607 cm^{-1}. **^{1}H NMR** (400 MHz, $CDCl_3$) δ 6.97 (dd, *J* = 8.3, 7.5 Hz, 1H), 6.78 – 6.71 (m, 2H), 5.37 (h, *J* = 1.7 Hz, 1H), 4.04 (dq, *J* = 10.7, 7.1 Hz, 1H), 3.94 (dq, *J* = 10.7, 7.1 Hz, 1H), 3.82 – 3.79 (m, 1H), 3.78 (s, 3H), 3.72 (dt, *J* = 14.5, 2.4 Hz, 1H), 2.45 – 2.32 (m, 2H), 2.30 (dd, *J* = 14.5, 0.9 Hz, 1H), 2.25 – 2.13 (m, 1H), 1.95 – 1.70 (m, 5H), 1.56 (q, *J* = 1.0 Hz, 3H), 0.90 (t, *J* = 7.1 Hz, 3H). **^{13}C NMR** (101 MHz, $CDCl_3$) δ 211.0, 167.8, 163.6, 147.5, 139.1, 130.6, 129.3, 125.2, 124.7, 119.1, 111.4, 62.0, 60.3, 56.1, 48.5, 44.1, 40.8, 36.6, 35.5, 34.7, 22.9, 20.5, 13.7. **HRMS** (ESI) m/z: [M^+] Calcd for $C_{23}H_{26}O_6$ 398.1729; found 398.1713. The enantiomeric excess was determined by HPLC using a Chiralpak IB column [*n*-hexanes/2-propanol (80:20)]; flow rate 1 mL/min, λ = 211 nm; τ_{major} = 11.7 min, τ_{minor} = 16.3 min (>99% ee); **$[\alpha]_D^{25}$** = -255.3 (c 0.9, $CHCl_3$).

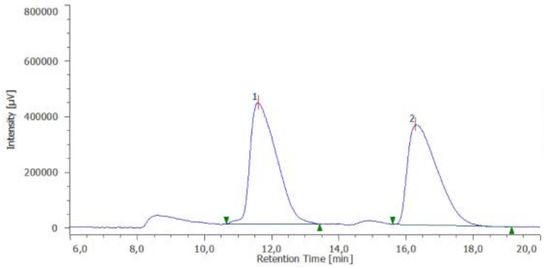

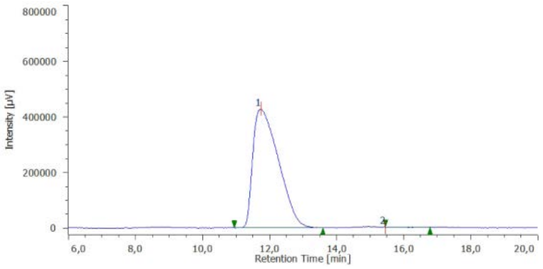

tR [min]	Area [µV·sec]	Height [µV]	Area%	Height%
11,600	23789653	436799	51,660	54,817
16,280	22261220	360033	48,340	45,183

tR [min]	Area [µV·sec]	Height [µV]	Area%	Height%
11,747	22633196	428342	99,975	99,773
15,467	5622	976	0,025	0,227

Ethyl (6a*R*,7*S*,10a*S*)-3-methoxy-9-methyl-3',6-dioxo-10,10a-dihydrospiro[benzo[c]chromene-7,1'-cyclohexane]-6a(6H)-carboxylate, **3g**.

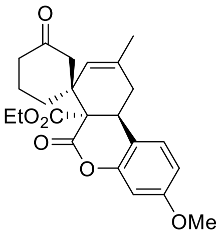

Following the general procedure **A**, starting from 3-carbethoxy-7-methoxy-coumarin **2g** (50 mg, 0.2 mmol) and 3-(2-methylallyl)cyclohex-2-en-1-one **1** (63 µL, 0.4 mmol), QN-NH_2 **V** (13 mg, 0.02 mmol), 4-methyl benzoic acid (11 mg, 0.04 mmol) and toluene (200 µL, 1M), compound **3g** was obtained as a white solid (41 mg, 51%; reaction ran for 1 day). The diastereoselectivity of the reaction was 88:12. **Mp**: 148-153 °C. **IR** (neat) ν_{max}: 2920, 1772, 1714, 1695, 1495, 1259, 1205, 1128, 1030, 856, 814 cm^{-1}. **^{1}H NMR** (400 MHz, $CDCl_3$) δ 6.94 – 6.90 (m, 1H), 6.78 – 6.72 (m, 2H), 5.44 (q, *J* = 1.7 Hz, 1H), 4.05 (dddd, *J* = 17.9, 10.7, 7.1, 3.7 Hz, 2H), 3.87 – 3.80 (m, 1H), 3.79 (s, 3H), 3.78 – 3.76 (m, 1H), 2.52 – 2.40 (m, 2H), 2.36 (dd, *J* = 14.5, 0.9 Hz, 1H), 2.31 – 2.19 (m, 1H), 2.02 – 1.79 (m, 5H), 1.64 (q, *J* = 1.0 Hz, 3H), 1.01 (t, *J* = 7.1 Hz, 3H).**^{13}C NMR** (101 MHz, $CDCl_3$) δ 211.2, 168.0, 164.6, 156.4, 143.9, 130.8, 129.2, 125.4, 117.6, 113.9, 112.6, 62.2, 60.5, 55.8, 48.7, 44.3, 41.0, 36.8, 35.7, 34.9, 23.0, 20.7, 14.0. **HRMS** (ESI) m/z: [M^+] Calcd for $C_{23}H_{26}O_6$ 398.1729; found 398.1720. The enantiomeric excess was determined by HPLC using a Chiralpak IB column [*n*-hexanes/2-propanol (80:20)]; flow rate 1 mL/min, λ = 213 nm; τ_{major} = 11.4 min, τ_{minor} = 18.5 min (99% ee); **$[\alpha]_D^{25}$** = -211.6 (c 0.5, $CHCl_3$).

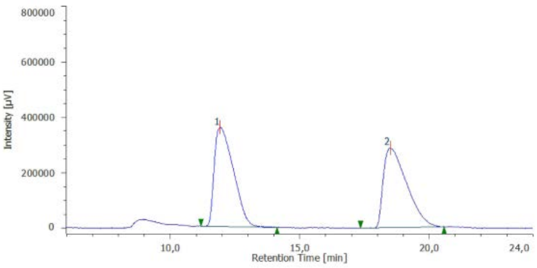

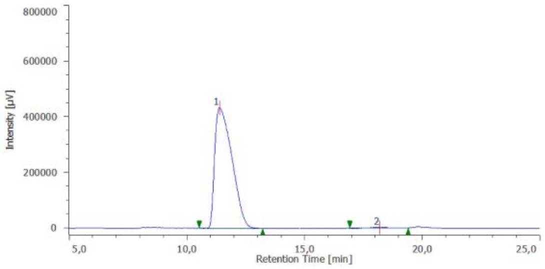

tR [min]	Area [µV·sec]	Height [µV]	Area%	Height%
11,920	18375813	357610	50,459	55,373
18,493	18041662	288214	49,541	44,627

tR [min]	Area [µV·sec]	Height [µV]	Area%	Height%
11,387	22023151	434005	99,522	99,462
18,187	105800	2348	0,478	0,538

Ethyl (6a*R*,7*S*,10a*S*)-3-chloro-9-methyl-3',6-dioxo-10,10a-dihydrospiro[benzo[c]chromene-7,1'-cyclohexane]-6a(6H)-carboxylate, **3h**.

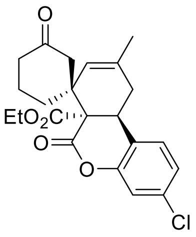

Following the general procedure **A**, starting from ethyl 7-chloro-2-oxo-2*H*-1-benzopyran-3-carboxylate **2h** (51 mg, 0.2 mmol) and 3-(2-methylallyl)cyclohex-2-en-1-one **1** (63 µL, 0.4 mmol), QN-NH_2 **V** (13 mg, 0.02 mmol), 4-methyl benzoic acid (11 mg, 0.04 mmol) and toluene (200 µL, 1M), of compound **3h** was obtained as white solid (38 mg, 41%, reaction ran for 1 day). The diastereoselectivity of the reaction was 51:49. **Mp**: 137-142 °C. **IR** (neat) ν_{max}: 1786, 1732, 1701, 1225, 1117, 1045, 850, 816 cm^{-1}. **^{1}H NMR** (400 MHz, $CDCl_3$) δ 7.16 (d, *J* = 8.1 Hz, 1H), 7.07 (dd, *J* = 8.1, 2.0 Hz, 1H), 7.02 (d, *J* = 2.0 Hz, 1H), 5.43 (q, *J* = 1.7 Hz, 1H), 4.07 (qd, *J* = 7.1, 3.1 Hz, 2H), 3.86 (dd, *J* = 11.1, 7.4 Hz, 1H), 3.74 (ddd, *J* = 14.4, 3.4, 1.4 Hz, 1H), 2.52 – 2.40 (m, 2H), 2.36 (dd, *J* = 14.4, 0.9 Hz, 1H), 2.32 – 2.18 (m, 1H), 2.00 – 1.78 (m, 5H), 1.63 (d, *J* = 1.3 Hz, 3H), 1.04 (td, *J* = 7.1, 0.6 Hz, 3H). **^{13}C NMR** (101 MHz, $CDCl_3$) δ 210.9, 167.6, 163.6, 150.5, 134.0, 130.7, 128.6, 126.8, 125.3, 124.9, 117.1, 62.44, 60.4, 48.6, 44.3, 40.9, 36.1, 35.7, 34.9, 23.0, 20.60, 14.0. **HRMS** (ESI) m/z: [M^+] Calcd for $C_{22}H_{23}ClO_5$ 402.1234; found 402.1218. The enantiomeric excess was determined by HPLC using a Chiralpak IA column [*n*-hexanes/2-propanol (90:10)]; flow rate 1 mL/min, λ = 208 nm; τ_{major} = 40.8 min, τ_{minor} = 64.5 min (>99% ee). **$[\alpha]_D^{25}$** = -310.6 (c 0.8, $CHCl_3$).

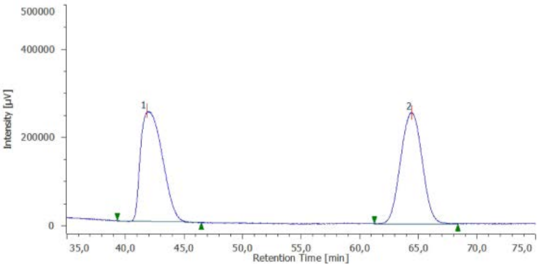

tR [min]	Area [µV·sec]	Height [µV]	Area%	Height%
41,853	31772492	249575	49,697	49,710
64,453	32159685	252482	50,303	50,290

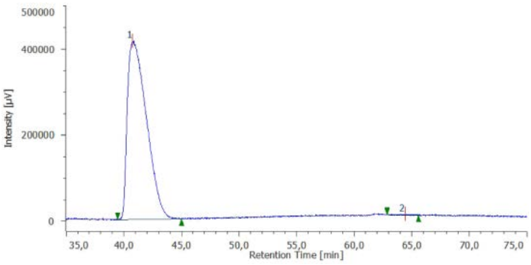

tR [min]	Area [µV·sec]	Height [µV]	Area%	Height%
40,773	47742668	415509	99,915	99,635
64,440	40528	1522	0,085	0,365

Ethyl (6a*R*,7*S*,10a*R*)-1-fluoro-9-methyl-3',6-dioxo-10,10a-dihydrospiro[benzo[c]chromene-7,1'-cyclohexane]-6a(6H)-carboxylate, **3i**.

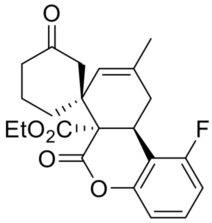

Following the general procedure **A**, starting from ethyl 5-fluoro-2-oxo-2H-chromene-3-carboxylate **2i** (48 mg, 0.2 mmol) and 3-(2-methylallyl)cyclohex-2-en-1-one **1** (63 µL, 0.4 mmol), QN-NH_2 **V** (13 mg, 0.02 mmol), 4-methyl benzoic acid (11 mg, 0.04 mmol) and toluene (200 µL, 1M), compound **3i** was obtained as a white solid (25 mg, 32%; reaction ran for 2 days). The diastereoselectivity of the reaction was 67:33. **Mp**: 130-135 °C. **IR** (neat) ν_{max}: 3062, 1784, 1714, 1626, 1595, 1468, 1257, 1130, 1022, 791 cm^{-1}. **^{1}H NMR** (400 MHz, $CDCl_3$) δ 7.19 (td, *J* = 8.3, 6.2 Hz, 1H), 6.89 – 6.77 (m, 2H), 5.43 (h, *J* = 1.6 Hz, 1H), 4.24 (dd, *J* = 11.0, 7.4 Hz, 1H), 4.06 (qd, *J* = 7.1, 0.8 Hz, 2H), 3.78 – 3.70 (m, 1H), 2.56 (dddd, *J* = 18.9, 7.4, 1.9, 0.9 Hz, 1H), 2.46 (m, 1H) 2.42 (ddd, *J* = 14.5, 12.5, 1.7 Hz, 1H), 2.33 – 2.20 (m, 1H), 2.07–1.72 (m, 5H), 1.64 (t, *J* = 1.2 Hz, 3H), 0.99 (t, *J* = 7.1 Hz, 3H). **^{13}C NMR** (101 MHz, $CDCl_3$). δ 210.9, 167.5, 163.4, 159.3 (d, *J* = 247.5 Hz), 151.1 (d, *J* = 6.7 Hz), 130.5, 129.0 (d, *J* = 9.3 Hz), 125.0, 116.6 (d, *J* = 22.1 Hz), 112.3 (d, *J* = 3.5 Hz), 111.3 (d, *J* = 21.3 Hz), 62.2, 60.0, 48.5, 44.1, 40.8, 35.6, 33.5, 30.1 (d, *J* = 2.0 Hz), 22.9, 20.5, 13.8. **^{19}F NMR** (377 MHz, $CDCl_3$) δ -120.24. **HRMS** (ESI) m/z: [M^+] Calcd for $C_{22}H_{23}FO_5$ 386.1530; found 386.1518. The enantiomeric excess was determined by HPLC using a Chiralpak IG column [*n*-hexanes/IPA (80:20)]; flow rate 1 mL/min, λ = 210 nm; τ_{minor} = 18.5 min, τ_{major} = 20.6 min (86% ee); **$[\alpha]_D^{25}$** = -171.8 (c 1.0, $CHCl_3$).

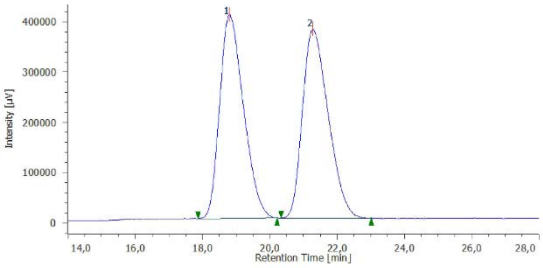

tR [min]	Area [µV·sec]	Height [µV]	Area%	Height%
18,800	20019624	405168	50,168	51,865
21,280	19885654	376032	49,832	48,135

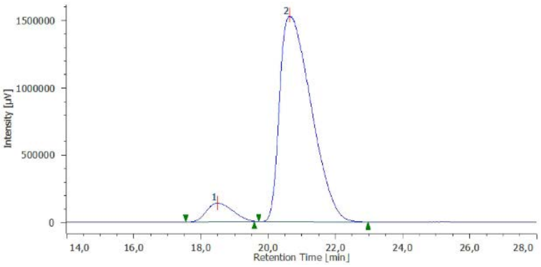

tR [min]	Area [µV·sec]	Height [µV]	Area%	Height%
18,493	7395859	136982	6,928	8,198
20,640	99358960	1534002	93,072	91,802

Ethyl (6a*R*,7*S*,10a*S*)-4-fluoro-9-methyl-3',6-dioxo-10,10a-dihydrospiro[benzo[c]chromene-7,1'-cyclohexane]-6a(6H)-carboxylate, **3j**.

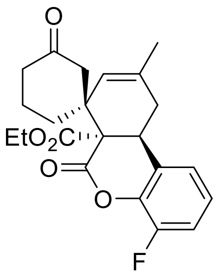

Following the general procedure **A**, starting from ethyl 8-fluoro-2-oxo-2H-chromene-3-carboxylate **2j** (48 mg, 0.2 mmol) and 3-(2-methylallyl)cyclohex-2-en-1-one **1** (63 µL, 0.4 mmol), QN-NH_2 **V** (13 mg, 0.02 mmol), 4-methyl benzoic acid (11 mg, 0.04 mmol) and toluene (200 µL, 1M), compound **3j** was obtained as a white solid (43 mg, 56%; reaction ran for 2 days). The diastereoselectivity of the reaction was 80:20. **Mp**: 176-182 °C. **IR** (neat) ν_{max}: 2968, 1786, 1731.6, 1709, 1410, 1221, 1196, 1120, 1082, 1026, 791 cm^{-1}. **^{1}H NMR** (400 MHz, $CDCl_3$) δ 7.02 (dtd, *J* = 11.5, 5.9, 2.8 Hz, 3H), 5.44 (h, *J* = 1.6 Hz, 1H), 4.12 (dq, *J* = 10.8, 7.1 Hz, 1H), 4.02 (dq, *J* = 10.8, 7.1 Hz, 1H), 3.92 (ddd, *J* = 11.2, 7.4, 1.0 Hz, 1H), 3.74 (dt, *J* = 14.5, 2.4 Hz, 1H), 2.49 (dddd, *J* = 19.3, 7.7, 2.1, 1.0 Hz, 1H), 2.45 – 2.39 (m, 1H), 2.36 (dd, *J* = 14.5, 0.9 Hz, 1H), 2.31 – 2.20 (m, 1H), 2.00 – 1.92 (m, 2H), 1.92 – 1.85 (m, 1H), 1.85 – 1.79 (m, 2H), 1.63 (q, *J* = 1.0 Hz, 3H), 1.00 (t, *J* = 7.1 Hz, 3H). **^{13}C NMR** (101 MHz, $CDCl_3$). δ 210.9, 167.6, 163.0, 145.0 (d, *J* = 251.7 Hz), 138.1 (d, *J* = 11.5 Hz), 130.70, 130.6, 125.3, 124.9 (d, *J* = 6.9 Hz), 122.7 (d, *J* = 3.7 Hz), 115.7 (d, *J* = 17.5 Hz), 62.4, 60.5, 48.6, 44.3, 40.9, 36.7 (d, *J* = 2.2 Hz), 35.6, 34.7, 23.0, 20.6, 13.8. **^{19}F NMR** (377 MHz, $CDCl_3$) δ -132.73. **HRMS** (ESI) m/z: [M^+] Calcd for $C_{22}H_{23}FO_5$ 386.1530; found 386.1518. The enantiomeric excess was determined by HPLC using a Chiralpak IG column [*n*-hexanes/IPA (60:40)]; flow rate 1 mL/min, λ = 212 nm; τ_{minor} = 10.7min, τ_{major} = 13.3 min (99% ee); **[α]$_D^{25}$** = -230.2 (c 1.0, $CHCl_3$).

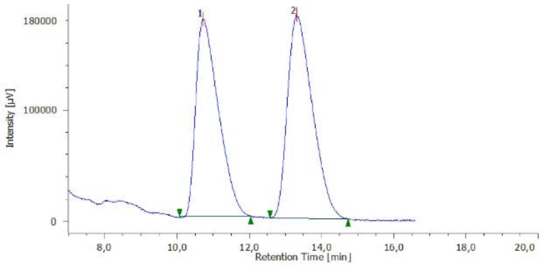

tR [min]	Area [µV·sec]	Height [µV]	Area%	Height%
10,720	7738855	177065	47,001	49,214
13,307	8726304	182724	52,999	50,786

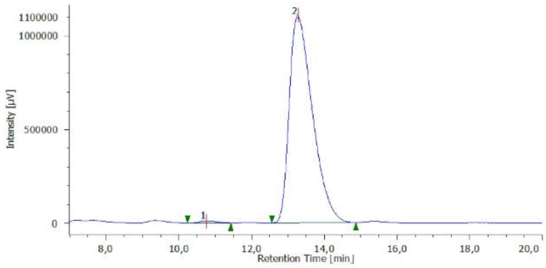

tR [min]	Area [µV·sec]	Height [µV]	Area%	Height%
10,760	337156	10313	0,688	0,923
13,280	48689915	1107539	99,312	99,077

Ethyl (6a*R*,7*S*,10a*S*)-4-bromo-9-methyl-3',6-dioxo-10,10a-dihydrospiro[benzo[c]chromene-7,1'-cyclohexane]-6a(6H)-carboxylate, **3k**.

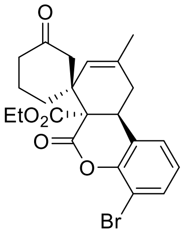

Following the general procedure **A**, starting from ethyl 8-bromo-2-oxo-2*H*-1-benzopyran-3-carboxylate **2k** (60 mg, 0.2 mmol) and 3-(2-methylallyl)cyclohex-2-en-1-one **1** (63 µL, 0.4 mmol), QN-NH_2 **V** (13 mg, 0.02 mmol), 4-methyl benzoic acid (11 mg, 0.04 mmol) and toluene (200 µL, 1M), compound **3k** was obtained as a white solid (55 mg, 61%; reaction ran for 3 days). The diastereoselectivity of the reaction was 76:24. **Mp**: 197-202 °C. **IR** (neat) ν_{max}: 1780, 1738, 1703, 1452, 1221, 1120, 1045, 1032, 775, 673 cm^{-1}. **^{1}H NMR** (400 MHz, $CDCl_3$) δ 7.45 (dd, *J* = 8.0, 1.5 Hz, 1H), 7.17 (dd, *J* = 7.6, 1.5 Hz, 1H), 6.97 (t, *J* = 7.8 Hz, 1H), 5.44 (q, *J* = 1.7 Hz, 1H), 4.15 (dq, *J* = 10.7, 7.1 Hz, 1H), 3.97 (dq, *J* = 10.7, 7.1 Hz, 1H), 3.88 (dd, *J* = 11.1, 7.5 Hz, 1H), 3.74 (dt, *J* = 14.4, 2.3 Hz, 1H), 2.52 – 2.39 (m, 2H), 2.37 – 2.31 (m, 1H), 2.26 (td, *J* = 15.0, 13.6, 6.8 Hz, 1H), 1.99 – 1.78 (m, 5H), 1.63 (d, *J* = 1.5 Hz, 3H), 0.99 (t, *J* = 7.1 Hz, 3H). **^{13}C NMR** (101 MHz, $CDCl_3$) δ 210.8, 167.4, 163.0, 147.1, 132.4, 130.4, 129.8, 126.7, 125.4, 125.2, 110.3, 62.3, 60.4, 48.5, 44.1, 40.8, 36.9, 35.5, 34.5, 22.9, 20.5, 13.8. **HRMS** (ESI) m/z: [M^+] calcd for $C_{22}H_{23}BrO_5$ 446.0729; found 446.0690. The enantiomeric excess was determined by HPLC using a Chiralpak

IG column [*n*-hexanes/2-propanol (70:30)]; flow rate 1 mL/min, λ = 227 nm; τ_{minor} = 15.4 min, τ_{major} = 18.1 min (99% ee); **[α]$_D^{25}$** = -244.2 (c 1.0, $CHCl_3$).

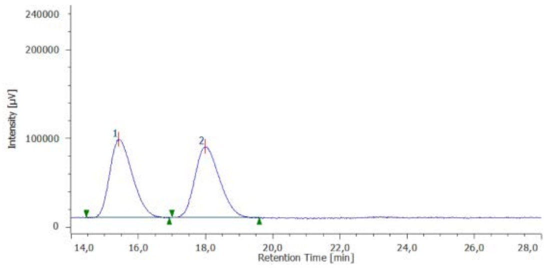

tR [min]	Area [µV·sec]	Height [µV]	Area%	Height%
15,413	4098125	87510	50,549	52,401
17,987	4009104	79490	49,451	47,599

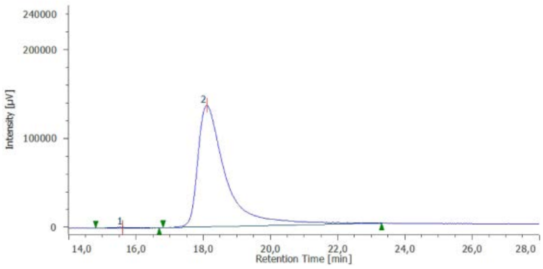

tR [min]	Area [µV·sec]	Height [µV]	Area%	Height%
15,613	40188	1031	0,504	0,748
18,107	7939537	136817	99,496	99,252

Ethyl (6a*R*,7*S*,10a*S*)-3-bromo-9-methyl-3',6-dioxo-10,10a-dihydrospiro[benzo[c]chromene-7,1'-cyclohexane]-6a(6H)-carboxylate, **3l**.

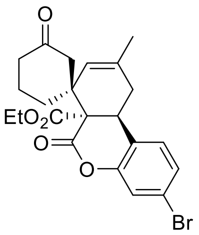

Following the general procedure **A**, starting from Ethyl 6-bromo-3-coumarincarboxylate **2i** (60 mg, 0.2 mmol) and 3-(2-methylallyl)cyclohex-2-en-1-one **1** (63 µL, 0.4 mmol), QN-NH_2 **V** (13 mg, 0.02 mmol), 4-methyl benzoic acid (11 mg, 0.04 mmol) and toluene (200 µL, 1M), compound **3l** was obtained as a white solid (48 mg, 60%; reaction ran for 1 day). The diastereoselectivity of the reaction was 78:22. **Mp**: 193-198 °C. **IR** (neat) ν_{max}: 1784, 1699, 1477, 1227, 1134, 814, 673 cm^{-1}. **^{1}H NMR** (400 MHz, $CDCl_3$) δ 7.39 – 7.32 (m, 2H), 6.89 (d, *J* = 8.4 Hz, 1H), 5.44 (q, *J* = 1.6 Hz, 1H), 4.07 (qd, *J* = 7.1, 2.3 Hz, 2H), 3.84 (dd, *J* = 11.1, 7.5 Hz, 1H), 3.75 (dt, *J* = 14.5, 2.3 Hz, 1H), 2.53 – 2.40 (m, 2H), 2.37 (dd, *J* = 14.5, 0.9 Hz, 1H), 2.26 (td, *J* = 13.9, 13.1, 6.4 Hz, 1H), 2.01 – 1.78 (m, 5H), 1.64 (d, *J* = 1.4 Hz, 3H), 1.02 (t, *J* = 7.1 Hz, 3H). **^{13}C NMR** (101 MHz, $CDCl_3$) δ 210.9, 167.6, 163.6, 149.2, 131.7, 130.6, 130.6, 130.3, 125.4, 118.5, 117.3, 62.4, 60.3, 48.6, 44.3, 40.9, 36.4, 35.7, 34.8, 23.0, 20.6, 14.0. **HRMS** (ESI) m/z: [M$^+$] Calcd for $C_{22}H_{23}BrO_5$ 446.0729; found 446.0696. The enantiomeric excess was determined by HPLC using a Chiralpak IG column [*n*-hexanes/2-propanol (70:30)]; flow rate 1 mL/min, λ = 211 nm; τ_{minor} = 17.2 min, τ_{major} = 19.3 min (95% ee); **[α]$_D^{25}$** = -161.6 (c 0.6, $CHCl_3$).

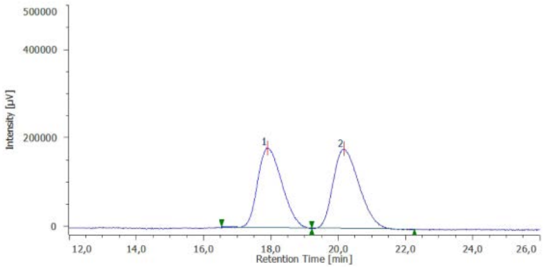

tR [min]	Area [µV·sec]	Height [µV]	Area%	Height%
17,893	8940130	179949	48,310	50,154
20,173	9565698	178847	51,690	49,846

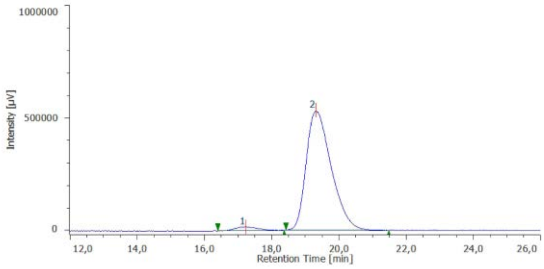

tR [min]	Area [µV·sec]	Height [µV]	Area%	Height%
17,227	771254	17218	2,747	3,133
19,307	27309232	532336	97,253	96,867

Ethyl (6a*R*,7*S*,10a*S*)-2,4-dibromo-9-methyl-3',6-dioxo-10,10a-dihydrospiro[benzo[c]chromene-7,1'-cyclohexane]-6a(6H)-carboxylate, **3m**.

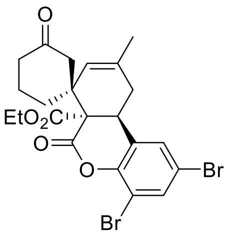

Following the general procedure **A**, starting from 6,8-Dibromo-3-ethoxycarbonyl coumarin **2m** (75 mg, 0.2 mmol) and 3-(2-methylallyl)cyclohex-2-en-1-one **1** (63 µL, 0.4 mmol), QN-NH_2 **V** (13 mg, 0.02 mmol), 4-methyl benzoic acid (11 mg, 0.04 mmol) and toluene (200 µL, 1M), compound **3m** was obtained as a white solid (63 mg, 60%; reaction ran for 5 days). The diastereoselectivity of the reaction was 73: 27. **Mp**: 193-197 °C. **IR** (neat) ν_{max}: 1788, 1699, 1446, 1238, 1047, 976, 775, 681 cm^{-1}. **^{1}H NMR** (300 MHz, $CDCl_3$) δ 7.61 (d, *J* = 2.2 Hz, 1H), 7.32 (d, *J* = 2.2 Hz, 1H), 5.44 (q, *J* = 1.7 Hz, 1H), 4.19 (dq, *J* = 10.7, 7.1 Hz, 1H), 4.02 (dq, *J* = 10.7, 7.1 Hz, 1H), 3.86 (dd, *J* = 11.2, 7.4 Hz, 1H), 3.71 (dt, *J* = 14.5, 2.3 Hz, 1H), 2.56 – 2.39 (m, 1H), 2.34 (dd, *J* = 14.5, 0.8 Hz, 1H), 2.30 – 2.18 (m, 1H), 2.00 – 1.78 (m, 5H), 1.63 (d, *J* = 1.3 Hz, 3H), 1.04 (t, *J* = 7.1 Hz, 3H). **^{13}C NMR** (101 MHz, $CDCl_3$) δ 210.7, 167.3, 162.5, 146.5, 134.7, 131.3, 130.4, 129.7, 125.3, 117.3, 111.3, 62.6, 60.2, 48.5, 44.2, 40.9, 37.0, 35.6, 34.4, 22.9, 20.6, 14.0. **HRMS** (ESI) m/z: [M^+] Calcd for $C_{22}H_{22}Br_2O_5$ 523.9834, found 523.9799. The enantiomeric excess was determined by HPLC using a Chiralpak IA column [*n*-hexanes/2-propanol (95:5)]; flow rate 1 mL/min, λ = 212 nm; τ_{minor} = 18.0 min, τ_{major} = 24.2 min (>99% ee); **$[\alpha]_D^{25}$** = -199.5 (c 1.3, $CHCl_3$).

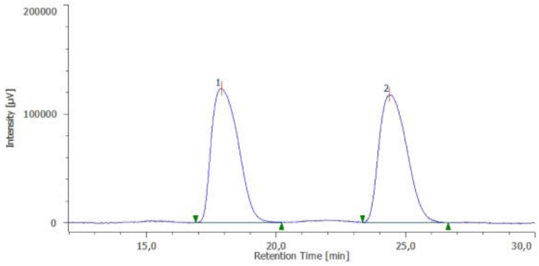

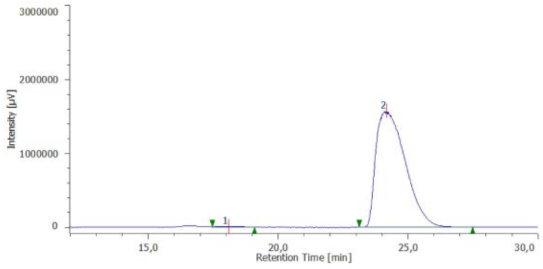

tR [min]	Area [µV·sec]	Height [µV]	Area%	Height%
17.893	8846031	123364	50.348	51.186
24.373	8723583	117649	49.652	48.814

tR [min]	Area [µV·sec]	Height [µV]	Area%	Height%
18.093	530119	9632	0.428	0.609
24.173	123290267	1572519	99.572	99.391

Ethyl (6a*R*,7*S*,10a*S*)-9-methyl-2-nitro-3',6-dioxo-10,10a-dihydrospiro[benzo[c]chromene-7,1'-cyclohexane]-6a(6H)-carboxylate, **3n**.

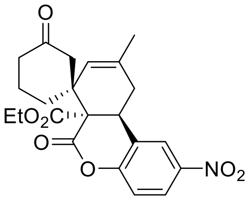

Following the general procedure **A**, starting from ethyl 6-nitro-2-oxo-2H-chromene-3-carboxylate **2n** (52 mg, 0.2 mmol) and 3-(2-methylallyl)cyclohex-2-en-1-one **1** (63 µL, 0.4 mmol), QN-NH_2 **V** (13 mg, 0.02 mmol), 4-methyl benzoic acid (11 mg, 0.04 mmol) and toluene (200 µL, 1M), compound **3n** was obtained as a pale yellow solid (47 mg, 57%; reaction ran for 2 days). The diastereoselectivity of the reaction was 78:22. **Mp**: 173-178 °C. **IR** (neat) ν_{max}: 2960, 1793, 1732, 1701, 1523, 1344, 1230, 1113, 1028, 964, 825 cm^{-1}. **^{1}H NMR** (400 MHz, $CDCl_3$) δ 8.22 – 8.07 (m, 2H), 7.13 (d, *J* = 8.7 Hz, 1H), 5.45 (h, *J* = 1.6 Hz, 1H), 4.08 (qd, *J* = 7.1, 1.4 Hz, 2H), 4.02 (dd, *J* = 11.2, 7.5 Hz, 1H), 3.69 (dt, *J* = 14.4, 2.3 Hz, 1H), 2.55 (dddd, *J* = 18.9, 7.5, 1.9, 0.9 Hz, 1H), 2.49 – 2.43 (m, 1H), 2.40 (dd, *J* = 14.5, 0.8 Hz, 1H), 2.27 (tdd, *J* = 13.4, 6.4, 1.7 Hz, 1H), 2.02 – 1.95 (m, 2H), 1.94 – 1.89 (m, 1H), 1.89 – 1.83 (m, 1H), 1.83 – 1.77 (m, 1H), 1.65 (q, *J* = 1.0 Hz, 3H), 1.02 (t, *J* = 7.1 Hz, 3H). **^{13}C NMR** (101 MHz, $CDCl_3$). δ 210.5, 167.2, 162.5, 154.5, 144.3, 130.4, 129.4, 125.3, 124.7, 123.7, 117.6, 62.7, 60.2, 48.5, 44.2, 40.9, 36.5, 35.6, 34.6, 22.9, 20.5, 14.1. **HRMS** (ESI) m/z: [M^+] Calcd for $C_{22}H_{23}NO_7$ 413.1475; found 413.1464. The enantiomeric excess was determined by HPLC using a Chiralpak

IG column [*n*-hexanes/IPA (90:10)]; flow rate 1 mL/min, λ = 212 nm; τ_{minor} = 108.4 min, τ_{major} = 118.0 min (94% ee); **[α]$_D^{25}$** = -173.8 (c 1.0, $CHCl_3$).

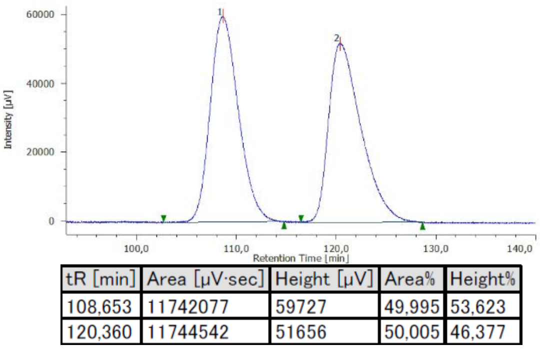

tR [min]	Area [µV·sec]	Height [µV]	Area%	Height%
108,653	11742077	59727	49,995	53,623
120,360	11744542	51656	50,005	46,377

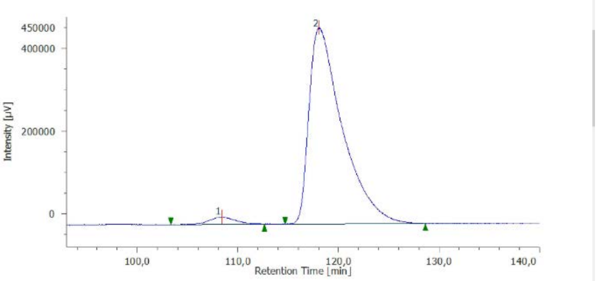

tR [min]	Area [µV·sec]	Height [µV]	Area%	Height%
108,387	3293267	17851	2,927	3,616
118,027	109208402	475873	97,073	96,384

Ethyl (1*S*,4a'*R*,12c'*S*)-2'-methyl-3,5'-dioxo-1',12c'-dihydrospiro[cyclohexane-1,4'-dibenzo[c,f]chromene]-4a'(5'H)-carboxylate, **3o**.

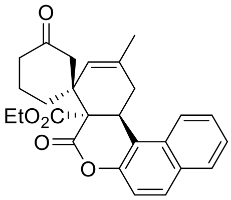

Following the general procedure **A**, starting from ethyl 3-oxo-3H-benzo[f]chromene-2-carboxylate **2o** (54 mg, 0.2 mmol) and 3-(2-methylallyl)cyclohex-2-en-1-one **1** (63 µL, 0.4 mmol), QN-NH_2 **V** (13 mg, 0.02 mmol), 4-methyl benzoic acid (11 mg, 0.04 mmol) and toluene (200 µL, 1M), compound **3o** was obtained as a white solid (63 mg, 75%; reaction ran for 2 days). The diastereoselectivity of the reaction was 78:22. **Mp**: 230-235 °C. **IR** (neat) ν_{max}: 2964, 1765, 1703, 1516, 1468, 1248, 1236, 1217, 1147, 823, 758 cm^{-1}. **^{1}H NMR** (400 MHz, $CDCl_3$) δ 8.06 – 7.97 (m, 1H), 7.87 – 7.82 (m, 1H), 7.74 (d, *J* = 8.9 Hz, 1H), 7.60 (ddd, *J* = 8.4, 6.9, 1.3 Hz, 1H), 7.47 (ddd, *J* = 8.1, 6.9, 1.1 Hz, 1H), 7.18 (d, *J* = 8.9 Hz, 1H), 5.51 (p, *J* = 1.6 Hz, 1H), 4.58 (dd, *J* = 11.2, 7.4 Hz, 1H), 3.94 (q, *J* = 7.1 Hz, 2H), 3.88 – 3.80 (m, 1H), 2.68 (dddd, *J* = 19.0, 7.4, 1.9, 0.9 Hz, 1H), 2.45 (ddd, *J* = 14.5, 11.2, 1.5 Hz, 2H), 2.29 (ddd, *J* = 14.7, 12.4, 6.9 Hz, 1H), 2.17 (dd, *J* = 12.0, 3.1 Hz, 1H), 2.06 – 1.93 (m, 2H), 1.92 – 1.80 (m, 2H), 1.66 (d, *J* = 1.3 Hz, 3H), 0.79 (t, *J* = 7.1 Hz, 3H). **^{13}C NMR** (101MHz, $CDCl_3$) δ 211.2, 167.9, 164.4, 147.7, 130.9, 130.7, 129.3, 129.0, 127.6, 125.4, 125.3, 122.4, 122.1, 116.9, 62.1, 60.2, 48.7, 44.3, 41.0, 35.9, 34.0, 32.4, 23.1, 20.7, 13.8. **HRMS** (ESI) m/z: [M^+] Calcd for $C_{26}H_{26}O_5$ 418.178, found 418.176. The enantiomeric excess was determined by HPLC using a Chiralpak IG column [*n*-hexanes/IPA (60:40)]; flow rate 1 mL/min, λ = 236 nm; τ_{minor} = 13.9 min, τ_{major} = 17.0 min (96% ee); **[α]$_D^{25}$** = -174.1 (c 1.0, $CHCl_3$).

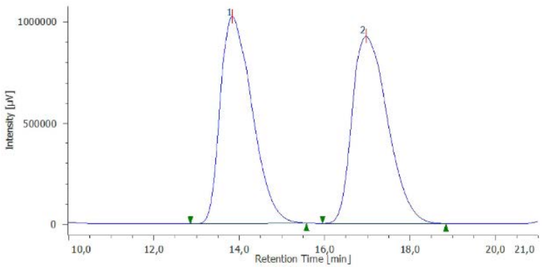

tR [min]	Area [µV·sec]	Height [µV]	Area%	Height%
13,840	53128775	1023764	49,742	52,511
16,973	53680776	925864	50,258	47,489

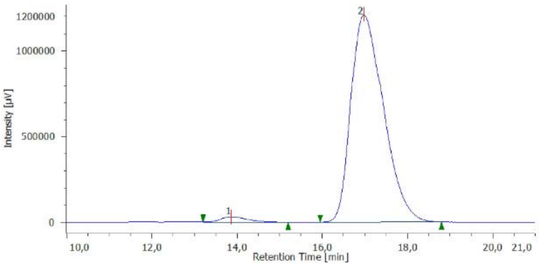

tR [min]	Area [µV·sec]	Height [µV]	Area%	Height%
13,867	1341943	30547	2,041	2,466
16,973	64398860	1208017	97,959	97,534

Isopropyl (6a*R*,7*S*,10a*S*)-9-methyl-3',6-dioxo-10,10a-dihydrospiro[benzo[c]chromene-7,1'-cyclohexane]-6a(6H)-carboxylate, **3p**.

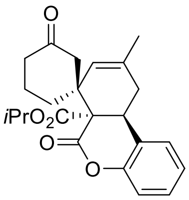

Following the general procedure **A**, starting from 3-isobutyrylcoumarin **2p** (43 mg, 0.2 mmol) and 3-(2-methylallyl)cyclohex-2-en-1-one **1** (63 µL, 0.4 mmol), QN-NH_2 **V** (13 mg, 0.02 mmol), 4-methyl benzoic acid (11 mg, 0.04 mmol) and toluene (200 µL, 1M), compound **3p** was obtained as a white solid (34mg, 45%; reaction ran for 2 days). The diastereoselectivity of the reaction was 79:21. **Mp**: 143-148 °C. **IR** (neat) ν_{max}: 1770, 1726, 1707, 1238, 1134, 1101, 767, 757 cm^{-1}. **^{1}H NMR** (300 MHz, $CDCl_3$) δ 7.28 – 7.17 (m, 2H), 7.08 (td, J = 7.4, 1.2 Hz, 1H), 7.02 – 6.96 (m, 1H), 5.44 (q, J = 1.7 Hz, 1H), 4.87 (hept, J = 6.2 Hz, 1H), 3.86 (dd, J = 11.1, 7.4 Hz, 1H), 3.78 (dt, J = 14.5, 2.3 Hz, 1H), 2.54 – 2.41 (m, 2H), 2.37 (d, J = 14.5 Hz, 1H), 2.26 (td, J = 14.2, 13.2, 6.5 Hz, 1H), 2.03 – 1.80 (m, 5H), 1.63 (s, 3H), 1.04 (d, J = 6.3 Hz, 3H), 0.93 (d, J = 6.2 Hz, 3H). **^{13}C NMR** (75 MHz, $CDCl_3$) δ 211.1, 167.3, 164.3, 150.1, 130.7, 128.6, 128.2, 127.6, 125.3, 124.5, 116.6, 70.2, 60.4, 48.5, 44.2, 40.9, 36.4, 35.6, 34.9, 22.9, 21.4, 21.2, 20.6. **HRMS** (ESI) m/z: [M^+] Calcd for $C_{23}H_{26}O_5$ 382,1780; found 382.1763. The enantiomeric excess was determined by HPLC using a Chiralpak IA column [*n*-hexanes/2-propanol (90:10)]; flow rate 1 mL/min, λ = 208 nm; τ_{minor} = 46.9 min, τ_{major} = 60.0 min (>99% ee); **$[\alpha]_D^{25}$** = -266.9 (c 0.9, $CHCl_3$).

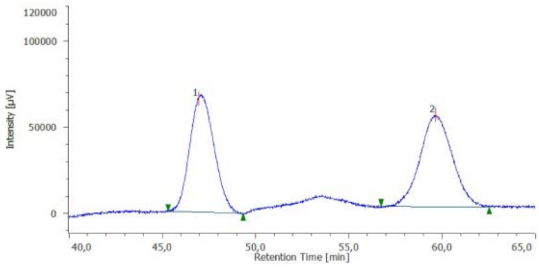

tR [min]	Area [µV·sec]	Height [µV]	Area%	Height%
46,947	6149800	65736	49,129	55,218
59,640	6367735	53313	50,871	44,782

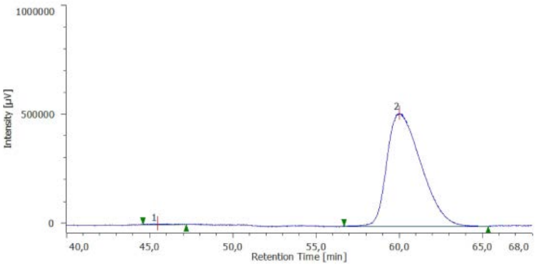

tR [min]	Area [µV·sec]	Height [µV]	Area%	Height%
45,467	173241	4188	0,233	0,795
60,013	74143233	522583	99,767	99,205

6a-benzoyl-9-methyl-10,10a-dihydrospiro[benzo[c]chromene-7,1'-cyclohexane]-3',6(6aH)-dione, **5**.

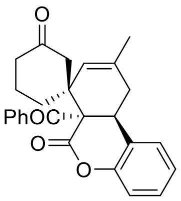

Following the general procedure **A**, starting from 3-benzoyl-chromen-2-one **4** (50 mg, 0.2 mmol) and 3-(2-methylallyl)cyclohex-2-en-1-one **1** (63 µL, 0.4 mmol), QN-NH_2 **V** (13 mg, 0.02 mmol), 4-methyl benzoic acid (11 mg, 0.04 mmol) and toluene (200 µL, 1M), compound **5** was obtained as a white solid mixture of two inseparable diastereoisomers (70 mg, 87%; reaction ran for 2 days). The diastereoselectivity of the reaction was 67:33. **Mp**: 139-144 °C. **IR** (neat) ν_{max}: 1728, 1662, 1612, 1219, 1057, 897, 752, 735 cm^{-1}. **^{1}H NMR** (300 MHz, $CDCl_3$) δ 7.47 – 7.36 (m, 7H), 7.32 (tdd, J = 7.1, 2.0, 0.9 Hz, 2H), 7.26 – 7.20 (m, 2H), 7.17 (td, J = 7.4, 1.3 Hz, 2H), 7.11 (dd, J = 8.0, 1.2 Hz, 2H), 6.03 (s, 1H), 5.98 (s, 1H), 3.92 (dt, J = 12.2, 6.3 Hz, 2H), 2.93 (d, J = 13.7 Hz, 1H), 2.75 (d, J = 13.5 Hz, 1H), 2.70 – 2.26 (m, 10H), 2.13 – 2.02 (m, 3H), 1.98 – 1.82 (m, 3H), 1.54 (s, 1H), 1.49 (s, 3H). **^{13}C NMR** (101 MHz, $CDCl_3$) δ 199.7, 199.3, 164.4, 164.2, 164.1, 164.0, 160.8, 153.6, 151.4, 151.4, 134.9, 134.9, 130.4, 130.3, 130.2, 129.3, 128.9, 128.6, 128.6, 128.5, 128.1, 125.2, 124.9, 124.7, 124.5, 117.0, 117.0, 79.2, 79.1, 50.3, 47.7, 44.9, 37.2, 37.2, 36.2, 34.1, 31.6, 31.3, 29.1, 28.9, 28.4, 26.8, 22.8, 19.0. **HRMS** (ESI) m/z: [M^+] Calcd for $C_{26}H_{24}O_4$ 400.1675; found 400.1655. The enantiomeric excess was determined by HPLC using a Chiralpak IA column [*n*-hexanes/2-propanol (90:10)]; flow rate 1 mL/min, λ = 220 nm; τ_1 = 46.7 min, τ_2 = 129.0 min (racemic). The other peak pair corresponds to the minor diastereoisomer.

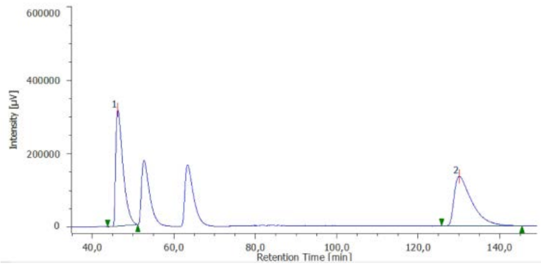

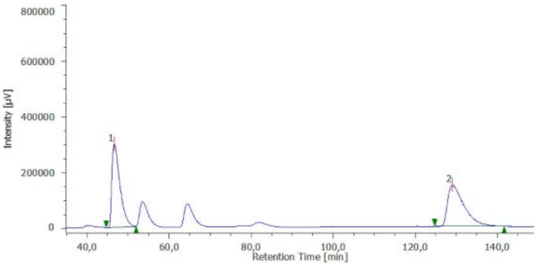

tR [min]	Area [μV·sec]	Height [μV]	Area%	Height%
46,267	39592213	316538	49,433	70,078
130,107	40500743	135155	50,567	29,922

tR [min]	Area [μV·sec]	Height [μV]	Area%	Height%
46,693	40621685	297081	49,647	66,803
129,040	41198869	147628	50,353	33,197

(6a*S*,7*R*,10a*S*)-9-methyl-6a-nitro-10,10a-dihydrospiro[benzo[c]chromene-7,1'-cyclohexane]-3',6(6aH)-dione, **7**.

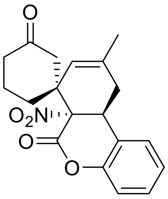

Following the general procedure **A**, starting from 3-nitrocoumarin **6** (38 mg, 0.2 mmol) and 3-(2-methylallyl)cyclohex-2-en-1-one **1** (63 µL, 0.4 mmol), QN-NH_2 **V** (13 mg, 0.02 mmol), 4-methyl benzoic acid (11 mg, 0.04 mmol) and toluene (200 µL, 1M), compound **7** was obtained as a white solid (18 mg, 26%; reaction ran for 5 days). The diastereoselectivity of the reaction was 29:71. It is worth mentioning that no reactivity was observed when procedure **B** was used. **Mp**: 155-160 °C. **IR** (neat) ν_{max}: 1778, 1716, 1546, 1228, 118, 1144, 763 cm^{-1}. **^{1}H NMR** (400 MHz, $CDCl_3$) δ 7.30 (ddd, J = 8.1, 7.4, 1.7 Hz, 1H), 7.22 (dd, J = 7.6, 1.7 Hz, 1H), 7.14 (td, J = 7.5, 1.2 Hz, 1H), 7.07 (dd, J = 8.2, 1.2 Hz, 1H), 5.58 (p, J = 1.7 Hz, 1H), 4.06 (dd, J = 10.7, 8.0 Hz, 1H), 3.16 (dd, J = 14.4, 4.1 Hz, 1H), 2.79 – 2.68 (m, 1H), 2.53 (d, J = 1.5 Hz, 2H), 2.42 (dt, J = 14.6, 3.0 Hz, 1H), 2.26 (ddd, J = 14.3, 12.8, 7.1 Hz, 1H), 2.14 – 1.97 (m, 3H), 1.75 – 1.68 (m, 1H), 1.67 – 1.63 (m, 3H).**^{13}C NMR** (101 MHz, $CDCl_3$) δ 208.4, 158.5, 149.5, 131.9, 129.8, 128.0, 125.6, 124.6, 123.7, 117.3, 97.1, 51.7, 46.0, 40.7, 37.3, 36.4, 29.1, 22.8, 20.8. **HRMS** (ESI) m/z: [M^+-H_2NO_2] Calcd for $C_{19}H_{19}O_5$ 293.1178, found 293.1170. The enantiomeric excess was determined by HPLC using a Chiralpak IB column [*n*-hexanes/2-propanol (90:10)]; flow rate 1 mL/min, λ = 210 nm; τ_{minor} = 17.7 min, τ_{major} = 21.1 min 67% ee); **$[\alpha]_D^{25}$** = +269.9 (c 0.4, $CHCl_3$).

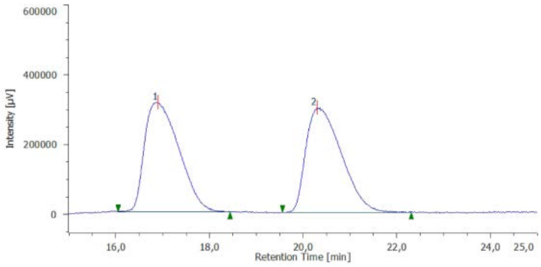

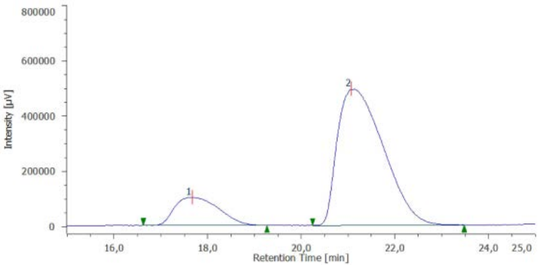

tR [min]	Area [μV·sec]	Height [μV]	Area%	Height%
16,907	16057939	314791	50,472	51,074
20,293	15757769	301555	49,528	48,926

tR [min]	Area [μV·sec]	Height [μV]	Area%	Height%
17,667	6706661	102414	16,609	17,151
21,067	33672662	494726	83,391	82,849

5.2 Spirocyclic characterization of 3a'-3e' and 3h'.

Ethyl (6a*S*,7*S*,10a*R*)-9-methyl-3',6-dioxo-10,10a-dihydrospiro[benzo[c]chromene-7,1'-cyclohexane]-6a(6H)-carboxylate, **3a'**.

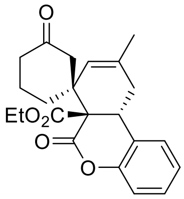

Following the general procedure **B**, starting from ethyl 3-coumarincarboxylate **2a** (44 mg, 0.2 mmol) and 3-(2-methylallyl)cyclohex-2-en-1-one **1** (63 µL, 0.8 mmol), QN-NH_2 **V** (13 mg, 0.02 mmol), Et_3N (11 mg, 0.04 mmol) and toluene (200 µL, 1M), compound **3a'** was obtained as a white solid (29 mg, 40%; reaction ran for 7 days). The diastereoselectivity ratio of the reaction was 39:61. **Mp**: 178-183 °C. **IR** (neat) ν_{max}: 1776, 1728, 1699, 1228, 1200, 1126, 1041, 1020, 762 cm^{-1}. **^{1}H NMR** (400 MHz, $CDCl_3$) δ 7.26 – 7.16 (m, 2H), 7.12 – 7.05 (m, 1H), 7.01 (dd, *J* = 8.1, 0.9 Hz, 1H), 5.59 (h, *J* = 1.7 Hz, 1H), 4.12 – 3.91 (m, 2H), 3.70 (dd, *J* = 10.8, 7.7 Hz, 1H), 3.14 (dd, *J* = 14.0, 3.5 Hz, 1H), 2.73 (d, *J* = 12.9 Hz, 1H), 2.50 (dd, *J* = 19.1, 7.6 Hz, 1H), 2.45 – 2.33 (m, 2H), 2.25 (dt, *J* = 14.0, 9.6 Hz, 1H), 2.06 (ddt, *J* = 10.0, 6.1, 3.3 Hz, 2H), 1.91 (dd, *J* = 19.1, 10.8 Hz, 1H), 1.76 – 1.66 (m, 1H), 1.62 (s, 3H), 0.94 (t, *J* = 7.1 Hz, 3H). **^{13}C NMR** (101 MHz, $CDCl_3$) δ 210.0, 168.4, 164.9, 150.2, 131.1, 128.8, 127.6 (2C), 124.6, 124.4, 116.6, 62.1, 60.9, 52.3, 44.9, 40.8, 36.0, 35.0, 30.6, 23.0, 21.0, 13.7. **HRMS** (ESI) m/z: [M^+] Calcd for $C_{22}H_{24}O_5$ 368.1624; found 368.1627. The enantiomeric excess was determined by HPLC using a Chiralpak IG column [*n*-hexanes/2-propanol (90:10)]; flow rate 1 mL/min, λ = 218 nm; τ_{minor} = 21.5 min, τ_{major} = 70.3 min >99% *ee*); **$[\alpha]_D^{25}$** = +250.4 (c 1.0, $CHCl_3$).

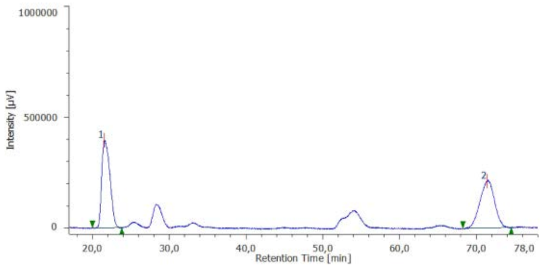

tR [min]	Area [µV·sec]	Height [µV]	Area%	Height%
21.547	29214450	393357	50.888	65.285
71.387	28195185	209166	49.112	34.715

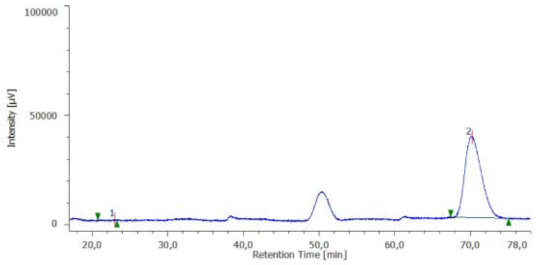

tR [min]	Area [µV·sec]	Height [µV]	Area%	Height%
23.040	12007	593	0.213	1.590
70.267	5615324	36692	99.787	98.410

Ethyl (6a*S*,7*S*,10a*R*)-4,9-dimethyl-3',6-dioxo-10,10a-dihydrospiro[benzo[c]chromene-7,1'-cyclohexane]-6a(6H)-carboxylate, **3b'**.

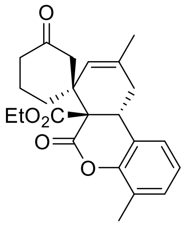

Following the general procedure **B**, starting from ethyl 8-methyl-2-oxo-2H-chromene-3-carboxylate **2b** (46 mg, 0.2 mmol) and 3-(2-methylallyl)cyclohex-2-en-1-one **1a** (63 µL, 0.8 mmol), QN-NH_2 **V** (13 mg, 0.02 mmol), Et_3N (11 mg, 0.04 mmol) and toluene (200 µL, 1M), compound **3b'** was obtained as a white solid (31mg, 41%; reaction ran for 7 days). The diastereoselectivity ratio of the reaction was 28:72. **Mp**: 179-184 °C. **IR** (neat) ν_{max}: 1770, 1734, 1701, 1471, 1437, 1225, 1200, 1126, 1039, 779 cm^{-1}. **^{1}H NMR** (400 MHz, $CDCl_3$) δ 7.01 (dd, *J* = 7.4, 1.9 Hz, 1H), 6.98 – 6.87 (m, 2H), 5.53 (s, 1H), 4.09 – 3.97 (m, 1H), 3.95 – 3.80 (m, 1H), 3.60 (dd, *J* = 10.8, 7.7 Hz, 1H), 3.08 (d, *J* = 15.1 Hz, 1H), 2.67 (d, *J* = 12.9 Hz, 1H), 2.47 – 2.29 (m, 3H), 2.21 (s, 3H), 2.04 – 1.94 (m, 2H), 1.90 – 1.79 (m, 1H), 1.67 – 1.58 (m, 2H), 1.56 (s, 3H), 0.85 (t, *J* = 7.1 Hz, 3H). **^{13}C NMR** (101 MHz, $CDCl_3$) δ 210.1, 168.5, 165.0, 148.4, 131.1, 130.1, 127.3, 126.0, 125.2, 124.3, 124.2, 61.9, 60.8, 52.3, 44.9, 40.8, 36.1, 34.9, 30.6, 23.0, 21.0, 15.5, 13.7. **HRMS** (ESI) m/z: [M^+] Calcd for $C_{23}H_{26}O_5$ 382.1780, found 382.1792. The enantiomeric excess was determined by HPLC using a Chiralpak IA column [*n*-hexanes/2-propanol (96:4)]; flow rate 1 mL/min, λ = 210 nm; τ_{major} = 13.3 min, τ_{minorr} = 53.3 min 92% *ee*); **$[\alpha]_D^{25}$** = +265.4 (c 1.0, $CHCl_3$).

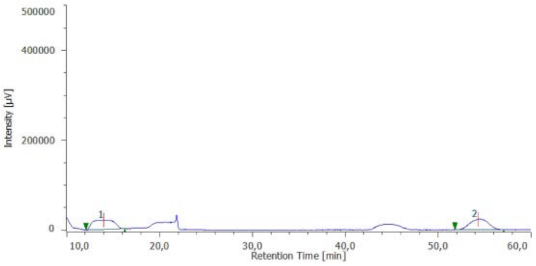

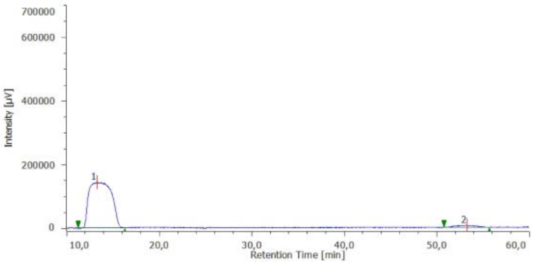

tR [min]	Area [µV·sec]	Height [µV]	Area%	Height%
14,013	3383489	20158	50,355	46,519
54,280	3335747	23175	49,645	53,481

tR [min]	Area [µV·sec]	Height [µV]	Area%	Height%
13,293	24772853	143928	95,990	96,131
53,267	1034791	5793	4,010	3,869

Ethyl (6a*S*,7*S*,10a*R*)-3,9-dimethyl-3',6-dioxo-10,10a-dihydrospiro[benzo[c]chromene-7,1'-cyclohexane]-6a(6H)-carboxylate, **3c'**.

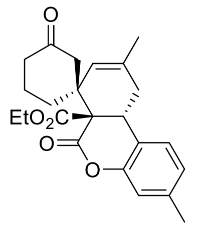

Following the general procedure **B**, starting from ethyl 7-methyl-2-oxo-2H-chromene-3-carboxylate **2c** (46 mg, 0.2 mmol) and 3-(2-methylallyl)cyclohex-2-en-1-one **1** (63 µL, 0.8 mmol), QN-NH_2 **V** (13 mg, 0.02 mmol), Et_3N (11 mg, 0.04 mmol) and toluene (200 µL, 1M), compound **3c'** was obtained as a white solid (25 mg, 32%; reaction ran for 7 days). The diastereoselectivity ratio of the reaction was 35:65. **Mp**: 95-100 °C. **IR** (neat) ν_{max}: 1774, 1728, 1709, 1624, 1448, 1294, 1232, 1132, 1038, 966, 831 cm^{-1}. **^{1}H NMR** (400 MHz, $CDCl_3$) δ 7.06 (d, *J* = 7.6 Hz, 1H), 6.88 (d, *J* = 7.6 Hz, 1H), 6.82 (s, 1H), 5.58 (s, 1H), 4.11 – 3.94 (m, 2H), 3.65 (dd, *J* = 10.7, 7.8 Hz, 1H), 3.13 (d, *J* = 14.0 Hz, 1H), 2.71 (d, *J* = 12.9 Hz, 1H), 2.52 – 2.34 (m, 3H), 2.31 (s, 3H), 2.29 – 2.18 (m, 1H), 2.05 (m, 2H), 1.87 (dd, *J* = 19.1, 10.8 Hz, 1H), 1.74 – 1.64 (m, 1H), 1.61 (s, 3H), 0.97 (t, *J* = 7.1 Hz, 3H). **^{13}C NMR** (101 MHz, $CDCl_3$) δ 210.0, 168.5, 165.1, 150.1, 139.0, 131.2, 127.3, 125.3, 124.6, 124.4, 117.0, 62.0, 61.0, 52.3, 44.9, 40.8, 35.7, 35.2, 30.6, 23.0, 21.2, 13.8. **HRMS** (ESI) m/z: [M^+] Calcd for $C_{23}H_{26}O_5$ 382.1780; found 382.1792. The enantiomeric excess was determined by HPLC using a Chiralpak IG column [*n*-hexanes/2-propanol (60:40)]; flow rate 1 mL/min, λ = 219 nm; τ_{major} = 7.9 min, τ_{minor} = 17.3 min, 83% *ee*); **$[\alpha]_D^{25}$** = +291.8 (c 1.0, $CHCl_3$).

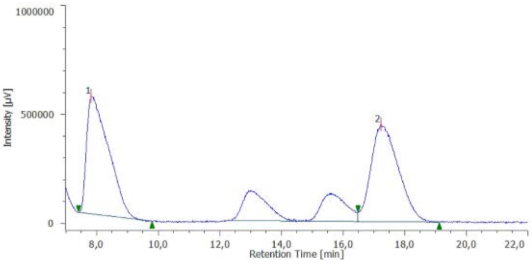

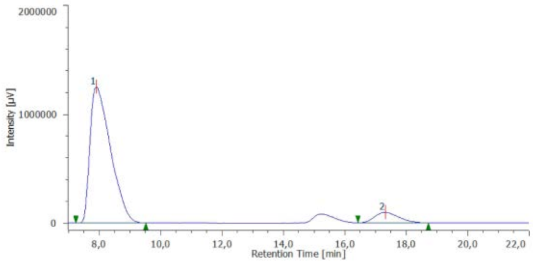

tR [min]	Area [µV·sec]	Height [µV]	Area%	Height%
7,827	26697989	540392	49,202	54,813
17,227	27564507	445494	50,798	45,187

tR [min]	Area [µV·sec]	Height [µV]	Area%	Height%
7,907	58013335	1251409	91,661	92,935
17,320	5277634	95129	8,339	7,065

Ethyl (6a*S*,7*S*,10a*R*)-2,9-dimethyl-3',6-dioxo-10,10a-dihydrospiro[benzo[c]chromene-7,1'-cyclohexane]-6a(6H)-carboxylate, **3d'**.

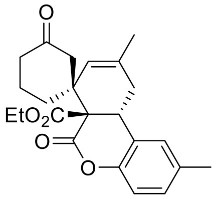

Following the general procedure **B**, starting from ethyl 6-methyl-2-oxo-2H-chromene-3-carboxylate **2d** (46 mg, 0.2 mmol) and 3-(2-methylallyl)cyclohex-2-en-1-one **1** (63 µL, 0.8 mmol), QN-NH_2 **V** (13 mg, 0.02 mmol), Et_3N (11 mg, 0.04 mmol) and toluene (200 µL, 1M), compound **3d'** was obtained as a white solid (19 mg, 22%; reaction ran for 7 days). The diastereoselectivity ratio of the reaction was 40:60. **Mp**: 160-165 °C. **IR** (neat) ν_{max}: 1776, 1701, 1495, 1288, 1254, 1196, 1140, 814 cm^{-1}. **^{1}H NMR** (400 MHz, $CDCl_3$) δ 7.05 – 7.00 (m, 1H), 6.98 (s, 1H), 6.89 (d, *J* = 8.2 Hz, 1H), 5.58 (s, 1H), 4.14 – 3.92 (m, 2H), 3.64 (dd, *J* = 10.7, 7.8 Hz, 1H), 3.14 (d, *J* = 14.0 Hz, 1H), 2.72 (d, *J* = 12.9 Hz, 1H), 2.52 – 2.34 (m, 3H), 2.29 (s, 3H), 2.27 – 2.19 (m, 1H), 2.09 – 1.99 (m, 2H), 1.88 (dd, *J* = 19.0, 10.8 Hz, 1H), 1.74 – 1.64 (m, 1H), 1.61 (s, 3H), 0.96 (t, *J* = 7.1 Hz, 3H). **^{13}C NMR** (101 MHz, $CDCl_3$) δ 210.0, 168.5, 165.1, 148.0, 134.3, 131.1, 129.2, 128.0, 127.3, 124.4, 116.3, 62.0, 60.9, 52.3, 44.9, 40.8, 36.0, 35.1, 30.6, 23.0, 21.0, 20.7, 13.8. **HRMS** (ESI) m/z: [M^+] Calcd for $C_{23}H_{26}O_5$ 382.1780; found 382.1787. The enantiomeric excess was determined by HPLC using a Chiralpak IG column [*n*-hexanes/2-propanol (80:20)]; flow rate 1 mL/min, λ = 209 nm; τ_{major} = 10.7 min, τ_{minor} = 49.5 min 87% ee); **$[\alpha]_D^{25}$** = +260.2 (c 1.0, $CHCl_3$).

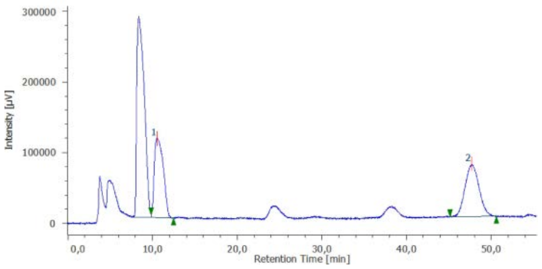

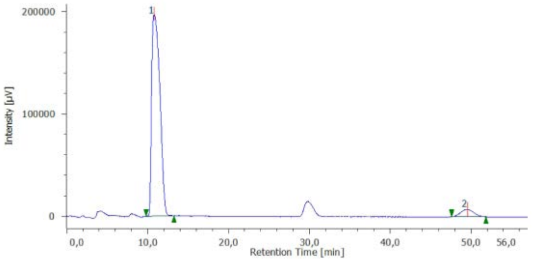

tR [min]	Area [µV·sec]	Height [µV]	Area%	Height%
10,413	13601266	174635	49,681	60,425
48,653	13776131	114378	50,319	39,575

tR [min]	Area [µV·sec]	Height [µV]	Area%	Height%
10,747	74909183	1063453	93,723	96,073
49,480	5016883	43467	6,277	3,927

Ethyl (6a*S*,7*S*,10a*R*)-1-methoxy-9-methyl-3',6-dioxo-10,10a-dihydrospiro[benzo[c]chromene-7,1'-cyclohexane]-6a(6H)-carboxylate, **3e'**.

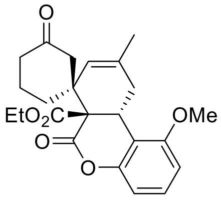

Following the general procedure **B**, starting from ethyl 5-methoxy-2-oxo-2H-chromene-3-carboxylate **2e** (50 mg, 0.2 mmol) and 3-(2-methylallyl)cyclohex-2-en-1-one **1** (63 µL, 0.8 mmol), QN-NH_2 **V** (13 mg, 0.02 mmol), Et_3N (11 mg, 0.04 mmol) and toluene (200 µL, 1M), compound **3e'** was obtained as a white solid (42 mg, 53%; reaction ran for 7 days). The diastereoselectivity ratio of the reaction was 36:64. **Mp**: 145-150 °C. **IR** (neat) ν_{max}: 2959, 1767, 1711, 1468, 1279, 1252, 1211, 1095, 1075, 978, 791 cm^{-1}. **^{1}H NMR** (400 MHz, $CDCl_3$) δ 7.20 (s, 1H), 7.09 (t, *J* = 8.3 Hz, 1H), 6.56 (ddd, *J* = 8.3, 4.1, 0.9 Hz, 2H), 5.50 (q, *J* = 1.7 Hz, 1H), 4.08 (dd, *J* = 10.8, 7.7 Hz, 1H), 4.04 – 3.88 (m, 2H), 3.78 (s, 3H), 3.05 (ddd, *J* = 14.0, 2.6, 1.3 Hz, 1H), 2.63 (d, *J* = 12.8 Hz, 1H), 2.53 – 2.41 (m, 2H), 2.36 – 2.28 (m, 1H), 2.24 – 2.14 (m, 1H), 2.04 – 1.96 (m, 2H), 1.73 (ddd, *J* = 19.0, 10.7, 1.0 Hz, 1H), 1.68 – 1.58 (m, 1H), 1.58 – 1.53 (m, 3H), 0.90 (t, *J* = 7.1 Hz, 3H). **^{13}C NMR** (101 MHz, $CDCl_3$) δ 210.3, 168.5, 165.0, 156.5, 151.1, 131.4, 128.7, 123.9, 116.7, 108.9, 106.5, 61.9, 60.6, 55.9, 52.4, 44.7, 40.8, 33.5, 30.5, 29.7, 23.1, 21.0, 13.8. **HRMS** (ESI) m/z: [M^+] Calcd for $C_{23}H_{26}O_6$ 398.1729; found 398.1714. The enantiomeric excess was determined by HPLC using a Chiralpak IG column [*n*-hexanes/2-propanol (90:10)]; flow rate 1 mL/min, λ = 209 nm; τ_{major} = 24.1 min, τ_{minor} = 55.9 min 96% *ee*); **$[\alpha]_D^{25}$** = +177.6 (c 0.8, $CHCl_3$).

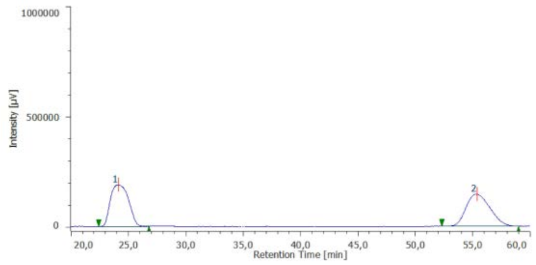

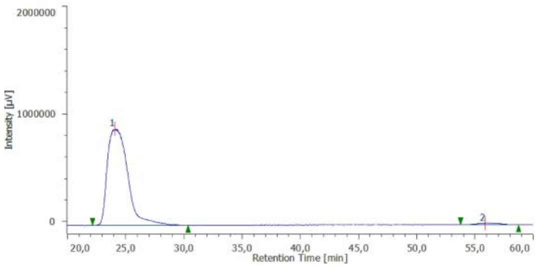

tR [min]	Area [µV·sec]	Height [µV]	Area%	Height%
24,147	20852248	189333	49,524	56,352
55,347	21252735	146651	50,476	43,648

tR [min]	Area [µV·sec]	Height [µV]	Area%	Height%
24,107	108695850	895933	98,064	98,426
55,893	2146147	14329	1,936	1,574

Ethyl (6a*S*,7*S*,10a*R*)-3-chloro-9-methyl-3',6-dioxo-10,10a-dihydrospiro[benzo[c]chromene-7,1'-cyclohexane]-6a(6H)-carboxylate, **3h'**.

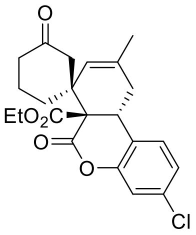

Following the general procedure **B**, starting from ethyl 7-chloro-2-oxo-2*H*-1-benzopyran-3-carboxylate **2h** (51 mg, 0.2 mmol) and 3-(2-methylallyl)cyclohex-2-en-1-one **1** (63 µL, 0.4 mmol), QN-NH_2 **V** (13 mg, 0.02 mmol), Et_3N (11 mg, 0.04 mmol) and toluene (200 µL, 1M), compound **3h'** was obtained as white solid (28 mg, 35%, the reaction ran for 7 days). The diastereoselectivity ratio of the reaction was 38:62. **Mp**: 138-143 °C. **IR** (neat) ν_{max}: 1786, 1732, 1701, 1225, 1117, 1045, 850, 816 cm^{-1}. **^{1}H NMR** (400 MHz, $CDCl_3$) δ 7.14 (d, *J* = 8.0 Hz, 1H), 7.10 – 7.03 (m, 2H), 5.59 (q, *J* = 1.7 Hz, 1H), 4.14 – 3.96 (m, 2H), 3.69 (dd, *J* = 10.8, 7.7 Hz, 1H), 3.11 (ddd, *J* = 14.0, 2.6, 1.4 Hz, 1H), 2.70 (d, *J* = 12.9 Hz, 1H), 2.54 – 2.36 (m, 3H), 2.32 – 2.18 (m, 1H), 2.11 – 2.01 (m, 2H), 1.87 (dddd, *J* = 19.0, 10.8, 2.1, 1.1 Hz, 1H), 1.69 (ddd, *J* = 14.0, 9.2, 8.0 Hz, 1H), 1.63 (q, *J* = 1.1 Hz, 3H), 1.02 (t, *J* = 7.1 Hz, 3H). **^{13}C NMR** (101 MHz, $CDCl_3$) δ 209.9, 168.3, 164.3, 150.7, 13427, 131.1, 128.7, 126.3, 124.9, 124.5, 117.2, 62.5, 60.8, 52.3, 45.0, 40.9, 35.8, 35.0, 30.6, 23.1, 14.0. **HRMS** (ESI) m/z: [M^+] Calcd for $C_{22}H_{23}ClO_5$ 402.1234; found 402.1218. The enantiomeric excess was determined by HPLC using a Chiralpak IA column [*n*-hexanes/2-propanol (80:20)]; flow rate 1 mL/min, λ = 214 nm; τ_{major} = 7.7 min, τ_{minor} = 34.2 min 77% ee). **$[\alpha]_D^{25}$** = +63.0 (c 0.6, $CHCl_3$).

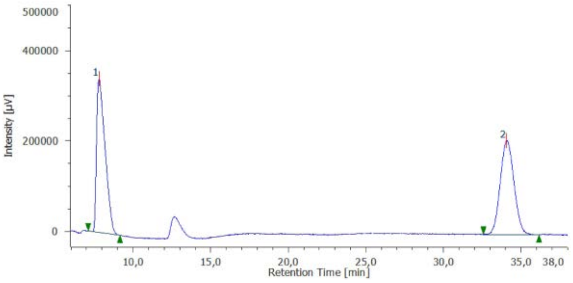

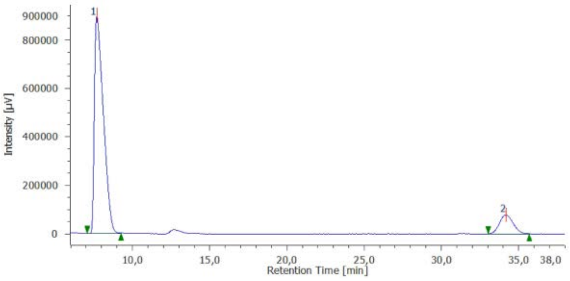

tR [min]	Area [µV·sec]	Height [µV]	Area%	Height%
7,813	12857556	339857	50,369	62,177
34,053	12669028	206740	49,631	37,823

tR [min]	Area [µV·sec]	Height [µV]	Area%	Height%
7,707	35965820	899522	88,275	92,012
34,187	4777210	78093	11,725	7,988

6 NMR Spectra.

6.1 NMR Spectra of 3a-3p, 5 and 7.

^{1}H NMR (300 MHz, $CDCl_3$) of **3a.**

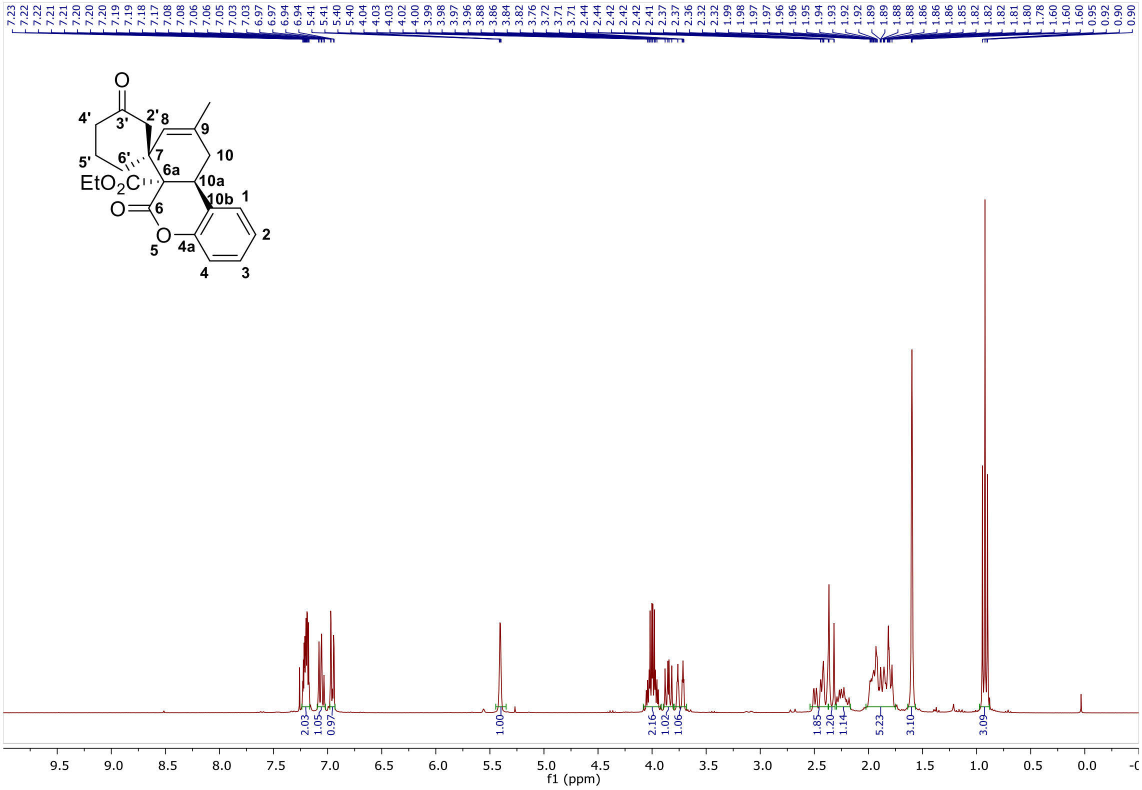

^{13}C NMR (75 MHz, $CDCl_3$) of **3a**.

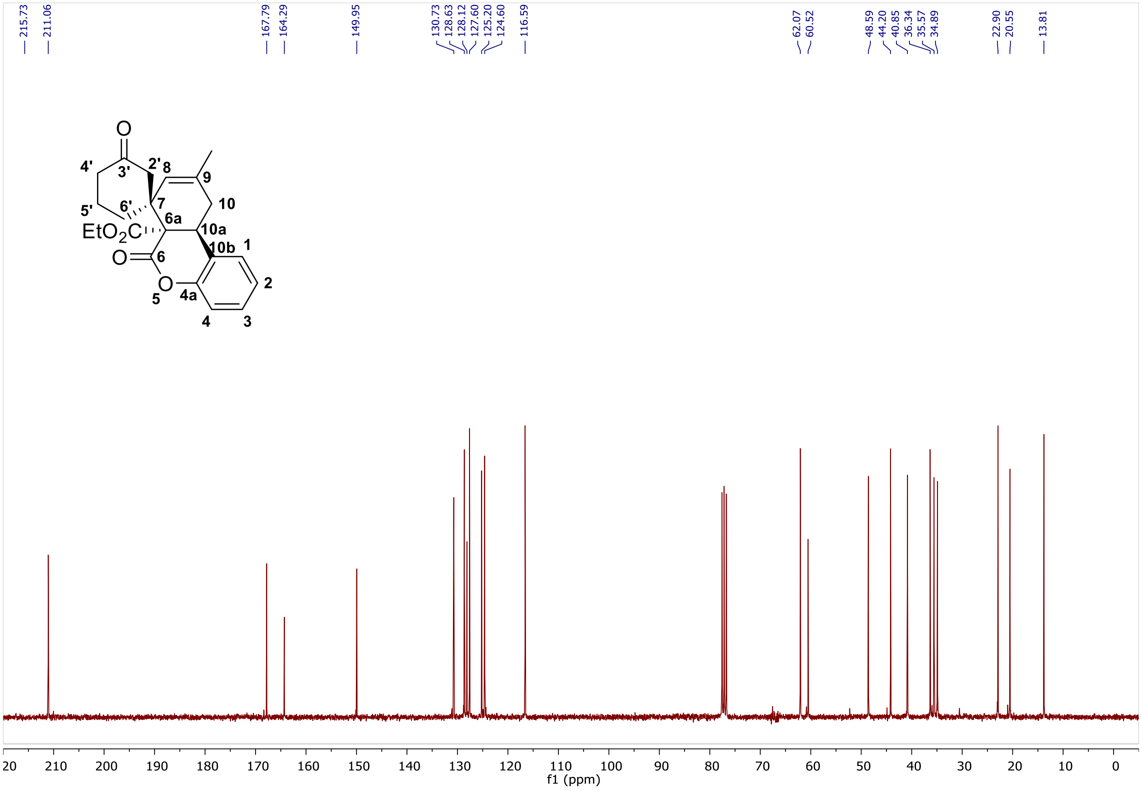

COSY of **3a**.

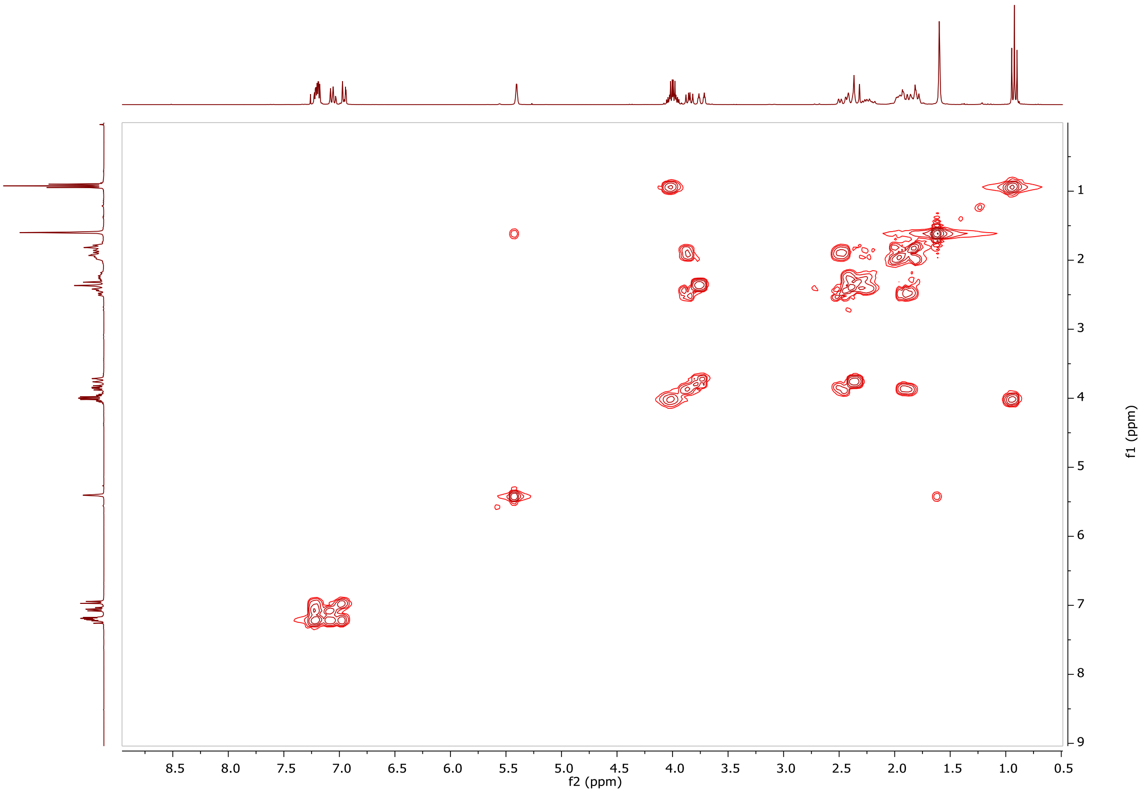

DEPT of **3a**.

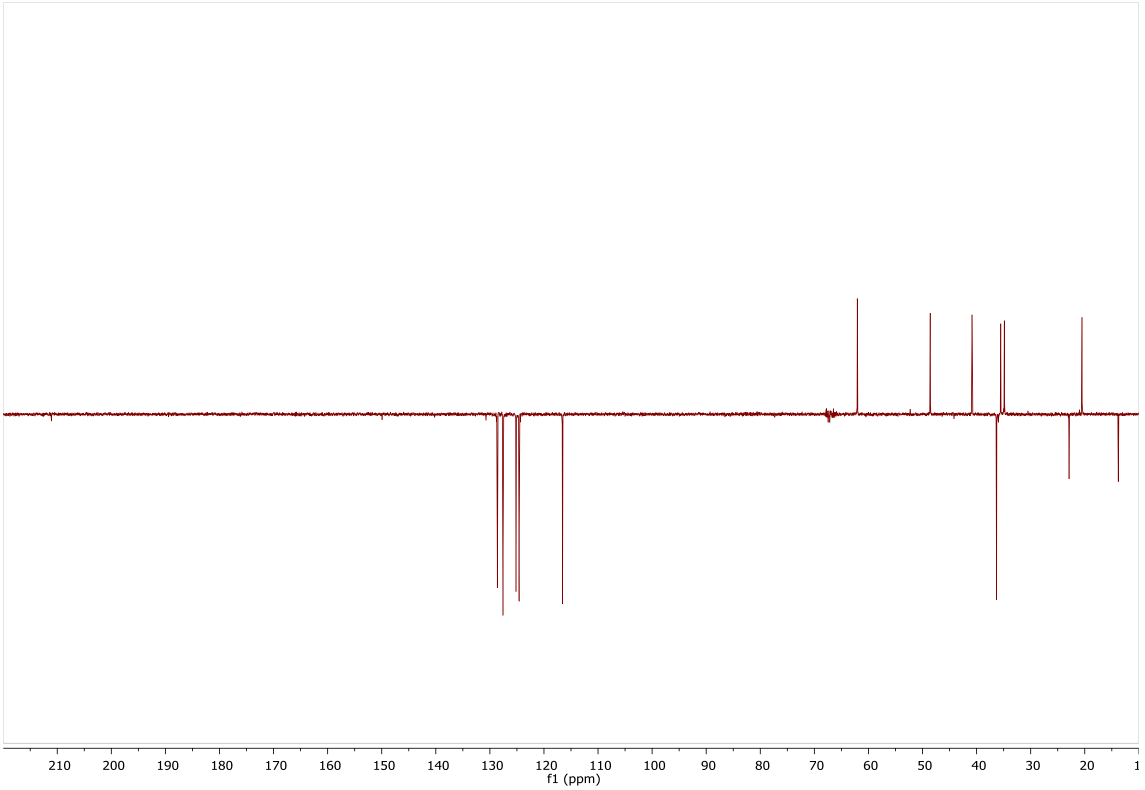

HSQC of **3a**.

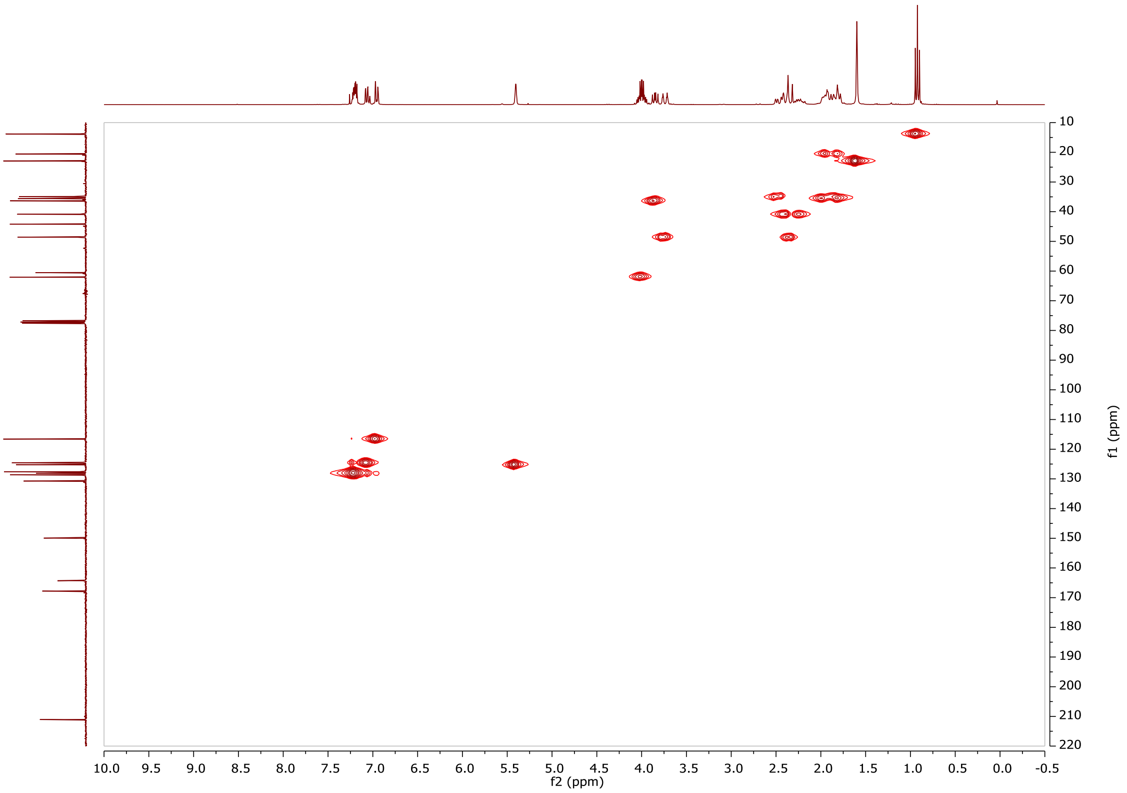

10
20
30
40
50
60
70
80
90
100
110
120
130
140
150
160
170
180
190
200
210
220
f1 (ppm)
10.0
9.5
9.0
8.5
8.0
7.5
7.0
6.5
6.0
5.5
5.0
4.5
4.0
3.5
3.0
2.5
2.0
1.5
1.0
0.5
0.0
-0.5
f2 (ppm)

^{1}H NMR (400 MHz, $CDCl_3$) of **3b**.

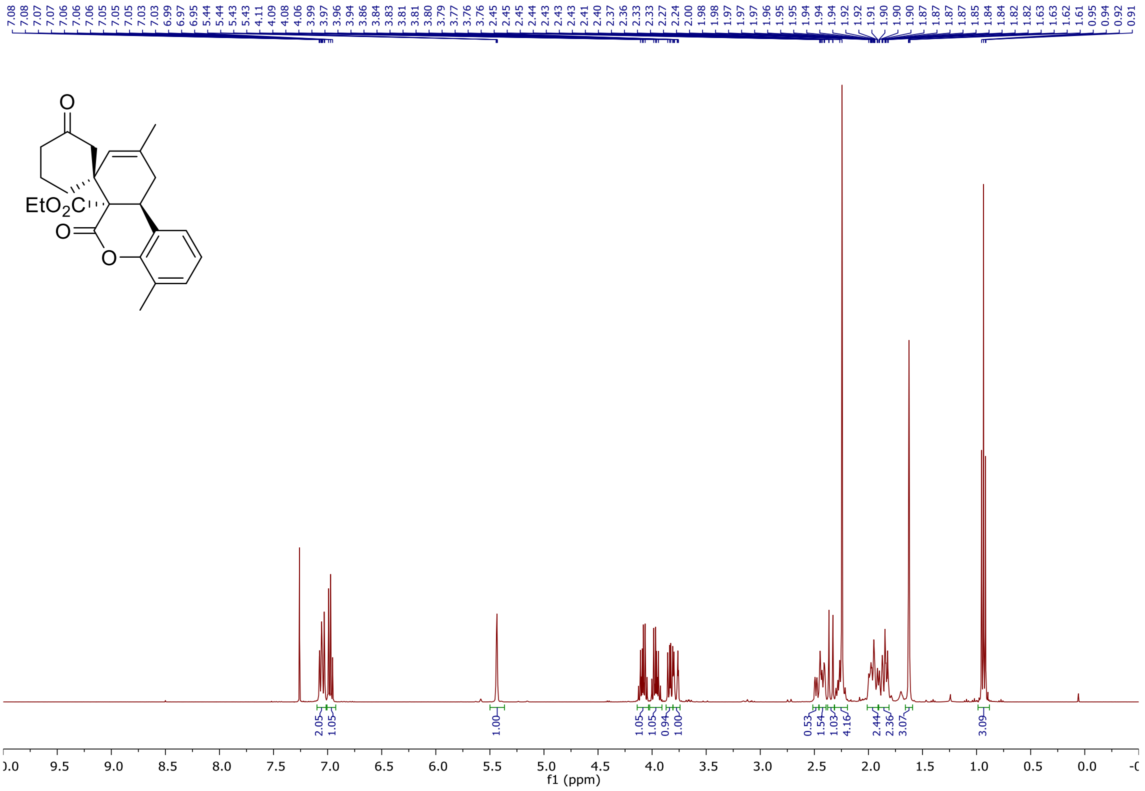

^{13}C MR (101 MHz, $CDCl_3$) of **3b**.

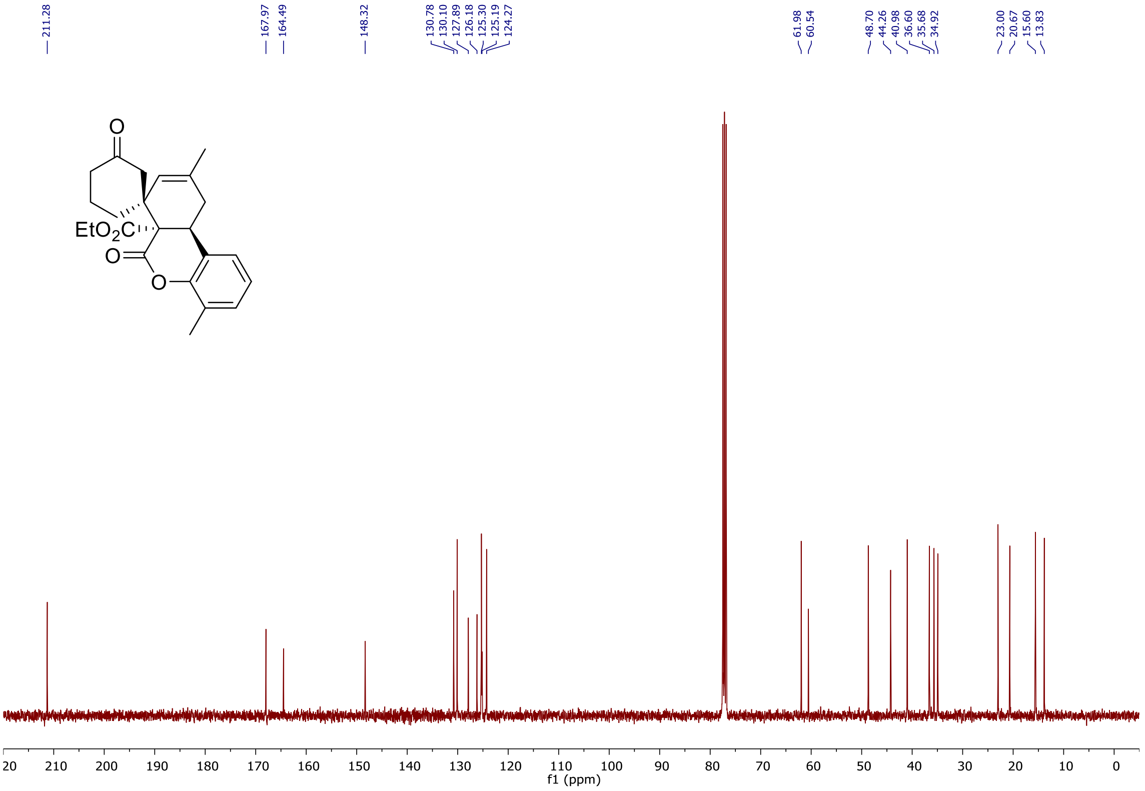

^{1}H NMR (400 MHz, $CDCl_3$) of **3c**.

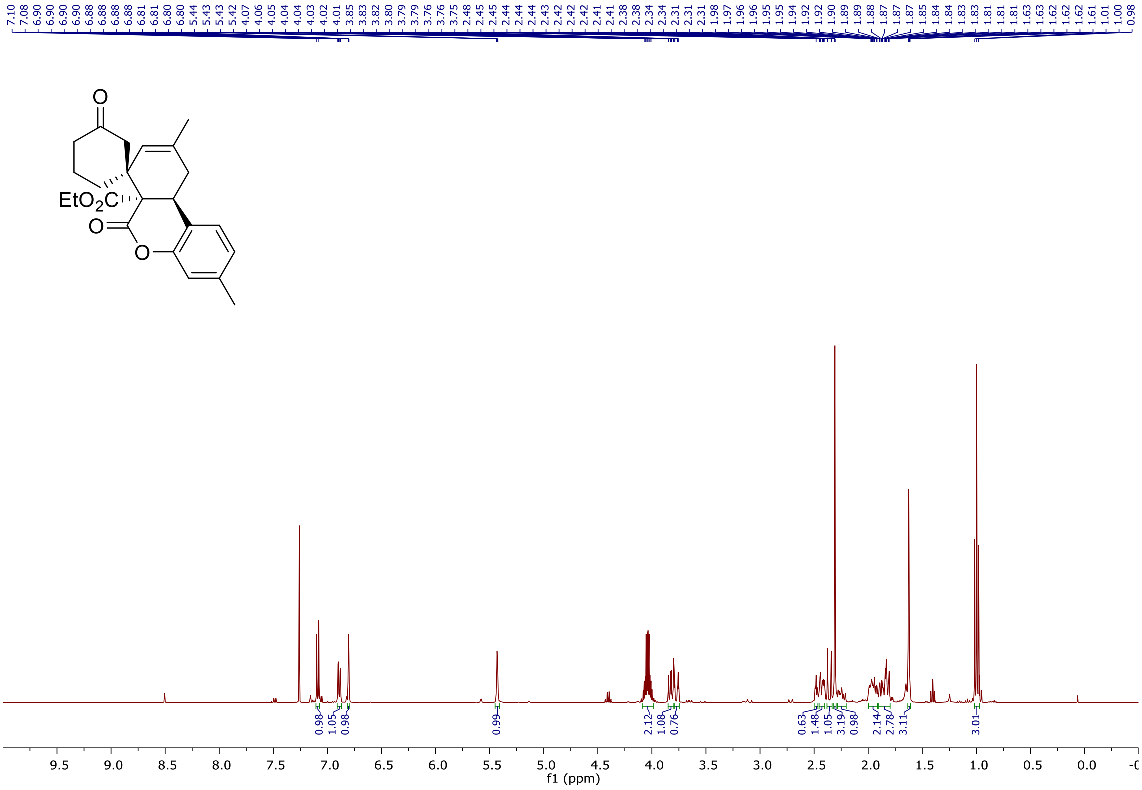

^{13}C NMR (101 MHz, $CDCl_3$) of **3c**.

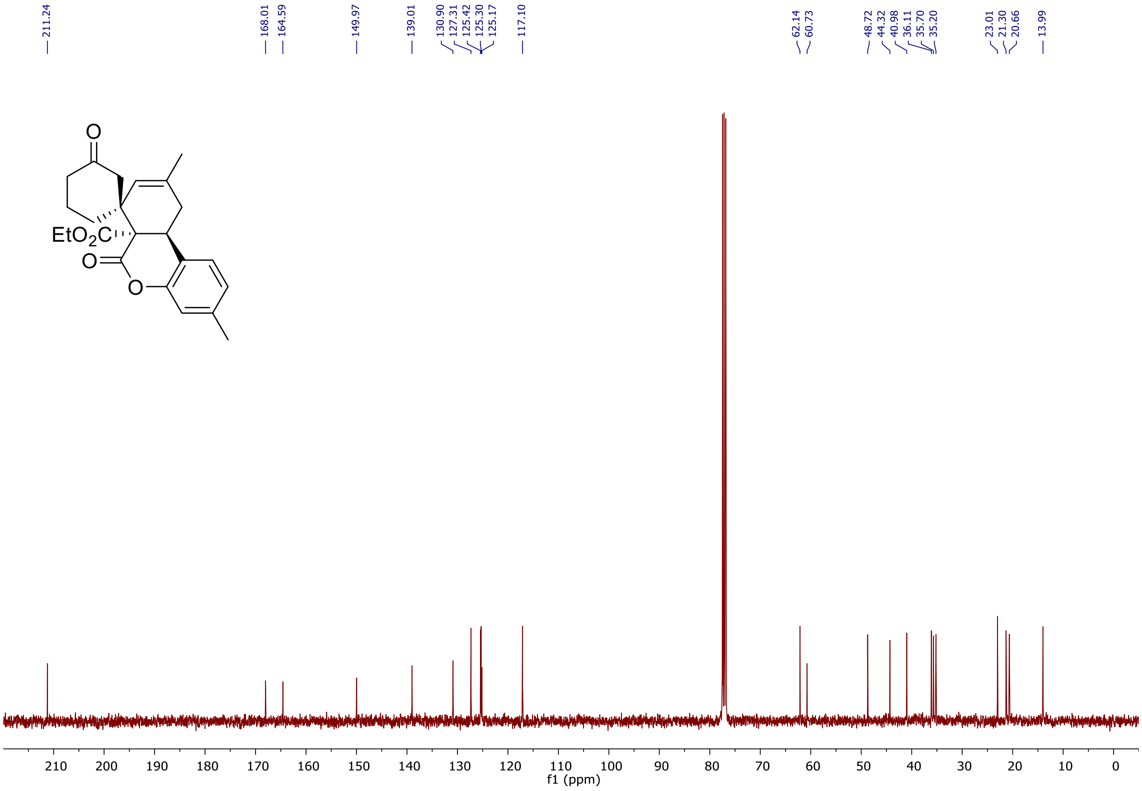

^{1}H NMR (400 MHz, $CDCl_3$) of **3d**.

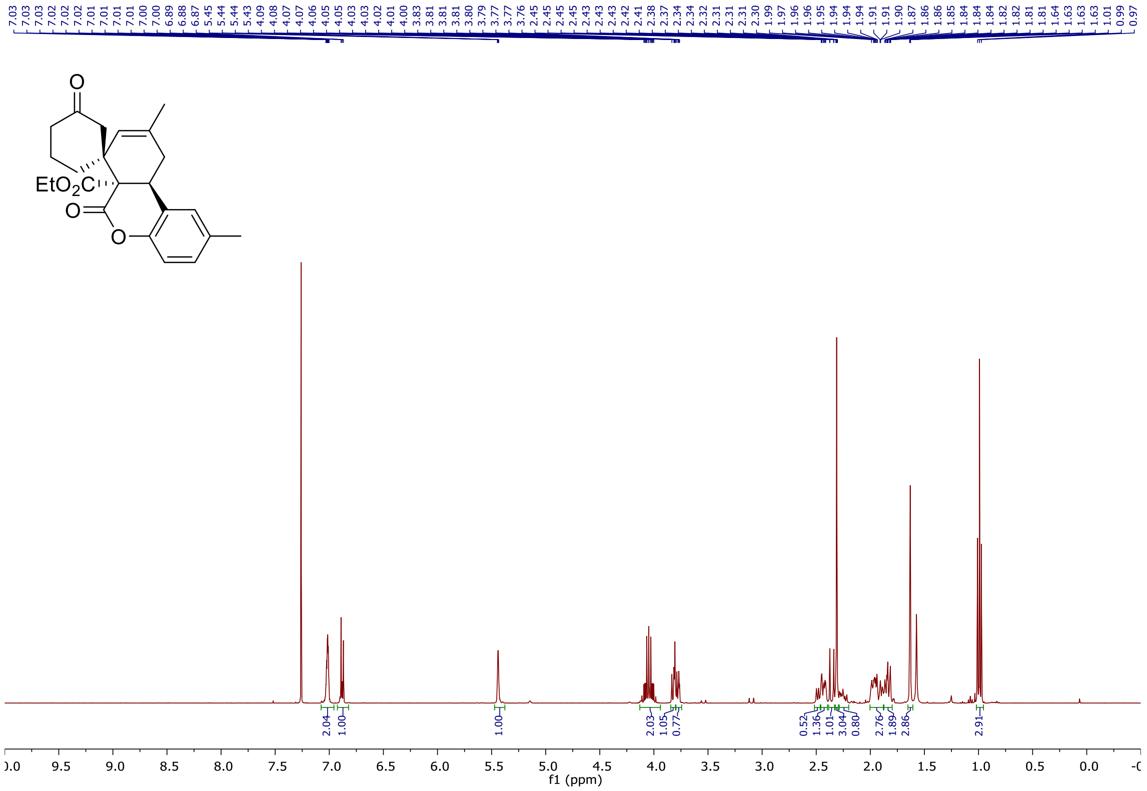

^{13}C NMR (101 MHz, $CDCl_3$) of **3d**.

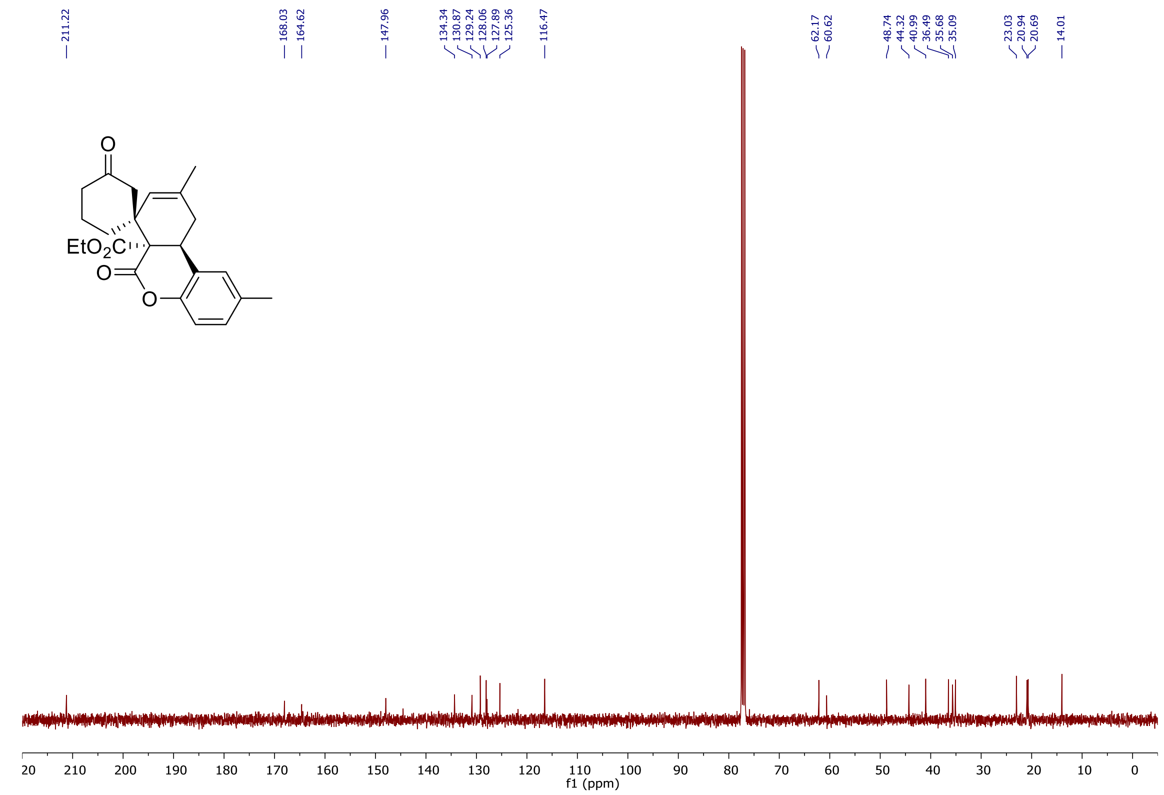

^{1}H NMR (400 MHz, $CDCl_3$) of **3e**.

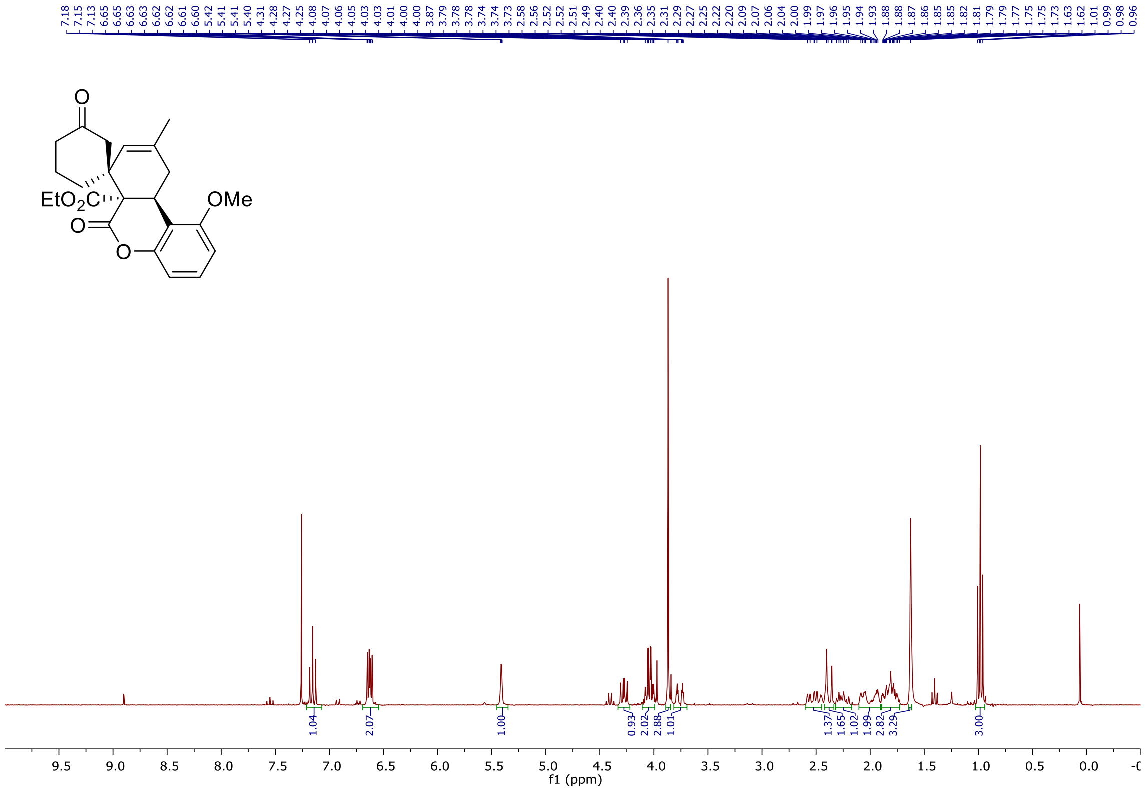

^{13}C NMR (101 MHz, $CDCl_3$) of **3e**.

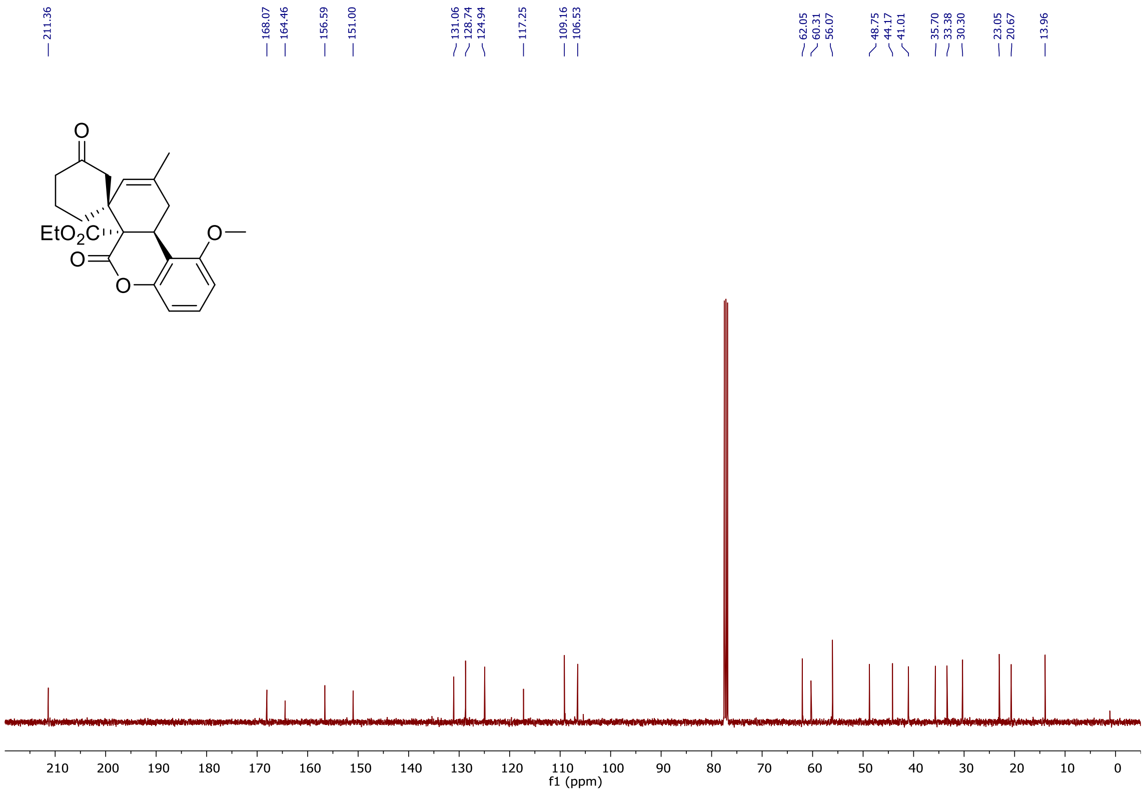

¹H NMR (400 MHz, $CDCl_3$) of **3f**.

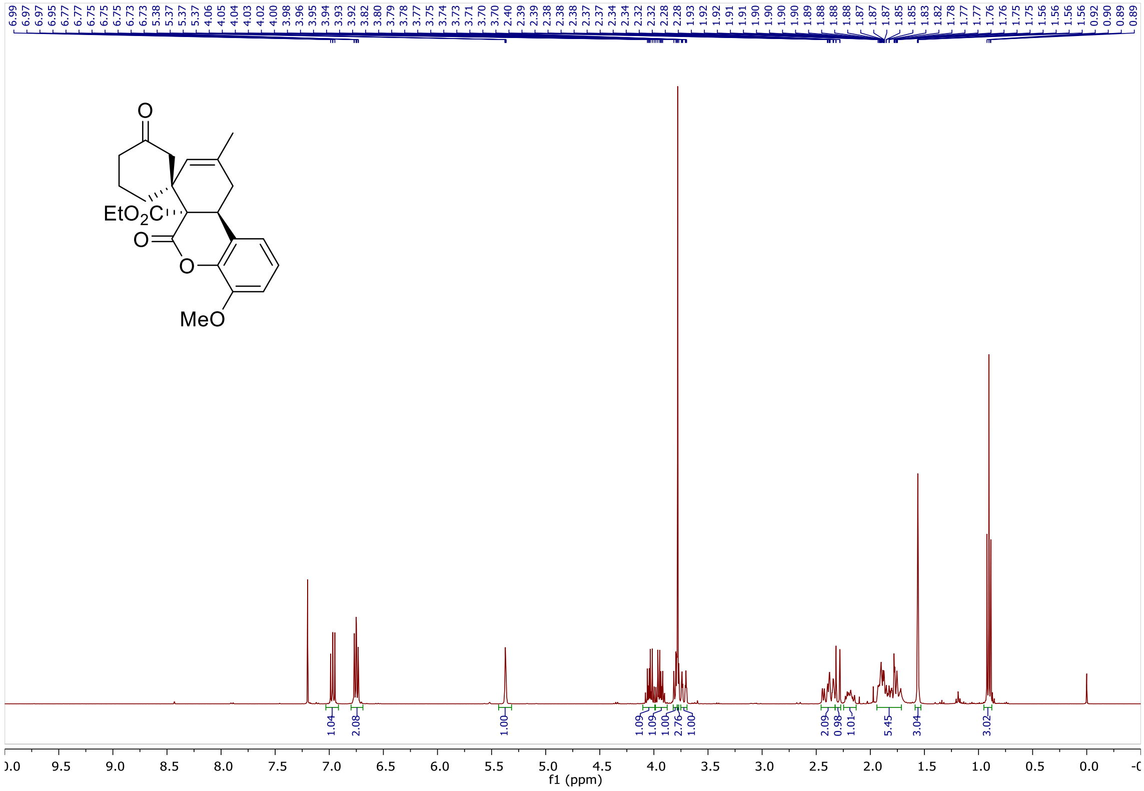

¹³C NMR (101 MHz, $CDCl_3$) of **3f**.

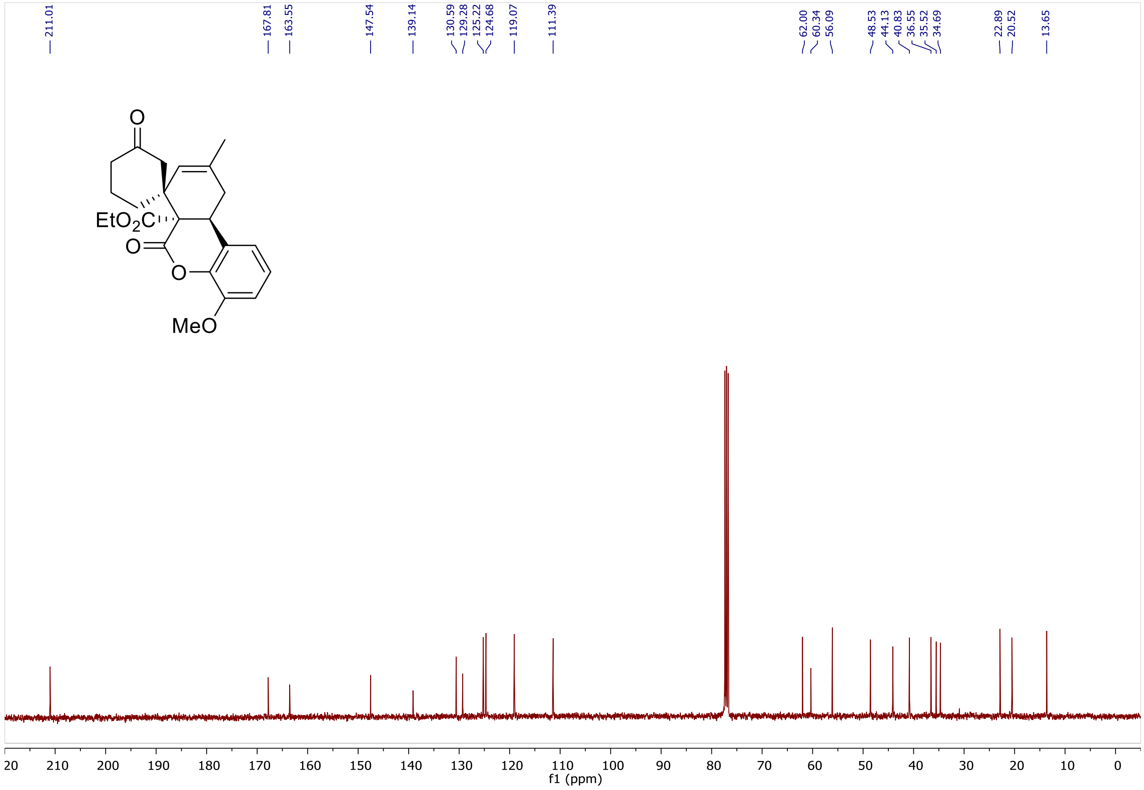

^{1}H NMR (400 MHz, $CDCl_3$) of **3g**.

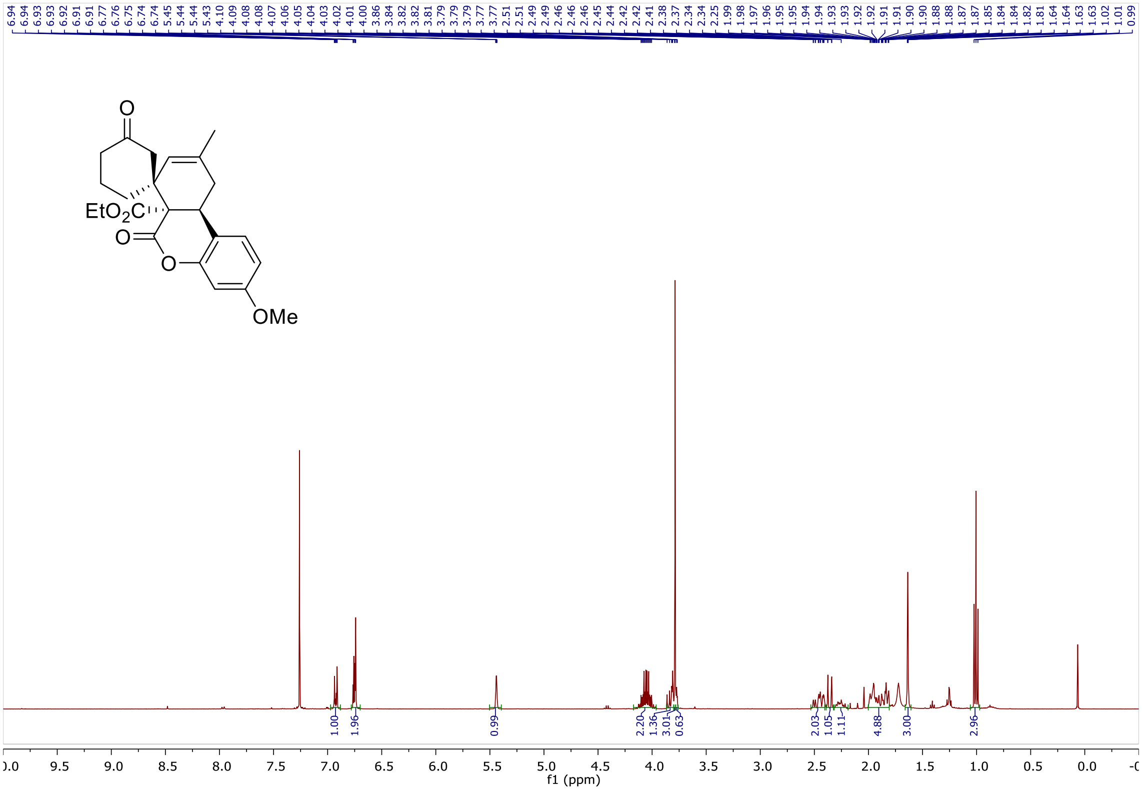

^{13}C NMR (101 MHz, $CDCl_3$) of **3g**.

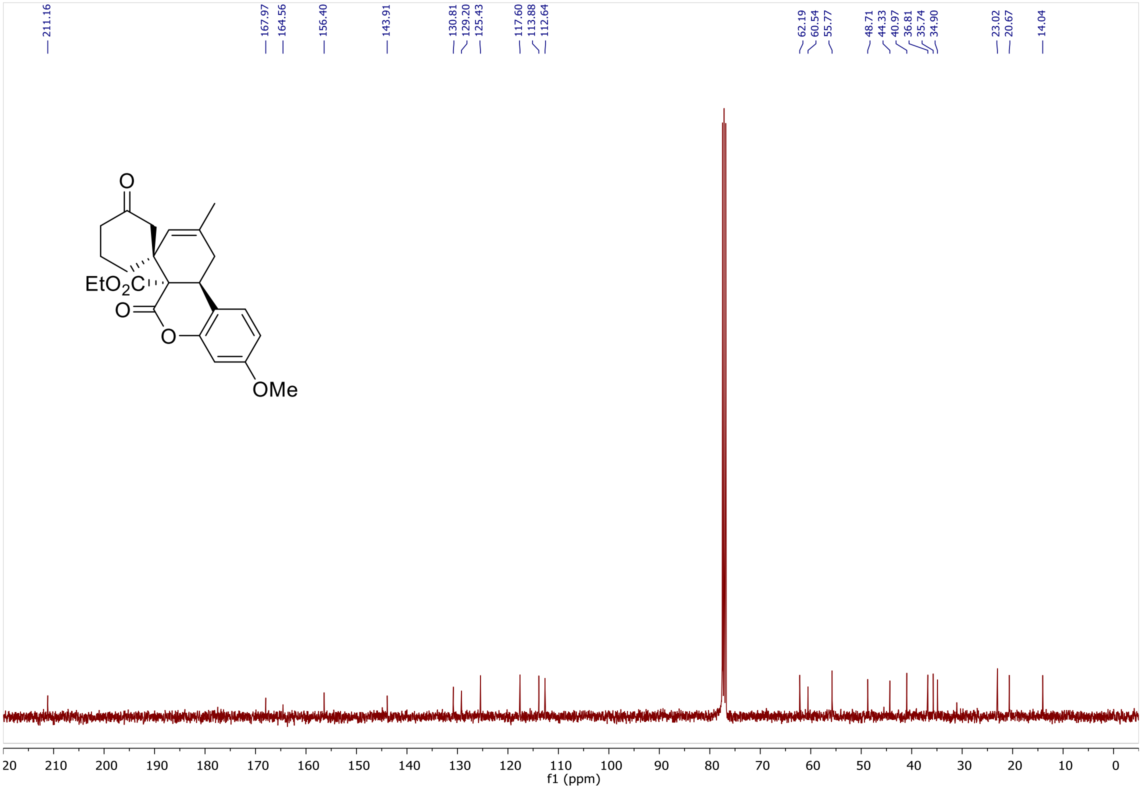

^{1}**H NMR** (400 MHz, $CDCl_3$) of **3h**.

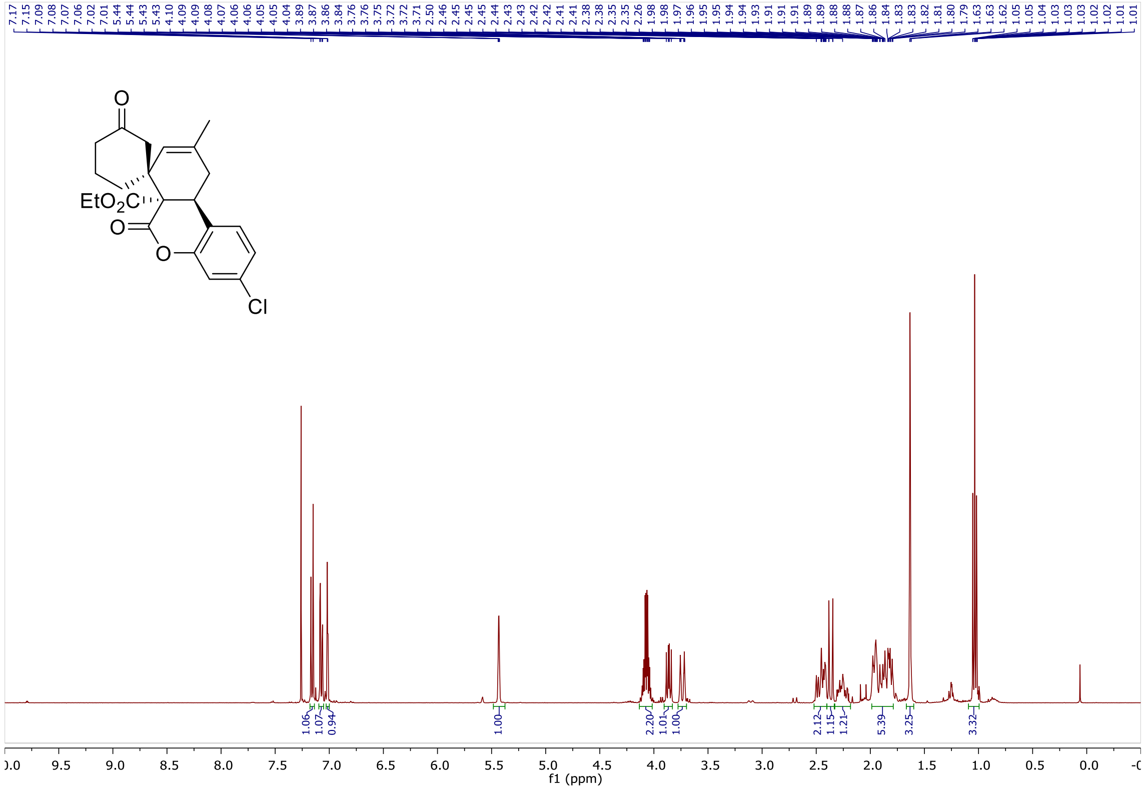

^{13}**C NMR** (101 MHz, $CDCl_3$) of **3h**.

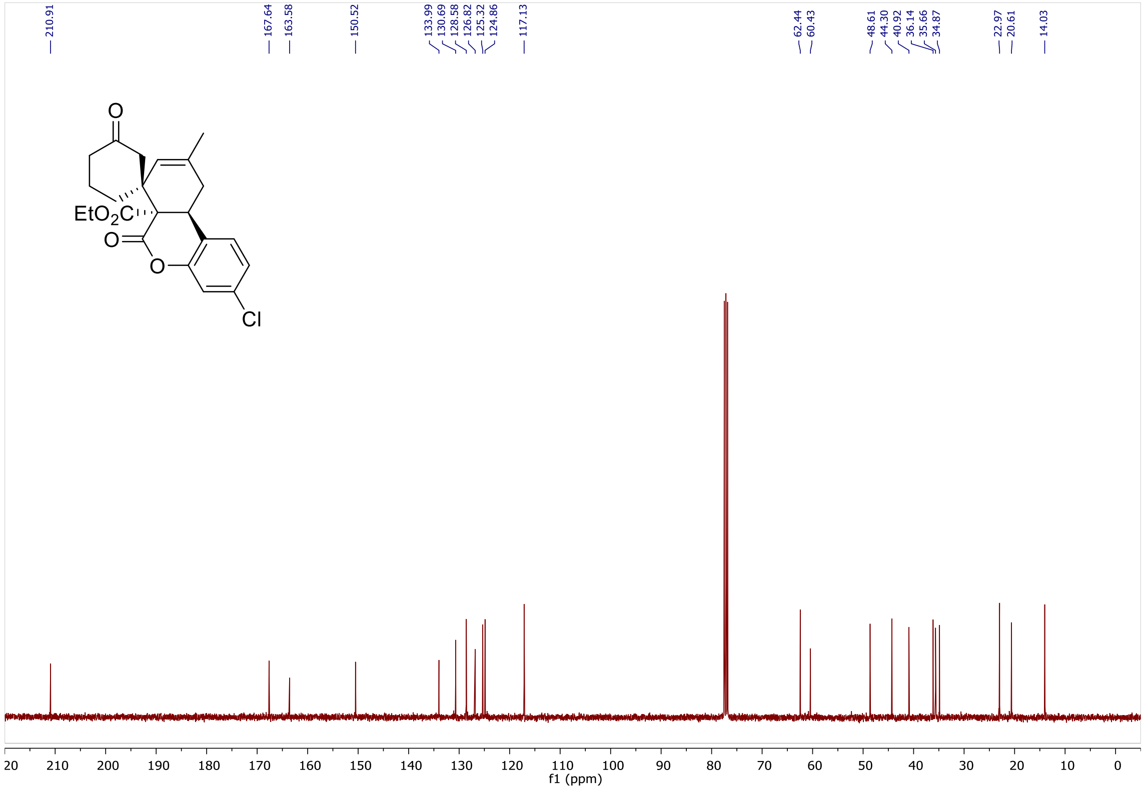

^{1}H NMR (400 MHz, $CDCl_3$) of **3i**.

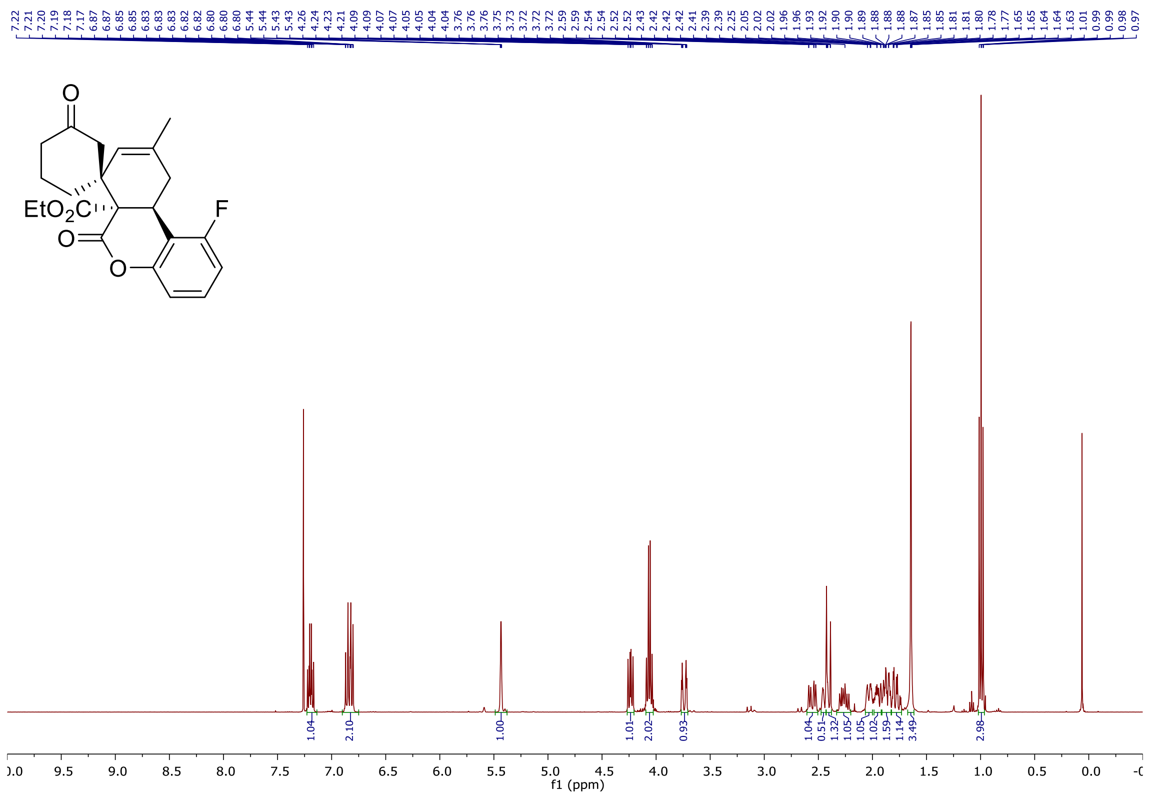

^{13}C NMR (101 MHz, $CDCl_3$) of **3i**.

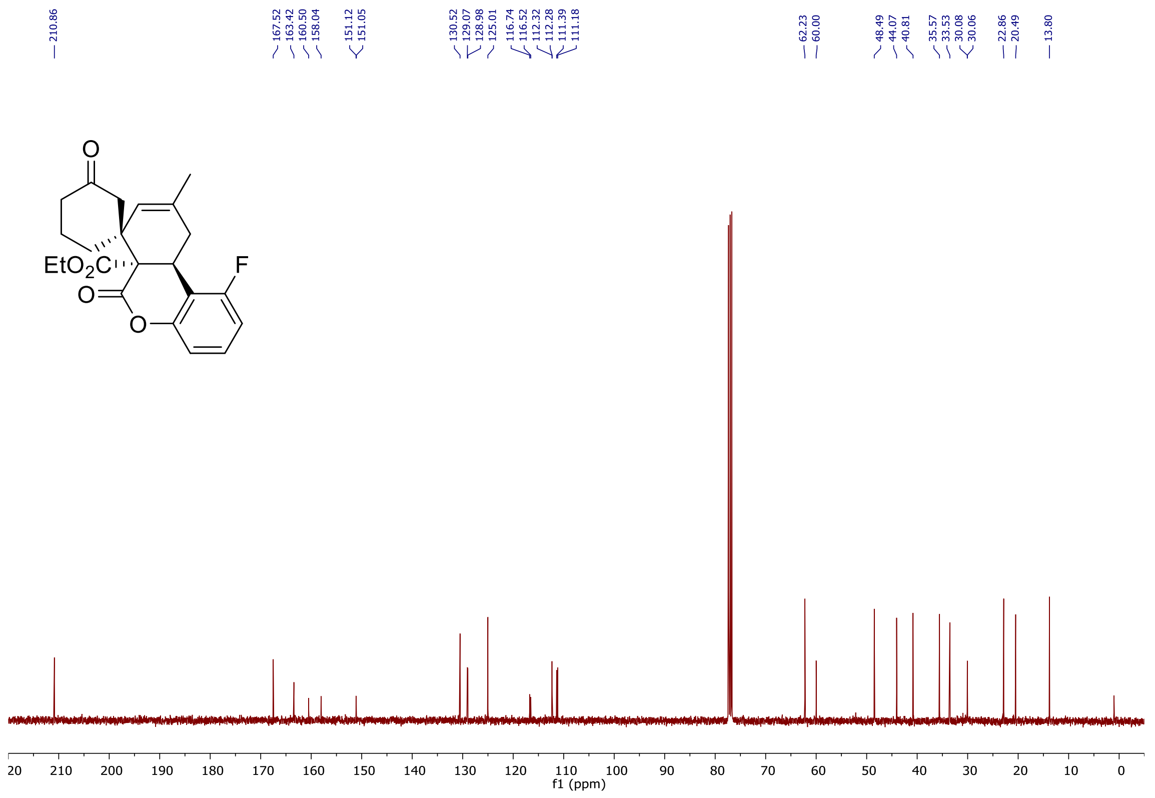

^{19}F NMR (377 MHz, $CDCl_3$) of **3i**.

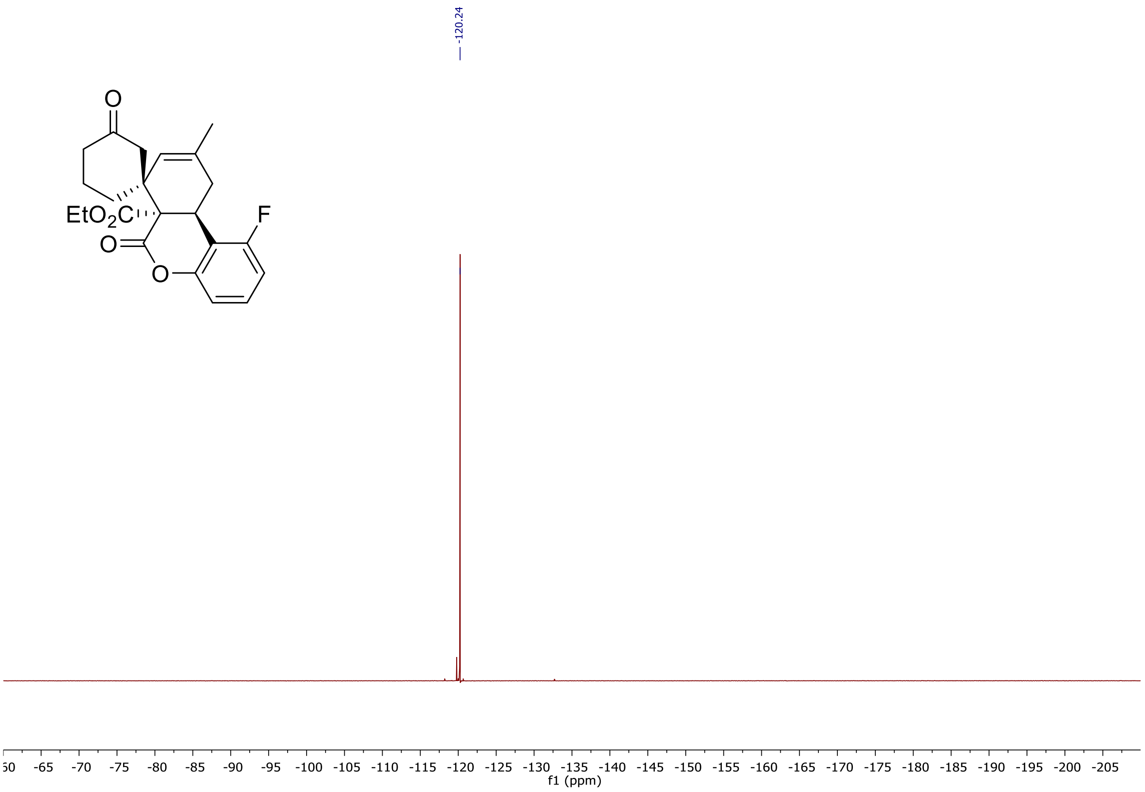

^{1}H NMR (400 MHz, $CDCl_3$) of **3j**.

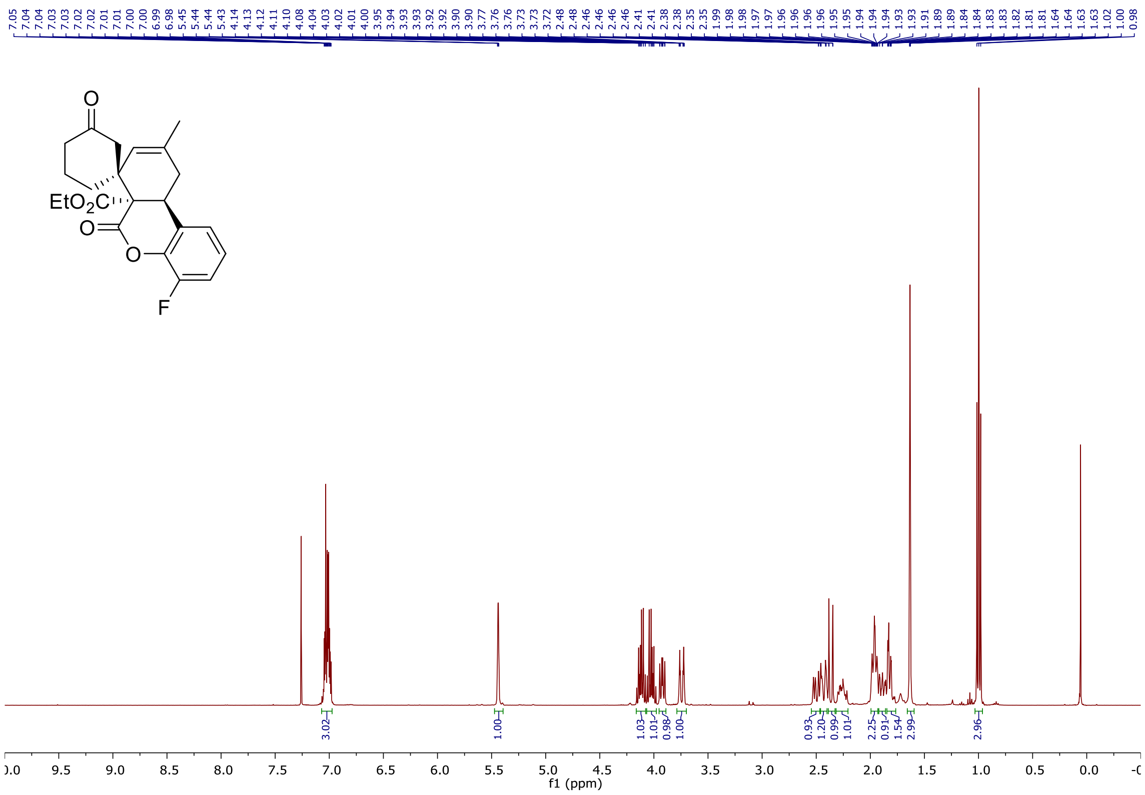

^{13}C NMR (101 MHz, $CDCl_3$) of **3j.**

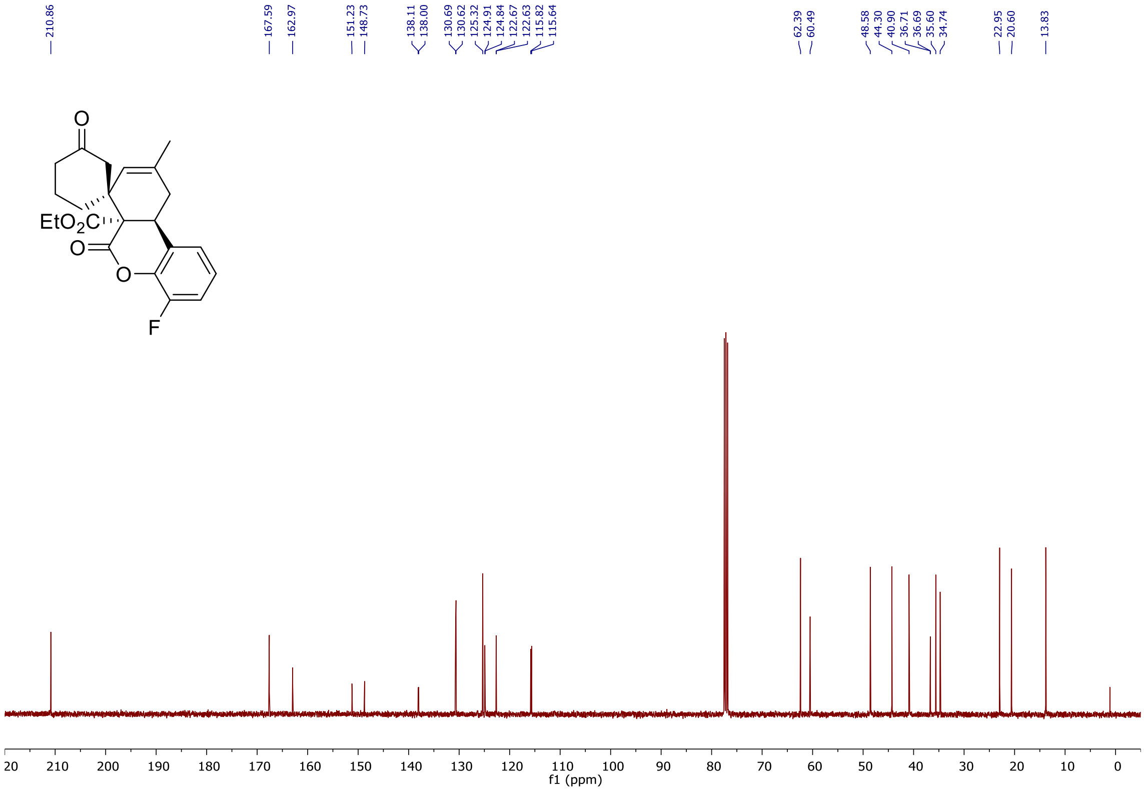

^{19}F NMR (377 MHz, $CDCl_3$) of **3j**.

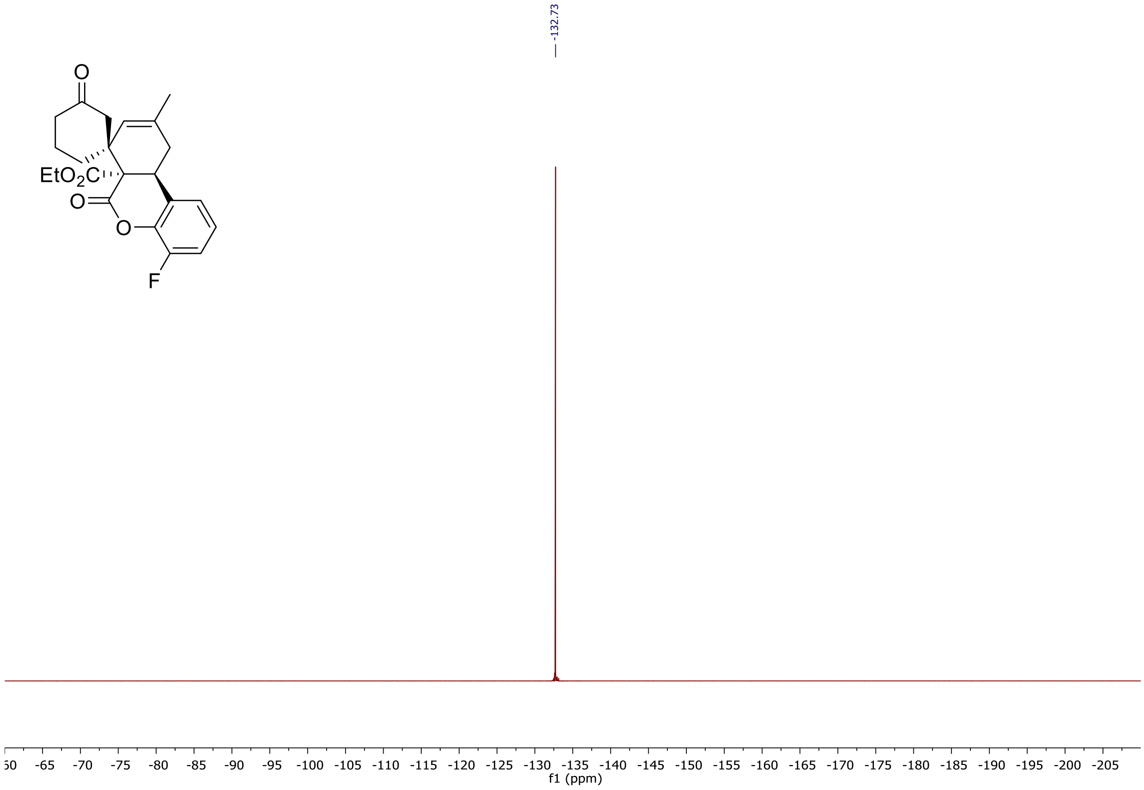

^{1}H NMR (400 MHz, CDCl₃) of **3k**.

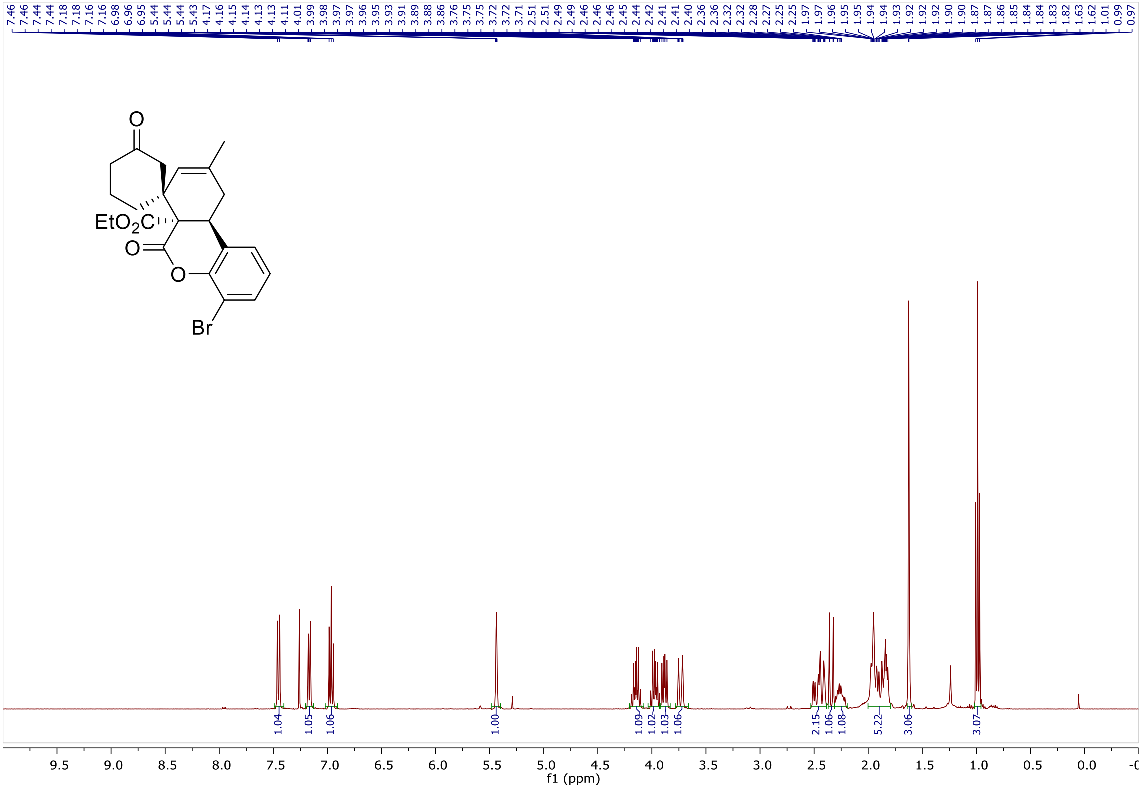

^{13}C NMR (101 MHz, CDCl₃) of **3k**.

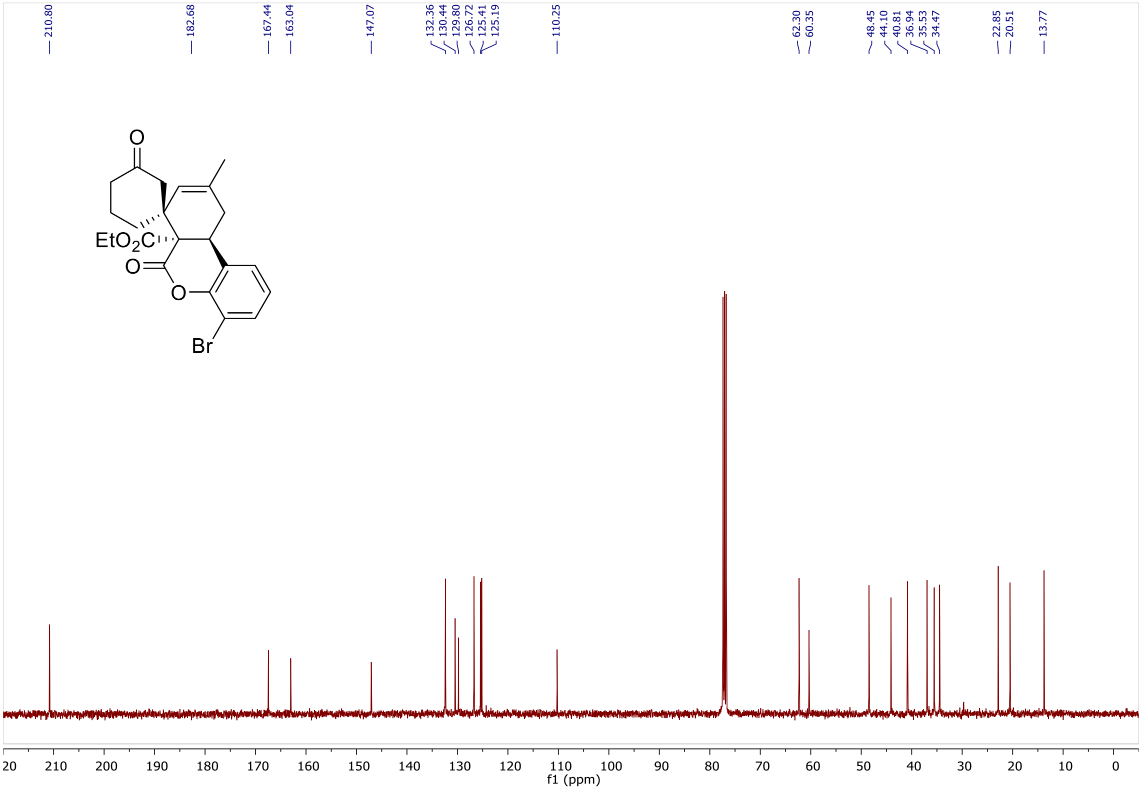

^{1}H NMR (400 MHz, $CDCl_3$) of **3l**.

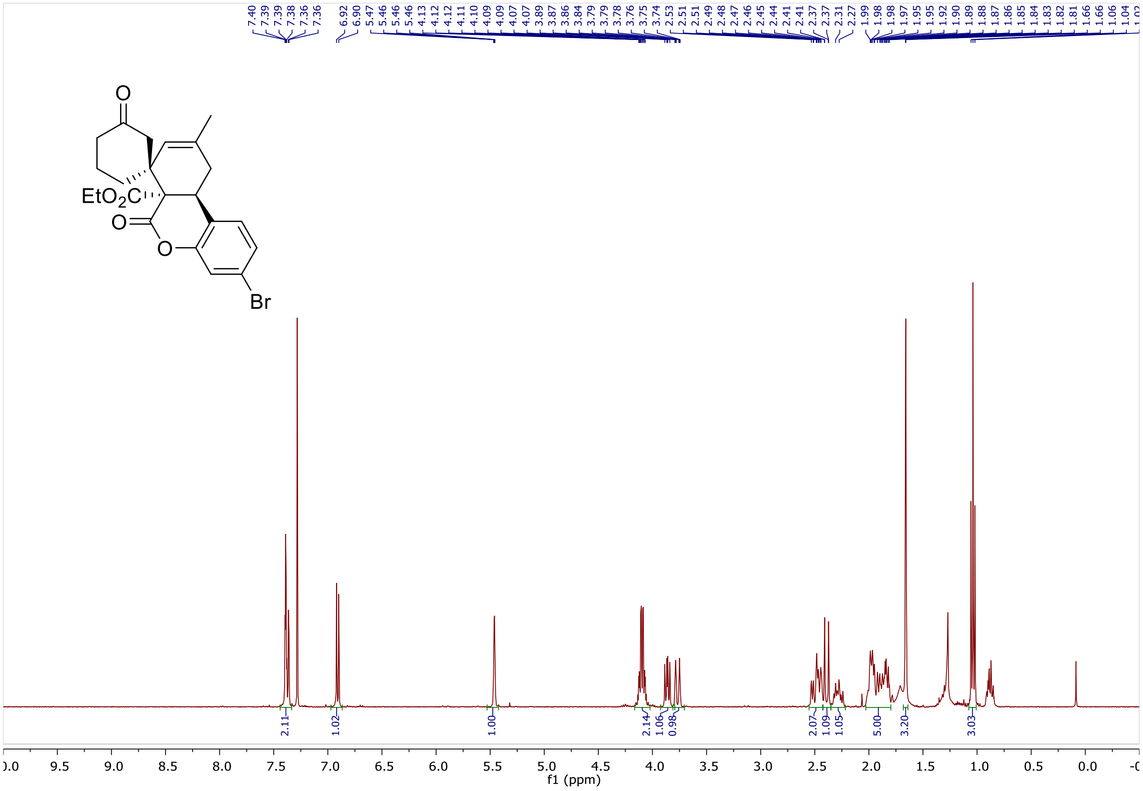

^{13}C NMR (101 MHz, $CDCl_3$) of **3l**.

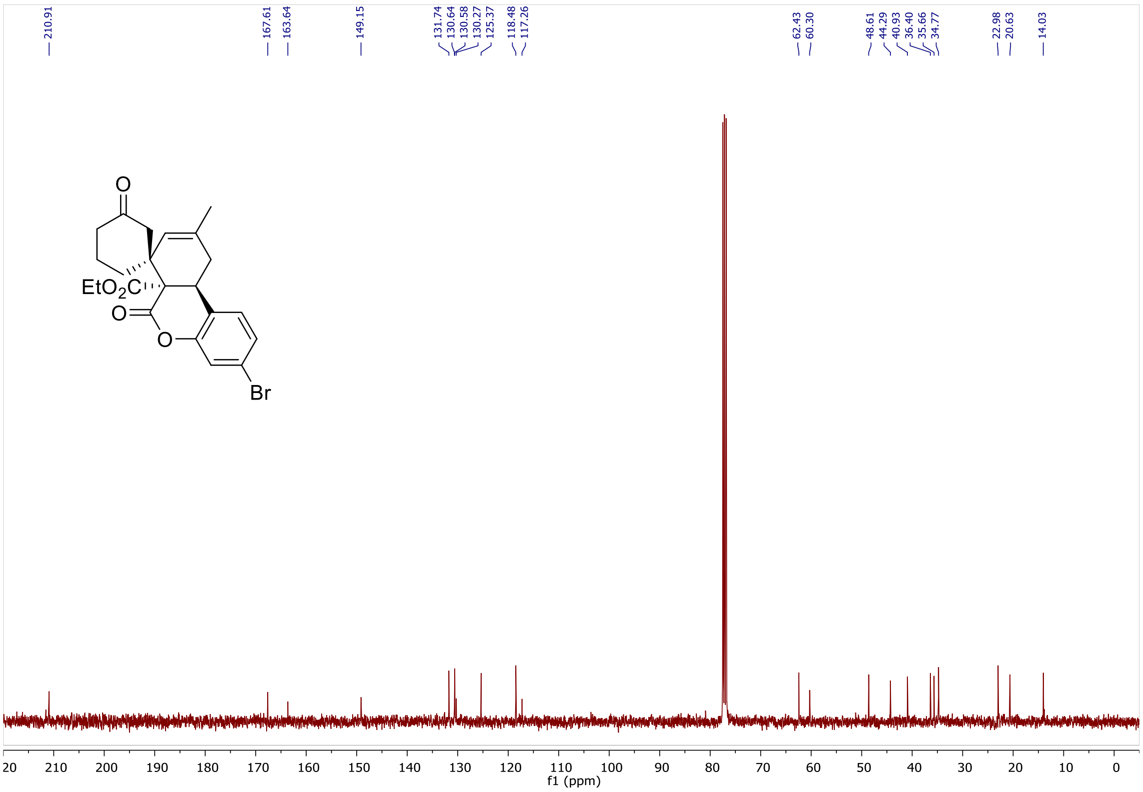

^{1}H NMR (300 MHz, $CDCl_3$) **3m**.

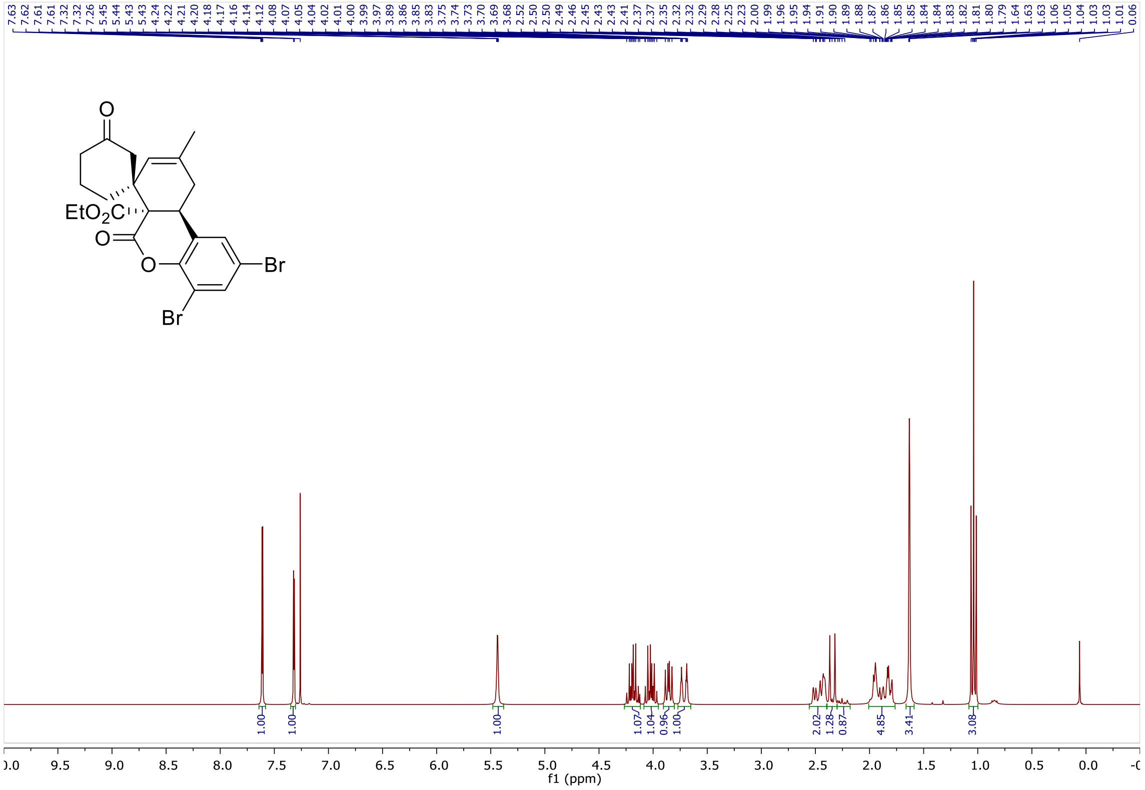

^{13}C NMR (101 MHz, $CDCl_3$) of **3m**.

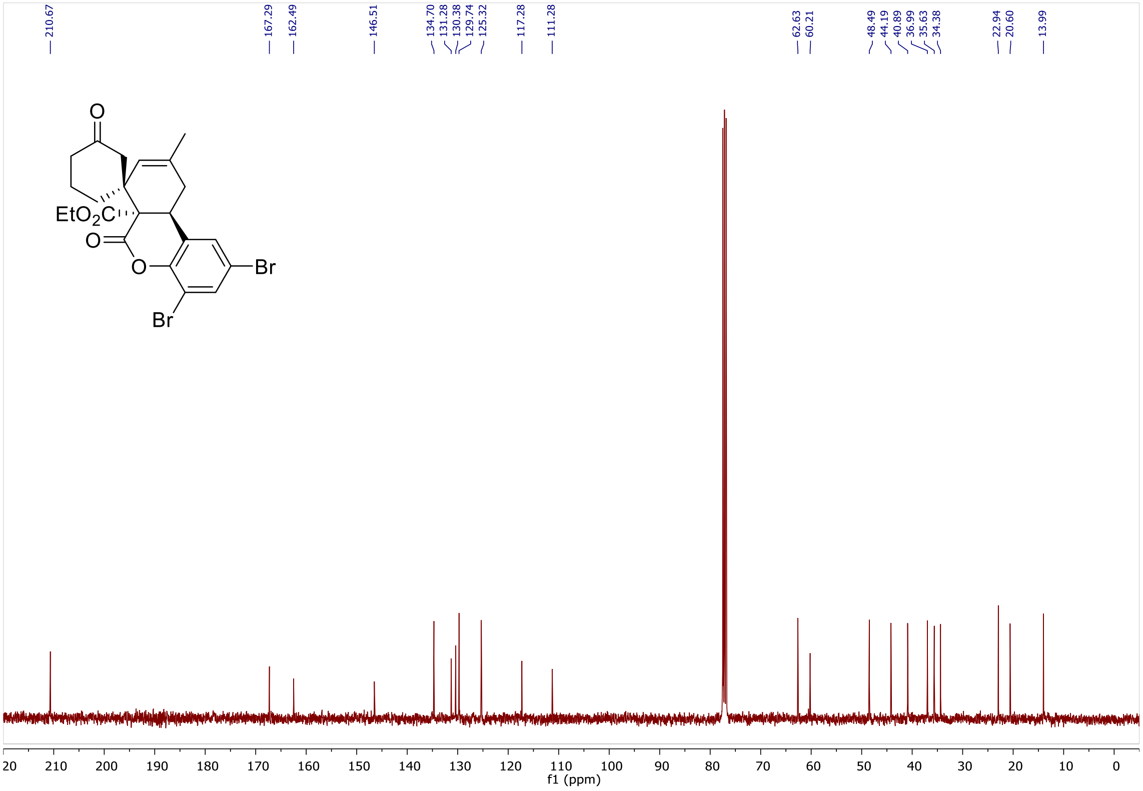

^{1}H NMR (400 MHz, $CDCl_3$) of **3n**.

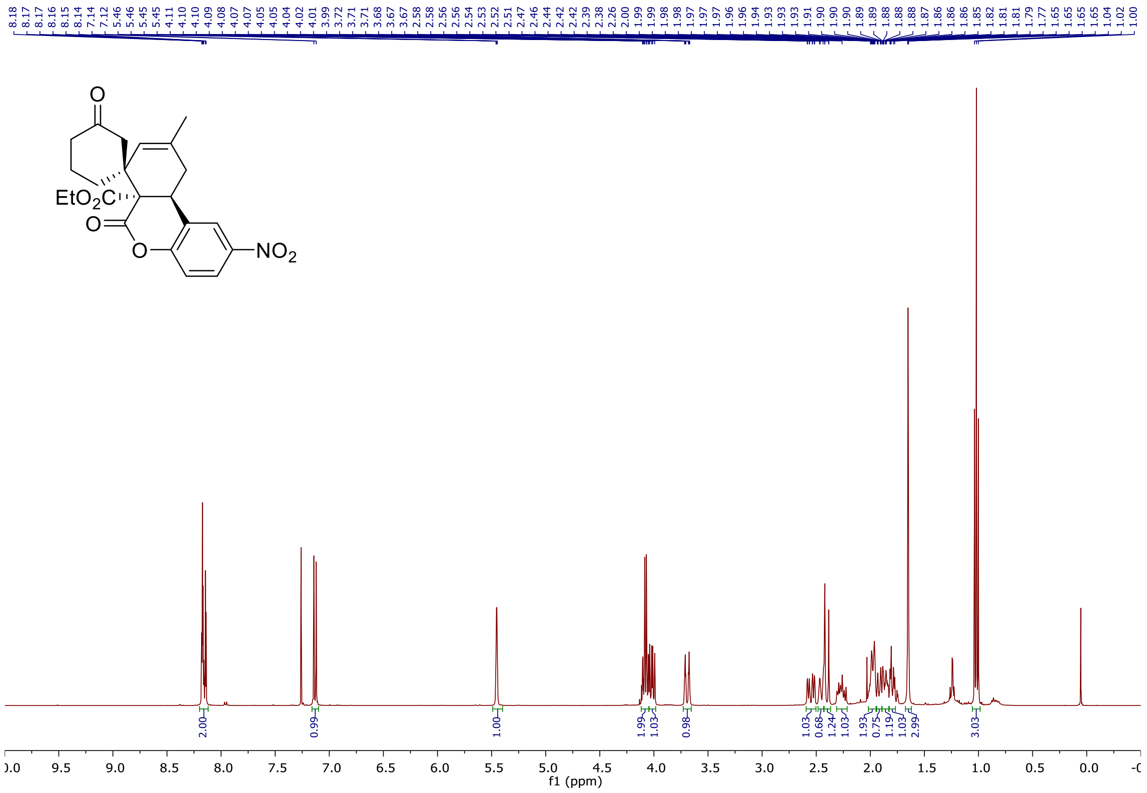

^{13}C NMR (101 MHz, $CDCl_3$) of **3n**.

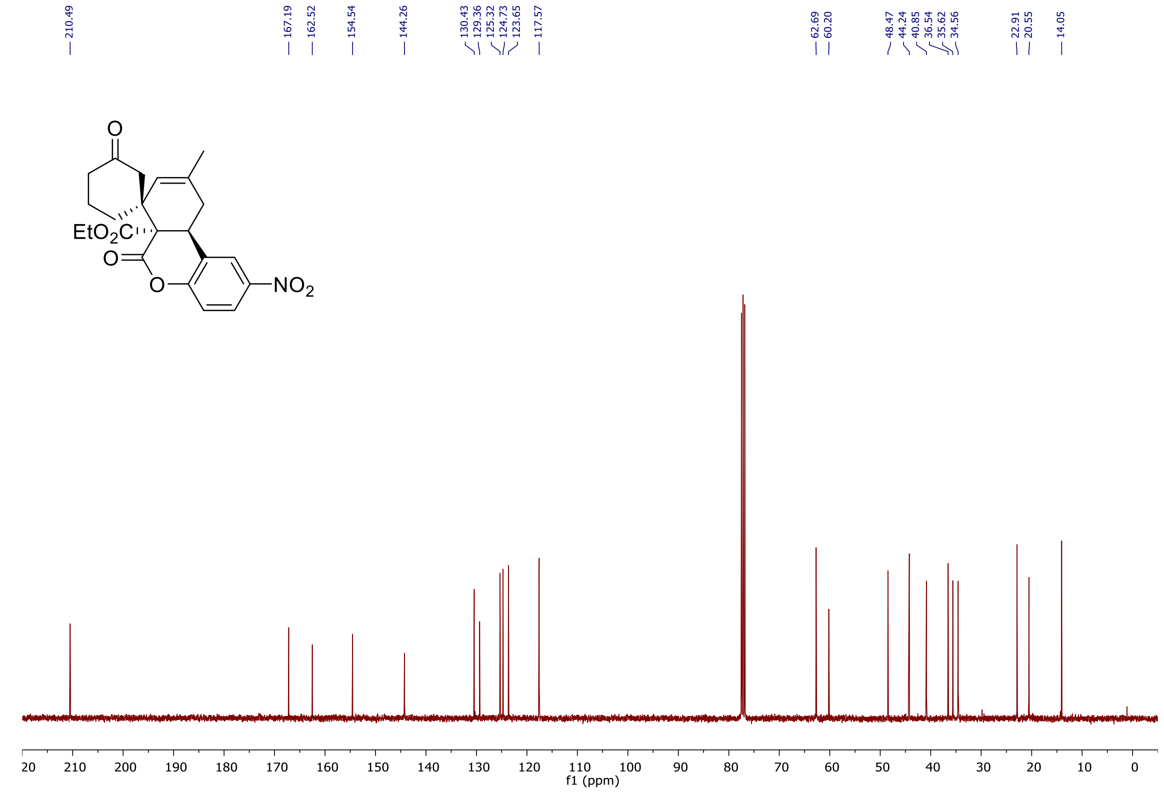

^{1}H NMR (400 MHz, $CDCl_3$) of **3o**.

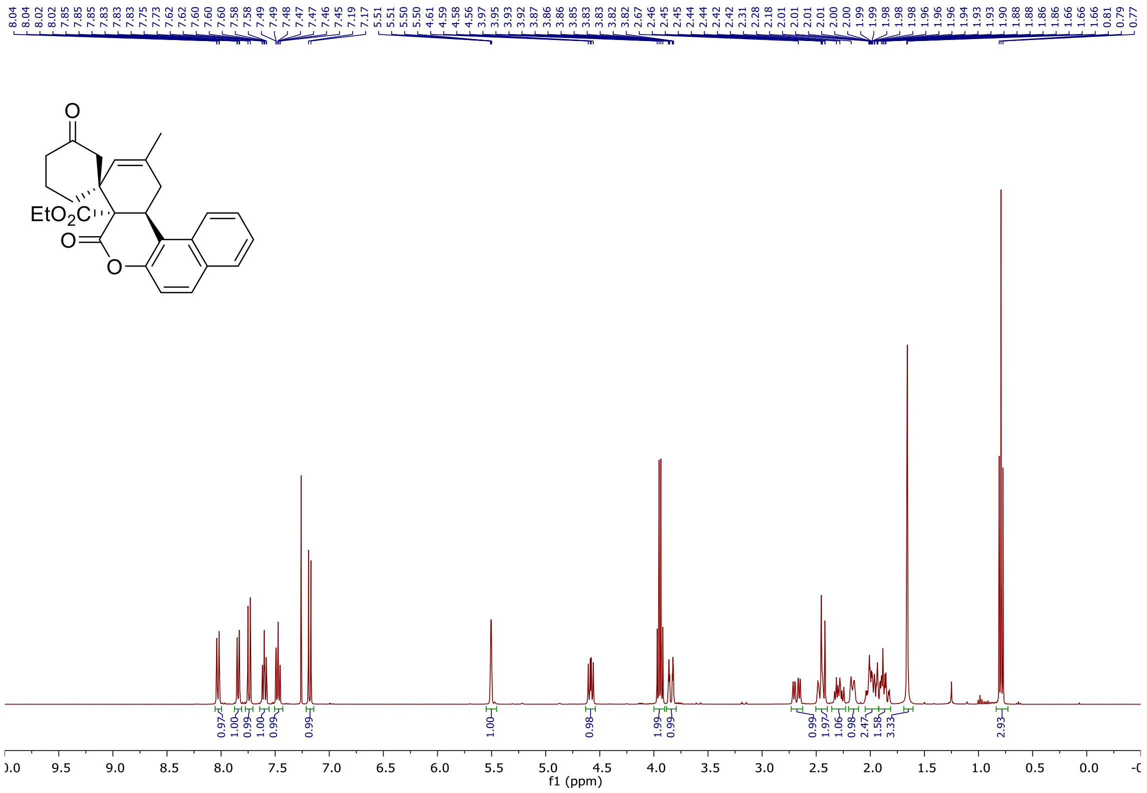

^{13}C NMR (101 MHz, $CDCl_3$) of **3o**.

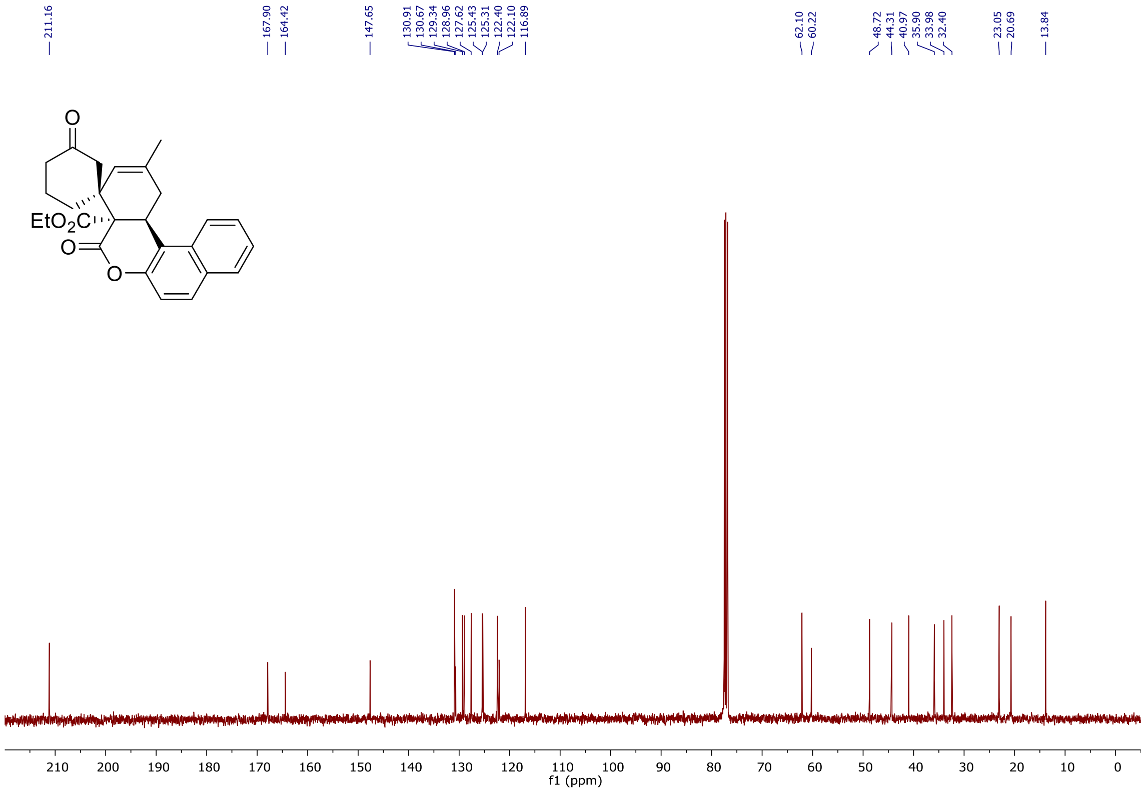

^{1}H NMR (300 MHz, $CDCl_3$) **3p**.

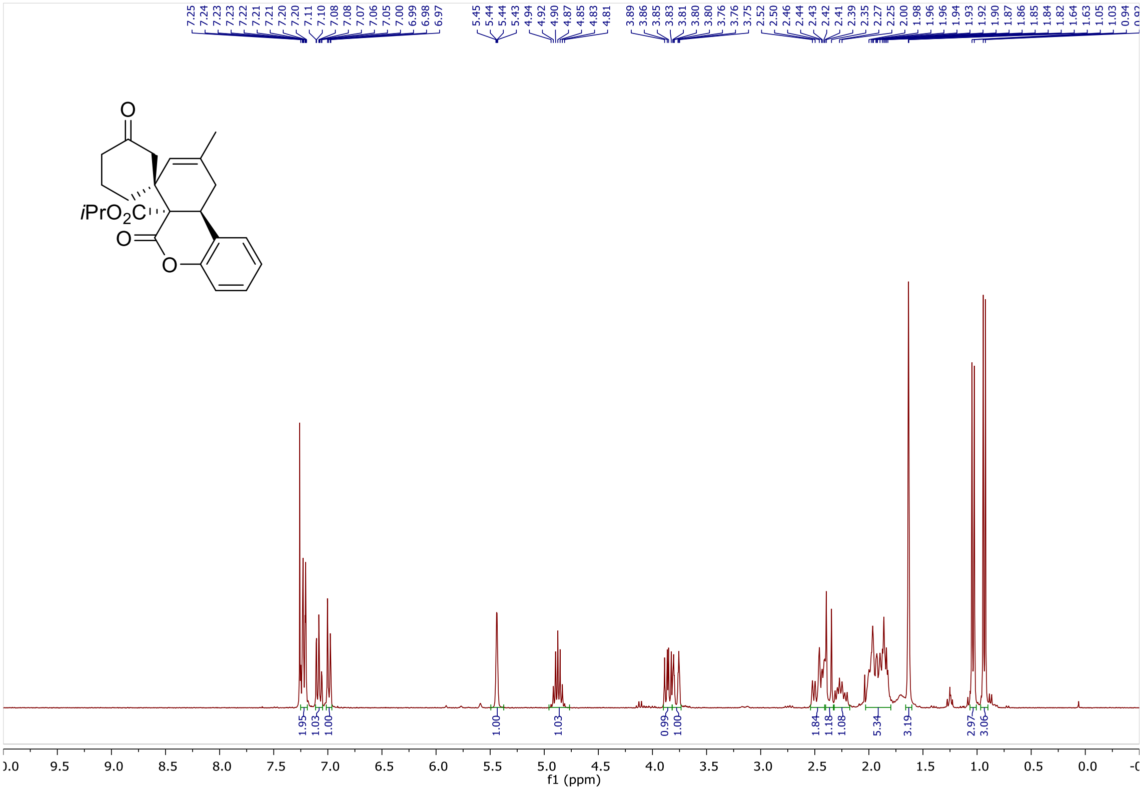

^{13}C NMR (75 MHz, $CDCl_3$) of **3p**.

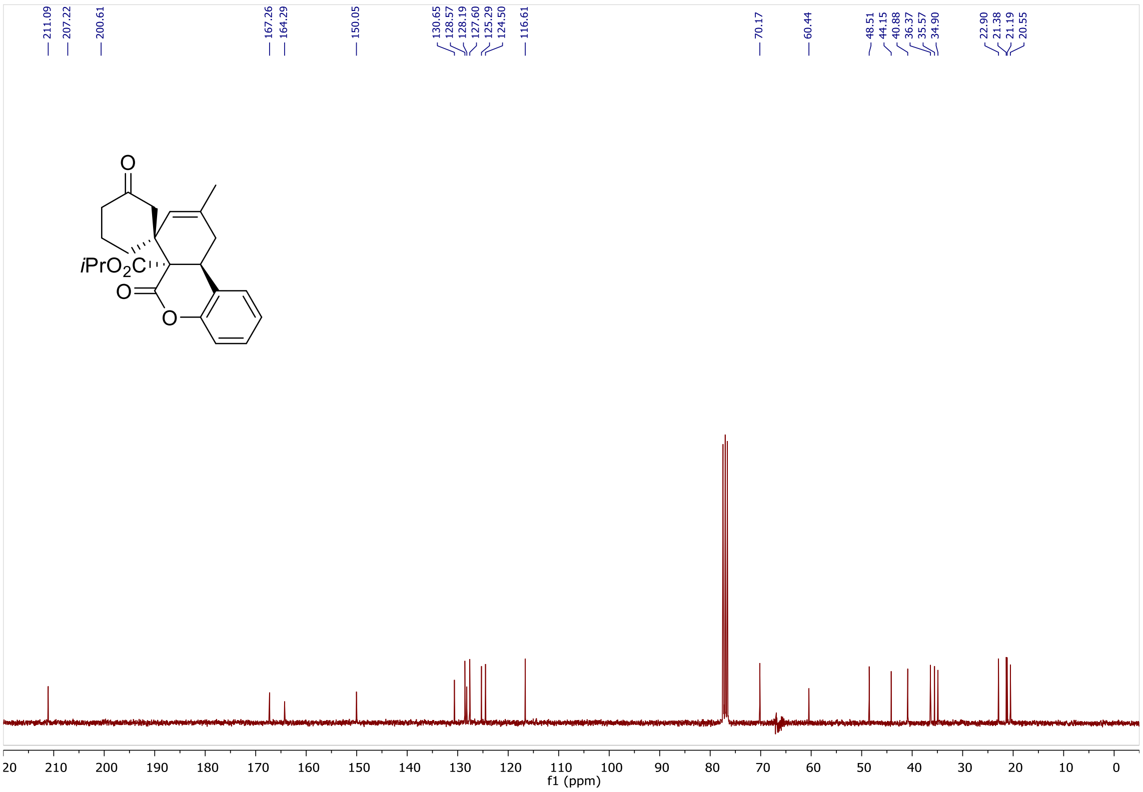

^{1}H NMR (300 MHz, $CDCl_3$) **5**.

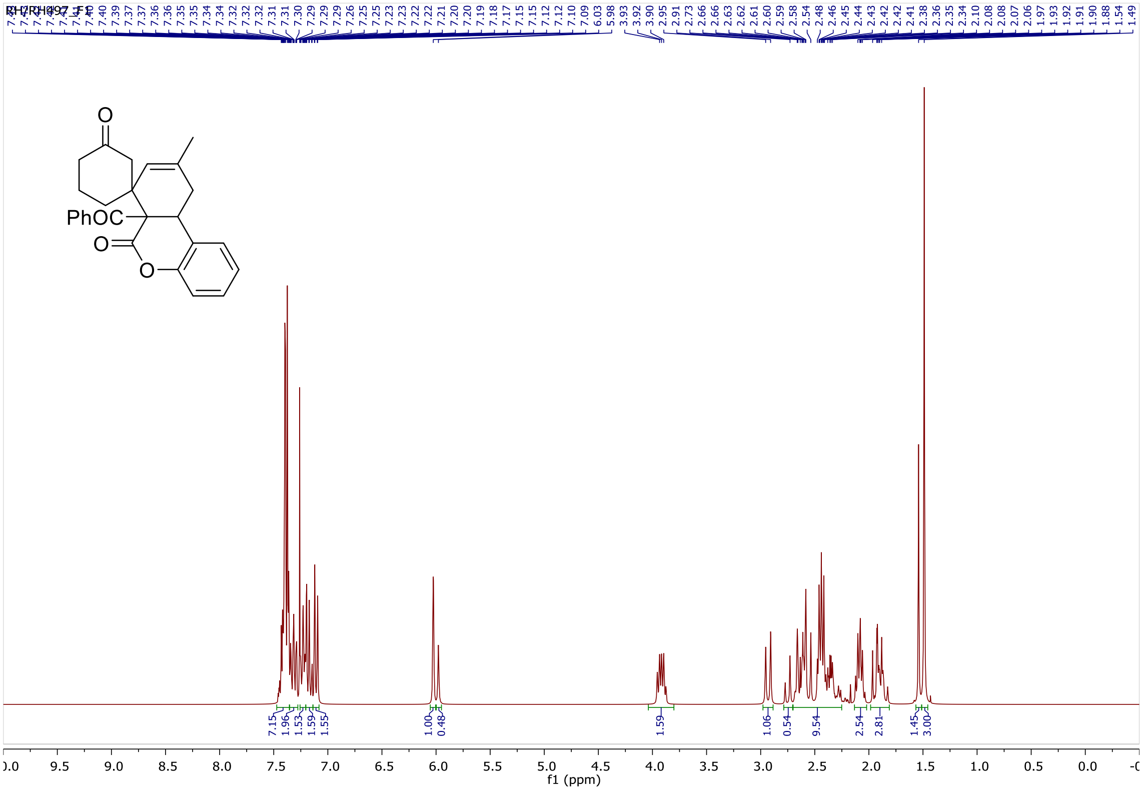

^{13}C NMR (101 MHz, $CDCl_3$) of **5**.

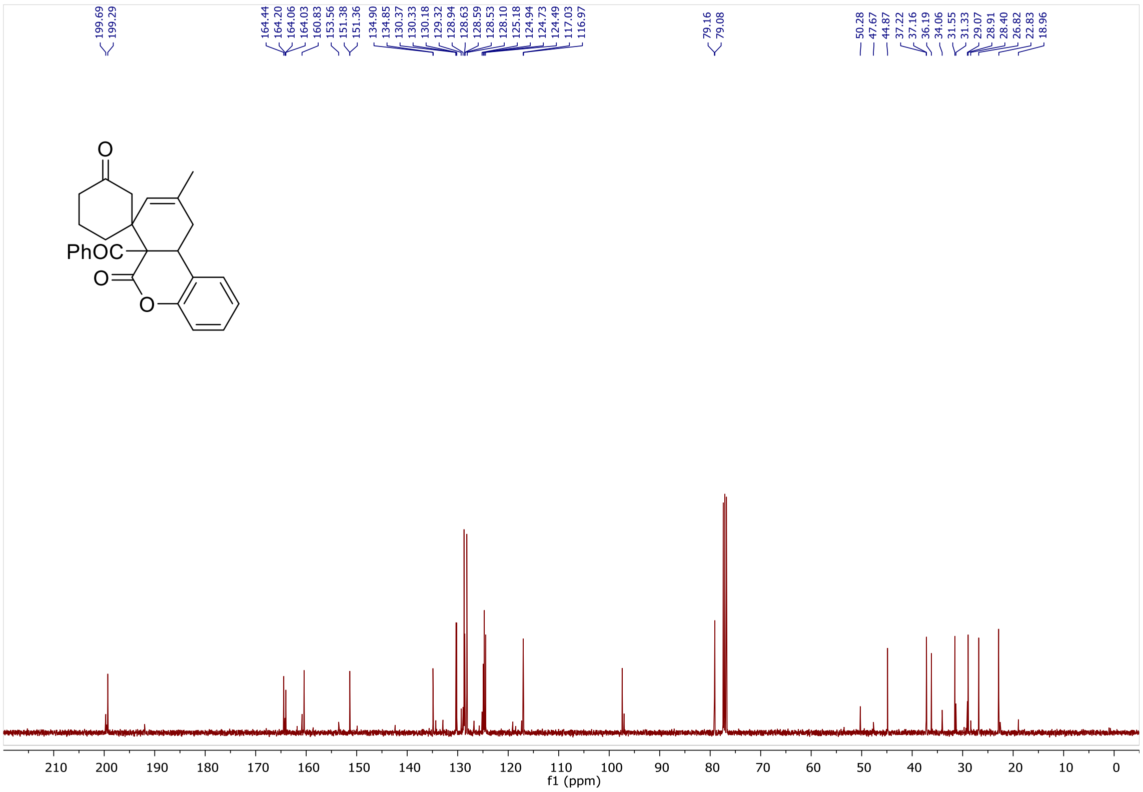

^{1}**H NMR** (400 MHz, $CDCl_3$) **7**.

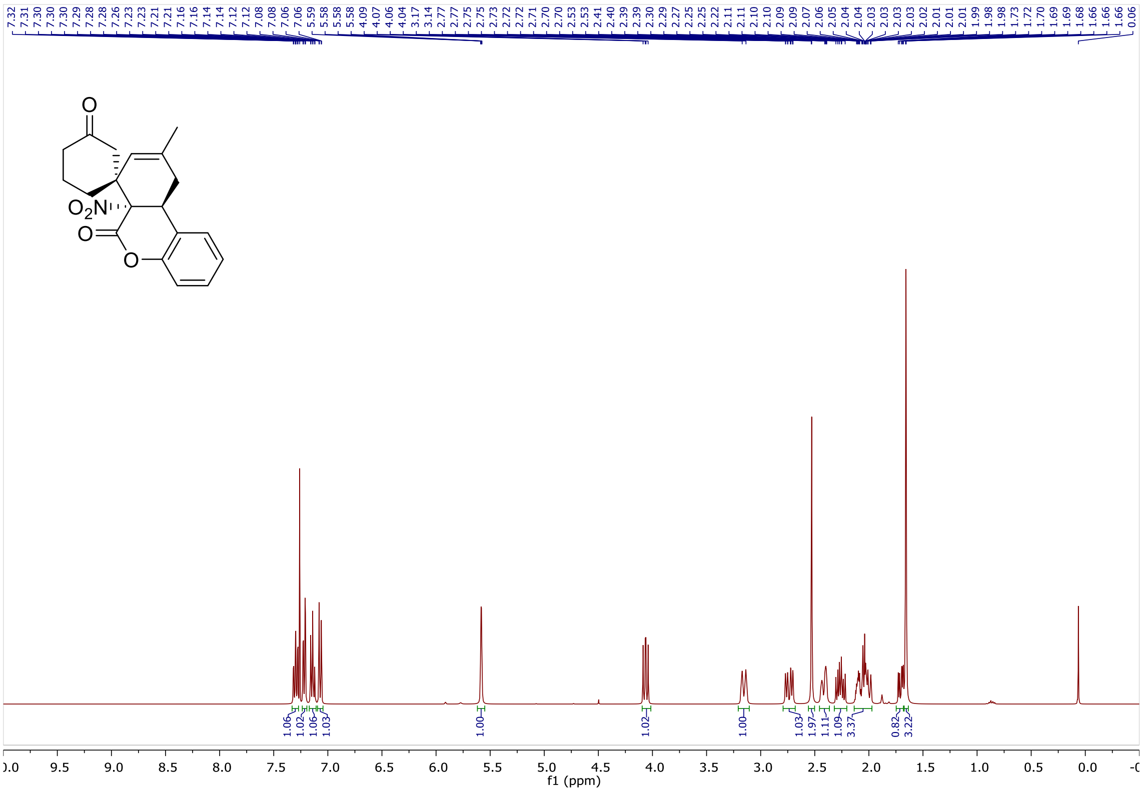

^{13}**C NMR** (101 MHz, $CDCl_3$) of **7.**

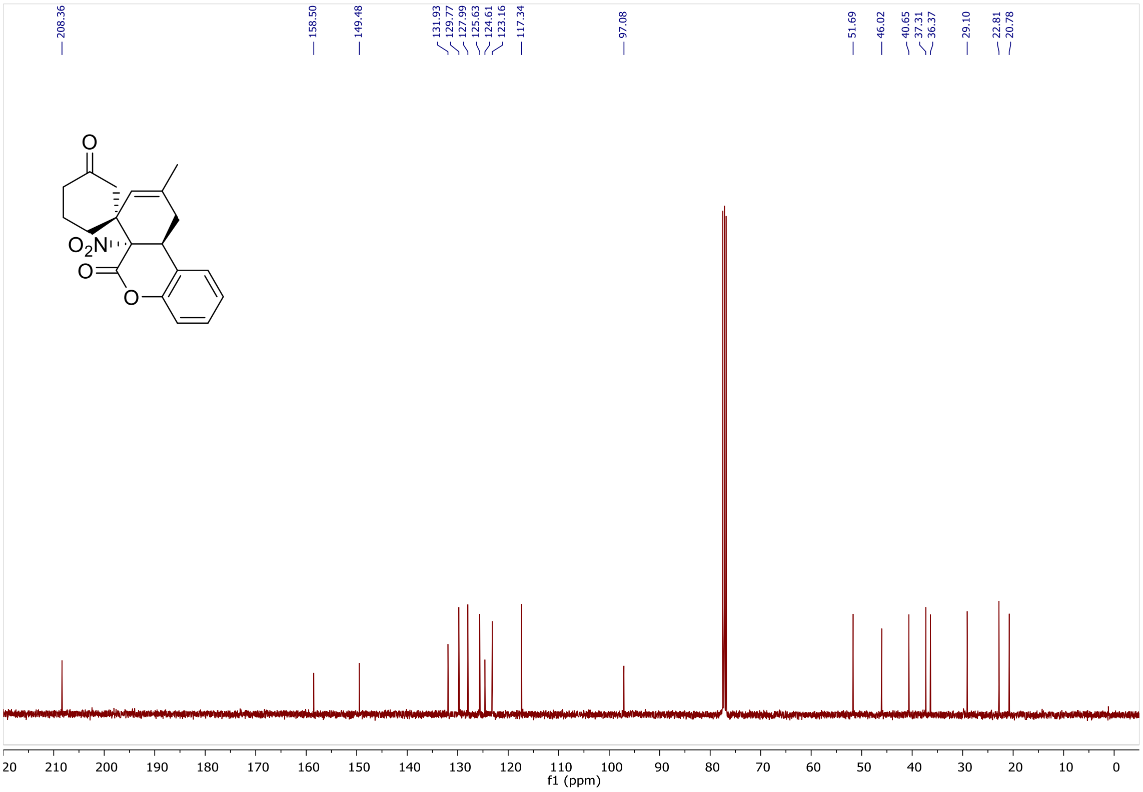

6.2 NMR Spectra of 3a'-3e' and 3h'.

^{1}H NMR (400 MHz, $CDCl_3$) **3a'**.

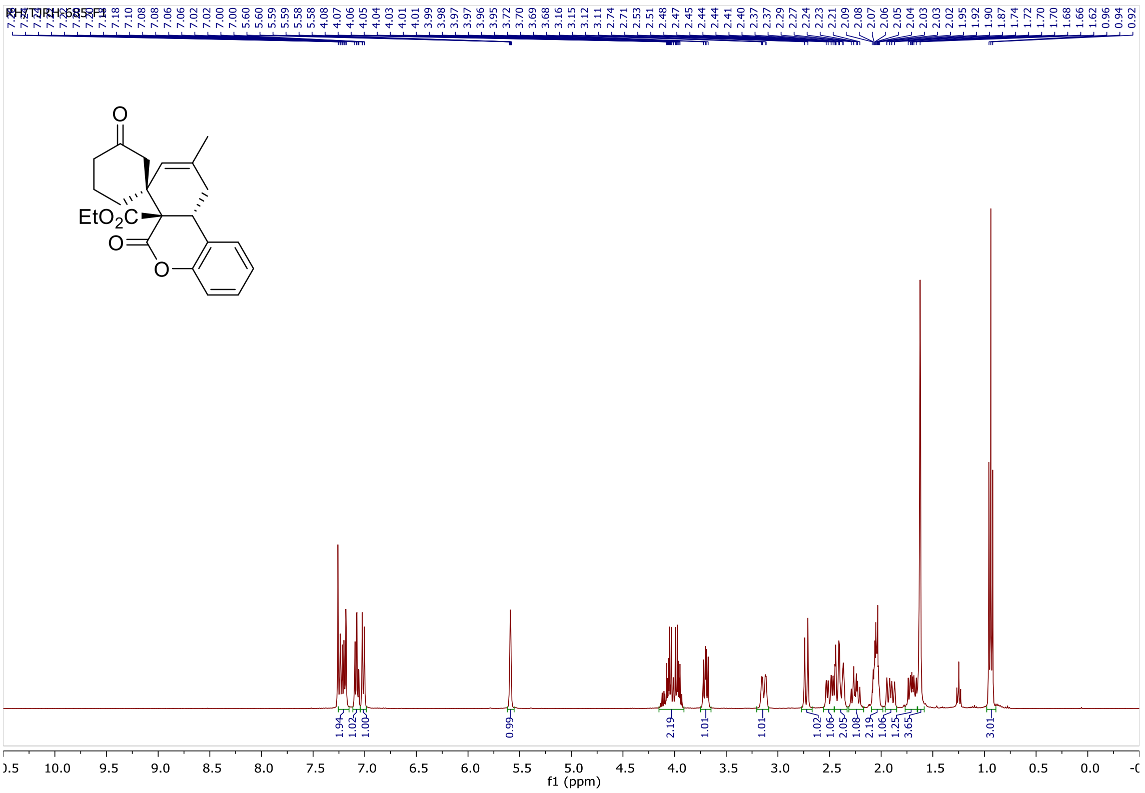

^{13}C NMR (101 MHz, $CDCl_3$) of **3a'**.

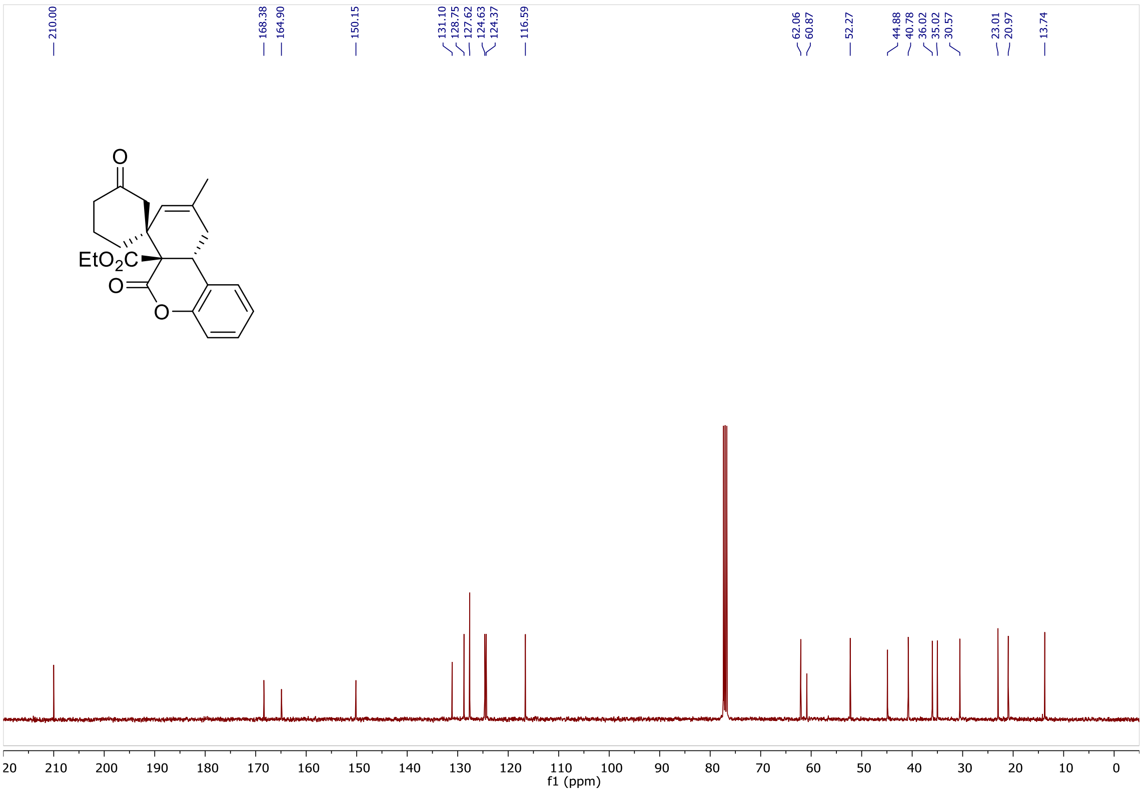

^{1}H NMR (400 MHz, $CDCl_3$) **3b'**.

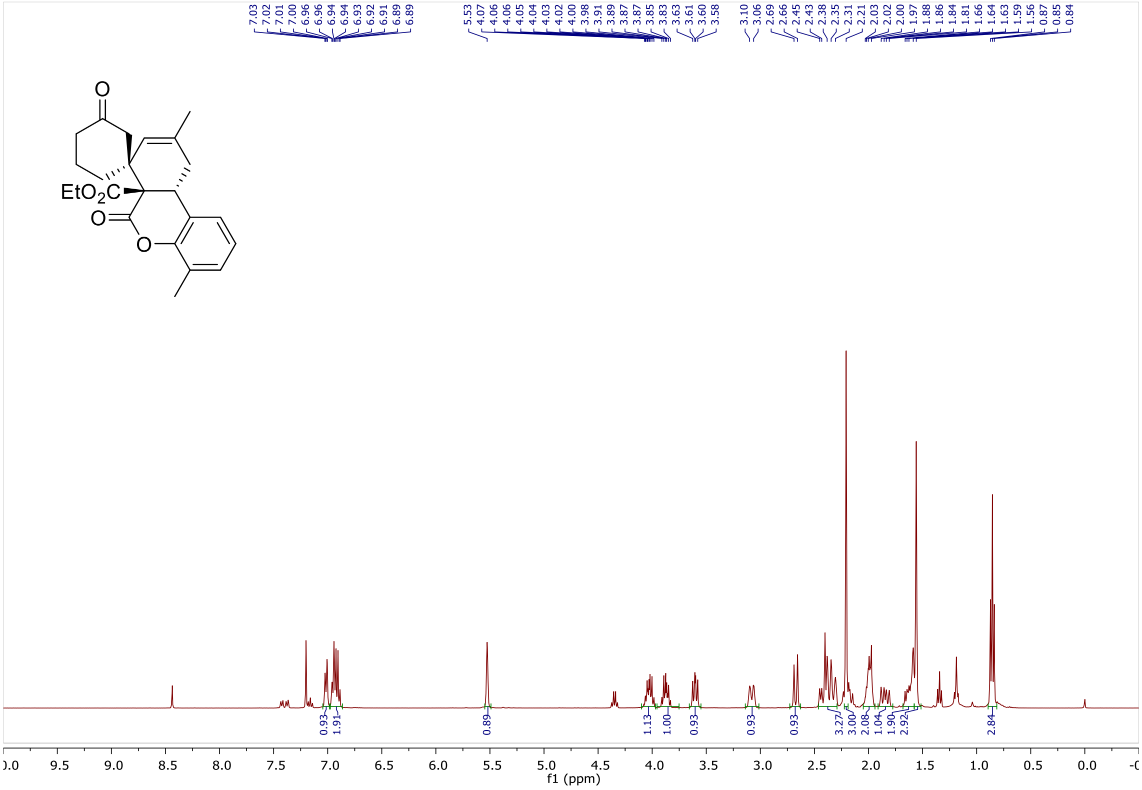

^{13}C NMR (101 MHz, $CDCl_3$) of **3b'**.

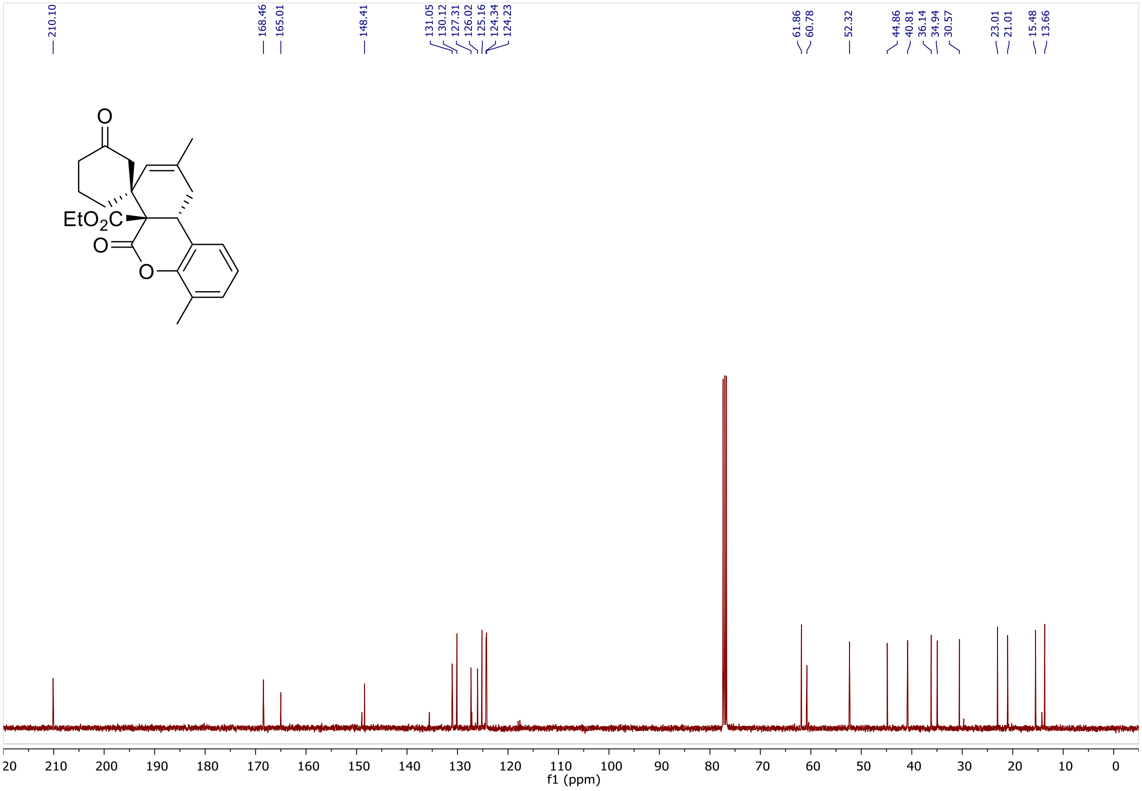

¹H NMR (400 MHz, $CDCl_3$) **3c'**.

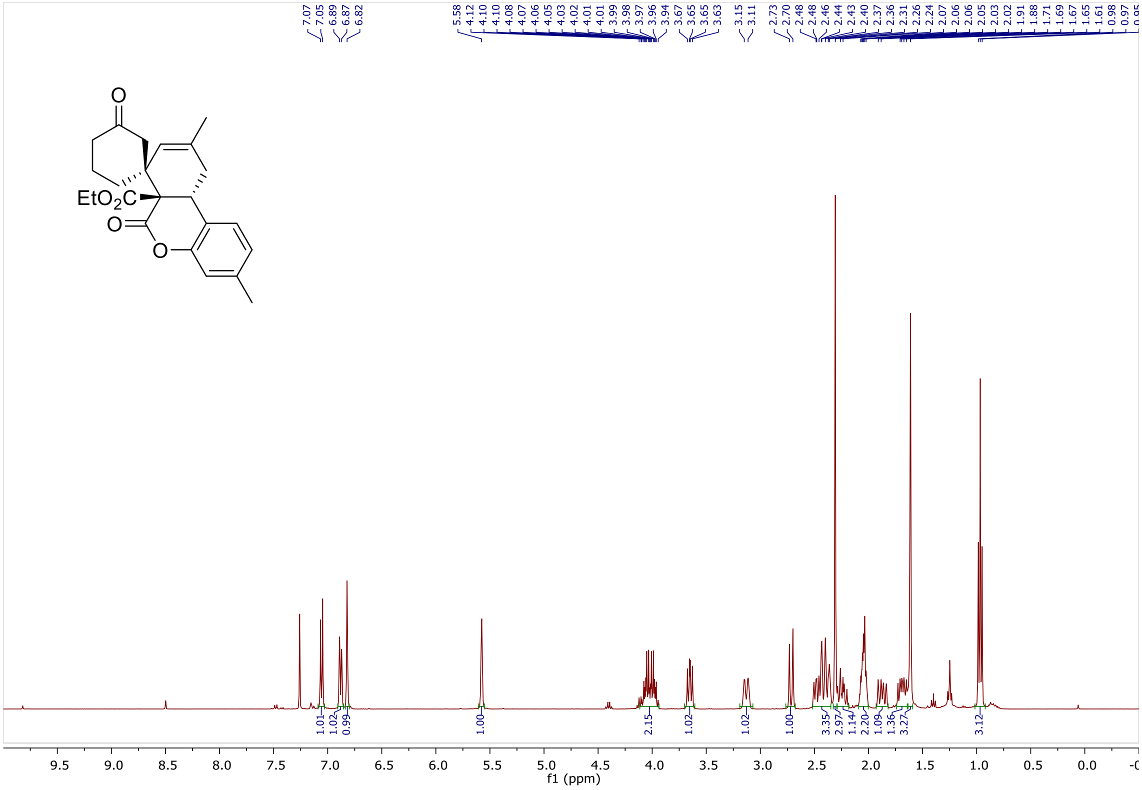

¹³C NMR (101 MHz, $CDCl_3$) of **3c'**.

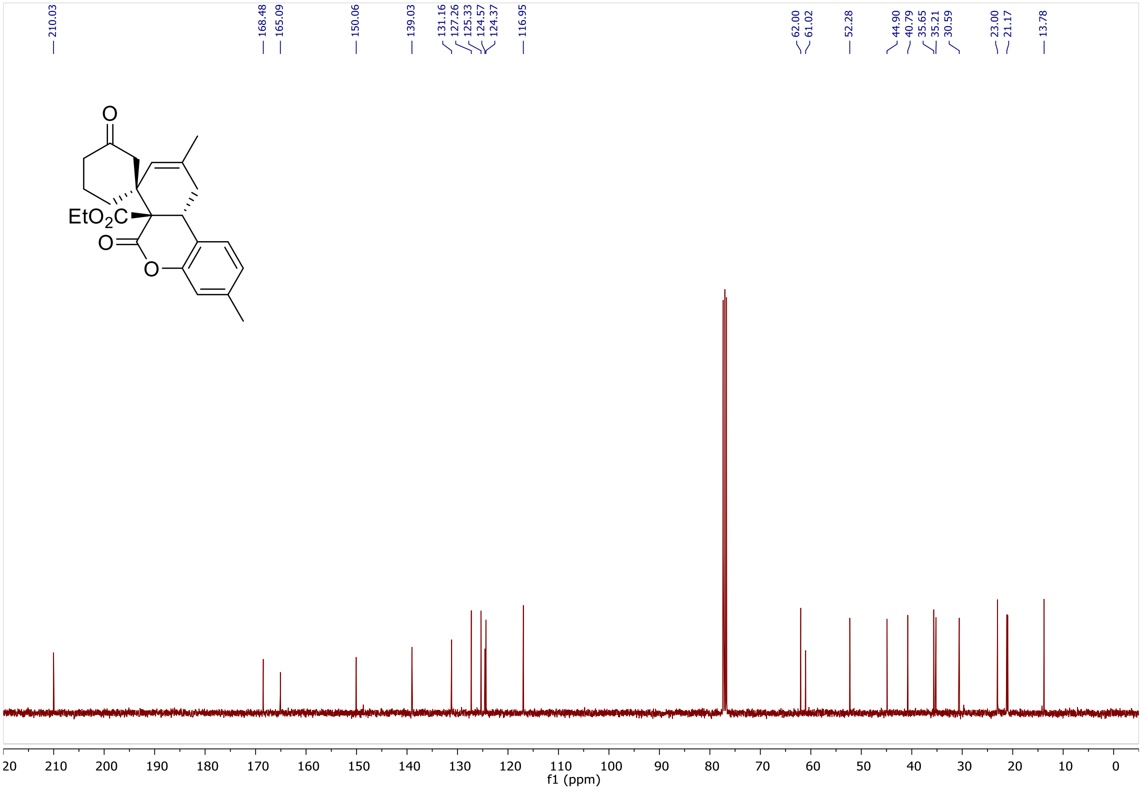

^{1}H NMR (400 MHz, $CDCl_3$) **3d'.**

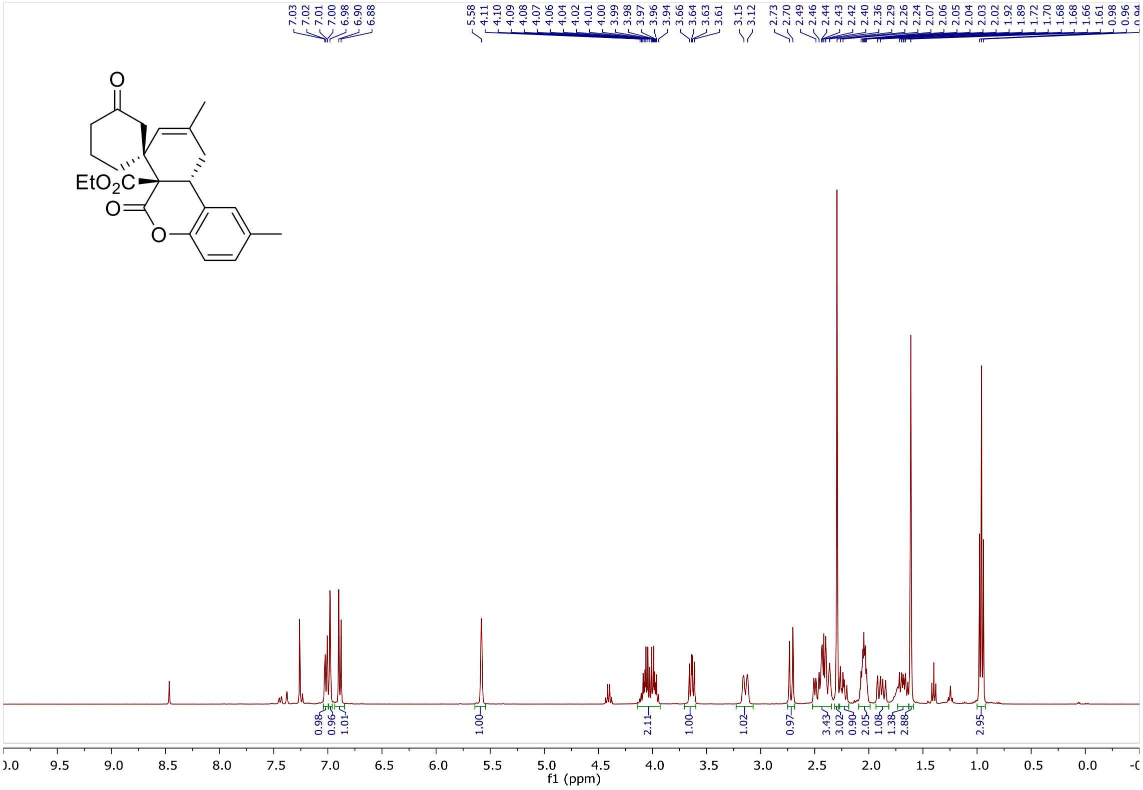

^{13}C NMR (101 MHz, $CDCl_3$) of **3d'**.

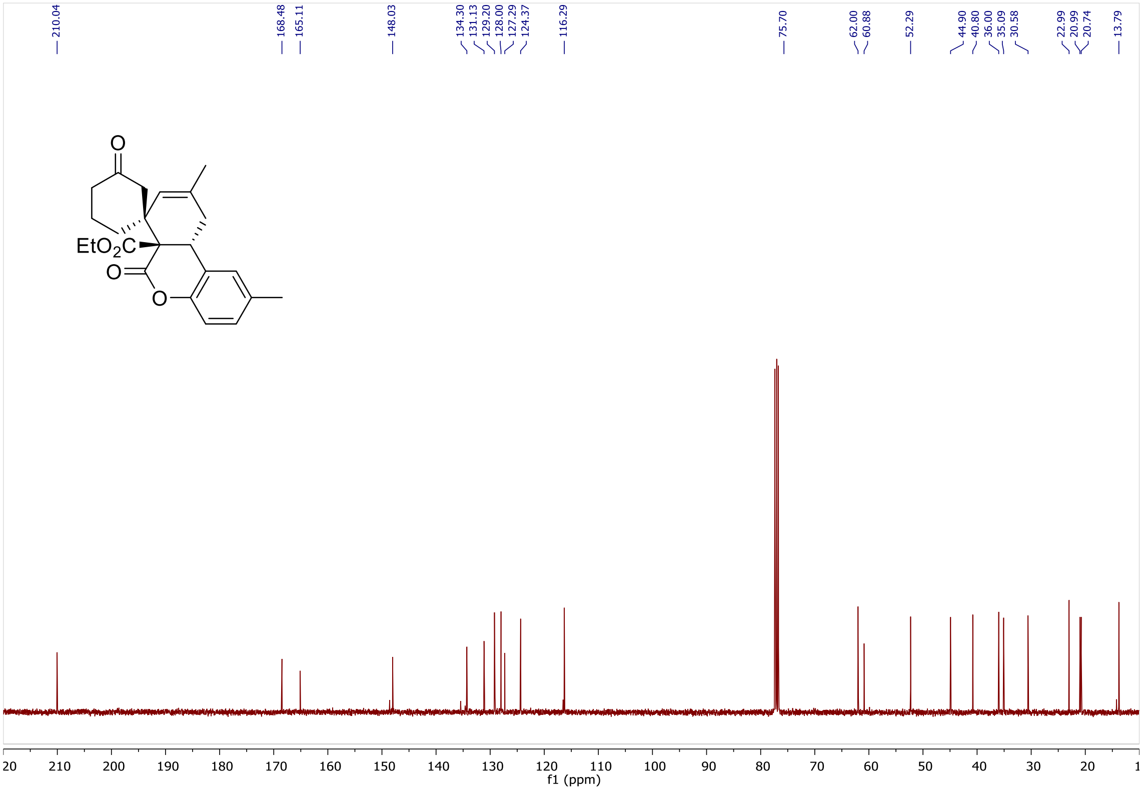

¹H NMR (400 MHz, $CDCl_3$) **3e'.**

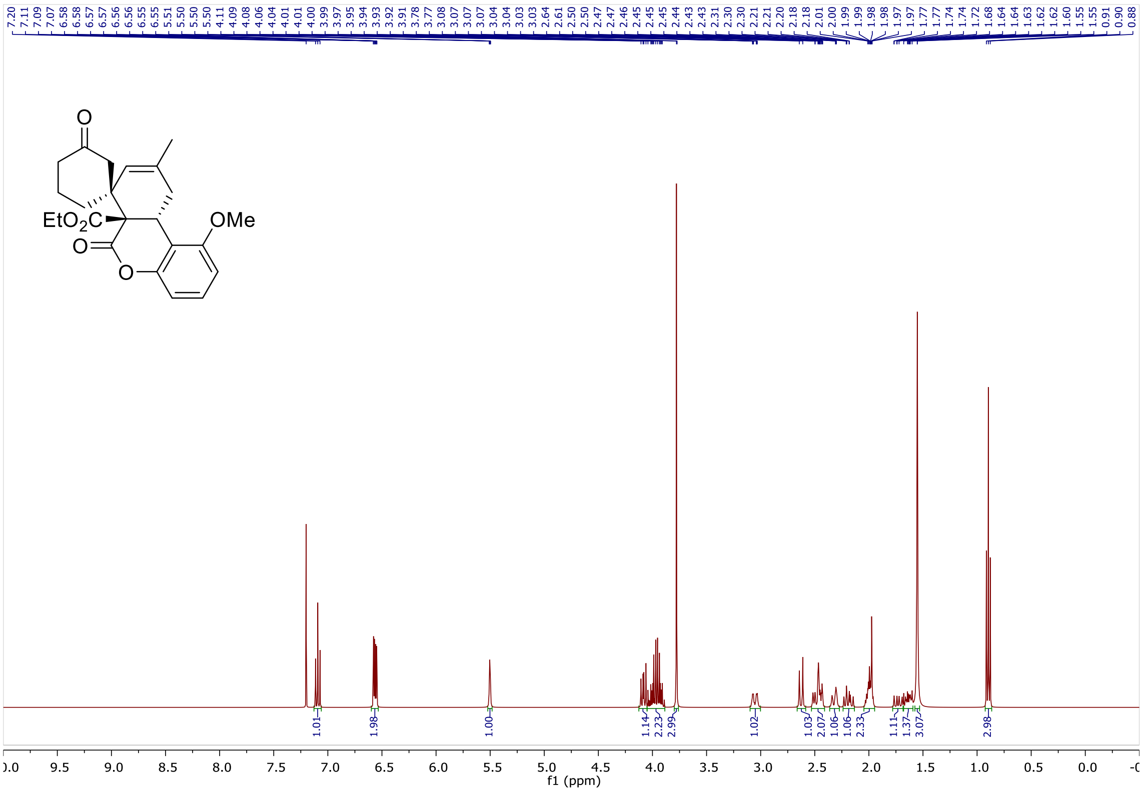

¹³C NMR (101 MHz, $CDCl_3$) of **3e'**.

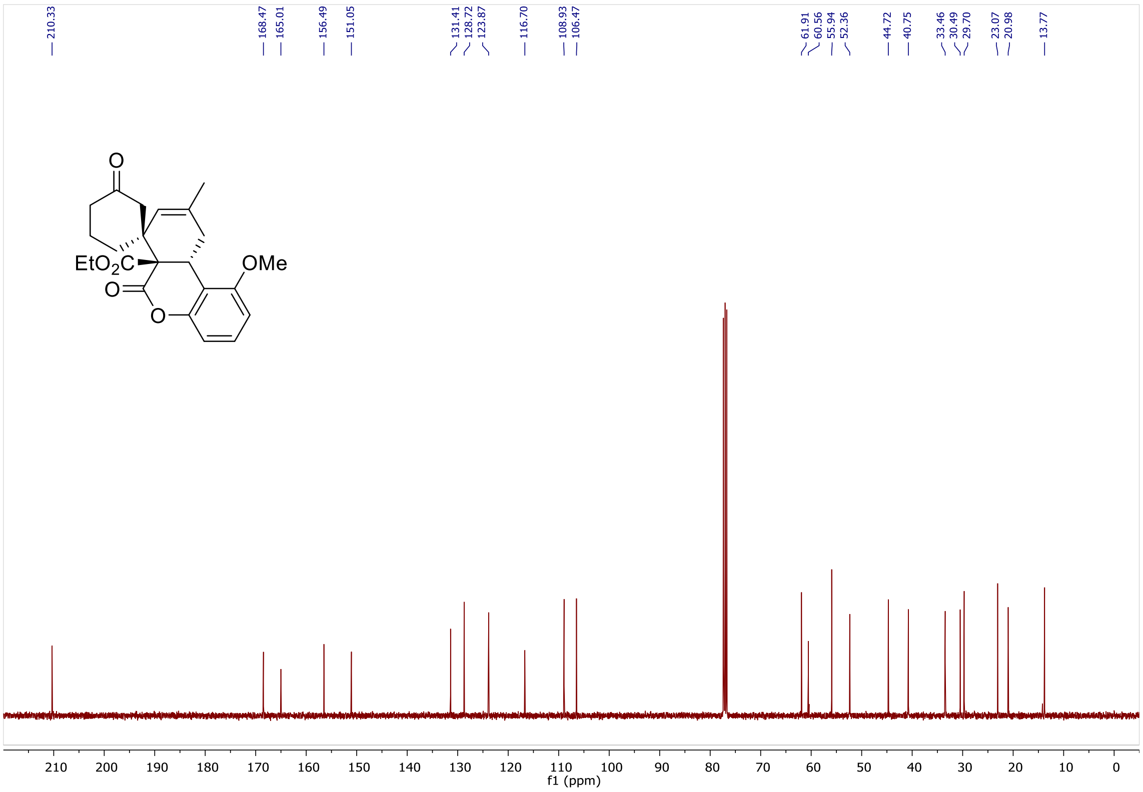

^{1}H NMR (400 MHz, $CDCl_3$) of **3h'.**

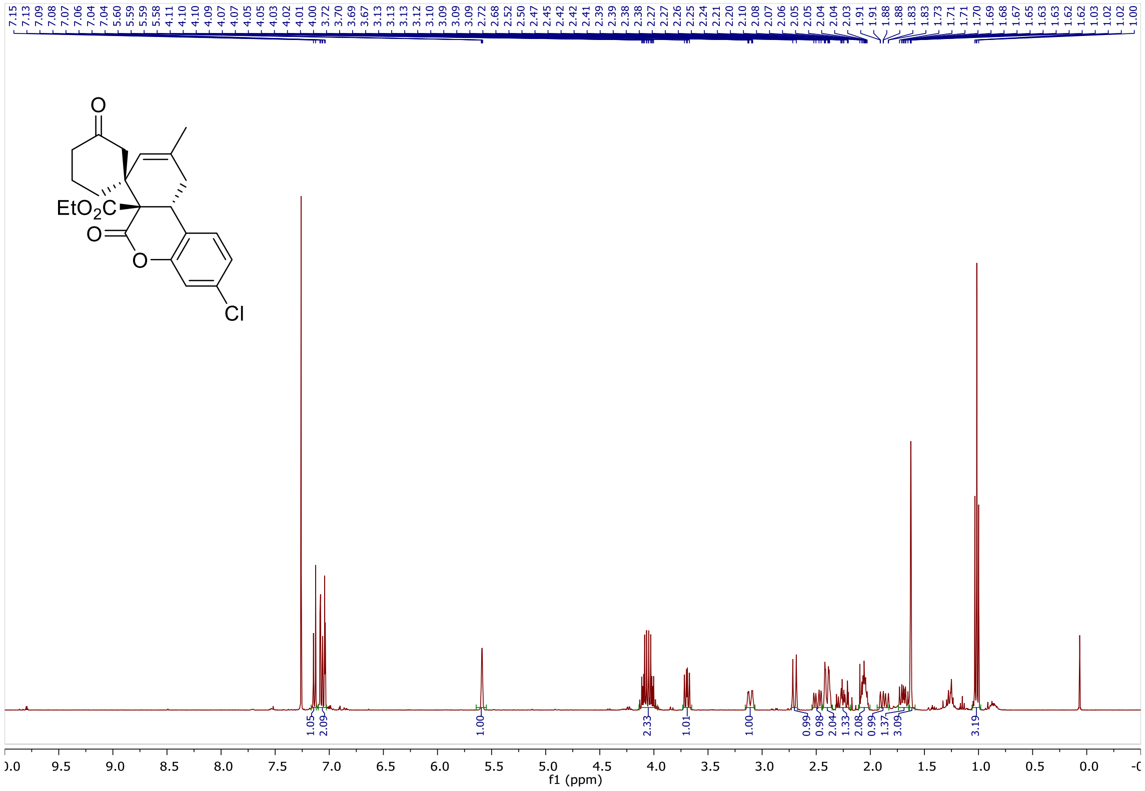

^{13}C NMR (101 MHz, $CDCl_3$) of **3h'.**

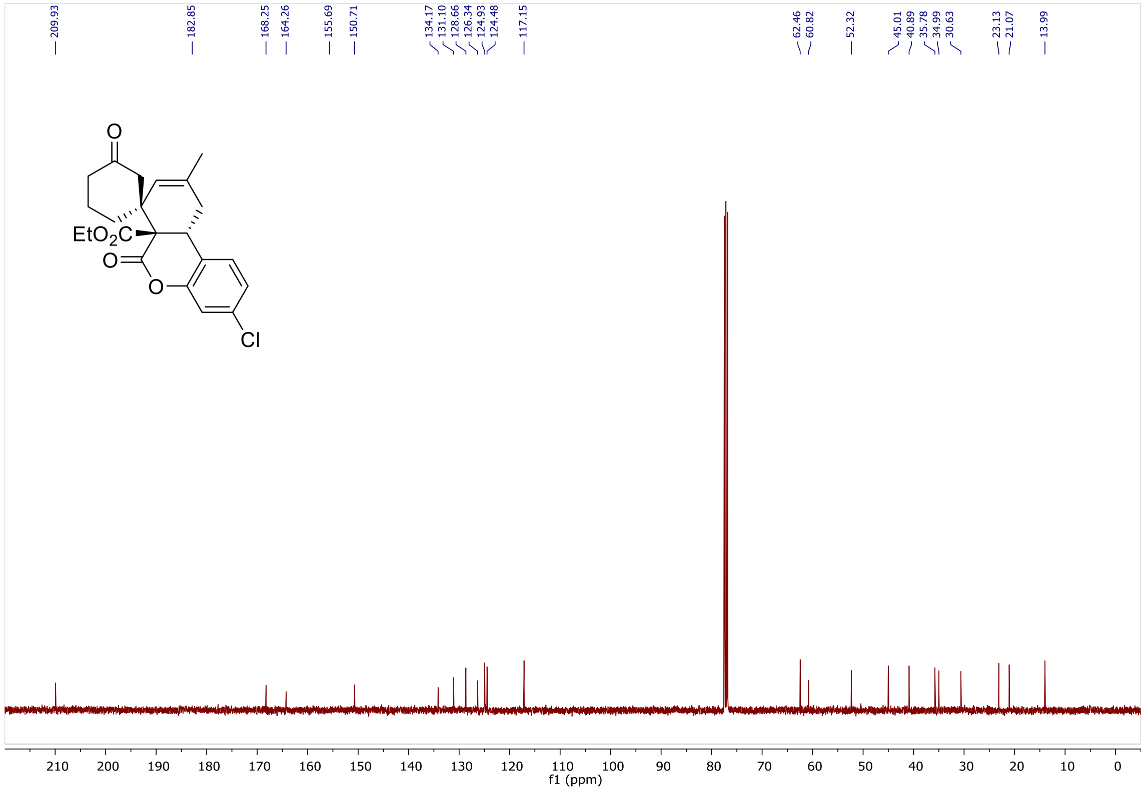

7 X-Ray Diffraction Analysis.

The samples were crystallized from mixtures of chloroform and hexane, or dichloromethane and hexane.

7.1 X-Ray Diffraction Analysis of 3a (CCDC 2395019).

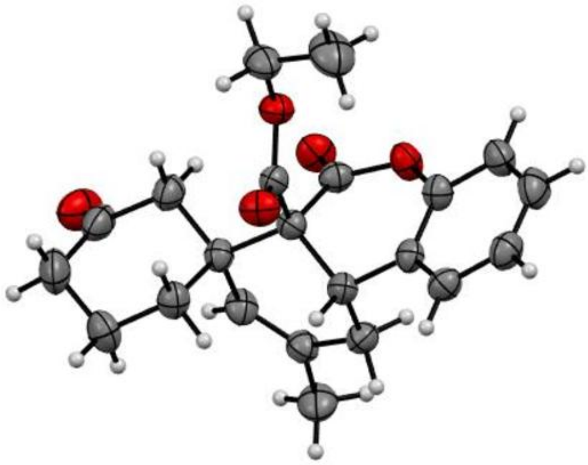

Figure S1. ORTEP diagram with thermal ellipsoids in 50% probability for **3a**.

checkCIF/PLATON report

Structure factors have been supplied for datablock(s) str2290

THIS REPORT IS FOR GUIDANCE ONLY. IF USED AS PART OF A REVIEW PROCEDURE FOR PUBLICATION, IT SHOULD NOT REPLACE THE EXPERTISE OF AN EXPERIENCED CRYSTALLOGRAPHIC REFEREE.

No syntax errors found. CIF dictionary Interpreting this report

Datablock: str2290

Bond precision:	C-C = 0.0069 A		Wavelength=1.54184
Cell:	a=8.7596(1)	b=10.2677(2)	c=21.2133(3)
	alpha=90	beta=97.623(2)	gamma=90
Temperature:	200 K		

	Calculated	Reported
Volume	1891.08(5)	1891.08(5)
Space group	P 21	P 21
Hall group	P 2yb	?
Moiety formula	C22 H24 O5	C22 H24 O5
Sum formula	C22 H24 O5	C22 H24 O5
Mr	368.41	368.41
Dx,g cm-3	1.294	1.294
Z	4	4
Mu (mm-1)	0.743	0.743
F000	784.0	784.0
F000'	786.49	
h,k,lmax	10,12,25	10,12,25
Nref	6669[3540]	6564
Tmin,Tmax	0.859,0.928	0.489,1.000
Tmin'	0.665	

Correction method= # Reported T Limits: Tmin=0.489 Tmax=1.000
AbsCorr = GAUSSIAN

Data completeness= 1.85/0.98 Theta(max)= 66.590

R(reflections)= 0.0731(6090) wR2(reflections)= 0.1944(6564)

S = 1.023 Npar= 486

7.2 X-Ray Diffraction Analysis of 3h' (CCDC 2395020).

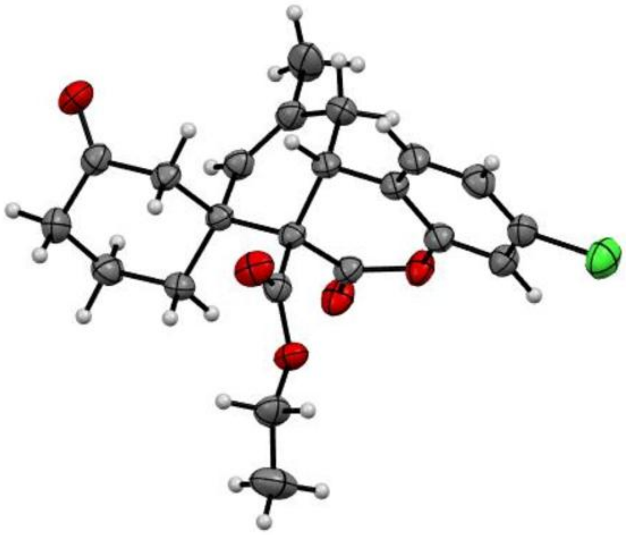

Figure S2. ORTEP diagram with thermal ellipsoids in 50% probability for **3h'**.

checkCIF/PLATON report

Structure factors have been supplied for datablock(s) str2299

THIS REPORT IS FOR GUIDANCE ONLY. IF USED AS PART OF A REVIEW PROCEDURE FOR PUBLICATION, IT SHOULD NOT REPLACE THE EXPERTISE OF AN EXPERIENCED CRYSTALLOGRAPHIC REFEREE.

No syntax errors found. CIF dictionary Interpreting this report

Datablock: str2299

```
Bond precision:   C-C = 0.0025 A          Wavelength=1.54184

Cell:             a=10.1533(1)     b=17.6329(2)       c=11.1054(1)
                  alpha=90         beta=96.280(1)     gamma=90
Temperature:      200 K

                 Calculated                  Reported
Volume           1976.29(3)                  1976.29(3)
Space group      P 21                        P 21
Hall group       P 2yb                       ?
Moiety formula   C22 H23 Cl O5               C22 H23 Cl O5
Sum formula      C22 H23 Cl O5               C44 H46 Cl2 O10
Mr               402.85                      805.71
Dx,g cm-3        1.354                       1.354
Z                4                           2
Mu (mm-1)        1.974                       1.974
F000             848.0                       848.0
F000'            851.96
h,k,lmax         12,21,13                    12,21,13
Nref             7746[ 4007]                 7633
Tmin,Tmax        0.676,0.811                 0.847,1.000
Tmin'            0.514

Correction method= # Reported T Limits: Tmin=0.847 Tmax=1.000
AbsCorr = MULTI-SCAN

Data completeness= 1.90/0.99       Theta(max)= 71.750

R(reflections)= 0.0308( 7368)                       wR2(reflections)=
                                                     0.0823( 7633)
S = 1.024                Npar= 509
```

8 Electronic Circular Dichroism of 7.

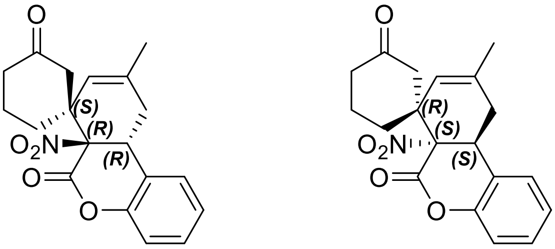

Enantiomer 1 **Enantiomer 2**

Figure S3. Two enantiomeric forms possible of compound **7**.

The ECD spectrum prediction was performed using TD-DFT theory with the ORCA 5.0.2 software, employing the double-hybrid B2PLYP functional and the Def2-QZVP basis set. The first 20 excitations were calculated. Solvent effects (acetonitrile) were considered in the calculations. The Tamm-Dancoff approximation was disabled, as it can lead to erroneous signals in the calculation of the circular dichroism spectrum. The ECD spectra of each conformer were calculated, and the spectra were weighted based on their previously calculated relative populations. Consistency and similarity were observed in the position, shape, and amplitude of the dichroism bands for all conformers, consistent with the high structural rigidity of the molecule found in the prior conformational study.

The circular dichroism spectrum was obtained using a Jasco J-810 instrument. The analysis range covered 200 to 400 nm. A blank correction was performed using pure solvent (acetonitrile). The final spectrum of the sample was recorded at a concentration of 0.0001 M, with three cumulative scans conducted on the same sample.

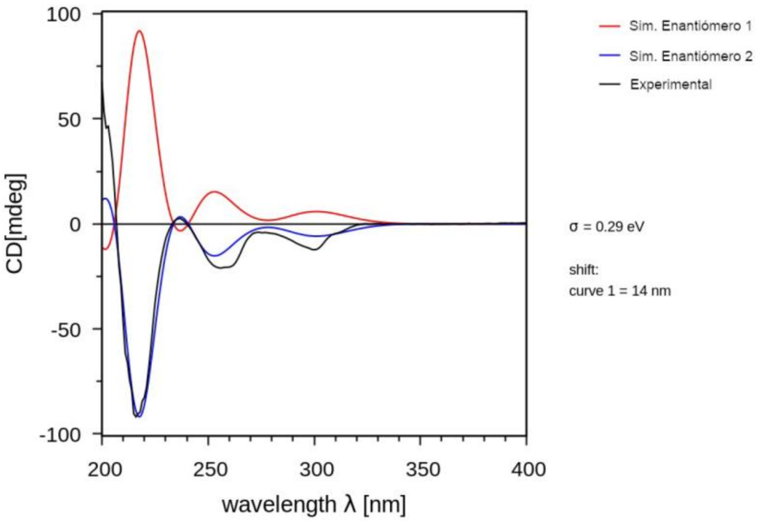

Figure S4. ECD graph of **7**.

Similarity factor Enantiomer 1 = 0.0003

Similarity factor Enantiomer 2 = 0.9823

Delta value: 0.9820

After comparing the values given for the two possible enantiomers of **7**, we can conclude that the absolute configuration is the one given by enantiomer 2.

9.1 Computational details

All the stationary points were fully optimized using the three-parameter hybrid functional B3LYP[14] using the 6-31G(d) basis set[15] and the Grimme D3 empirical dispersion correction with the Becke–Jones damping potential (D3BJ)[16]. Solvent effects were tackled by means of the Polarization Continuum Model[17] (PCM) using toluene (ε = 2.37) as implicit model solvents.

The optimized stationary points were fully characterized by harmonic analysis.[18] All reactants, products and intermediates had positive definite Hessians (no imaginary frequencies, NIMAG=0). Transition structures showed one and only one imaginary frequency (NIMAG=1) associated with nuclear motion along the reaction coordinate under study. All the calculations were carried out with the Gaussian 16 suite of programs.[19]

3a:**3a'** selectivities have been computed by using the Eyring–Polanyi equation[20] from the previously computed Gibbs free activation energies (ΔG^a) associated with the least energetic transition steps related to the initial C–C bond formation, by using equation(e-SI1) and imposing the normalization conditions of equation(e-SI2):

$$\frac{[3a]}{[3a']} = e^{-\Delta\Delta G/RT} \qquad \text{e-SI3}$$

$$[\boldsymbol{3a}] + [\boldsymbol{3a'}] = 100 \qquad \text{e-SI3}$$

where $\Delta\Delta G^a$ is the difference between the activation Gibbs free energies and R is the gas constant.

[14] C. Lee, W. Yang, R. G. Parr, *Phys. Rev. B: Condens. Matter Mater. Phys.* **1988**, *37*, 785–789.
[15] B. P. Pritchard, D. Altarawy, B. Didier, T. D. Gibson, T. L. Windus, *J. Chem. Inf. Model.* 2019, 59, 11, 4814–4820.
[16] S. Grimme,S. Ehrlich, L Goerigk, *J. Comp. Chem. Phys.* **2011**, *32*, 1556–1465.
[17] a) R. Cammi, J. Tomasi, *J. Comput. Chem.* **1995**, *16*, 1449–1458; b) J. Tomasi, B. Mennucci, R. Cammi, *Chem. Rev.* **2005**, *105*, 2999–3094.
[18] J. McIver Jr., A. Komornicki, *J. Am. Chem. Soc.* **1972**, *94*, 2625-2633.
[19] Gaussian 16, Revision C.01, Frisch, M. J et al. Gaussian, Inc., Wallingford CT, 2016.
[20] H. Eyring, M. Polanyi, *Z. Phys. Chem. B* **1931**, *12*, 279–311.

Theoretical TOF values were computed by means of equation e-SI3[21]

$$TOF = \frac{k_B T}{h} e^{-\delta G/RT} \qquad \text{e-SI3}$$

where k_B, R an h corresponds to the Boltzmann, ideal gas and Plank constants; T corresponds to the temperature of the system and δG corresponds to the effective "activation" energy of the catalytic cycle, which refers to the energy spam between the most energetic transition structure and the less energetic intermediate of the catalytic cycle. Noteworthy, this latter energetic value is not necessarily determined by the rate-determining step.[20b]

[21] a) S. Kozuch, S. Shaik, *J. Am. Chem. Soc.* **2006**, *128*, 3355–3365.;b) S. Kozuch, S. Shaik, *Acc. Chem. Res.* **2011**, *42*, 101–110.

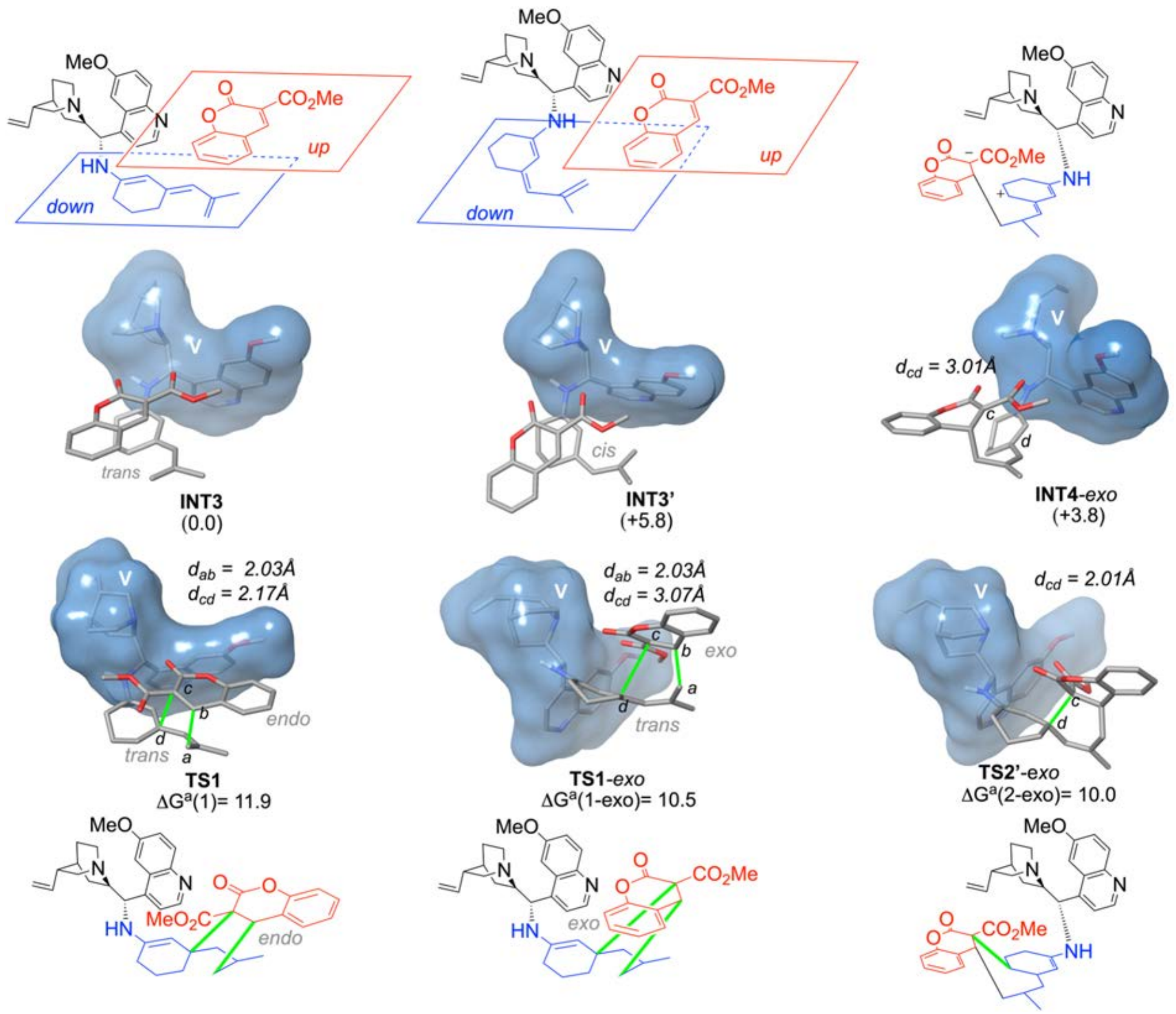

Figure S5. Main geometrical features and schematic representation of the stationary points associated with the formation of spiro [4+2] cycloadducts 3a and 3a' from ketone 1a and alkene 2a catalyzed by V computed at B3LYP-D3BJ(SCRF=PCM,toluene)/6-31G(d) level of theory. Relative Gibbs energies and distances are in kcal mol^{-1} and Å, respectively.

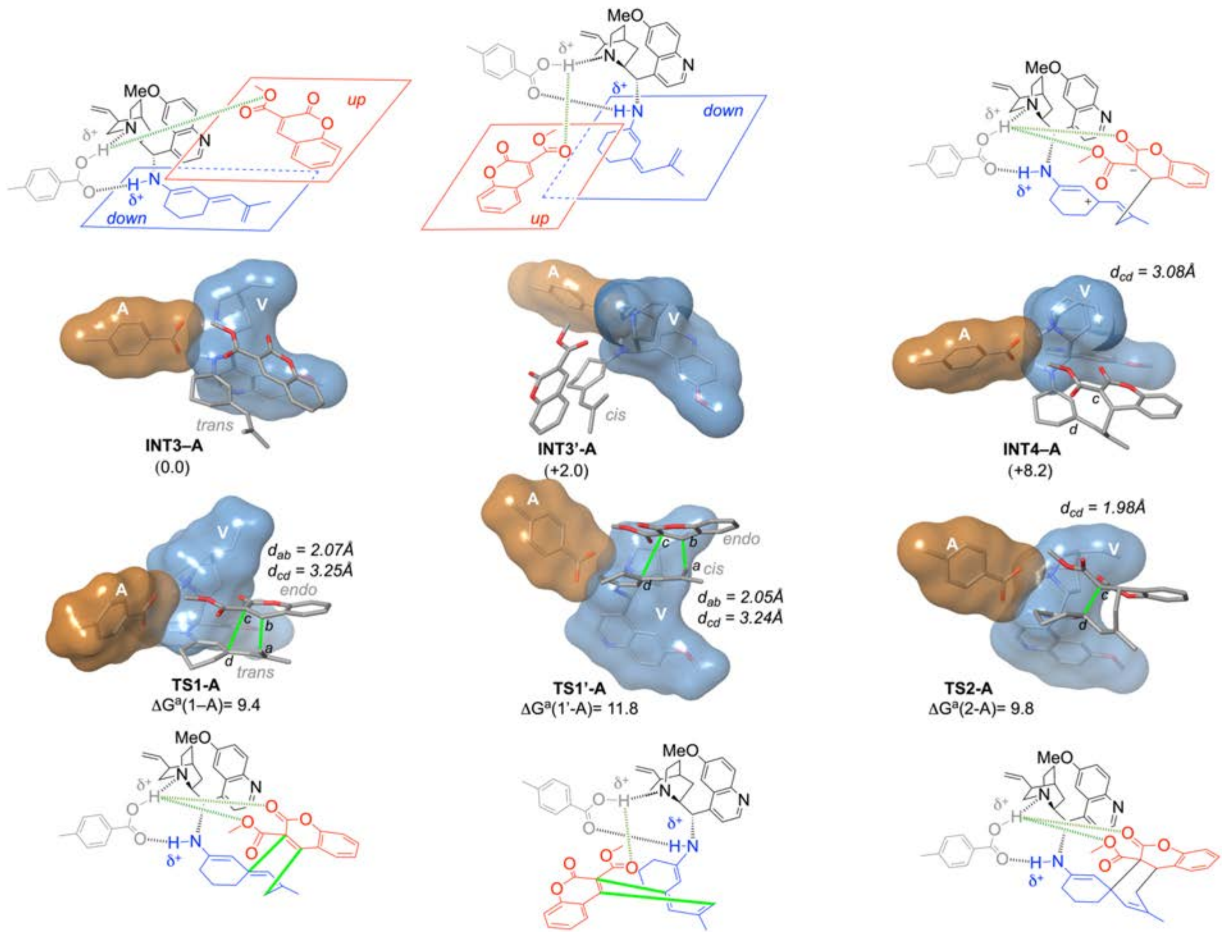

Figure S6. Main geometrical features and schematic representation of the stationary points associated with the formation of spiro [4+2] cycloadducts **3a** and **3a'** from ketone **1a** and alkene **2a** catalyzed by **V** in the presence of acidic additive (p-methyl benzoic acid, denoted as **A**) computed at B3LYP-D3BJ(SCRF=PCM,toluene)/6-31G(d) level of theory. Relative Gibbs energies and distances are in kcal mol^{-1} and Å, respectively.

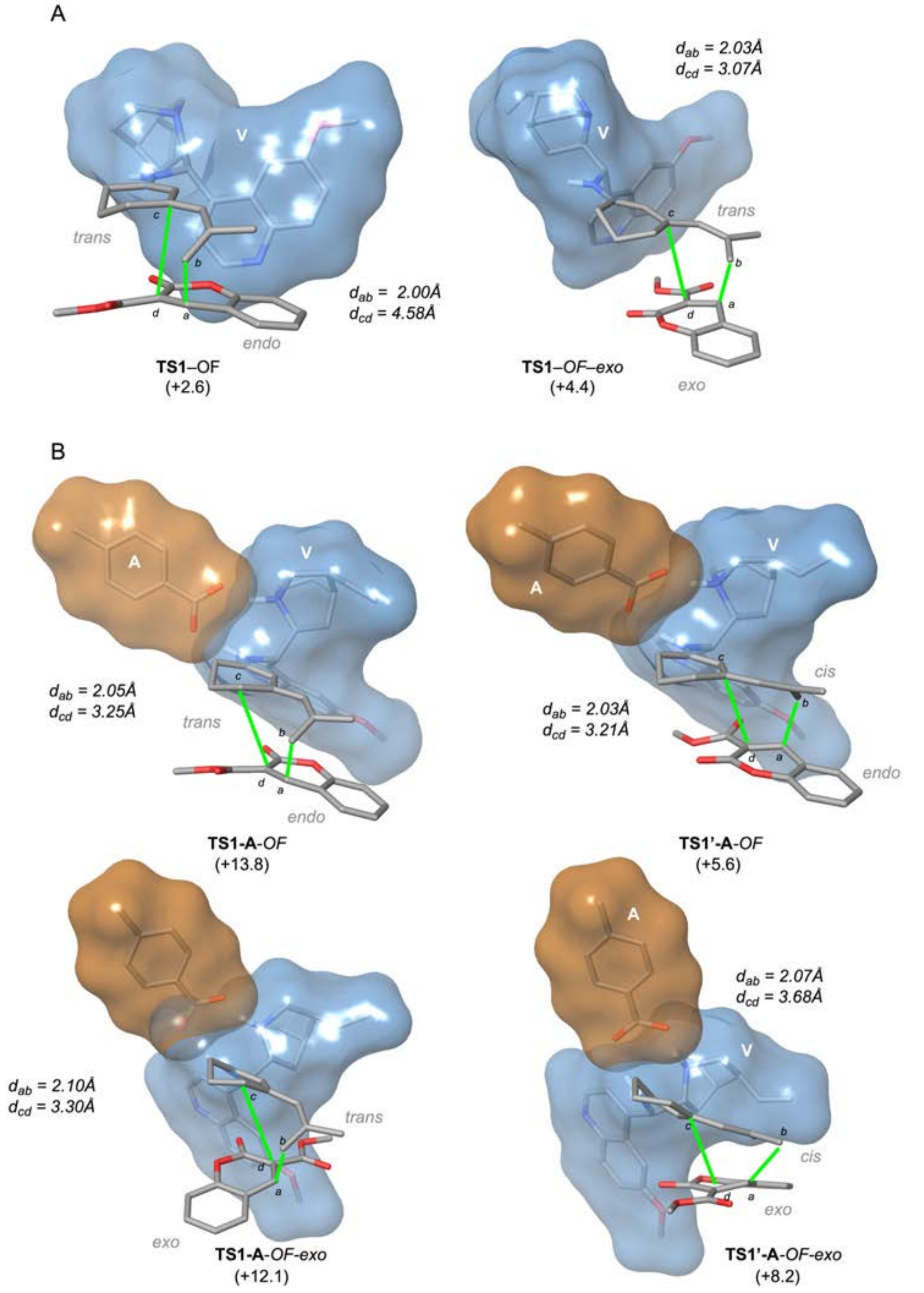

Figure S7. Main geometrical features and relative energy (related to (A) **TS1** and (B) **TS1**–A, respectively) of additional transition structures associated with the formation of spiro [4+2] cycloadducts **3a** and **3a'** from ketone **1a** and alkene **2a** catalyzed by **V** in (A) absence or (B) in presence of p–methyl benzoic acid computed at B3LYP-D3BJ(SCRF=PCM,toluene)/6-31G(d) level of theory. Relative Gibbs energies and distances are in kcal mol^{-1} and Å, respectively. The presence of acidic additive (p-methyl benzoic acid) is indicated as **A**.

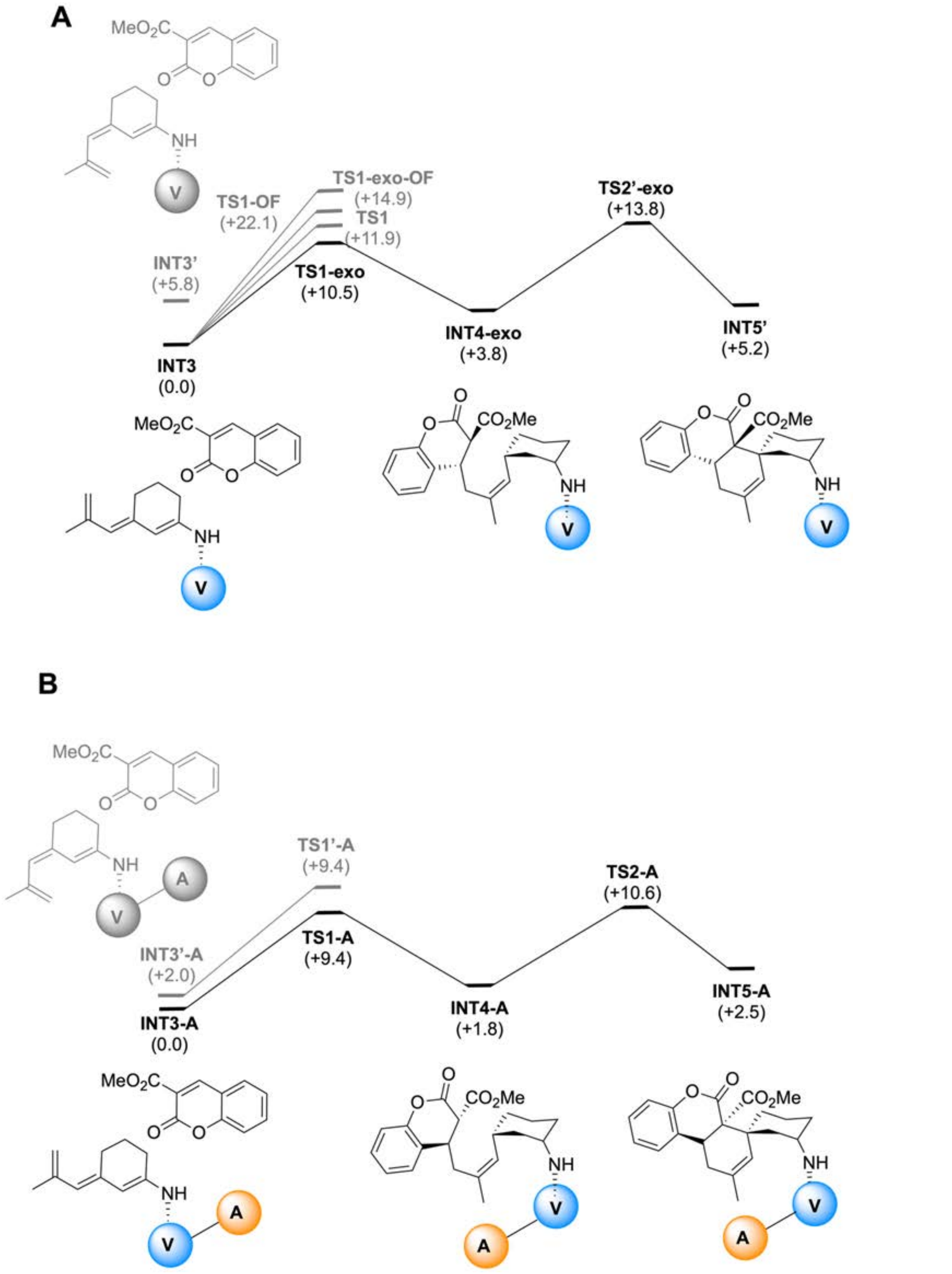

Figure S8. Energetic profiles associated with the formation of spiro [4+2] cycloadducts **3a** and **3a’** from ketone **1a** and alkene **2a** catalyzed by **V** in (A) absence or (B) in presence of p–methyl benzoic acid computed at B3LYP-D3BJ(SCRF=PCM,toluene)/6-31G(d) level of theory. Relative Gibbs energies and distances are in kcal mol^{-1} and Å, respectively. The presence of acidic additive (p-methyl benzoic acid) is indicated as **A**. Only complete profile of the most energetically favorable

processes and the second most stable transition structure associated with the first CC bond formation are included for clarity.

Table S4. Total electronic energies[a] (E, in a.u.), zero point correction of the energy[b] (ZPCE), thermal corrections to Gibbs free energies[b] (TCGFE, in a.u.), and number of imaginary frequencies[c] (NIMAG) of all stationary points discussed in the main text.

Structure	E	ZPCE	TCGFE	NIMAG(ν)
**2a**	-724.945969	0.170712	0.131106	0
**INT2**	-1405.043130	0.619334	0.555797	0
**INT2'**	-1405.035242	0.619559	0.556651	0
**INT3**-*exo*	-2130.017769	0.791829	0.711097	0
**INT3**-*endo*	-2130.019407	0.796327	0.719829	0
**INT3'**	-2130.010377	0.790693	0.713204	0
**TS1**	-2130.003342	0.792462	0.715664	1 (-452.9236)
**TS1**-*exo*	-2130.005450	0.792387	0.715493	1 (-302.4981)
**TS1**-*OF*	-2129.998883	0.792763	0.715326	1 (-493.3300)
**TS1**-*OF-exo*	-2129.992455	0.792264	0.711794	1 (-484.4314)
**INT4**-*exo*	-2130.020521	0.795610	0.719923	0
**TS2**-*exo*	-2130.002718	0.794536	0.717994	1 (-371.5974)
**INT5'**	-2130.013655	0.796631	0.719988	0
**INT2-A**	-1865.248520	0.765529	0.685817	0
**INT2'A**	-1865.244350	0.765723	0.687437	0
**INT3-A**	-2590.242957	0.939175	0.845546	0
**INT3'A**	-2590.238881	0.939210	0.844722	0
**TS1-A**	-2590.232074	0.940465	0.849626	1 (-360.3869)
**TS1-'A**	-2590.227070	0.939819	0.848487	1 (-393.1898)
**TS1-A**-*OF*	-2590.202687	0.938405	0.842174	1 (-403.8283)
**TS1'-A**-*OF*	-2590.217532	0.938803	0.845535	1 (-458.9858)
**TS1-A**-*OF-exo*	-2590.207424	0.937932	0.844306	1 (-333.1160)
**TS1'-A**-*OF-exo*	-2590.21550	0.938592	0.846076	1 (-360.9283)
**INT4-A**	-2590.246654	0.943052	0.852266	0
**TS2-A**	-2590.221496	0.942609	0.852678	1 (-377.9621)
**INT5-A**	-2590.227486	0.943965	0.853284	0

[a]Computed at B3LYP-D3BJ(PCM)/6-31G* level of theory. [b] Computed at 298.15 K. [c] If NIMAGE is not zero, imaginary frequency is in cm^{-1}.

Cartesian coordinates (optimized at the B3LYP-D3BJ(PCM)/6-31G* level) of all the stationary points collected in the main text

2a

```
 ---------------------------------------------------------------------
 Center     Atomic      Atomic             Coordinates (Angstroms)
 Number     Number       Type             X           Y           Z
 ---------------------------------------------------------------------
      1          6           0        3.182041    0.850466   -0.013567
      2          6           0        1.809023    0.603231   -0.011610
      3          6           0        1.303480   -0.709592    0.011723
      4          6           0        2.211184   -1.787354    0.031846
      5          6           0        3.576101   -1.551023    0.028397
      6          6           0        4.056364   -0.230508    0.006030
      7          8           0        0.971101    1.673723   -0.034179
```

8	6	0	-0.425879	1.571353	-0.035863
9	6	0	-0.961289	0.202547	0.006282
10	6	0	-0.116893	-0.867659	0.019726
11	6	0	-2.439158	0.016639	0.048219
12	8	0	-3.262614	0.888196	0.223857
13	8	0	-1.040901	2.606409	-0.081031
14	8	0	-2.767877	-1.287130	-0.126231
15	6	0	-4.174405	-1.573946	-0.061772
16	1	0	-0.531424	-1.869680	0.043204
17	1	0	3.536716	1.874912	-0.031185
18	1	0	5.126500	-0.047468	0.003857
19	1	0	4.274196	-2.381414	0.043432
20	1	0	1.820725	-2.800769	0.050051
21	1	0	-4.710708	-1.029264	-0.842542
22	1	0	-4.258675	-2.649447	-0.216144
23	1	0	-4.578420	-1.291378	0.913527

INT2

Center Number	Atomic Number	Atomic Type	Coordinates (Angstroms) X	Y	Z
1	6	0	-6.805098	-0.898165	-1.547358
2	6	0	-6.026973	0.033069	-0.960885
3	6	0	-6.633587	1.338708	-0.488728
4	6	0	-4.582500	-0.074982	-0.753934
5	6	0	-3.818955	-1.176648	-0.512246
6	6	0	-2.386072	-1.035515	-0.342843
7	6	0	-1.550835	-2.077218	-0.071818
8	6	0	-2.086210	-3.470845	0.156820
9	6	0	-3.553579	-3.431078	0.584452
10	6	0	-4.382322	-2.575818	-0.381376
11	7	0	-0.184037	-1.972596	-0.018900
12	6	0	0.546557	-0.734465	-0.166191
13	6	0	0.284795	0.256139	0.966614
14	6	0	-0.064525	-0.186073	2.222899
15	6	0	-0.309115	0.736889	3.265067
16	7	0	-0.246372	2.044980	3.121379
17	6	0	0.061933	2.520274	1.879517
18	6	0	0.335474	1.671429	0.754175
19	6	0	0.619140	2.272830	-0.494532
20	6	0	0.644666	3.648941	-0.636794
21	6	0	0.386341	4.487575	0.477711
22	6	0	0.102888	3.924724	1.700945
23	8	0	0.929880	4.117520	-1.884816
24	6	0	0.951397	5.524251	-2.092115
25	6	0	2.028831	-1.141488	-0.413188
26	7	0	2.494856	-2.182272	0.537951
27	6	0	3.069660	-1.585236	1.764807
28	6	0	4.359994	-0.775225	1.452647
29	6	0	4.428468	-0.587632	-0.073027
30	6	0	3.066943	-0.005965	-0.514371
31	6	0	4.677008	-1.981727	-0.696945
32	6	0	3.559910	-2.938280	-0.141564
33	6	0	4.761181	-2.016989	-2.202609
34	6	0	4.968177	-0.987331	-3.025546
35	1	0	1.992864	-1.651458	-1.381686
36	1	0	0.208884	-0.260361	-1.095440
37	1	0	-5.425462	-2.530203	-0.054678
38	1	0	-4.389496	-3.063977	-1.368098
39	1	0	-7.875955	-0.744970	-1.653604
40	1	0	-6.403682	-1.816182	-1.961260
41	1	0	-4.056591	0.881599	-0.750114
42	1	0	-6.143647	2.192375	-0.975967
43	1	0	-7.704622	1.391717	-0.706394
44	1	0	-6.492121	1.471757	0.591518
45	1	0	-1.978493	-0.035404	-0.458064
46	1	0	-3.960903	-4.447278	0.633466
47	1	0	-3.621943	-3.004556	1.593179
48	1	0	-1.975005	-4.059793	-0.765799
49	1	0	-1.477177	-3.976845	0.918325
50	1	0	0.361201	-2.714053	0.407428
51	1	0	3.270343	-2.401846	2.465983
52	1	0	2.312728	-0.947519	2.219128

53	1	0	3.094272	0.385970	-1.534291
54	1	0	2.817235	0.830070	0.145595
55	1	0	5.254530	-1.299713	1.809524
56	1	0	4.332219	0.198682	1.954002
57	1	0	5.240916	0.096874	-0.336235
58	1	0	3.109361	-3.520415	-0.952733
59	1	0	3.977263	-3.647928	0.579973
60	1	0	4.664983	-3.013822	-2.637769
61	1	0	-0.160936	-1.249031	2.415392
62	1	0	-0.570012	0.367332	4.255860
63	1	0	-0.103910	4.545596	2.566490
64	1	0	0.405826	5.565649	0.374212
65	1	0	0.831864	1.675617	-1.372229
66	1	0	5.036678	-1.131303	-4.100318
67	1	0	5.087752	0.031282	-2.667917
68	1	0	1.187184	5.664483	-3.147864
69	1	0	-0.023551	5.977452	-1.874808
70	1	0	1.722438	6.009279	-1.480803
71	1	0	5.643247	-2.344915	-0.315998

INT2'

Center Number	Atomic Number	Atomic Type	Coordinates (Angstroms) X	Y	Z
1	6	0	-6.805098	-0.898165	-1.547358
2	6	0	-6.026973	0.033069	-0.960885
3	6	0	-6.633587	1.338708	-0.488728
4	6	0	-4.582500	-0.074982	-0.753934
5	6	0	-3.818955	-1.176648	-0.512246
6	6	0	-2.386072	-1.035515	-0.342843
7	6	0	-1.550835	-2.077218	-0.071818
8	6	0	-2.086210	-3.470845	0.156820
9	6	0	-3.553579	-3.431078	0.584452
10	6	0	-4.382322	-2.575818	-0.381376
11	7	0	-0.184037	-1.972596	-0.018900
12	6	0	0.546557	-0.734465	-0.166191
13	6	0	0.284795	0.256139	0.966614
14	6	0	-0.064525	-0.186073	2.222899
15	6	0	-0.309115	0.736889	3.265067
16	7	0	-0.246372	2.044980	3.121379
17	6	0	0.061933	2.520274	1.879517
18	6	0	0.335474	1.671429	0.754175
19	6	0	0.619140	2.272830	-0.494532
20	6	0	0.644666	3.648941	-0.636794
21	6	0	0.386341	4.487575	0.477711
22	6	0	0.102888	3.924724	1.700945
23	8	0	0.929880	4.117520	-1.884816
24	6	0	0.951397	5.524251	-2.092115
25	6	0	2.028831	-1.141488	-0.413188
26	7	0	2.494856	-2.182272	0.537951
27	6	0	3.069660	-1.585236	1.764807
28	6	0	4.359994	-0.775225	1.452647
29	6	0	4.428468	-0.587632	-0.073027
30	6	0	3.066943	-0.005965	-0.514371
31	6	0	4.677008	-1.981727	-0.696945
32	6	0	3.559910	-2.938280	-0.141564
33	6	0	4.761181	-2.016989	-2.202609
34	6	0	4.968177	-0.987331	-3.025546
35	1	0	1.992864	-1.651458	-1.381686
36	1	0	0.208884	-0.260361	-1.095440
37	1	0	-5.425462	-2.530203	-0.054678
38	1	0	-4.389496	-3.063977	-1.368098
39	1	0	-7.875955	-0.744970	-1.653604
40	1	0	-6.403682	-1.816182	-1.961260
41	1	0	-4.056591	0.881599	-0.750114
42	1	0	-6.143647	2.192375	-0.975967
43	1	0	-7.704622	1.391717	-0.706394
44	1	0	-6.492121	1.471757	0.591518
45	1	0	-1.978493	-0.035404	-0.458064
46	1	0	-3.960903	-4.447278	0.633466
47	1	0	-3.621943	-3.004556	1.593179
48	1	0	-1.975005	-4.059793	-0.765799

49	1	0	-1.477177	-3.976845	0.918325
50	1	0	0.361201	-2.714053	0.407428
51	1	0	3.270343	-2.401846	2.465983
52	1	0	2.312728	-0.947519	2.219128
53	1	0	3.094272	0.385970	-1.534291
54	1	0	2.817235	0.830070	0.145595
55	1	0	5.254530	-1.299713	1.809524
56	1	0	4.332219	0.198682	1.954002
57	1	0	5.240916	0.096874	-0.336235
58	1	0	3.109361	-3.520415	-0.952733
59	1	0	3.977263	-3.647928	0.579973
60	1	0	4.664983	-3.013822	-2.637769
61	1	0	-0.160936	-1.249031	2.415392
62	1	0	-0.570012	0.367332	4.255860
63	1	0	-0.103910	4.545596	2.566490
64	1	0	0.405826	5.565649	0.374212
65	1	0	0.831864	1.675617	-1.372229
66	1	0	5.036678	-1.131303	-4.100318
67	1	0	5.087752	0.031282	-2.667917
68	1	0	1.187184	5.664483	-3.147864
69	1	0	-0.023551	5.977452	-1.874808
70	1	0	1.722438	6.009279	-1.480803
71	1	0	5.643247	-2.344915	-0.315998

INT3–*exo*

Center Number	Atomic Number	Atomic Type	Coordinates (Angstroms) X	Y	Z
1	6	0	-6.076195	-2.654133	-0.601079
2	6	0	-5.012565	-1.777193	-0.812641
3	6	0	-5.226895	-0.438329	-1.177876
4	6	0	-6.550702	0.014005	-1.338963
5	6	0	-7.615210	-0.849439	-1.136505
6	6	0	-7.374325	-2.183159	-0.765962
7	8	0	-3.748822	-2.258109	-0.638700
8	6	0	-2.590959	-1.500160	-0.848894
9	6	0	-2.806807	-0.094537	-1.207946
10	6	0	-4.074756	0.392841	-1.346653
11	8	0	-1.545532	-2.085544	-0.694206
12	6	0	-1.621988	0.776989	-1.421703
13	8	0	-0.463967	0.412763	-1.436164
14	8	0	-1.987483	2.065057	-1.632275
15	6	0	-0.894882	2.984545	-1.795379
16	6	0	-4.905541	1.434575	1.536027
17	6	0	-3.769978	2.168128	1.482733
18	6	0	-3.865171	3.654970	1.202706
19	6	0	-2.407580	1.673759	1.580733
20	6	0	-1.913419	0.464297	1.985472
21	6	0	-0.508527	0.199790	1.804719
22	6	0	0.046769	-1.032004	1.971110
23	6	0	-0.749485	-2.172267	2.549783
24	6	0	-1.924096	-1.647113	3.377298
25	6	0	-2.760183	-0.644639	2.572366
26	7	0	1.341479	-1.329838	1.611538
27	6	0	2.059869	-0.478003	0.668172
28	6	0	2.645121	0.739054	1.364316
29	6	0	3.016830	0.706375	2.687154
30	6	0	3.597745	1.849427	3.287264
31	7	0	3.810526	2.987894	2.660160
32	6	0	3.438779	3.054993	1.346025
33	6	0	2.851543	1.955645	0.637273
34	6	0	2.493893	2.123051	-0.721446
35	6	0	2.698892	3.335194	-1.358052
36	6	0	3.281798	4.424151	-0.662513
37	6	0	3.641135	4.275667	0.658990
38	8	0	2.299012	3.402709	-2.663794
39	6	0	2.489722	4.622065	-3.368244
40	6	0	3.186429	-1.211668	-0.110465
41	7	0	2.680982	-1.764961	-1.382542
42	6	0	3.827951	-2.245459	-2.159907
43	6	0	4.539099	-3.466658	-1.470087

44	6	0	3.900364	-3.623768	-0.073686
45	6	0	3.974779	-2.289972	0.698132
46	6	0	2.419525	-3.995550	-0.282675
47	6	0	1.752679	-2.885200	-1.152082
48	6	0	6.037389	-3.327541	-1.507500
49	6	0	6.896935	-3.493623	-0.500546
50	1	0	3.887508	-0.427752	-0.409396
51	1	0	1.340655	-0.140573	-0.083795
52	1	0	-3.545756	-0.215096	3.203031
53	1	0	-3.278489	-1.182436	1.766045
54	1	0	-4.215262	1.434756	-1.605725
55	1	0	-5.875212	1.912085	1.425073
56	1	0	-4.917700	0.362328	1.671216
57	1	0	-1.658719	2.385359	1.233026
58	1	0	-4.903959	3.990800	1.132302
59	1	0	-3.365550	4.239729	1.985202
60	1	0	-3.361936	3.893214	0.256397
61	1	0	-5.869976	-3.679681	-0.315266
62	1	0	-8.209748	-2.857539	-0.604786
63	1	0	-8.634101	-0.497050	-1.259724
64	1	0	-6.718638	1.051270	-1.613262
65	1	0	0.084306	1.003552	1.384449
66	1	0	-2.556222	-2.478366	3.708600
67	1	0	-1.536289	-1.154071	4.277393
68	1	0	-1.114138	-2.796577	1.722089
69	1	0	-0.095690	-2.803254	3.166660
70	1	0	1.553645	-2.315378	1.575011
71	1	0	1.476467	-3.279231	-2.135316
72	1	0	0.835806	-2.498315	-0.706768
73	1	0	5.014472	-1.976217	0.818320
74	1	0	3.576101	-2.424181	1.709694
75	1	0	2.342691	-4.974025	-0.770862
76	1	0	1.919932	-4.101828	0.690395
77	1	0	4.407363	-4.414281	0.489429
78	1	0	4.529247	-1.410977	-2.273328
79	1	0	3.475583	-2.517773	-3.160458
80	1	0	6.437332	-3.062932	-2.488768
81	1	0	2.847267	-0.190869	3.272204
82	1	0	3.893804	1.809756	4.335069
83	1	0	4.087006	5.096358	1.211750
84	1	0	3.445435	5.373116	-1.158802
85	1	0	2.039375	1.320088	-1.288979
86	1	0	7.966619	-3.371471	-0.647483
87	1	0	6.576895	-3.761931	0.502282
88	1	0	-0.313428	3.047200	-0.872246
89	1	0	-0.232812	2.670182	-2.603404
90	1	0	-1.357411	3.945612	-2.021175
91	1	0	2.108072	4.445720	-4.374852
92	1	0	1.930534	5.446053	-2.907197
93	1	0	3.551337	4.892333	-3.425132
94	1	0	4.289118	-4.369019	-2.048694

INT3–*endo*

Center Number	Atomic Number	Atomic Type	Coordinates (Angstroms) X	Y	Z
1	6	0	-3.830842	2.140855	-1.543724
2	6	0	-3.411727	0.810762	-1.499233
3	6	0	-4.330627	-0.251372	-1.459781
4	6	0	-5.706783	0.047705	-1.480949
5	6	0	-6.136099	1.363559	-1.537086
6	6	0	-5.194807	2.407863	-1.563918
7	8	0	-2.072206	0.576927	-1.510839
8	6	0	-1.509489	-0.693614	-1.438176
9	6	0	-2.456128	-1.802810	-1.297256
10	6	0	-3.801129	-1.572899	-1.342488
11	8	0	-0.302897	-0.743821	-1.512902
12	6	0	-2.019505	-3.208414	-1.089283
13	8	0	-2.805237	-4.118988	-0.876236
14	8	0	-0.691099	-3.391745	-1.144233
15	6	0	-0.252718	-4.747015	-0.961227
16	6	0	-4.788546	-1.964391	1.636409
17	6	0	-4.241964	-0.742748	1.826546

18	6	0	-5.138392	0.458291	2.042416
19	6	0	-2.821068	-0.430786	1.768849
20	6	0	-1.739351	-1.234347	1.990788
21	6	0	-0.412922	-0.712987	1.760019
22	6	0	0.711653	-1.476560	1.833188
23	6	0	0.667470	-2.892239	2.347996
24	6	0	-0.580726	-3.124157	3.200493
25	6	0	-1.842021	-2.672390	2.455663
26	7	0	1.951105	-1.028802	1.442490
27	6	0	2.081335	0.145687	0.587606
28	6	0	2.031291	1.420569	1.415935
29	6	0	2.688445	1.496479	2.622390
30	6	0	2.693858	2.706198	3.354464
31	7	0	2.088224	3.808124	2.962373
32	6	0	1.404510	3.757549	1.780262
33	6	0	1.333782	2.583807	0.956036
34	6	0	0.574902	2.628764	-0.239247
35	6	0	-0.075424	3.791893	-0.614649
36	6	0	0.006526	4.956737	0.188604
37	6	0	0.730515	4.928243	1.358741
38	8	0	-0.791480	3.738925	-1.780039
39	6	0	-1.304412	4.953717	-2.311592
40	6	0	3.361050	0.136915	-0.290825
41	7	0	3.109916	-0.520595	-1.589167
42	6	0	4.283943	-0.315312	-2.443401
43	6	0	5.553699	-1.061412	-1.892171
44	6	0	5.181057	-1.593661	-0.492441
45	6	0	4.644223	-0.443936	0.386331
46	6	0	4.067802	-2.643312	-0.680613
47	6	0	2.879822	-1.965529	-1.430433
48	6	0	6.783257	-0.197132	-1.972331
49	6	0	7.685268	0.027184	-1.015097
50	1	0	3.545222	1.186124	-0.540000
51	1	0	1.245407	0.133827	-0.114297
52	1	0	-2.726110	-2.793247	3.089898
53	1	0	-1.999863	-3.334022	1.592993
54	1	0	-4.475728	-2.417806	-1.266047
55	1	0	-5.865916	-2.099850	1.686662
56	1	0	-4.203232	-2.843689	1.399730
57	1	0	-2.598212	0.605066	1.509623
58	1	0	-6.197290	0.185135	2.017086
59	1	0	-4.926753	0.941383	3.004738
60	1	0	-4.963277	1.210116	1.262838
61	1	0	-3.077447	2.919307	-1.563400
62	1	0	-5.535658	3.438327	-1.598316
63	1	0	-7.197150	1.590498	-1.549397
64	1	0	-6.419832	-0.769923	-1.434920
65	1	0	-0.339078	0.312856	1.414692
66	1	0	-0.663637	-4.182542	3.471773
67	1	0	-0.485439	-2.555511	4.133677
68	1	0	0.679031	-3.585261	1.493916
69	1	0	1.575981	-3.098972	2.929434
70	1	0	2.666768	-1.734557	1.368027
71	1	0	2.768977	-2.382790	-2.436618
72	1	0	1.923206	-2.113665	-0.926574
73	1	0	5.398262	0.339985	0.488511
74	1	0	4.451069	-0.814938	1.399019
75	1	0	4.452368	-3.499434	-1.247325
76	1	0	3.757726	-3.039722	0.296404
77	1	0	6.051814	-2.056267	-0.016384
78	1	0	4.471637	0.762718	-2.501171
79	1	0	4.040031	-0.661500	-3.453389
80	1	0	6.927653	0.285838	-2.941110
81	1	0	3.190976	0.621617	3.020601
82	1	0	3.226734	2.755389	4.303530
83	1	0	0.800444	5.804665	1.994814
84	1	0	-0.501442	5.867412	-0.105177
85	1	0	0.479599	1.764837	-0.886402
86	1	0	8.543021	0.670960	-1.189449
87	1	0	7.613231	-0.420909	-0.028110
88	1	0	-0.556609	-5.121487	0.019577
89	1	0	-0.672573	-5.393652	-1.735548
90	1	0	0.833662	-4.708490	-1.038968
91	1	0	-1.749797	4.691277	-3.272422
92	1	0	-2.076099	5.388339	-1.663171
93	1	0	-0.506978	5.690329	-2.467806
94	1	0	5.735584	-1.933781	-2.538293

INT3'

Center Number	Atomic Number	Atomic Type	Coordinates (Angstroms) X	Y	Z
1	6		0.000058810	0.000237211	0.000238828
2	6		0.000084367	0.000185151	0.000224803
3	6		0.000272312	0.000074158	0.000072136
4	6		0.000070354	0.000039170	0.000078883
5	6		0.000165897	0.000117804	0.000110518
6	6		0.000090766	0.000215509	0.000158285
7	8		0.000156296	0.000328909	0.000215510
8	6		0.000282954	0.000023575	0.000223690
9	6		0.000071444	0.000143649	-0.000255965
10	6		0.000194632	-0.000096004	-0.000041596
11	8		-0.000331001	0.000366491	0.000194115
12	6		0.000055647	0.000458459	-0.000266574
13	8		-0.001021304	0.000831109	-0.000436464
14	8		0.000494625	-0.001481352	0.001603658
15	6		0.000224174	-0.000742816	-0.000219594
16	6		-0.001445292	-0.001262164	0.001808877
17	6		0.004055463	0.000337058	-0.002440735
18	6		-0.003683976	0.001219071	-0.001753158
19	6		0.001911276	-0.001602788	-0.000742833
20	6		-0.000841013	0.000600105	-0.000397015
21	6		-0.000871368	0.000782619	-0.000077780
22	6		0.000131984	0.000178281	0.000306710
23	6		0.000086482	0.000070813	0.000122062
24	6		-0.000260324	-0.000114302	-0.000185930
25	6		-0.000013305	-0.000110724	0.000506935
26	7		0.000227525	-0.001012107	-0.000190185
27	6		-0.000024626	0.000083551	-0.000058684
28	6		-0.000027377	0.000017820	-0.000109559
29	6		-0.000092133	0.000007241	-0.000088301
30	6		-0.000124392	-0.000006007	-0.000065778
31	7		-0.000122161	0.000005757	-0.000056642
32	6		-0.000068996	-0.000005190	-0.000064500
33	6		-0.000001165	-0.000000728	-0.000093734
34	6		0.000069305	0.000003254	-0.000049137
35	6		0.000059865	0.000042122	-0.000131068
36	6		-0.000041448	0.000000763	-0.000046729
37	6		-0.000097899	0.000027535	-0.000079501
38	8		0.000104778	0.000011575	-0.000045166
39	6		-0.000015067	0.000069654	-0.000062657
40	6		-0.000056810	-0.000031019	-0.000128671
41	7		0.000081065	-0.000124661	-0.000051610
42	6		0.000036045	-0.000034419	-0.000008810
43	6		0.000056425	-0.000019886	0.000021650
44	6		-0.000007968	-0.000007045	-0.000001424
45	6		-0.000040493	0.000027796	-0.000005936
46	6		0.000030275	-0.000017756	-0.000056072
47	6		0.000032063	-0.000132894	-0.000045647
48	6		0.000060475	-0.000028727	0.000062612
49	6		0.000046936	0.000006784	0.000088643
50	1		-0.000030095	-0.000019164	-0.000081468
51	1		0.000012280	0.000088879	-0.000149890
52	1		0.000099833	-0.000378017	-0.000531349
53	1		-0.000203335	-0.000345420	0.000150124
54	1		0.000136096	-0.000298654	-0.000186064
55	1		0.000129525	-0.000803178	0.002339923
56	1		-0.003305621	0.001195855	0.001116586
57	1		0.000290555	-0.000199847	0.000118645
58	1		-0.001656885	-0.002055916	0.003600699
59	1		0.003406004	0.003828120	0.000906647
60	1		0.001621620	-0.002687099	-0.004292086
61	1		-0.000007300	0.000261991	0.000283008
62	1		0.000104571	0.000253035	0.000117122
63	1		0.000144458	0.000149754	0.000057104
64	1		0.000113857	0.000010320	0.000056321
65	1		-0.000370750	-0.000104029	0.000033538
66	1		0.000294210	0.000177006	-0.000139411
67	1		-0.000286452	0.000214055	-0.000503571
68	1		0.000101781	0.000227835	-0.000267195

69	1	-0.000101439	-0.000061323	-0.000149799
70	1	-0.000302654	0.001041095	-0.000077147
71	1	0.000086570	-0.000093677	-0.000102809
72	1	0.000056050	-0.000142533	-0.000187326
73	1	-0.000045227	0.000030835	0.000001939
74	1	-0.000075106	0.000032699	-0.000046283
75	1	0.000050878	-0.000038948	-0.000022547
76	1	-0.000022406	-0.000001385	-0.000047900
77	1	-0.000005022	0.000028002	0.000026888
78	1	0.000044050	-0.000049790	0.000006338
79	1	0.000130586	-0.000061097	-0.000040111
80	1	0.000093813	-0.000063445	0.000057690
81	1	-0.000127097	-0.000009177	-0.000098400
82	1	-0.000157043	-0.000027160	-0.000042489
83	1	-0.000130467	0.000021144	0.000007614
84	1	-0.000021654	0.000026406	-0.000006744
85	1	-0.000002307	0.000017315	-0.000125802
86	1	0.000047867	-0.000013377	0.000096563
87	1	0.000029123	0.000026226	0.000074300
88	1	-0.000525912	-0.000127310	-0.000482591
89	1	0.000332176	-0.000032916	0.000751280
90	1	-0.000026838	0.000223885	0.000054182
91	1	0.000046652	0.000064796	-0.000042394
92	1	-0.000039897	-0.000068607	-0.000036972
93	1	-0.000044955	0.000122958	0.000001879
94	1	0.000091783	-0.000042548	0.000021496

TS1

Center Number	Atomic Number	Atomic Type	Coordinates (Angstroms) X	Y	Z
1	6	0	4.039569	-2.003862	-1.743330
2	6	0	3.574474	-0.731026	-1.407034
3	6	0	4.448007	0.356331	-1.274300
4	6	0	5.811372	0.143420	-1.520847
5	6	0	6.289643	-1.116683	-1.866183
6	6	0	5.400331	-2.193883	-1.967232
7	8	0	2.226102	-0.598611	-1.227153
8	6	0	1.609486	0.655014	-1.093697
9	6	0	2.473370	1.787308	-0.912512
10	6	0	3.895884	1.640702	-0.830371
11	8	0	0.389618	0.623050	-1.148166
12	6	0	1.964700	3.143778	-0.800343
13	8	0	2.678685	4.113951	-0.539270
14	8	0	0.625497	3.292409	-0.979109
15	6	0	0.157499	4.643015	-0.932026
16	6	0	4.471313	1.857653	1.106278
17	6	0	4.046905	0.654624	1.713145
18	6	0	5.027279	-0.494079	1.794722
19	6	0	2.707345	0.361051	1.984385
20	6	0	1.608525	1.228407	2.087753
21	6	0	0.321258	0.686510	1.893146
22	6	0	-0.815559	1.470816	1.870535
23	6	0	-0.781898	2.893814	2.362620
24	6	0	0.456251	3.148403	3.223625
25	6	0	1.721611	2.685205	2.490525
26	7	0	-1.989088	1.029093	1.379624
27	6	0	-2.105470	-0.178691	0.562363
28	6	0	-2.102390	-1.434530	1.414503
29	6	0	-2.795631	-1.492398	2.601157
30	6	0	-2.824855	-2.694660	3.346510
31	7	0	-2.209810	-3.800743	2.983143
32	6	0	-1.494717	-3.768106	1.818873
33	6	0	-1.399537	-2.605533	0.981375
34	6	0	-0.613978	-2.665063	-0.195526
35	6	0	0.052243	-3.831132	-0.535641
36	6	0	-0.050933	-4.984721	0.282752
37	6	0	-0.808082	-4.943061	1.431560
38	8	0	0.795357	-3.790545	-1.679630
39	6	0	1.420261	-4.987099	-2.123759
40	6	0	-3.343654	-0.169004	-0.374912
41	7	0	-3.004675	0.416520	-1.685055
42	6	0	-4.150551	0.233813	-2.581639

43	6	0	-5.394156	1.081871	-2.127707
44	6	0	-5.061585	1.638622	-0.727111
45	6	0	-4.640534	0.483155	0.206130
46	6	0	-3.880722	2.616699	-0.889584
47	6	0	-2.704326	1.853879	-1.572011
48	6	0	-6.672696	0.297346	-2.248907
49	6	0	-7.636239	0.163251	-1.335701
50	1	0	-3.550393	-1.223211	-0.580145
51	1	0	-1.233666	-0.177892	-0.092411
52	1	0	2.602391	2.815706	3.127061
53	1	0	1.876654	3.328571	1.619367
54	1	0	4.465654	2.525549	-1.099753
55	1	0	5.544203	2.007509	1.003120
56	1	0	3.912859	2.775335	1.235635
57	1	0	2.459138	-0.700008	2.021470
58	1	0	6.051141	-0.167837	1.599092
59	1	0	4.994075	-0.961083	2.785853
60	1	0	4.779603	-1.270159	1.059953
61	1	0	3.317124	-2.808206	-1.827300
62	1	0	5.768650	-3.181542	-2.229398
63	1	0	7.349202	-1.264181	-2.051484
64	1	0	6.496435	0.981822	-1.422840
65	1	0	0.257639	-0.350545	1.589030
66	1	0	0.532770	4.213870	3.463572
67	1	0	0.357300	2.604817	4.170789
68	1	0	-0.775932	3.555967	1.486380
69	1	0	-1.702343	3.113183	2.919060
70	1	0	-2.766000	1.672638	1.393116
71	1	0	-2.542252	2.224801	-2.589093
72	1	0	-1.755908	1.973459	-1.046274
73	1	0	-5.435840	-0.263652	0.264077
74	1	0	-4.510840	0.858756	1.228538
75	1	0	-4.191673	3.479022	-1.490861
76	1	0	-3.587059	3.019384	0.089938
77	1	0	-5.926909	2.163384	-0.309460
78	1	0	-4.401187	-0.832962	-2.592655
79	1	0	-3.843119	0.510186	-3.595472
80	1	0	-6.797614	-0.207492	-3.209117
81	1	0	-3.314218	-0.615777	2.975948
82	1	0	-3.384602	-2.732409	4.280355
83	1	0	-0.894149	-5.811554	2.076469
84	1	0	0.467341	-5.898052	0.016858
85	1	0	-0.488098	-1.805736	-0.843949
86	1	0	-8.524176	-0.429667	-1.537061
87	1	0	-7.586988	0.638841	-0.360149
88	1	0	0.357462	5.099007	0.042377
89	1	0	0.636417	5.250828	-1.704635
90	1	0	-0.917429	4.581872	-1.109331
91	1	0	1.910299	-4.732691	-3.064706
92	1	0	2.172843	-5.340628	-1.407519
93	1	0	0.685792	-5.782654	-2.300421
94	1	0	-5.480510	1.940363	-2.810876

TS1–*exo*

Center Number	Atomic Number	Atomic Type	Coordinates (Angstroms) X	Y	Z
1	6	0	-6.051402	-2.662816	-0.621833
2	6	0	-5.027130	-1.716069	-0.609634
3	6	0	-5.261122	-0.380319	-0.960033
4	6	0	-6.559836	-0.008985	-1.338439
5	6	0	-7.590116	-0.943333	-1.361583
6	6	0	-7.334165	-2.271121	-0.997246
7	8	0	-3.779790	-2.135966	-0.229957
8	6	0	-2.615710	-1.370486	-0.479836
9	6	0	-2.826949	-0.000519	-0.861312
10	6	0	-4.140490	0.554404	-0.871577
11	8	0	-1.573377	-1.972406	-0.291537
12	6	0	-1.665845	0.808974	-1.206193
13	8	0	-0.495301	0.453721	-1.251421
14	8	0	-2.027822	2.093640	-1.527146
15	6	0	-0.935771	2.975477	-1.798813
16	6	0	-4.600453	1.379789	1.013059

17	6	0	-3.531072	2.261558	1.255241
18	6	0	-3.707492	3.694701	0.804688
19	6	0	-2.247842	1.852643	1.628546
20	6	0	-1.788014	0.588381	2.041622
21	6	0	-0.421087	0.307805	1.865338
22	6	0	0.098943	-0.963691	2.038992
23	6	0	-0.686634	-2.030764	2.753807
24	6	0	-1.820682	-1.409323	3.570986
25	6	0	-2.656266	-0.465971	2.696126
26	7	0	1.306670	-1.312848	1.560923
27	6	0	2.048703	-0.488157	0.608120
28	6	0	2.727851	0.681036	1.299099
29	6	0	3.187356	0.592152	2.591787
30	6	0	3.862497	1.689259	3.179419
31	7	0	4.086722	2.832567	2.566283
32	6	0	3.628884	2.955766	1.284099
33	6	0	2.938330	1.907389	0.590462
34	6	0	2.490611	2.131451	-0.732423
35	6	0	2.714294	3.349770	-1.351796
36	6	0	3.401920	4.387264	-0.672517
37	6	0	3.844609	4.184356	0.615732
38	8	0	2.230518	3.474240	-2.622018
39	6	0	2.416442	4.708130	-3.302662
40	6	0	3.098540	-1.279624	-0.218409
41	7	0	2.509195	-1.805217	-1.462576
42	6	0	3.592824	-2.354945	-2.284097
43	6	0	4.248829	-3.621845	-1.622955
44	6	0	3.671364	-3.728746	-0.195442
45	6	0	3.872311	-2.395405	0.554369
46	6	0	2.161184	-4.005012	-0.327549
47	6	0	1.526295	-2.868427	-1.185583
48	6	0	5.749290	-3.589582	-1.736004
49	6	0	6.644501	-3.809276	-0.771246
50	1	0	3.821093	-0.527259	-0.546049
51	1	0	1.311203	-0.114185	-0.106446
52	1	0	-3.432026	0.022879	3.294454
53	1	0	-3.174817	-1.065357	1.940605
54	1	0	-4.260390	1.494241	-1.396933
55	1	0	-5.574089	1.809804	0.789353
56	1	0	-4.650813	0.425607	1.517009
57	1	0	-1.457161	2.578486	1.441752
58	1	0	-4.573234	4.157988	1.291868
59	1	0	-2.824102	4.300519	1.024439
60	1	0	-3.878982	3.733826	-0.278330
61	1	0	-5.824965	-3.687303	-0.345432
62	1	0	-8.135384	-3.004072	-1.011858
63	1	0	-8.589756	-0.642961	-1.660396
64	1	0	-6.748913	1.025869	-1.612386
65	1	0	0.184652	1.065804	1.386901
66	1	0	-2.459896	-2.196550	3.983334
67	1	0	-1.400324	-0.850826	4.416144
68	1	0	-1.092363	-2.708221	1.992610
69	1	0	-0.013960	-2.615648	3.394425
70	1	0	1.586919	-2.275323	1.678466
71	1	0	1.196817	-3.256353	-2.154612
72	1	0	0.646880	-2.427459	-0.715366
73	1	0	4.933914	-2.142102	0.604575
74	1	0	3.540285	-2.501399	1.594338
75	1	0	1.998188	-4.984676	-0.791197
76	1	0	1.700944	-4.061868	0.668945
77	1	0	4.155366	-4.544781	0.351010
78	1	0	4.342182	-1.567331	-2.423337
79	1	0	3.185956	-2.600319	-3.270549
80	1	0	6.116993	-3.359732	-2.738312
81	1	0	3.024176	-0.311502	3.169457
82	1	0	4.228114	1.605427	4.202169
83	1	0	4.369567	4.965794	1.155670
84	1	0	3.579474	5.340421	-1.155760
85	1	0	1.945722	1.373553	-1.282242
86	1	0	7.711354	-3.763678	-0.972196
87	1	0	6.357370	-4.049128	0.248689
88	1	0	-0.318399	3.112025	-0.905753
89	1	0	-0.300519	2.590238	-2.598226
90	1	0	-1.390142	3.923877	-2.090371
91	1	0	1.950366	4.579559	-4.280382
92	1	0	1.929094	5.536904	-2.774017
93	1	0	3.480768	4.938008	-3.435523

94	1	0	3.905897	-4.505484	-2.182163

TS1-OF

Center Number	Atomic Number	Atomic Type	Coordinates (Angstroms) X	Y	Z
1	6	0	2.998006	2.887537	-1.352337
2	6	0	3.374962	1.577943	-1.041984
3	6	0	4.649155	1.282507	-0.540142
4	6	0	5.562972	2.334791	-0.388994
5	6	0	5.208471	3.642505	-0.703899
6	6	0	3.918317	3.916895	-1.175626
7	8	0	2.447237	0.601701	-1.277140
8	6	0	2.760518	-0.764826	-1.211118
9	6	0	4.056826	-1.101422	-0.692230
10	6	0	4.950818	-0.107365	-0.174194
11	8	0	1.872963	-1.509465	-1.602788
12	6	0	4.552048	-2.470176	-0.644416
13	8	0	5.509324	-2.818138	0.047832
14	8	0	3.899618	-3.354755	-1.439407
15	6	0	4.369095	-4.703635	-1.355296
16	6	0	4.979094	-0.232926	1.819751
17	6	0	3.882697	0.549489	2.242536
18	6	0	4.157462	1.990133	2.619047
19	6	0	2.524583	0.227024	2.108780
20	6	0	1.844631	-0.962226	1.817847
21	6	0	0.501150	-0.828878	1.395510
22	6	0	-0.279170	-1.895086	0.997026
23	6	0	0.205644	-3.313939	1.136813
24	6	0	1.731563	-3.386980	1.090955
25	6	0	2.372974	-2.364027	2.036263
26	7	0	-1.527029	-1.741851	0.505255
27	6	0	-2.255113	-0.480547	0.435607
28	6	0	-1.649579	0.498232	-0.561025
29	6	0	-0.855408	0.083009	-1.599438
30	6	0	-0.300290	1.036505	-2.488664
31	7	0	-0.494917	2.334028	-2.395360
32	6	0	-1.281488	2.778181	-1.367851
33	6	0	-1.893375	1.903194	-0.412290
34	6	0	-2.679025	2.462830	0.621806
35	6	0	-2.859686	3.833242	0.715560
36	6	0	-2.255363	4.699889	-0.228807
37	6	0	-1.485388	4.172263	-1.241994
38	8	0	-3.636147	4.265643	1.750150
39	6	0	-3.854009	5.663162	1.893876
40	6	0	-3.757407	-0.689193	0.127024
41	7	0	-4.525928	-1.084978	1.325979
42	6	0	-5.953487	-0.952425	1.000041
43	6	0	-6.392391	-1.943871	-0.139357
44	6	0	-5.116373	-2.656428	-0.639315
45	6	0	-4.060090	-1.625524	-1.082362
46	6	0	-4.539012	-3.464966	0.537164
47	6	0	-4.270753	-2.483568	1.717981
48	6	0	-7.217088	-1.248315	-1.190449
49	6	0	-7.089825	-1.336994	-2.515462
50	1	0	-4.127595	0.308212	-0.118932
51	1	0	-2.206712	-0.032939	1.434487
52	1	0	3.455262	-2.413691	1.946575
53	1	0	2.136355	-2.629943	3.078900
54	1	0	6.002062	-0.382676	-0.208335
55	1	0	5.964144	0.163526	2.058450
56	1	0	4.951991	-1.313623	1.827532
57	1	0	1.867732	1.096074	2.159209
58	1	0	5.227170	2.196075	2.695493
59	1	0	3.735655	2.670652	1.869449
60	1	0	3.688292	2.231922	3.580484
61	1	0	2.000017	3.065339	-1.741251
62	1	0	3.632960	4.936677	-1.417690
63	1	0	5.927676	4.446366	-0.579446
64	1	0	6.555855	2.112732	-0.005814
65	1	0	0.118795	0.177176	1.271421
66	1	0	2.063721	-4.395424	1.360339
67	1	0	2.052283	-3.187066	0.067634

68	1	0	-0.174017	-3.705489	2.092880
69	1	0	-0.232824	-3.936763	0.346935
70	1	0	-1.972897	-2.563393	0.125261
71	1	0	-4.931760	-2.702848	2.562319
72	1	0	-3.246847	-2.564980	2.088310
73	1	0	-4.420419	-1.033180	-1.925948
74	1	0	-3.166476	-2.150346	-1.442164
75	1	0	-5.238932	-4.253187	0.836412
76	1	0	-3.618311	-3.974406	0.220150
77	1	0	-5.358074	-3.328617	-1.468550
78	1	0	-6.132901	0.084188	0.695277
79	1	0	-6.529763	-1.131010	1.913631
80	1	0	-8.007886	-0.611318	-0.789037
81	1	0	-0.580312	-0.956538	-1.716377
82	1	0	0.351942	0.691873	-3.286602
83	1	0	-1.011666	4.816758	-1.975518
84	1	0	-2.391293	5.772364	-0.160792
85	1	0	-3.164779	1.841605	1.365807
86	1	0	-7.752989	-0.791977	-3.181439
87	1	0	-6.333263	-1.954536	-2.991161
88	1	0	5.421528	-4.771224	-1.643835
89	1	0	4.257767	-5.091311	-0.338323
90	1	0	3.746224	-5.271026	-2.047851
91	1	0	-4.492354	5.775177	2.771354
92	1	0	-2.912924	6.202357	2.058736
93	1	0	-4.364142	6.082917	1.018029
94	1	0	-7.037141	-2.710840	0.315707

TS1-*OF-exo*

Center Number	Atomic Number	Atomic Type	Coordinates (Angstroms) X	Y	Z
1	6	0	7.610587	-2.072135	-0.437738
2	6	0	6.464928	-1.272678	-0.443370
3	6	0	6.445741	-0.028451	0.199033
4	6	0	7.611611	0.413006	0.840811
5	6	0	8.761745	-0.369177	0.847309
6	6	0	8.755092	-1.617668	0.211707
7	8	0	5.363614	-1.745929	-1.098069
8	6	0	4.281948	-0.896994	-1.473394
9	6	0	4.222545	0.369550	-0.804429
10	6	0	5.185713	0.723454	0.196414
11	8	0	3.538253	-1.382965	-2.306471
12	6	0	3.197162	1.368236	-1.075790
13	8	0	3.177815	2.476453	-0.538010
14	8	0	2.250329	0.996869	-1.969215
15	6	0	1.217943	1.959940	-2.195366
16	6	0	4.530063	0.293734	2.029732
17	6	0	3.314001	0.999097	2.164050
18	6	0	3.412689	2.420024	2.671425
19	6	0	2.056339	0.589010	1.692988
20	6	0	1.586129	-0.608037	1.141566
21	6	0	0.231232	-0.614894	0.713725
22	6	0	-0.433537	-1.747435	0.289803
23	6	0	0.274386	-3.071231	0.190032
24	6	0	1.777197	-2.868677	0.013061
25	6	0	2.342814	-1.918624	1.074547
26	7	0	-1.753663	-1.754494	-0.005961
27	6	0	-2.633916	-0.608680	0.166944
28	6	0	-2.458597	0.422331	-0.937980
29	6	0	-2.101995	0.053851	-2.213452
30	6	0	-2.011487	1.031948	-3.232906
31	7	0	-2.221544	2.317433	-3.040516
32	6	0	-2.556125	2.717044	-1.778178
33	6	0	-2.709301	1.807199	-0.679846
34	6	0	-3.084048	2.316005	0.584722
35	6	0	-3.283244	3.673774	0.772750
36	6	0	-3.117505	4.577006	-0.308561
37	6	0	-2.764598	4.098846	-1.550073
38	8	0	-3.638272	4.057785	2.029477
39	6	0	-3.842169	5.442604	2.286030
40	6	0	-4.125916	-0.999232	0.282956
41	7	0	-4.465124	-1.517506	1.624277

42	6	0	-5.931569	-1.538446	1.731003
43	6	0	-6.583759	-2.525516	0.694319
44	6	0	-5.442800	-3.083363	-0.184712
45	6	0	-4.655542	-1.935306	-0.844656
46	6	0	-4.477413	-3.860071	0.729418
47	6	0	-3.968665	-2.889994	1.837722
48	6	0	-7.728462	-1.878196	-0.040589
49	6	0	-7.975028	-1.906581	-1.351379
50	1	0	-4.659059	-0.047280	0.212841
51	1	0	-2.369660	-0.157880	1.127168
52	1	0	3.395607	-1.756854	0.861525
53	1	0	2.291212	-2.388249	2.069928
54	1	0	5.272923	1.791748	0.371283
55	1	0	5.371424	0.672591	2.604984
56	1	0	4.524437	-0.785448	1.962500
57	1	0	1.298349	1.368939	1.739459
58	1	0	4.144180	2.508757	3.481245
59	1	0	3.729432	3.071266	1.847093
60	1	0	2.446986	2.789719	3.028248
61	1	0	7.586400	-3.028794	-0.949207
62	1	0	9.649088	-2.234653	0.215994
63	1	0	9.659651	-0.013527	1.343563
64	1	0	7.601528	1.380774	1.336421
65	1	0	-0.304312	0.326706	0.758923
66	1	0	2.295081	-3.831382	0.068831
67	1	0	1.978317	-2.446955	-0.975349
68	1	0	0.067228	-3.642953	1.107519
69	1	0	-0.142701	-3.652808	-0.642210
70	1	0	-2.142877	-2.606999	-0.381648
71	1	0	-4.323619	-3.205892	2.823666
72	1	0	-2.878485	-2.866679	1.886008
73	1	0	-5.285265	-1.367374	-1.532442
74	1	0	-3.847853	-2.359468	-1.454572
75	1	0	-4.984379	-4.725852	1.169937
76	1	0	-3.643949	-4.256686	0.133818
77	1	0	-5.847775	-3.751535	-0.951209
78	1	0	-6.294999	-0.518000	1.568783
79	1	0	-6.193893	-1.821569	2.755407
80	1	0	-8.424711	-1.337812	0.603936
81	1	0	-1.874992	-0.980802	-2.445715
82	1	0	-1.743577	0.726718	-4.243176
83	1	0	-2.636476	4.771761	-2.391522
84	1	0	-3.267065	5.640255	-0.166091
85	1	0	-3.231545	1.664694	1.438393
86	1	0	-8.843886	-1.406882	-1.770596
87	1	0	-7.335248	-2.429336	-2.056680
88	1	0	0.593983	2.076704	-1.303402
89	1	0	1.635409	2.933416	-2.464682
90	1	0	0.621298	1.560700	-3.013588
91	1	0	-4.110168	5.511007	3.341082
92	1	0	-2.929294	6.022707	2.104672
93	1	0	-4.659233	5.847665	1.676703
94	1	0	-7.004244	-3.371831	1.257960

INT4-*exo*

Center Number	Atomic Number	Atomic Type	Coordinates (Angstroms) X	Y	Z
1	6	0	-5.847627	-2.716672	-0.816288
2	6	0	-4.904736	-1.708424	-0.612341
3	6	0	-5.173448	-0.380831	-0.955106
4	6	0	-6.412454	-0.078542	-1.524650
5	6	0	-7.367387	-1.073612	-1.737395
6	6	0	-7.083273	-2.393171	-1.375009
7	8	0	-3.699936	-2.063121	-0.055057
8	6	0	-2.529954	-1.288317	-0.329948
9	6	0	-2.752054	0.058476	-0.697020
10	6	0	-4.132695	0.654680	-0.625860
11	8	0	-1.484021	-1.902804	-0.135404
12	6	0	-1.634828	0.864021	-1.102506
13	8	0	-0.448321	0.543928	-1.188261
14	8	0	-2.021702	2.153377	-1.417196
15	6	0	-0.952295	3.045401	-1.726718

16	6	0	-4.509653	1.260935	0.805842
17	6	0	-3.511482	2.265972	1.273973
18	6	0	-3.790313	3.694465	0.900821
19	6	0	-2.313780	1.923864	1.808317
20	6	0	-1.827061	0.593708	2.122623
21	6	0	-0.495906	0.327317	1.877245
22	6	0	0.034363	-0.967899	2.030906
23	6	0	-0.710389	-2.017127	2.812451
24	6	0	-1.809284	-1.383794	3.667189
25	6	0	-2.677222	-0.455306	2.808338
26	7	0	1.206863	-1.308296	1.512590
27	6	0	1.955945	-0.482759	0.557853
28	6	0	2.640167	0.679061	1.254419
29	6	0	3.081146	0.594242	2.554280
30	6	0	3.766084	1.685924	3.141326
31	7	0	4.019709	2.817906	2.519430
32	6	0	3.583780	2.936209	1.229302
33	6	0	2.881352	1.894639	0.536645
34	6	0	2.455691	2.112824	-0.793739
35	6	0	2.714167	3.319140	-1.423142
36	6	0	3.415219	4.349234	-0.746028
37	6	0	3.834952	4.152376	0.550775
38	8	0	2.249184	3.439045	-2.699540
39	6	0	2.459490	4.664418	-3.388736
40	6	0	3.005133	-1.287949	-0.253646
41	7	0	2.417136	-1.869987	-1.471822
42	6	0	3.509860	-2.413472	-2.286072
43	6	0	4.211830	-3.635839	-1.588662
44	6	0	3.646856	-3.717165	-0.154341
45	6	0	3.810225	-2.356575	0.553867
46	6	0	2.144815	-4.042247	-0.265384
47	6	0	1.469335	-2.953338	-1.154007
48	6	0	5.709866	-3.556322	-1.711431
49	6	0	6.616766	-3.715671	-0.745717
50	1	0	3.707941	-0.532302	-0.614398
51	1	0	1.214862	-0.113696	-0.155883
52	1	0	-3.435388	0.041372	3.421867
53	1	0	-3.211441	-1.064249	2.072409
54	1	0	-4.206703	1.481072	-1.335193
55	1	0	-5.500786	1.717292	0.708407
56	1	0	-4.608490	0.420969	1.492571
57	1	0	-1.560132	2.707994	1.883513
58	1	0	-4.718163	4.048599	1.368620
59	1	0	-2.975677	4.363366	1.193214
60	1	0	-3.926171	3.774326	-0.185444
61	1	0	-5.595135	-3.736145	-0.542684
62	1	0	-7.820166	-3.174871	-1.536760
63	1	0	-8.325943	-0.822287	-2.181840
64	1	0	-6.626860	0.952153	-1.798513
65	1	0	0.100553	1.083554	1.385673
66	1	0	-2.429961	-2.167304	4.112505
67	1	0	-1.358380	-0.813512	4.488353
68	1	0	-1.147468	-2.703844	2.078594
69	1	0	-0.003227	-2.586044	3.428801
70	1	0	1.508030	-2.264449	1.647351
71	1	0	1.148781	-3.382656	-2.108386
72	1	0	0.577186	-2.525471	-0.695688
73	1	0	4.863621	-2.070565	0.597841
74	1	0	3.481021	-2.442149	1.597348
75	1	0	2.006895	-5.040918	-0.695103
76	1	0	1.694394	-4.082352	0.737175
77	1	0	4.159476	-4.500757	0.412969
78	1	0	4.232674	-1.608431	-2.460883
79	1	0	3.100618	-2.705413	-3.258540
80	1	0	6.064525	-3.345033	-2.722409
81	1	0	2.901949	-0.300990	3.140386
82	1	0	4.115301	1.605759	4.169983
83	1	0	4.368748	4.928596	1.089480
84	1	0	3.619879	5.292656	-1.237598
85	1	0	1.889938	1.366658	-1.337901
86	1	0	7.680392	-3.640047	-0.954427
87	1	0	6.343350	-3.934136	0.282776
88	1	0	-0.312169	3.205541	-0.852901
89	1	0	-0.329711	2.663138	-2.537982
90	1	0	-1.427141	3.984585	-2.018165
91	1	0	1.999751	4.534468	-4.369192
92	1	0	1.979981	5.504458	-2.870928

93	1	0	3.528279	4.877118	-3.514339
94	1	0	3.896167	-4.548079	-2.116971

TS2-*exo*

Center Number	Atomic Number	Atomic Type	Coordinates (Angstroms) X	Y	Z
1	6	0	-5.759855	-2.656962	-1.586044
2	6	0	-4.860290	-1.753498	-1.021759
3	6	0	-5.106795	-0.384459	-0.994774
4	6	0	-6.291394	0.078407	-1.578438
5	6	0	-7.206875	-0.804088	-2.148928
6	6	0	-6.942132	-2.176774	-2.144384
7	8	0	-3.693742	-2.278217	-0.498208
8	6	0	-2.548579	-1.495604	-0.391359
9	6	0	-2.724423	-0.036792	-0.178763
10	6	0	-4.149456	0.541803	-0.291413
11	8	0	-1.501301	-2.099436	-0.358170
12	6	0	-1.677830	0.763661	-0.892797
13	8	0	-0.635072	0.344114	-1.357986
14	8	0	-1.992895	2.084012	-0.913445
15	6	0	-0.989614	2.953476	-1.461134
16	6	0	-4.775782	0.974507	1.077568
17	6	0	-3.850628	1.899110	1.813806
18	6	0	-4.332205	3.286801	2.123165
19	6	0	-2.593893	1.496212	2.049456
20	6	0	-2.089695	0.135507	1.716478
21	6	0	-0.638429	0.049415	1.661787
22	6	0	0.063887	-1.084473	1.939925
23	6	0	-0.576361	-2.236151	2.671535
24	6	0	-1.805046	-1.742296	3.434435
25	6	0	-2.778250	-1.019675	2.493757
26	7	0	1.383610	-1.232363	1.628779
27	6	0	2.043522	-0.390623	0.643381
28	6	0	2.552828	0.905599	1.251977
29	6	0	2.873779	1.007265	2.584447
30	6	0	3.379830	2.225485	3.099889
31	7	0	3.564582	3.313333	2.381086
32	6	0	3.239161	3.247747	1.054572
33	6	0	2.729956	2.063310	0.427760
34	6	0	2.410614	2.093779	-0.949530
35	6	0	2.571930	3.256745	-1.683625
36	6	0	3.082981	4.428453	-1.070240
37	6	0	3.407298	4.412512	0.268560
38	8	0	2.193924	3.194440	-2.993604
39	6	0	2.331829	4.360448	-3.794417
40	6	0	3.196721	-1.101793	-0.115644
41	7	0	2.690936	-1.792596	-1.317204
42	6	0	3.844319	-2.256771	-2.095723
43	6	0	4.657249	-3.369736	-1.338600
44	6	0	4.080947	-3.448086	0.091448
45	6	0	4.093322	-2.049477	0.742327
46	6	0	2.622762	-3.932373	-0.024565
47	6	0	1.853950	-2.953372	-0.962892
48	6	0	6.140894	-3.142193	-1.449954
49	6	0	7.045662	-3.168967	-0.469624
50	1	0	3.826429	-0.296054	-0.501643
51	1	0	1.289032	-0.148193	-0.111725
52	1	0	-3.600945	-0.605535	3.081572
53	1	0	-3.218053	-1.764429	1.827314
54	1	0	-4.079793	1.459197	-0.876531
55	1	0	-5.734157	1.461214	0.870230
56	1	0	-5.003753	0.073140	1.654690
57	1	0	-1.867210	2.192306	2.464555
58	1	0	-5.216955	3.265264	2.773557
59	1	0	-3.558360	3.884329	2.614892
60	1	0	-4.633198	3.805694	1.202163
61	1	0	-5.514950	-3.713901	-1.585750
62	1	0	-7.649441	-2.872563	-2.585800
63	1	0	-8.120967	-0.424784	-2.595604
64	1	0	-6.494117	1.146831	-1.576366
65	1	0	-0.117645	0.905840	1.249493
66	1	0	-2.321883	-2.582260	3.912112

67	1	0	-1.483910	-1.059358	4.230019
68	1	0	-0.855055	-3.014863	1.951924
69	1	0	0.154379	-2.678713	3.362008
70	1	0	1.817967	-2.106194	1.879614
71	1	0	1.583230	-3.448146	-1.901085
72	1	0	0.924675	-2.598320	-0.517702
73	1	0	5.112368	-1.657245	0.782836
74	1	0	3.758644	-2.121835	1.784048
75	1	0	2.597633	-4.954902	-0.418704
76	1	0	2.161103	-3.975755	0.971108
77	1	0	4.661615	-4.151271	0.697516
78	1	0	4.483417	-1.389760	-2.297830
79	1	0	3.481726	-2.630261	-3.059048
80	1	0	6.486502	-2.937187	-2.465471
81	1	0	2.725408	0.158827	3.243853
82	1	0	3.638123	2.291615	4.156278
83	1	0	3.796131	5.298829	0.759338
84	1	0	3.216431	5.338261	-1.643008
85	1	0	2.004897	1.225797	-1.455457
86	1	0	8.099023	-2.993873	-0.670754
87	1	0	6.780880	-3.371393	0.564352
88	1	0	-0.144554	3.031127	-0.773703
89	1	0	-0.627092	2.580823	-2.420772
90	1	0	-1.476026	3.922761	-1.575971
91	1	0	1.974464	4.082385	-4.786758
92	1	0	1.723660	5.188228	-3.408325
93	1	0	3.378739	4.681060	-3.862297
94	1	0	4.444940	-4.331119	-1.830488

INT5'

Center Number	Atomic Number	Atomic Type	Coordinates (Angstroms) X	Y	Z
1	6	0	2.947162	-2.452910	-1.705398
2	7	0	3.487899	-1.089116	-1.573067
3	6	0	3.606834	-0.695762	-0.152299
4	6	0	4.575148	-1.648903	0.614871
5	6	0	5.047897	-2.747606	-0.359524
6	6	0	3.814358	-3.482923	-0.918022
7	6	0	5.790540	-2.116262	-1.556667
8	6	0	4.823992	-1.043715	-2.178375
9	6	0	2.193933	-0.509182	0.469732
10	6	0	2.169003	0.712323	1.373229
11	6	0	2.137123	0.605573	2.742771
12	6	0	2.085614	1.773317	3.541574
13	7	0	2.057187	2.999685	3.061026
14	6	0	2.093513	3.137222	1.701161
15	6	0	2.157657	2.024793	0.799982
16	6	0	2.188236	2.267185	-0.593615
17	6	0	2.153158	3.561736	-1.086786
18	6	0	2.077720	4.664668	-0.197174
19	6	0	2.050950	4.445667	1.162544
20	8	0	2.200878	3.688693	-2.441655
21	6	0	2.098110	4.989314	-3.005569
22	6	0	7.149049	-1.535077	-1.267871
23	6	0	7.866984	-1.658959	-0.150185
24	7	0	1.697075	-1.690757	1.163329
25	6	0	0.316276	-1.908316	1.220740
26	6	0	-0.585075	-0.924493	1.061241
27	6	0	-2.085479	-1.105022	1.123180
28	6	0	-2.422643	-2.624095	1.007910
29	6	0	-1.529682	-3.449496	1.937347
30	6	0	-0.068452	-3.339281	1.500343
31	6	0	-2.624758	-0.602195	2.455629
32	6	0	-3.928908	-0.411839	2.672649
33	6	0	-4.500104	0.016588	3.993963
34	6	0	-4.869765	-0.578840	1.507193
35	6	0	-4.284835	0.104772	0.245227
36	6	0	-5.207160	-0.070043	-0.931775
37	6	0	-4.865104	-0.886555	-2.004053
38	8	0	-3.627077	-1.502626	-2.090835
39	6	0	-2.554285	-1.074432	-1.361211
40	6	0	-2.786424	-0.278184	-0.067375

41	6	0	-6.466100	0.543398	-0.960428
42	6	0	-7.354600	0.326747	-2.010550
43	6	0	-6.989484	-0.515955	-3.064380
44	6	0	-5.735886	-1.120515	-3.067201
45	8	0	-1.459342	-1.408729	-1.735513
46	6	0	-2.115039	1.079559	-0.335040
47	8	0	-1.861615	1.756276	0.795770
48	6	0	-1.295842	3.072044	0.624660
49	8	0	-1.928960	1.529074	-1.446053
50	1	0	4.058983	0.298397	-0.177022
51	1	0	1.523605	-0.300099	-0.375438
52	1	0	-3.472538	-2.801310	1.248276
53	1	0	-2.256863	-2.976812	-0.013988
54	1	0	-4.280366	1.169074	0.500842
55	1	0	-5.836824	-0.111243	1.721625
56	1	0	-5.086882	-1.632827	1.296452
57	1	0	-1.897244	-0.446191	3.247595
58	1	0	-5.241993	-0.707467	4.358014
59	1	0	-3.720553	0.118413	4.755365
60	1	0	-5.021347	0.979934	3.904917
61	1	0	-5.411162	-1.762225	-3.879220
62	1	0	-7.673725	-0.692504	-3.888688
63	1	0	-8.324928	0.813654	-2.009451
64	1	0	-6.747084	1.199341	-0.140180
65	1	0	-0.235450	0.089417	0.933878
66	1	0	-1.847533	-4.498070	1.925894
67	1	0	-1.638765	-3.091900	2.968295
68	1	0	0.099297	-3.940898	0.593495
69	1	0	0.597988	-3.749028	2.271336
70	1	0	2.216685	-2.534935	0.971017
71	1	0	2.932167	-2.691791	-2.773284
72	1	0	1.906544	-2.451489	-1.370792
73	1	0	5.429865	-1.080651	0.989466
74	1	0	4.101236	-2.104602	1.490436
75	1	0	4.129704	-4.305686	-1.569840
76	1	0	3.248256	-3.942821	-0.096610
77	1	0	5.699567	-3.457735	0.159840
78	1	0	5.223550	-0.035072	-2.025526
79	1	0	4.717745	-1.195561	-3.257559
80	1	0	7.571356	-0.955081	-2.091182
81	1	0	2.123195	-0.376403	3.202027
82	1	0	2.057235	1.676943	4.626448
83	1	0	1.996529	5.275333	1.860193
84	1	0	2.044160	5.678407	-0.577559
85	1	0	2.247185	1.453422	-1.307961
86	1	0	8.846558	-1.198226	-0.056612
87	1	0	7.517367	-2.224046	0.709224
88	1	0	-1.002815	3.391004	1.623448
89	1	0	-0.429357	3.028334	-0.034895
90	1	0	-2.045135	3.747678	0.203723
91	1	0	2.125906	4.846345	-4.086510
92	1	0	1.153617	5.473341	-2.727867
93	1	0	2.937362	5.627855	-2.702443
94	1	0	5.936913	-2.908457	-2.306596

INT2-A

Center Number	Atomic Number	Atomic Type	Coordinates (Angstroms) X	Y	Z
1	6	0	3.016652	6.306764	-2.118811
2	6	0	3.501735	5.379832	-1.269764
3	6	0	4.765263	5.658447	-0.481869
4	6	0	2.925135	4.053073	-1.046077
5	6	0	1.623234	3.657065	-1.074883
6	6	0	1.292045	2.266133	-0.821293
7	6	0	0.019811	1.786443	-0.797253
8	6	0	-1.170366	2.708592	-0.919383
9	6	0	-0.792498	4.148098	-0.571150
10	6	0	0.461302	4.591249	-1.334472
11	7	0	-0.326176	0.444927	-0.697680
12	6	0	0.691869	-0.546585	-0.419193
13	6	0	1.221966	-0.489761	1.016315
14	6	0	0.383530	-0.175634	2.064634

15	6	0	0.893473	-0.102403	3.382523
16	7	0	2.157032	-0.295355	3.700711
17	6	0	3.020531	-0.570071	2.680518
18	6	0	2.612195	-0.680365	1.308389
19	6	0	3.599684	-0.950784	0.331363
20	6	0	4.929785	-1.108970	0.679414
21	6	0	5.330558	-1.007568	2.036181
22	6	0	4.388532	-0.744521	3.003736
23	8	0	5.790240	-1.365691	-0.345511
24	6	0	7.175247	-1.501884	-0.050248
25	6	0	0.239721	-1.931401	-0.956816
26	7	0	-1.155255	-2.374381	-0.572851
27	6	0	-1.219554	-2.967080	0.804017
28	6	0	-0.422385	-4.289906	0.824735
29	6	0	0.389235	-4.399929	-0.479886
30	6	0	1.196709	-3.099249	-0.636703
31	6	0	-0.604737	-4.574575	-1.648144
32	6	0	-1.616900	-3.399664	-1.569808
33	6	0	0.014839	-4.693908	-3.021501
34	6	0	1.271243	-5.038649	-3.304502
35	8	0	-3.353759	-1.059142	-0.788599
36	6	0	-3.551144	-0.224340	0.156259
37	8	0	-2.647966	0.191495	0.932743
38	6	0	-4.948021	0.301693	0.331571
39	6	0	-5.985072	-0.120535	-0.507415
40	6	0	-7.275508	0.374147	-0.336574
41	6	0	-7.564570	1.302623	0.672856
42	6	0	-6.520757	1.716723	1.512200
43	6	0	-5.229149	1.225583	1.344368
44	6	0	-8.956290	1.862700	0.835529
45	1	0	0.169082	-1.797730	-2.038831
46	1	0	1.532279	-0.312604	-1.078368
47	1	0	-1.941018	-1.611592	-0.647341
48	1	0	0.736538	5.615261	-1.065026
49	1	0	0.234252	4.605333	-2.411754
50	1	0	3.496954	7.276425	-2.221918
51	1	0	2.154478	6.122893	-2.749815
52	1	0	3.653313	3.287352	-0.771114
53	1	0	5.530992	4.900751	-0.696162
54	1	0	5.183665	6.641375	-0.718728
55	1	0	4.574549	5.613787	0.598071
56	1	0	2.133286	1.599210	-0.656150
57	1	0	-1.627834	4.821759	-0.793453
58	1	0	-0.599332	4.217151	0.506933
59	1	0	-1.565727	2.652751	-1.944783
60	1	0	-1.969921	2.346774	-0.262277
61	1	0	-1.201380	0.307506	-0.176212
62	1	0	-2.279259	-3.097108	1.029521
63	1	0	-0.818163	-2.227469	1.491411
64	1	0	1.941663	-3.175756	-1.430584
65	1	0	1.734912	-2.912278	0.295018
66	1	0	-1.095783	-5.147580	0.924459
67	1	0	0.250413	-4.300541	1.687242
68	1	0	1.062897	-5.259181	-0.424631
69	1	0	-1.731898	-2.878515	-2.522373
70	1	0	-2.608399	-3.713070	-1.241029
71	1	0	-0.672362	-4.506708	-3.847777
72	1	0	-0.669143	0.026253	1.892278
73	1	0	0.214712	0.130156	4.201695
74	1	0	4.669888	-0.656722	4.047964
75	1	0	6.369509	-1.131069	2.316385
76	1	0	3.348165	-1.044910	-0.717799
77	1	0	-8.073851	0.037127	-0.993970
78	1	0	-6.727036	2.432156	2.305077
79	1	0	-4.420020	1.544350	1.992640
80	1	0	-5.762148	-0.838715	-1.289122
81	1	0	-9.077449	2.791119	0.261666
82	1	0	-9.715438	1.158833	0.479108
83	1	0	-9.172757	2.098944	1.882673
84	1	0	1.608914	-5.128477	-4.332806
85	1	0	2.008121	-5.254814	-2.536102
86	1	0	7.665611	-1.681835	-1.007769
87	1	0	7.581123	-0.587788	0.399643
88	1	0	7.362217	-2.351094	0.618519
89	1	0	-1.170010	-5.499615	-1.467918

INT3-A

Center Number	Atomic Number	Atomic Type	Coordinates (Angstroms) X	Y	Z
1	6	0	5.837032	-0.396241	1.112408
2	6	0	4.532744	-0.738691	1.463741
3	6	0	4.135781	-2.077628	1.618912
4	6	0	5.099367	-3.090779	1.443967
5	6	0	6.402539	-2.761445	1.105870
6	6	0	6.766776	-1.415055	0.933907
7	8	0	3.642897	0.276057	1.651731
8	6	0	2.314138	0.083631	2.007538
9	6	0	1.868433	-1.304133	2.113481
10	6	0	2.759621	-2.323431	1.913710
11	6	0	0.459253	-1.687066	2.414521
12	8	0	0.090686	-2.850516	2.438703
13	8	0	1.665600	1.100172	2.161391
14	8	0	-0.338589	-0.643923	2.687199
15	6	0	-1.721441	-0.925989	2.982694
16	6	0	3.098480	-5.459737	-0.450532
17	6	0	3.374751	-4.315741	-1.110537
18	6	0	4.634127	-4.205418	-1.943461
19	6	0	2.574139	-3.095318	-1.058132
20	6	0	1.227767	-2.940368	-0.892376
21	6	0	0.676551	-1.607875	-0.836067
22	6	0	-0.647905	-1.351665	-0.617986
23	6	0	-1.660811	-2.469319	-0.580392
24	6	0	-1.131815	-3.720471	-1.279912
25	6	0	0.257149	-4.091402	-0.747400
26	7	0	-1.171318	-0.108661	-0.358568
27	6	0	-0.333792	1.070877	-0.366167
28	6	0	-0.028276	1.568025	-1.776905
29	6	0	-0.981452	1.511478	-2.770925
30	6	0	-0.658538	1.921857	-4.086017
31	7	0	0.534034	2.338631	-4.458800
32	6	0	1.510607	2.370500	-3.505279
33	6	0	1.288809	2.007359	-2.134187
34	6	0	2.375798	2.073552	-1.227835
35	6	0	3.635240	2.457255	-1.655994
36	6	0	3.855344	2.811274	-3.011938
37	6	0	2.808814	2.771081	-3.904315
38	8	0	4.615243	2.475405	-0.706736
39	6	0	5.922118	2.877848	-1.093374
40	6	0	-0.828309	2.101444	0.680671
41	7	0	-2.319554	2.357521	0.706567
42	6	0	-2.759558	3.320325	-0.354938
43	6	0	-2.171113	4.714398	-0.042378
44	6	0	-1.106663	4.568404	1.062694
45	6	0	-0.123577	3.470007	0.622040
46	6	0	-1.828778	4.169730	2.368284
47	6	0	-2.679324	2.908962	2.056198
48	6	0	-0.938304	3.960459	3.571176
49	6	0	0.301714	4.418896	3.737692
50	8	0	-4.122564	0.481224	0.888233
51	6	0	-4.455971	0.029585	-0.258439
52	8	0	-3.799383	0.235891	-1.317237
53	6	0	-5.689786	-0.824862	-0.332657
54	6	0	-6.455860	-1.079105	0.810310
55	6	0	-7.594212	-1.877057	0.732574
56	6	0	-7.996224	-2.443789	-0.484897
57	6	0	-7.225706	-2.180378	-1.626027
58	6	0	-6.087070	-1.382795	-1.552967
59	6	0	-9.210600	-3.336308	-0.560246
60	1	0	-0.622834	1.627840	1.640577
61	1	0	0.612326	0.768983	0.076121
62	1	0	-2.928305	1.462214	0.646904
63	1	0	0.646266	-4.975375	-1.261916
64	1	0	0.170235	-4.352513	0.316033
65	1	0	2.404935	-3.344299	1.996526
66	1	0	3.743902	-6.328897	-0.547102
67	1	0	2.241924	-5.562188	0.205773
68	1	0	3.143885	-2.179626	-1.221364
69	1	0	5.266825	-3.389166	-1.572022
70	1	0	5.220140	-5.129199	-1.917940

71	1	0	4.395672	-3.974086	-2.989240
72	1	0	6.089852	0.649430	0.984402
73	1	0	7.787154	-1.163600	0.660727
74	1	0	7.141163	-3.543871	0.965233
75	1	0	4.792681	-4.125745	1.545584
76	1	0	1.381514	-0.789709	-0.944202
77	1	0	-1.826549	-4.554681	-1.132001
78	1	0	-1.071450	-3.535654	-2.360067
79	1	0	-1.886167	-2.697150	0.470716
80	1	0	-2.595586	-2.123087	-1.036429
81	1	0	-2.139897	0.023217	-0.677254
82	1	0	-3.850647	3.302488	-0.358549
83	1	0	-2.414752	2.926317	-1.307205
84	1	0	0.762746	3.424176	1.254644
85	1	0	0.209656	3.692744	-0.394997
86	1	0	-2.959093	5.403775	0.279115
87	1	0	-1.719060	5.132936	-0.946416
88	1	0	-0.581560	5.517964	1.197614
89	1	0	-2.513689	2.104380	2.774795
90	1	0	-3.750392	3.114425	2.024149
91	1	0	-1.398032	3.395826	4.383481
92	1	0	-1.976851	1.129793	-2.563663
93	1	0	-1.426812	1.889307	-4.857214
94	1	0	2.952021	3.038690	-4.946136
95	1	0	4.838290	3.113191	-3.352322
96	1	0	2.257871	1.844291	-0.176322
97	1	0	-8.183011	-2.065671	1.627474
98	1	0	-7.525307	-2.606682	-2.580754
99	1	0	-5.489635	-1.176679	-2.434572
100	1	0	-6.144494	-0.641428	1.752662
101	1	0	-8.937429	-4.388051	-0.401538
102	1	0	-9.948752	-3.073564	0.204584
103	1	0	-9.694709	-3.271622	-1.540288
104	1	0	0.849345	4.230285	4.656136
105	1	0	0.822765	4.993098	2.977233
106	1	0	-1.835355	-1.953058	3.329000
107	1	0	-2.009316	-0.221518	3.765645
108	1	0	-2.319942	-0.750842	2.086728
109	1	0	6.526364	2.838139	-0.185678
110	1	0	6.347659	2.198562	-1.842420
111	1	0	5.929970	3.902373	-1.485116
112	1	0	-2.530821	4.977313	2.619561

INT3'-A

Center Number	Atomic Number	Atomic Type	Coordinates (Angstroms) X	Y	Z
1	6	0	-7.480849	-1.369926	-0.670010
2	6	0	-6.296587	-0.794355	-0.207366
3	6	0	-5.471986	-1.459951	0.715291
4	6	0	-5.861265	-2.732430	1.173768
5	6	0	-7.034485	-3.313794	0.719847
6	6	0	-7.842038	-2.629349	-0.203464
7	8	0	-5.968493	0.440500	-0.678296
8	6	0	-4.797178	1.119287	-0.324635
9	6	0	-3.905115	0.408348	0.595563
10	6	0	-4.252921	-0.815144	1.092590
11	6	0	-2.589010	0.954394	1.005405
12	8	0	-1.769030	0.289391	1.631923
13	8	0	-4.658130	2.218269	-0.807742
14	8	0	-2.382580	2.223106	0.655363
15	6	0	-1.125940	2.794155	1.063878
16	6	0	-2.429576	-2.579684	-0.454035
17	6	0	-3.087975	-2.155177	-1.557838
18	6	0	-4.119005	-3.063203	-2.195953
19	6	0	-2.976028	-0.840647	-2.175969
20	6	0	-1.932900	0.049832	-2.181412
21	6	0	-0.639683	-0.209148	-1.613027
22	6	0	0.395722	0.689146	-1.645717
23	6	0	0.230464	2.047523	-2.288614
24	6	0	-0.839318	1.994610	-3.382523
25	6	0	-2.139411	1.406923	-2.829126
26	7	0	1.643731	0.438535	-1.151078

27	6	0	2.004550	-0.791772	-0.477178
28	6	0	3.466327	-1.144074	-0.723546
29	6	0	4.440687	-0.167372	-0.765935
30	6	0	5.793954	-0.524347	-0.971541
31	7	0	6.217506	-1.760174	-1.137988
32	6	0	5.274670	-2.745775	-1.142538
33	6	0	3.870976	-2.500749	-0.955391
34	6	0	2.983702	-3.601437	-1.029641
35	6	0	3.447598	-4.886481	-1.250649
36	6	0	4.836095	-5.129125	-1.408405
37	6	0	5.717192	-4.074461	-1.356596
38	8	0	2.499611	-5.863072	-1.301757
39	6	0	2.914745	-7.198753	-1.561872
40	6	0	1.505065	-0.800690	0.999673
41	7	0	2.100069	0.264010	1.896766
42	6	0	3.478163	-0.044061	2.408183
43	6	0	3.454688	-1.367451	3.202641
44	6	0	2.022216	-1.923635	3.199081
45	6	0	1.608903	-2.155193	1.736738
46	6	0	1.093891	-0.890838	3.887490
47	6	0	1.179870	0.433851	3.078820
48	6	0	-0.324368	-1.378931	4.019681
49	6	0	-0.869697	-1.773521	5.170160
50	8	0	2.026026	2.765905	1.248033
51	6	0	2.444154	3.335616	0.185079
52	8	0	3.118366	2.780893	-0.723266
53	6	0	2.046699	4.775519	-0.003590
54	6	0	1.333867	5.460629	0.987598
55	6	0	0.939937	6.781319	0.789549
56	6	0	1.244185	7.453511	-0.402503
57	6	0	1.966802	6.764256	-1.385949
58	6	0	2.363639	5.443874	-1.191270
59	6	0	0.783003	8.871851	-0.630776
60	1	0	0.456387	-0.505081	0.927629
61	1	0	1.409498	-1.582465	-0.939873
62	1	0	2.127184	1.245628	1.426849
63	1	0	-2.900253	1.333101	-3.613709
64	1	0	-2.547597	2.087720	-2.070160
65	1	0	-3.586422	-1.301565	1.794535
66	1	0	-1.752422	-1.947999	0.106352
67	1	0	-2.600394	-3.578475	-0.061126
68	1	0	-3.850227	-0.548039	-2.757401
69	1	0	-3.880229	-3.257997	-3.248891
70	1	0	-4.194790	-4.021049	-1.673517
71	1	0	-5.109523	-2.590414	-2.176017
72	1	0	-8.090952	-0.825692	-1.382449
73	1	0	-8.759698	-3.086426	-0.560879
74	1	0	-7.328189	-4.297015	1.072865
75	1	0	-5.217440	-3.253270	1.876565
76	1	0	-0.483407	-1.184211	-1.177304
77	1	0	-1.016865	3.000451	-3.778791
78	1	0	-0.474803	1.376110	-4.212822
79	1	0	-0.051904	2.787732	-1.528852
80	1	0	1.194608	2.378249	-2.687316
81	1	0	2.280672	1.240792	-1.079628
82	1	0	3.761082	0.814301	3.020282
83	1	0	4.149357	-0.083603	1.556801
84	1	0	0.649023	-2.675778	1.670664
85	1	0	2.355181	-2.798981	1.266738
86	1	0	3.796693	-1.202267	4.229251
87	1	0	4.134634	-2.091245	2.742253
88	1	0	1.981946	-2.866939	3.750703
89	1	0	0.209248	0.729593	2.682396
90	1	0	1.584897	1.258647	3.666988
91	1	0	-0.918885	-1.390492	3.108682
92	1	0	4.193804	0.883113	-0.634874
93	1	0	6.550867	0.258127	-0.988750
94	1	0	6.783153	-4.229280	-1.487271
95	1	0	5.206668	-6.132576	-1.578537
96	1	0	1.912757	-3.477082	-0.927176
97	1	0	0.388723	7.302514	1.568997
98	1	0	2.219006	7.271427	-2.314413
99	1	0	2.922954	4.910213	-1.952089
100	1	0	1.100277	4.942858	1.911554
101	1	0	-0.214793	8.892657	-1.088865
102	1	0	0.719696	9.428627	0.309961
103	1	0	1.460054	9.409603	-1.302484

104	1	0	-1.893456	-2.133688	5.222858
105	1	0	-0.314198	-1.748412	6.105943
106	1	0	-1.068150	2.840740	2.154279
107	1	0	-0.288759	2.212220	0.685932
108	1	0	-1.110523	3.797473	0.641904
109	1	0	2.001634	-7.795135	-1.573797
110	1	0	3.415221	-7.280905	-2.534256
111	1	0	3.582107	-7.572443	-0.775563
112	1	0	1.492674	-0.718956	4.894047

TS1-A

Center Number	Atomic Number	Atomic Type	Coordinates (Angstroms) X	Y	Z
1	6	0	6.355516	1.644687	0.719802
2	6	0	5.408057	0.635494	0.545899
3	6	0	5.787497	-0.679179	0.249296
4	6	0	7.155593	-0.971236	0.151160
5	6	0	8.112464	0.023832	0.325297
6	6	0	7.708220	1.335492	0.603284
7	8	0	4.090229	0.984406	0.675893
8	6	0	3.060154	0.032056	0.738979
9	6	0	3.416168	-1.327190	0.465880
10	6	0	4.729706	-1.659471	0.014135
11	6	0	2.484214	-2.440108	0.568405
12	8	0	2.784062	-3.589826	0.242589
13	8	0	1.969017	0.504891	1.038548
14	8	0	1.244075	-2.146792	1.036147
15	6	0	0.376177	-3.282898	1.196060
16	6	0	4.677947	-1.890023	-2.042541
17	6	0	4.310278	-0.606290	-2.493120
18	6	0	5.419883	0.348732	-2.869806
19	6	0	3.010750	-0.091519	-2.407950
20	6	0	1.781434	-0.763599	-2.284742
21	6	0	0.653572	-0.020547	-1.880735
22	6	0	-0.611377	-0.575632	-1.733978
23	6	0	-0.866190	-2.003307	-2.150901
24	6	0	0.166413	-2.472118	-3.173070
25	6	0	1.588659	-2.216359	-2.662692
26	7	0	-1.671199	0.074278	-1.220534
27	6	0	-1.589040	1.378397	-0.572006
28	6	0	-2.891471	2.153475	-0.707296
29	6	0	-4.117608	1.522644	-0.657596
30	6	0	-5.309127	2.280409	-0.747825
31	7	0	-5.345884	3.588996	-0.886260
32	6	0	-4.150553	4.239034	-0.982432
33	6	0	-2.878687	3.573153	-0.916941
34	6	0	-1.707607	4.350539	-1.082621
35	6	0	-1.773008	5.719691	-1.276285
36	6	0	-3.028585	6.378157	-1.311544
37	6	0	-4.182028	5.642813	-1.170137
38	8	0	-0.580317	6.358523	-1.425420
39	6	0	-0.584531	7.766047	-1.635393
40	6	0	-0.976545	1.214255	0.847614
41	7	0	-1.767490	0.342675	1.798358
42	6	0	-2.980701	0.987003	2.409150
43	6	0	-2.584065	2.329298	3.052305
44	6	0	-1.050465	2.435043	3.055761
45	6	0	-0.586984	2.512045	1.591918
46	6	0	-0.475932	1.183744	3.771979
47	6	0	-0.828244	-0.059426	2.908976
48	6	0	1.003614	1.275248	4.028481
49	6	0	1.539090	1.363375	5.246126
50	8	0	-2.645603	-2.038657	1.211357
51	6	0	-3.313933	-2.426645	0.198554
52	8	0	-3.751889	-1.673001	-0.715788
53	6	0	-3.551596	-3.906018	0.068525
54	6	0	-3.114496	-4.794711	1.058051
55	6	0	-3.312041	-6.165069	0.913015
56	6	0	-3.948240	-6.686274	-0.222304
57	6	0	-4.390863	-5.789559	-1.204422
58	6	0	-4.196044	-4.418265	-1.063110
59	6	0	-4.128214	-8.174475	-0.393529

60	1	0	-0.067980	0.637431	0.678791
61	1	0	-0.827688	1.946453	-1.107755
62	1	0	-2.105126	-0.575855	1.348733
63	1	0	2.322685	-2.493178	-3.425303
64	1	0	1.779207	-2.865576	-1.800414
65	1	0	5.003357	-2.704078	0.120207
66	1	0	5.697439	-2.218484	-2.234442
67	1	0	3.952499	-2.690249	-1.988634
68	1	0	2.939779	0.995449	-2.387019
69	1	0	6.334099	0.150147	-2.303558
70	1	0	5.664833	0.235919	-3.934971
71	1	0	5.130475	1.391472	-2.707647
72	1	0	6.017351	2.650407	0.946528
73	1	0	8.449882	2.117542	0.736188
74	1	0	9.168116	-0.216370	0.244489
75	1	0	7.457255	-1.990467	-0.076210
76	1	0	0.831549	1.006482	-1.584899
77	1	0	0.030354	-3.539265	-3.376468
78	1	0	0.011950	-1.937132	-4.118322
79	1	0	-0.818818	-2.630076	-1.250849
80	1	0	-1.887398	-2.097433	-2.530805
81	1	0	-2.524901	-0.493251	-1.073373
82	1	0	-3.355016	0.264543	3.136968
83	1	0	-3.731280	1.097400	1.633008
84	1	0	0.496619	2.633152	1.518709
85	1	0	-1.051909	3.387108	1.133829
86	1	0	-2.981137	2.388386	4.070400
87	1	0	-3.010825	3.161721	2.483063
88	1	0	-0.734111	3.334025	3.591930
89	1	0	0.053684	-0.491894	2.434113
90	1	0	-1.338021	-0.835772	3.481882
91	1	0	1.641079	1.259148	3.146293
92	1	0	-4.197406	0.444951	-0.544068
93	1	0	-6.267679	1.767072	-0.692930
94	1	0	-5.156407	6.118559	-1.207446
95	1	0	-3.087630	7.449599	-1.458394
96	1	0	-0.720586	3.905182	-1.070334
97	1	0	-2.969520	-6.844200	1.690414
98	1	0	-4.893872	-6.174313	-2.088629
99	1	0	-4.539163	-3.723472	-1.822022
100	1	0	-2.625891	-4.395106	1.939906
101	1	0	-3.252880	-8.623582	-0.881412
102	1	0	-4.253937	-8.676100	0.571652
103	1	0	-5.000052	-8.403288	-1.014962
104	1	0	2.613246	1.438709	5.391960
105	1	0	0.924838	1.361547	6.145158
106	1	0	0.346553	-3.880729	0.282433
107	1	0	0.730598	-3.918808	2.013261
108	1	0	-0.610519	-2.875995	1.411967
109	1	0	0.463216	8.053401	-1.730643
110	1	0	-1.119740	8.032858	-2.554693
111	1	0	-1.030869	8.296998	-0.785752
112	1	0	-0.988275	1.102546	4.738458

TS1’-A

Center Number	Atomic Number	Atomic Type	Coordinates (Angstroms) X	Y	Z
1	6	0	-7.554660	-0.363996	-0.363763
2	6	0	-6.206488	-0.058252	-0.166963
3	6	0	-5.408041	-0.821293	0.694504
4	6	0	-5.999026	-1.892046	1.380610
5	6	0	-7.342045	-2.205146	1.196945
6	6	0	-8.117155	-1.441174	0.315366
7	8	0	-5.701825	1.012939	-0.848149
8	6	0	-4.427631	1.558455	-0.575701
9	6	0	-3.585664	0.797510	0.312216
10	6	0	-3.988403	-0.484737	0.792173
11	6	0	-2.284739	1.264913	0.741661
12	8	0	-1.498206	0.561082	1.392828
13	8	0	-4.203192	2.616664	-1.135168
14	8	0	-1.974357	2.534017	0.410172
15	6	0	-0.772840	3.058064	0.991511

16	6	0	-3.026984	-1.945017	-0.295183
17	6	0	-3.489706	-1.730269	-1.611149
18	6	0	-4.681673	-2.544335	-2.061276
19	6	0	-3.068908	-0.685689	-2.444519
20	6	0	-1.912836	0.117662	-2.381999
21	6	0	-0.724506	-0.251210	-1.722945
22	6	0	0.393152	0.572182	-1.666331
23	6	0	0.356028	1.944875	-2.293125
24	6	0	-0.558331	1.936879	-3.519820
25	6	0	-1.947275	1.425677	-3.138149
26	7	0	1.556466	0.226594	-1.086726
27	6	0	1.803123	-1.044437	-0.419589
28	6	0	3.211426	-1.550290	-0.703800
29	6	0	4.286379	-0.687884	-0.775118
30	6	0	5.585572	-1.195409	-1.013402
31	7	0	5.862637	-2.471252	-1.184126
32	6	0	4.816718	-3.346014	-1.155651
33	6	0	3.455102	-2.945588	-0.931112
34	6	0	2.450169	-3.941508	-0.964134
35	6	0	2.761458	-5.271862	-1.186082
36	6	0	4.108848	-5.667479	-1.385783
37	6	0	5.102373	-4.716766	-1.371221
38	8	0	1.710302	-6.136521	-1.195586
39	6	0	1.965805	-7.514854	-1.442674
40	6	0	1.344209	-0.981790	1.065071
41	7	0	2.092307	0.003505	1.936772
42	6	0	3.405825	-0.508480	2.457021
43	6	0	3.161840	-1.737664	3.358379
44	6	0	1.672801	-2.112026	3.288924
45	6	0	1.307055	-2.332422	1.811087
46	6	0	0.851487	-0.948175	3.900946
47	6	0	1.202767	0.342502	3.108636
48	6	0	-0.629780	-1.225623	3.916501
49	6	0	-1.299604	-1.601909	5.005792
50	8	0	2.457626	2.471353	1.242924
51	6	0	2.835387	2.997349	0.145845
52	8	0	3.286288	2.360783	-0.848204
53	6	0	2.679748	4.487405	0.021786
54	6	0	2.154787	5.241820	1.077814
55	6	0	1.977614	6.616080	0.943396
56	6	0	2.319670	7.274434	-0.246150
57	6	0	2.854400	6.513642	-1.294939
58	6	0	3.031033	5.138727	-1.165788
59	6	0	2.092336	8.757634	-0.402977
60	1	0	0.337750	-0.561107	1.018678
61	1	0	1.116136	-1.765826	-0.862745
62	1	0	2.275242	0.952111	1.445540
63	1	0	-2.586316	1.320352	-4.021984
64	1	0	-2.452157	2.155808	-2.486982
65	1	0	-3.479077	-0.825626	1.688394
66	1	0	-2.053107	-1.598947	0.019872
67	1	0	-3.374814	-2.839134	0.217842
68	1	0	-3.760373	-0.423271	-3.243136
69	1	0	-4.346567	-3.514739	-2.452829
70	1	0	-5.365035	-2.749212	-1.231787
71	1	0	-5.240319	-2.041726	-2.856142
72	1	0	-8.137859	0.250865	-1.041200
73	1	0	-9.165202	-1.681832	0.163200
74	1	0	-7.785816	-3.038125	1.733533
75	1	0	-5.383703	-2.485052	2.052882
76	1	0	-0.687702	-1.227563	-1.263815
77	1	0	-0.628794	2.946972	-3.935941
78	1	0	-0.121055	1.293609	-4.294355
79	1	0	-0.032703	2.658151	-1.556866
80	1	0	1.371331	2.267447	-2.537430
81	1	0	2.287712	0.958144	-1.040862
82	1	0	3.861143	0.329336	2.988315
83	1	0	4.030540	-0.742133	1.600525
84	1	0	0.309587	-2.769467	1.707232
85	1	0	2.017306	-3.041696	1.379684
86	1	0	3.448591	-1.520655	4.392313
87	1	0	3.776999	-2.575739	3.016478
88	1	0	1.483576	-3.024808	3.860162
89	1	0	0.315875	0.822662	2.696427
90	1	0	1.753663	1.066738	3.710300
91	1	0	-1.157790	-1.091724	2.974971
92	1	0	4.163126	0.384659	-0.649730

93	1	0	6.424496	-0.502885	-1.055348
94	1	0	6.139898	-4.989767	-1.532663
95	1	0	4.360744	-6.706764	-1.557588
96	1	0	1.403791	-3.700777	-0.823393
97	1	0	1.568332	7.190607	1.771167
98	1	0	3.132724	7.007905	-2.222850
99	1	0	3.442872	4.550053	-1.978278
100	1	0	1.891283	4.733540	1.998959
101	1	0	1.091693	8.961580	-0.806598
102	1	0	2.167235	9.277935	0.557471
103	1	0	2.817396	9.203035	-1.091960
104	1	0	-2.366101	-1.807777	4.976658
105	1	0	-0.809202	-1.713253	5.971369
106	1	0	-0.869691	3.116704	2.080321
107	1	0	0.098852	2.451475	0.750738
108	1	0	-0.658367	4.058728	0.574943
109	1	0	0.991954	-8.005042	-1.415832
110	1	0	2.422588	-7.667164	-2.427861
111	1	0	2.611895	-7.947939	-0.669417
112	1	0	1.189585	-0.827927	4.936599

TS1-A-*OF*

Center Number	Atomic Number	Atomic Type	Coordinates (Angstroms) X	Y	Z
1	6	0	6.287635	0.733523	0.056570
2	6	0	5.473482	-0.365244	-0.229524
3	6	0	5.935854	-1.674578	-0.037218
4	6	0	7.248347	-1.860895	0.418783
5	6	0	8.071376	-0.774191	0.699614
6	6	0	7.582216	0.525946	0.525319
7	8	0	4.222864	-0.106255	-0.714371
8	6	0	3.403586	-1.121002	-1.274207
9	6	0	3.863704	-2.473482	-1.094033
10	6	0	5.012663	-2.778284	-0.306141
11	6	0	3.209886	-3.618871	-1.712603
12	8	0	3.405420	-4.781988	-1.358400
13	8	0	2.400597	-0.713007	-1.828446
14	8	0	2.385422	-3.324857	-2.748363
15	6	0	1.795019	-4.458676	-3.393769
16	6	0	4.415337	-3.472971	1.532260
17	6	0	3.942008	-2.318249	2.187269
18	6	0	4.910884	-1.631978	3.126884
19	6	0	2.756000	-1.630092	1.897135
20	6	0	1.599463	-1.979491	1.178896
21	6	0	0.750391	-0.921445	0.787321
22	6	0	-0.456225	-1.100136	0.124389
23	6	0	-1.015789	-2.487380	-0.057635
24	6	0	0.123365	-3.490539	-0.221339
25	6	0	1.131354	-3.397627	0.927148
26	7	0	-1.208256	-0.098425	-0.364508
27	6	0	-0.888050	1.306873	-0.173747
28	6	0	-1.428619	2.189477	-1.287077
29	6	0	-2.614694	1.912174	-1.933330
30	6	0	-3.093093	2.788030	-2.937765
31	7	0	-2.476552	3.886340	-3.319512
32	6	0	-1.286011	4.174320	-2.718254
33	6	0	-0.697520	3.354570	-1.696364
34	6	0	0.554803	3.740173	-1.163974
35	6	0	1.197184	4.887214	-1.596542
36	6	0	0.608350	5.702922	-2.596129
37	6	0	-0.603215	5.341960	-3.138185
38	8	0	2.391154	5.162474	-1.003051
39	6	0	3.121731	6.301168	-1.443374
40	6	0	-1.274121	1.713950	1.276531
41	7	0	-2.721109	1.440522	1.622842
42	6	0	-3.632299	2.586695	1.276768
43	6	0	-3.310504	3.783276	2.196682
44	6	0	-1.941945	3.528498	2.856476
45	6	0	-0.957664	3.146819	1.730649
46	6	0	-2.114439	2.364552	3.862060
47	6	0	-2.799972	1.194282	3.101964
48	6	0	-0.853369	1.918865	4.566899

49	6	0	0.262100	2.630341	4.731879
50	8	0	-4.147529	-0.607846	1.040365
51	6	0	-4.398094	-1.131074	-0.095544
52	8	0	-3.721026	-0.947164	-1.145478
53	6	0	-5.592607	-2.040224	-0.180214
54	6	0	-6.425472	-2.239358	0.926092
55	6	0	-7.537958	-3.072111	0.830536
56	6	0	-7.844873	-3.731083	-0.367639
57	6	0	-7.004732	-3.526243	-1.471810
58	6	0	-5.894012	-2.692941	-1.381654
59	6	0	-9.033794	-4.655205	-0.464265
60	1	0	-0.721369	1.017630	1.912013
61	1	0	0.200334	1.385351	-0.207976
62	1	0	-3.145437	0.537564	1.177500
63	1	0	1.961710	-4.068620	0.723813
64	1	0	0.664717	-3.753726	1.859181
65	1	0	5.468670	-3.742835	-0.509954
66	1	0	5.343349	-3.900434	1.908681
67	1	0	3.747669	-4.204548	1.099973
68	1	0	2.754568	-0.595558	2.239733
69	1	0	4.934573	-2.155326	4.092525
70	1	0	5.929524	-1.646461	2.727042
71	1	0	4.630768	-0.591734	3.316953
72	1	0	5.894256	1.731598	-0.106841
73	1	0	8.216123	1.380390	0.744658
74	1	0	9.085729	-0.935104	1.051878
75	1	0	7.611694	-2.875908	0.559971
76	1	0	1.128830	0.080571	0.954603
77	1	0	-0.274688	-4.508728	-0.284181
78	1	0	0.635921	-3.271896	-1.160736
79	1	0	-1.624943	-2.727636	0.826366
80	1	0	-1.694237	-2.504096	-0.914540
81	1	0	-2.138096	-0.361883	-0.742261
82	1	0	-4.649613	2.209024	1.391589
83	1	0	-3.472697	2.816893	0.226145
84	1	0	0.082649	3.190916	2.058288
85	1	0	-1.068266	3.856913	0.907454
86	1	0	-4.085052	3.909268	2.960583
87	1	0	-3.281912	4.702750	1.604901
88	1	0	-1.601539	4.428784	3.374236
89	1	0	-2.319906	0.231669	3.289074
90	1	0	-3.859263	1.093861	3.341943
91	1	0	-0.905282	0.921918	5.006482
92	1	0	-3.200453	1.029111	-1.696373
93	1	0	-4.034357	2.557735	-3.434135
94	1	0	-1.071049	5.942748	-3.911069
95	1	0	1.106388	6.600615	-2.941460
96	1	0	1.058292	3.151139	-0.407966
97	1	0	-8.181801	-3.213795	1.695788
98	1	0	-7.229232	-4.025337	-2.411894
99	1	0	-5.246073	-2.529227	-2.236075
100	1	0	-6.189880	-1.728798	1.853791
101	1	0	-8.733987	-5.701084	-0.315932
102	1	0	-9.786556	-4.420060	0.294899
103	1	0	-9.509399	-4.592746	-1.448938
104	1	0	1.104779	2.228703	5.286800
105	1	0	0.380543	3.635984	4.338513
106	1	0	2.565958	-5.116878	-3.804076
107	1	0	1.179145	-5.030815	-2.694344
108	1	0	1.178177	-4.046829	-4.193122
109	1	0	4.039551	6.309374	-0.854166
110	1	0	3.373956	6.228175	-2.507972
111	1	0	2.566075	7.229722	-1.262519
112	1	0	-2.818650	2.698198	4.636654

TS1’-A-*OF*

Center Number	Atomic Number	Atomic Type	Coordinates (Angstroms) X	Y	Z
1	6	0	4.028743	-3.871176	-2.717180
2	6	0	3.912215	-2.991159	-1.640688
3	6	0	4.831098	-3.007630	-0.581709
4	6	0	5.888636	-3.927636	-0.632940

5	6	0	6.020864	-4.806221	-1.703680
6	6	0	5.083783	-4.780155	-2.743705
7	8	0	2.864717	-2.109851	-1.659745
8	6	0	2.801637	-0.994495	-0.790880
9	6	0	3.712321	-1.003645	0.315035
10	6	0	4.597815	-2.100074	0.540370
11	6	0	3.790582	0.074456	1.289682
12	8	0	4.581499	0.078833	2.229959
13	8	0	1.955902	-0.171081	-1.097627
14	8	0	2.927844	1.120001	1.104493
15	6	0	3.080023	2.185664	2.046919
16	6	0	3.745229	-3.466641	1.878243
17	6	0	3.152437	-2.665109	2.874138
18	6	0	3.969132	-2.352707	4.106644
19	6	0	1.967075	-1.951260	2.663373
20	6	0	0.952009	-2.161119	1.701561
21	6	0	0.248779	-1.057334	1.206547
22	6	0	-0.720898	-1.158909	0.206733
23	6	0	-1.269589	-2.509273	-0.176288
24	6	0	-0.150272	-3.555052	-0.110109
25	6	0	0.495860	-3.543989	1.276459
26	7	0	-1.189459	-0.112283	-0.478807
27	6	0	-0.668227	1.237760	-0.334982
28	6	0	-0.766096	2.026214	-1.630189
29	6	0	-1.849193	1.919592	-2.477569
30	6	0	-1.929880	2.746171	-3.624589
31	7	0	-1.030574	3.650236	-3.952652
32	6	0	0.073732	3.749328	-3.156348
33	6	0	0.282114	2.935284	-1.991834
34	6	0	1.494249	3.069898	-1.276860
35	6	0	2.443001	4.001715	-1.656133
36	6	0	2.221680	4.836903	-2.781079
37	6	0	1.064077	4.697289	-3.512377
38	8	0	3.568494	4.051241	-0.887198
39	6	0	4.627206	4.910733	-1.288244
40	6	0	-1.276390	1.905512	0.926942
41	7	0	-2.788391	1.913044	0.981265
42	6	0	-3.404834	3.079366	0.262482
43	6	0	-3.018630	4.388181	0.984760
44	6	0	-1.909116	4.075671	2.003459
45	6	0	-0.785443	3.324701	1.266133
46	6	0	-2.520085	3.189735	3.122081
47	6	0	-3.191222	1.969859	2.428763
48	6	0	-1.515496	2.780911	4.166628
49	6	0	-1.520195	3.224167	5.423246
50	8	0	-4.213664	-0.170643	0.503301
51	6	0	-4.329009	-0.822775	-0.586858
52	8	0	-3.637828	-0.632963	-1.625989
53	6	0	-5.351507	-1.925606	-0.609163
54	6	0	-6.153251	-2.183619	0.508202
55	6	0	-7.088454	-3.215056	0.482513
56	6	0	-7.246083	-4.017595	-0.655627
57	6	0	-6.441819	-3.749949	-1.772475
58	6	0	-5.506073	-2.719710	-1.751393
59	6	0	-8.236038	-5.156280	-0.670869
60	1	0	-1.007997	1.230506	1.741612
61	1	0	0.392285	1.119224	-0.112048
62	1	0	-3.248170	0.987262	0.609218
63	1	0	1.296465	-4.277410	1.351932
64	1	0	-0.257230	-3.852323	2.019358
65	1	0	5.442664	-1.888193	1.186115
66	1	0	4.621055	-4.056236	2.141504
67	1	0	3.131938	-3.913906	1.108333
68	1	0	1.867007	-1.033136	3.242073
69	1	0	3.829469	-3.139001	4.861547
70	1	0	5.036745	-2.302098	3.873556
71	1	0	3.676632	-1.399317	4.553909
72	1	0	3.297439	-3.821951	-3.517297
73	1	0	5.179696	-5.465076	-3.581144
74	1	0	6.846800	-5.510531	-1.730707
75	1	0	6.604611	-3.945268	0.184826
76	1	0	0.633997	-0.084562	1.483754
77	1	0	-0.556086	-4.548154	-0.329525
78	1	0	0.604397	-3.327279	-0.871760
79	1	0	-2.073515	-2.776359	0.524546
80	1	0	-1.719924	-2.459792	-1.170711
81	1	0	-2.020378	-0.272114	-1.073795

82	1	0	-4.480381	2.893332	0.251116
83	1	0	-3.045642	3.054197	-0.762394
84	1	0	0.119815	3.255534	1.874784
85	1	0	-0.518456	3.872783	0.359210
86	1	0	-3.885693	4.823325	1.492495
87	1	0	-2.665219	5.121412	0.253809
88	1	0	-1.522358	4.998410	2.443576
89	1	0	-2.900706	1.017528	2.876175
90	1	0	-4.280589	2.028337	2.446628
91	1	0	-0.739573	2.080636	3.855931
92	1	0	-2.657054	1.219922	-2.281693
93	1	0	-2.794574	2.656999	-4.280475
94	1	0	0.880728	5.306766	-4.391199
95	1	0	2.961624	5.569241	-3.080851
96	1	0	1.749993	2.403767	-0.464759
97	1	0	-7.708045	-3.403322	1.356417
98	1	0	-6.554010	-4.357871	-2.667360
99	1	0	-4.883968	-2.511506	-2.615169
100	1	0	-6.031824	-1.563101	1.389423
101	1	0	-7.756227	-6.100112	-0.379451
102	1	0	-9.059660	-4.980211	0.028564
103	1	0	-8.660338	-5.304273	-1.669512
104	1	0	-0.768269	2.912203	6.142247
105	1	0	-2.279558	3.917013	5.780106
106	1	0	2.758022	1.870631	3.045076
107	1	0	4.119354	2.515932	2.100800
108	1	0	2.451017	2.996182	1.677937
109	1	0	5.429247	4.748841	-0.566873
110	1	0	4.987032	4.660989	-2.293715
111	1	0	4.323593	5.964964	-1.261973
112	1	0	-3.301892	3.776286	3.616769

TS1-A-*OF-exo*

Center Number	Atomic Number	Atomic Type	Coordinates (Angstroms) X	Y	Z
1	6	0	4.028743	-3.871176	-2.717180
2	6	0	3.912215	-2.991159	-1.640688
3	6	0	4.831098	-3.007630	-0.581709
4	6	0	5.888636	-3.927636	-0.632940
5	6	0	6.020864	-4.806221	-1.703680
6	6	0	5.083783	-4.780155	-2.743705
7	8	0	2.864717	-2.109851	-1.659745
8	6	0	2.801637	-0.994495	-0.790880
9	6	0	3.712321	-1.003645	0.315035
10	6	0	4.597815	-2.100074	0.540370
11	6	0	3.790582	0.074456	1.289682
12	8	0	4.581499	0.078833	2.229959
13	8	0	1.955902	-0.171081	-1.097627
14	8	0	2.927844	1.120001	1.104493
15	6	0	3.080023	2.185664	2.046919
16	6	0	3.745229	-3.466641	1.878243
17	6	0	3.152437	-2.665109	2.874138
18	6	0	3.969132	-2.352707	4.106644
19	6	0	1.967075	-1.951260	2.663373
20	6	0	0.952009	-2.161119	1.701561
21	6	0	0.248779	-1.057334	1.206547
22	6	0	-0.720898	-1.158909	0.206733
23	6	0	-1.269589	-2.509273	-0.176288
24	6	0	-0.150272	-3.555052	-0.110109
25	6	0	0.495860	-3.543989	1.276459
26	7	0	-1.189459	-0.112283	-0.478807
27	6	0	-0.668227	1.237760	-0.334982
28	6	0	-0.766096	2.026214	-1.630189
29	6	0	-1.849193	1.919592	-2.477569
30	6	0	-1.929880	2.746171	-3.624589
31	7	0	-1.030574	3.650236	-3.952652
32	6	0	0.073732	3.749328	-3.156348
33	6	0	0.282114	2.935284	-1.991834
34	6	0	1.494249	3.069898	-1.276860
35	6	0	2.443001	4.001715	-1.656133
36	6	0	2.221680	4.836903	-2.781079
37	6	0	1.064077	4.697289	-3.512377

38	8	0	3.568494	4.051241	-0.887198
39	6	0	4.627206	4.910733	-1.288244
40	6	0	-1.276390	1.905512	0.926942
41	7	0	-2.788391	1.913044	0.981265
42	6	0	-3.404834	3.079366	0.262482
43	6	0	-3.018630	4.388181	0.984760
44	6	0	-1.909116	4.075671	2.003459
45	6	0	-0.785443	3.324701	1.266133
46	6	0	-2.520085	3.189735	3.122081
47	6	0	-3.191222	1.969859	2.428763
48	6	0	-1.515496	2.780911	4.166628
49	6	0	-1.520195	3.224167	5.423246
50	8	0	-4.213664	-0.170643	0.503301
51	6	0	-4.329009	-0.822775	-0.586858
52	8	0	-3.637828	-0.632963	-1.625989
53	6	0	-5.351507	-1.925606	-0.609163
54	6	0	-6.153251	-2.183619	0.508202
55	6	0	-7.088454	-3.215056	0.482513
56	6	0	-7.246083	-4.017595	-0.655627
57	6	0	-6.441819	-3.749949	-1.772475
58	6	0	-5.506073	-2.719710	-1.751393
59	6	0	-8.236038	-5.156280	-0.670869
60	1	0	-1.007997	1.230506	1.741612
61	1	0	0.392285	1.119224	-0.112048
62	1	0	-3.248170	0.987262	0.609218
63	1	0	1.296465	-4.277410	1.351932
64	1	0	-0.257230	-3.852323	2.019358
65	1	0	5.442664	-1.888193	1.186115
66	1	0	4.621055	-4.056236	2.141504
67	1	0	3.131938	-3.913906	1.108333
68	1	0	1.867007	-1.033136	3.242073
69	1	0	3.829469	-3.139001	4.861547
70	1	0	5.036745	-2.302098	3.873556
71	1	0	3.676632	-1.399317	4.553909
72	1	0	3.297439	-3.821951	-3.517297
73	1	0	5.179696	-5.465076	-3.581144
74	1	0	6.846800	-5.510531	-1.730707
75	1	0	6.604611	-3.945268	0.184826
76	1	0	0.633997	-0.084562	1.483754
77	1	0	-0.556086	-4.548154	-0.329525
78	1	0	0.604397	-3.327279	-0.871760
79	1	0	-2.073515	-2.776359	0.524546
80	1	0	-1.719924	-2.459792	-1.170711
81	1	0	-2.020378	-0.272114	-1.073795
82	1	0	-4.480381	2.893332	0.251116
83	1	0	-3.045642	3.054197	-0.762394
84	1	0	0.119815	3.255534	1.874784
85	1	0	-0.518456	3.872783	0.359210
86	1	0	-3.885693	4.823325	1.492495
87	1	0	-2.665219	5.121412	0.253809
88	1	0	-1.522358	4.998410	2.443576
89	1	0	-2.900706	1.017528	2.876175
90	1	0	-4.280589	2.028337	2.446628
91	1	0	-0.739573	2.080636	3.855931
92	1	0	-2.657054	1.219922	-2.281693
93	1	0	-2.794574	2.656999	-4.280475
94	1	0	0.880728	5.306766	-4.391199
95	1	0	2.961624	5.569241	-3.080851
96	1	0	1.749993	2.403767	-0.464759
97	1	0	-7.708045	-3.403322	1.356417
98	1	0	-6.554010	-4.357871	-2.667360
99	1	0	-4.883968	-2.511506	-2.615169
100	1	0	-6.031824	-1.563101	1.389423
101	1	0	-7.756227	-6.100112	-0.379451
102	1	0	-9.059660	-4.980211	0.028564
103	1	0	-8.660338	-5.304273	-1.669512
104	1	0	-0.768269	2.912203	6.142247
105	1	0	-2.279558	3.917013	5.780106
106	1	0	2.758022	1.870631	3.045076
107	1	0	4.119354	2.515932	2.100800
108	1	0	2.451017	2.996182	1.677937
109	1	0	5.429247	4.748841	-0.566873
110	1	0	4.987032	4.660989	-2.293715
111	1	0	4.323593	5.964964	-1.261973
112	1	0	-3.301892	3.776286	3.616769

TS1'-A-*OF-exo*

Center Number	Atomic Number	Atomic Type	Coordinates (Angstroms) X	Y	Z
1	6	0	-6.252863	-0.709350	1.185080
2	6	0	-7.599395	-1.063906	1.183660
3	6	0	-8.484926	-0.530145	0.237252
4	6	0	-7.980620	0.376619	-0.705617
5	6	0	-6.635011	0.733049	-0.708533
6	6	0	-5.756495	0.192932	0.237449
7	6	0	-9.936763	-0.940664	0.216452
8	6	0	-4.301721	0.574985	0.225397
9	8	0	-3.555392	0.018453	1.099172
10	8	0	-3.907875	1.386653	-0.656354
11	7	0	-1.270754	1.029420	1.727275
12	6	0	0.014730	1.019997	0.931370
13	6	0	1.001514	1.960123	1.648048
14	6	0	0.564807	2.113718	3.117978
15	6	0	-0.698114	2.991518	3.140886
16	6	0	-1.703255	2.413823	2.124525
17	6	0	0.207399	0.735630	3.732274
18	6	0	-1.038851	0.204329	2.962373
19	6	0	-0.177976	1.209204	-0.606261
20	6	0	-0.263718	2.659338	-1.075833
21	6	0	-1.467248	3.318062	-1.218095
22	6	0	-1.490315	4.676731	-1.618096
23	7	0	-0.417494	5.398638	-1.859998
24	6	0	0.790870	4.776808	-1.731429
25	6	0	0.934973	3.396156	-1.361071
26	6	0	2.235816	2.853688	-1.267962
27	6	0	3.352304	3.640405	-1.487109
28	6	0	3.214374	5.004172	-1.848189
29	6	0	1.955150	5.545814	-1.972614
30	6	0	1.368615	-0.223290	3.718279
31	6	0	1.891527	-0.787658	4.806101
32	8	0	4.556373	3.020858	-1.317675
33	6	0	5.743131	3.754060	-1.591156
34	7	0	-1.316269	0.437387	-1.092121
35	6	0	-1.400579	-0.900562	-1.194151
36	6	0	-0.429637	-1.811069	-0.780630
37	6	0	-0.600438	-3.204254	-0.922160
38	6	0	-1.742206	-3.707786	-1.797497
39	6	0	-2.378952	-2.629434	-2.666445
40	6	0	-2.684864	-1.407038	-1.802548
41	6	0	0.129226	-4.233245	-0.304567
42	6	0	1.051144	-4.261925	0.755014
43	6	0	1.641442	-3.156860	1.392890
44	6	0	3.367776	-2.807836	0.298262
45	6	0	3.004496	-2.461543	-1.037797
46	6	0	3.981710	-1.769693	1.126819
47	6	0	3.830514	-0.440593	0.714979
48	8	0	3.069184	-0.138788	-0.377115
49	6	0	2.724939	-1.104910	-1.379423
50	6	0	4.715880	-2.025184	2.294720
51	6	0	5.287742	-0.982097	3.016881
52	6	0	5.146491	0.337269	2.567705
53	6	0	4.426547	0.615048	1.408435
54	6	0	2.785055	-3.577819	-1.951137
55	8	0	2.312871	-3.232744	-3.171792
56	6	0	2.036981	-4.332811	-4.041547
57	6	0	1.552150	-5.631964	1.159719
58	8	0	2.192099	-0.612062	-2.356658
59	8	0	3.019130	-4.748380	-1.654610
60	1	0	0.366162	-0.003986	1.045997
61	1	0	0.712411	0.763563	-1.058434
62	1	0	-2.135097	0.577984	1.233965
63	1	0	-1.372383	-4.538551	-2.409534
64	1	0	-2.509599	-4.136996	-1.135858
65	1	0	3.757015	-3.812360	0.423060
66	1	0	1.188604	-2.178330	1.297124
67	1	0	2.103058	-3.328431	2.361256
68	1	0	-0.138678	-5.223935	-0.668232
69	1	0	2.311488	-5.960987	0.440653
70	1	0	2.002277	-5.620318	2.156719

71	1	0	0.745191	-6.371779	1.151550
72	1	0	4.337454	1.621443	1.012869
73	1	0	5.608834	1.150894	3.118556
74	1	0	5.854035	-1.192469	3.918827
75	1	0	4.836859	-3.054078	2.623621
76	1	0	0.479007	-1.442361	-0.338355
77	1	0	-3.297286	-3.007637	-3.127286
78	1	0	-1.695881	-2.343618	-3.475328
79	1	0	-3.371775	-1.681128	-0.991668
80	1	0	-3.165137	-0.610614	-2.375374
81	1	0	-2.225574	0.920051	-1.140752
82	1	0	-2.714447	2.310497	2.520762
83	1	0	-1.755822	3.003021	1.211941
84	1	0	2.009553	1.554519	1.564883
85	1	0	1.014077	2.945934	1.178719
86	1	0	-1.129238	3.012833	4.147347
87	1	0	-0.450543	4.021739	2.868409
88	1	0	1.366102	2.575193	3.700904
89	1	0	-0.922119	-0.831093	2.635992
90	1	0	-1.952340	0.269421	3.555657
91	1	0	1.816752	-0.447899	2.752749
92	1	0	-2.415754	2.830158	-1.019255
93	1	0	-2.451534	5.176539	-1.727932
94	1	0	1.818070	6.584809	-2.253766
95	1	0	4.086131	5.620938	-2.030675
96	1	0	2.405149	1.813397	-1.031036
97	1	0	-7.973562	-1.764288	1.926989
98	1	0	-8.653783	0.806086	-1.444091
99	1	0	-6.243552	1.434217	-1.437616
100	1	0	-5.568056	-1.122253	1.917870
101	1	0	-10.092816	-1.804809	-0.442990
102	1	0	-10.286579	-1.225868	1.213966
103	1	0	-10.576041	-0.131959	-0.152716
104	1	0	2.745017	-1.455081	4.736808
105	1	0	1.491778	-0.596705	5.800115
106	1	0	2.937589	-4.924053	-4.229130
107	1	0	1.273567	-4.985564	-3.606130
108	1	0	1.672851	-3.887269	-4.968063
109	1	0	6.565829	3.059029	-1.418831
110	1	0	5.770038	4.098656	-2.631929
111	1	0	5.848643	4.615699	-0.919844
112	1	0	-0.085607	0.896706	4.775585

INT4-A

Center Number	Atomic Number	Atomic Type	Coordinates (Angstroms) X	Y	Z
1	6	0	6.246388	-1.035303	0.776571
2	6	0	4.965972	-1.557238	0.578848
3	6	0	4.739491	-2.934504	0.547758
4	6	0	5.828126	-3.788416	0.747489
5	6	0	7.114097	-3.286688	0.946839
6	6	0	7.321262	-1.904057	0.952418
7	8	0	3.948028	-0.656834	0.402892
8	6	0	2.598846	-1.032219	0.585493
9	6	0	2.304641	-2.415045	0.577286
10	6	0	3.358509	-3.436495	0.230967
11	6	0	1.011164	-2.966609	0.839886
12	8	0	0.758766	-4.177577	0.783952
13	8	0	1.846580	-0.061028	0.702817
14	8	0	-0.005376	-2.078298	1.140226
15	6	0	-1.250454	-2.698311	1.490277
16	6	0	3.273090	-3.939741	-1.279163
17	6	0	3.537998	-2.840695	-2.258503
18	6	0	4.952030	-2.704960	-2.756432
19	6	0	2.618356	-1.897083	-2.575647
20	6	0	1.203124	-1.876657	-2.245359
21	6	0	0.618638	-0.667868	-1.939393
22	6	0	-0.745764	-0.574239	-1.581637
23	6	0	-1.698789	-1.688049	-1.940880
24	6	0	-1.060107	-2.706109	-2.884173
25	6	0	0.333065	-3.106797	-2.380806
26	7	0	-1.266842	0.446722	-0.924009

27	6	0	-0.503263	1.510466	-0.277083
28	6	0	-0.801139	2.864054	-0.894517
29	6	0	-2.073529	3.218811	-1.291347
30	6	0	-2.301870	4.490765	-1.868378
31	7	0	-1.360176	5.387870	-2.075235
32	6	0	-0.084982	5.052024	-1.723685
33	6	0	0.265487	3.792118	-1.129761
34	6	0	1.624832	3.534873	-0.826779
35	6	0	2.600673	4.481211	-1.090831
36	6	0	2.253243	5.730965	-1.666660
37	6	0	0.938995	5.998444	-1.971633
38	8	0	3.874260	4.131913	-0.764251
39	6	0	4.922318	5.058703	-1.019643
40	6	0	-0.572400	1.359206	1.266234
41	7	0	-1.944257	1.264355	1.893076
42	6	0	-2.620630	2.593028	2.061791
43	6	0	-1.810010	3.454928	3.054260
44	6	0	-0.485242	2.737342	3.367030
45	6	0	0.225801	2.428291	2.041109
46	6	0	-0.805590	1.420904	4.124557
47	6	0	-1.782743	0.610645	3.242407
48	6	0	0.457803	0.702373	4.538248
49	6	0	1.010656	-0.379595	3.989241
50	8	0	-3.775650	-0.423271	1.125527
51	6	0	-4.381444	-0.263923	0.016510
52	8	0	-3.978280	0.497162	-0.911743
53	6	0	-5.635484	-1.056035	-0.207376
54	6	0	-6.120854	-1.919326	0.781698
55	6	0	-7.280380	-2.657935	0.563993
56	6	0	-7.984468	-2.556413	-0.644129
57	6	0	-7.494072	-1.685708	-1.627272
58	6	0	-6.334189	-0.945942	-1.414943
59	6	0	-9.222693	-3.382685	-0.889628
60	1	0	-0.124710	0.381555	1.434220
61	1	0	0.544450	1.279769	-0.437562
62	1	0	-2.620125	0.603841	1.372810
63	1	0	0.806263	-3.807049	-3.075240
64	1	0	0.239307	-3.628712	-1.421005
65	1	0	3.161935	-4.343095	0.811444
66	1	0	4.008083	-4.743284	-1.401830
67	1	0	2.280225	-4.375802	-1.386951
68	1	0	2.976076	-0.998573	-3.079091
69	1	0	5.663925	-2.662727	-1.925185
70	1	0	5.223281	-3.584400	-3.356698
71	1	0	5.083357	-1.813021	-3.376146
72	1	0	6.375660	0.042179	0.790593
73	1	0	8.318664	-1.500899	1.103730
74	1	0	7.947738	-3.966703	1.095257
75	1	0	5.659324	-4.862847	0.730717
76	1	0	1.257058	0.200537	-1.843955
77	1	0	-1.701505	-3.589717	-2.958530
78	1	0	-0.975442	-2.276912	-3.889742
79	1	0	-1.996500	-2.175479	-1.004820
80	1	0	-2.613724	-1.252158	-2.356043
81	1	0	-2.310321	0.478706	-0.849396
82	1	0	-3.634141	2.378439	2.406237
83	1	0	-2.686802	3.048625	1.076803
84	1	0	1.234436	2.043695	2.199351
85	1	0	0.306958	3.354324	1.466979
86	1	0	-2.379039	3.618514	3.975352
87	1	0	-1.610662	4.436227	2.613273
88	1	0	0.148184	3.375319	3.990485
89	1	0	-1.442837	-0.406236	3.058526
90	1	0	-2.783475	0.556752	3.673392
91	1	0	0.974998	1.187313	5.366975
92	1	0	-2.908563	2.532816	-1.180032
93	1	0	-3.312341	4.765281	-2.167023
94	1	0	0.649365	6.944026	-2.418052
95	1	0	3.014127	6.473919	-1.872294
96	1	0	1.949200	2.602733	-0.377069
97	1	0	-7.649050	-3.323983	1.340662
98	1	0	-8.030659	-1.588843	-2.568226
99	1	0	-5.954766	-0.271246	-2.174712
100	1	0	-5.576836	-1.998403	1.716623
101	1	0	-8.966166	-4.354125	-1.332743
102	1	0	-9.761147	-3.583370	0.042340
103	1	0	-9.907956	-2.881151	-1.580663

104	1	0	1.951523	-0.767150	4.369780
105	1	0	0.594919	-0.912752	3.140329
106	1	0	-1.483074	-3.521153	0.812089
107	1	0	-1.205876	-3.098124	2.509623
108	1	0	-2.014330	-1.923101	1.419950
109	1	0	5.837779	4.566940	-0.688765
110	1	0	5.000979	5.291745	-2.088624
111	1	0	4.784185	5.987993	-0.453244
112	1	0	-1.336689	1.703581	5.042386

TS2-A

Center Number	Atomic Number	Atomic Type	Coordinates (Angstroms) X	Y	Z
1	6	0	6.085289	-0.770633	0.795218
2	6	0	4.885346	-1.433393	0.542176
3	6	0	4.831049	-2.793678	0.260740
4	6	0	6.035222	-3.507649	0.287414
5	6	0	7.248522	-2.873008	0.546959
6	6	0	7.273907	-1.495741	0.785834
7	8	0	3.730155	-0.678234	0.634119
8	6	0	2.516225	-1.305250	0.828875
9	6	0	2.286738	-2.576340	0.106830
10	6	0	3.535133	-3.443437	-0.163723
11	6	0	1.164837	-3.413806	0.653668
12	8	0	1.209372	-4.633833	0.722096
13	8	0	1.702131	-0.684224	1.478477
14	8	0	0.060937	-2.718335	0.967470
15	6	0	-1.073112	-3.499498	1.391236
16	6	0	3.673949	-3.827181	-1.671475
17	6	0	3.799816	-2.563514	-2.477507
18	6	0	5.069648	-2.329054	-3.242550
19	6	0	2.836729	-1.637081	-2.356912
20	6	0	1.567674	-1.864719	-1.601329
21	6	0	0.837972	-0.623258	-1.325668
22	6	0	-0.518661	-0.493774	-1.375665
23	6	0	-1.379964	-1.633689	-1.863788
24	6	0	-0.586278	-2.486862	-2.846874
25	6	0	0.697135	-3.008717	-2.195983
26	7	0	-1.218591	0.648580	-1.100550
27	6	0	-0.647062	1.832432	-0.490684
28	6	0	-1.407460	3.089274	-0.893916
29	6	0	-2.772257	3.087084	-1.093243
30	6	0	-3.436895	4.281046	-1.462923
31	7	0	-2.837326	5.438176	-1.648385
32	6	0	-1.483262	5.470264	-1.481576
33	6	0	-0.705473	4.322607	-1.107303
34	6	0	0.695148	4.474585	-0.975440
35	6	0	1.309672	5.695073	-1.194361
36	6	0	0.539286	6.828723	-1.558656
37	6	0	-0.823866	6.705271	-1.696501
38	8	0	2.662644	5.720355	-1.034953
39	6	0	3.349822	6.944938	-1.261875
40	6	0	-0.410053	1.619133	1.037348
41	7	0	-1.579733	0.987879	1.757713
42	6	0	-2.636789	1.973004	2.164621
43	6	0	-2.054963	2.942050	3.213764
44	6	0	-0.534328	2.722799	3.292087
45	6	0	0.033734	2.842693	1.863987
46	6	0	-0.282937	1.313399	3.873019
47	6	0	-1.043511	0.292075	2.978460
48	6	0	1.172628	0.946050	4.053716
49	6	0	2.187222	1.795385	4.210933
50	8	0	-3.126809	-0.981860	1.040969
51	6	0	-3.967380	-0.834372	0.092674
52	8	0	-3.973185	0.125788	-0.724389
53	6	0	-5.002587	-1.914435	-0.074318
54	6	0	-5.097762	-2.970297	0.839029
55	6	0	-6.045468	-3.975094	0.661186
56	6	0	-6.924183	-3.952903	-0.430429
57	6	0	-6.822467	-2.892240	-1.341463
58	6	0	-5.878328	-1.884555	-1.165461
59	6	0	-7.969201	-5.027107	-0.606701

60	1	0	0.360333	0.851963	1.093511
61	1	0	0.361610	1.937905	-0.895763
62	1	0	-2.088924	0.217080	1.206504
63	1	0	1.283743	-3.540300	-2.947318
64	1	0	0.415198	-3.750158	-1.446696
65	1	0	3.401068	-4.376438	0.389300
66	1	0	4.555989	-4.461868	-1.795014
67	1	0	2.806855	-4.429295	-1.953483
68	1	0	2.958765	-0.657791	-2.815695
69	1	0	5.936823	-2.382773	-2.570980
70	1	0	5.215975	-3.101026	-4.010126
71	1	0	5.071762	-1.351157	-3.734044
72	1	0	6.064543	0.293429	1.006081
73	1	0	8.214815	-0.989762	0.980394
74	1	0	8.169598	-3.447712	0.558724
75	1	0	6.016748	-4.575941	0.086848
76	1	0	1.449717	0.228422	-1.045424
77	1	0	-1.186609	-3.338221	-3.188186
78	1	0	-0.340419	-1.885466	-3.730667
79	1	0	-1.709764	-2.233042	-1.008481
80	1	0	-2.284365	-1.230693	-2.328998
81	1	0	-2.238739	0.537937	-1.049685
82	1	0	-3.472727	1.378798	2.536623
83	1	0	-2.962549	2.484600	1.262764
84	1	0	1.124595	2.887229	1.871480
85	1	0	-0.330530	3.774207	1.423778
86	1	0	-2.512985	2.772790	4.193969
87	1	0	-2.272725	3.974531	2.924224
88	1	0	-0.087242	3.482007	3.939239
89	1	0	-0.398080	-0.509357	2.617316
90	1	0	-1.907552	-0.146793	3.479850
91	1	0	1.379657	-0.122097	4.070802
92	1	0	-3.361451	2.183239	-0.973269
93	1	0	-4.516022	4.261623	-1.607372
94	1	0	-1.436413	7.555455	-1.978386
95	1	0	1.013004	7.787211	-1.732151
96	1	0	1.332042	3.645933	-0.691806
97	1	0	-6.106246	-4.791998	1.376799
98	1	0	-7.491419	-2.860686	-2.198559
99	1	0	-5.796944	-1.063692	-1.869733
100	1	0	-4.420474	-2.990971	1.685929
101	1	0	-8.181751	-5.208503	-1.665504
102	1	0	-7.652004	-5.972338	-0.154304
103	1	0	-8.915844	-4.739040	-0.130383
104	1	0	3.200240	1.433001	4.358623
105	1	0	2.059837	2.874569	4.196478
106	1	0	-1.308314	-4.262827	0.644975
107	1	0	-0.856497	-3.991146	2.343650
108	1	0	-1.888350	-2.782533	1.481193
109	1	0	4.404534	6.729148	-1.086618
110	1	0	3.217196	7.294774	-2.292909
111	1	0	3.018975	7.726390	-0.566758
112	1	0	-0.751818	1.282079	4.866806

INT5-A

Center Number	Atomic Number	Atomic Type	Coordinates (Angstroms) X	Y	Z
1	6	0	5.782518	-1.129777	0.080765
2	6	0	4.545868	-1.757854	0.200299
3	6	0	4.401572	-3.141163	0.229796
4	6	0	5.576418	-3.903790	0.196850
5	6	0	6.830230	-3.304494	0.091716
6	6	0	6.931713	-1.912942	0.013021
7	8	0	3.442935	-0.928720	0.369152
8	6	0	2.317134	-1.486129	0.924301
9	6	0	1.841066	-2.783916	0.280736
10	6	0	3.034402	-3.798336	0.190345
11	6	0	0.740787	-3.382366	1.172588
12	8	0	0.780438	-4.499810	1.649012
13	8	0	1.784128	-0.889267	1.827502
14	8	0	-0.302591	-2.556297	1.317884
15	6	0	-1.443544	-3.080855	2.026173

16	6	0	2.958879	-4.615973	-1.124732
17	6	0	3.066583	-3.649631	-2.280582
18	6	0	4.122701	-3.885394	-3.319274
19	6	0	2.275504	-2.571541	-2.249772
20	6	0	1.190369	-2.408267	-1.198831
21	6	0	0.689467	-0.980663	-1.177045
22	6	0	-0.583901	-0.591508	-1.404110
23	6	0	-1.636850	-1.580299	-1.841874
24	6	0	-0.979339	-2.781067	-2.513640
25	6	0	0.036394	-3.409240	-1.560184
26	7	0	-1.046086	0.715779	-1.330419
27	6	0	-0.289675	1.741648	-0.634876
28	6	0	-0.748000	3.139194	-1.030298
29	6	0	-2.072790	3.420386	-1.292411
30	6	0	-2.471024	4.738121	-1.618059
31	7	0	-1.647540	5.761237	-1.709096
32	6	0	-0.321319	5.508792	-1.510453
33	6	0	0.197474	4.209315	-1.184174
34	6	0	1.600038	4.063498	-1.056377
35	6	0	2.454472	5.140942	-1.212245
36	6	0	1.936924	6.428264	-1.504376
37	6	0	0.579243	6.593282	-1.650460
38	8	0	3.783495	4.875468	-1.067746
39	6	0	4.709850	5.941462	-1.236171
40	6	0	-0.168857	1.426262	0.889025
41	7	0	-1.476392	1.172213	1.605570
42	6	0	-2.318708	2.391701	1.840742
43	6	0	-1.446678	3.488815	2.474664
44	6	0	-0.143285	2.845066	2.981517
45	6	0	0.670367	2.397836	1.754729
46	6	0	-0.510451	1.632484	3.862508
47	6	0	-1.143014	0.556470	2.936668
48	6	0	0.611722	1.034370	4.674138
49	6	0	1.820362	1.555712	4.879307
50	8	0	-3.280883	-0.583065	0.935789
51	6	0	-4.068080	-0.201893	0.003961
52	8	0	-3.867058	0.791961	-0.742528
53	6	0	-5.304485	-1.028630	-0.225125
54	6	0	-5.596986	-2.131014	0.585277
55	6	0	-6.741849	-2.890736	0.357723
56	6	0	-7.622933	-2.573236	-0.684839
57	6	0	-7.325068	-1.464809	-1.490045
58	6	0	-6.182048	-0.703012	-1.265423
59	6	0	-8.845861	-3.416285	-0.950880
60	1	0	0.297980	0.445409	0.909184
61	1	0	0.743493	1.649533	-0.979273
62	1	0	-2.108625	0.445713	1.115089
63	1	0	0.462294	-4.304602	-2.014020
64	1	0	-0.516945	-3.732684	-0.677665
65	1	0	2.933334	-4.484528	1.034550
66	1	0	3.772584	-5.345639	-1.146116
67	1	0	2.026038	-5.189511	-1.137124
68	1	0	2.344575	-1.797268	-3.009684
69	1	0	5.117694	-3.912252	-2.854613
70	1	0	3.979228	-4.854039	-3.817124
71	1	0	4.119047	-3.102583	-4.084105
72	1	0	5.823753	-0.045969	0.059749
73	1	0	7.903038	-1.436971	-0.080937
74	1	0	7.723618	-3.920760	0.066018
75	1	0	5.503100	-4.986870	0.243310
76	1	0	1.449723	-0.235316	-0.966888
77	1	0	-1.733936	-3.529273	-2.782711
78	1	0	-0.486239	-2.466664	-3.441981
79	1	0	-2.217594	-1.900958	-0.969006
80	1	0	-2.337877	-1.078919	-2.518225
81	1	0	-2.063226	0.781804	-1.227494
82	1	0	-3.126553	2.065206	2.499091
83	1	0	-2.763976	2.677995	0.893797
84	1	0	1.588983	1.890487	2.059028
85	1	0	0.953221	3.284453	1.186604
86	1	0	-1.990533	3.969838	3.293718
87	1	0	-1.212151	4.261385	1.735213
88	1	0	0.426304	3.576593	3.560648
89	1	0	-0.454117	-0.265252	2.736654
90	1	0	-2.074736	0.148515	3.332340
91	1	0	0.372069	0.075754	5.136633
92	1	0	-2.832838	2.648306	-1.244399

93	1	0	-3.525057	4.942042	-1.800605
94	1	0	0.156795	7.564070	-1.888283
95	1	0	2.598558	7.277504	-1.623404
96	1	0	2.053770	3.104542	-0.839015
97	1	0	-6.959615	-3.743177	0.997331
98	1	0	-7.999816	-1.198898	-2.300691
99	1	0	-5.948817	0.155130	-1.886395
100	1	0	-4.918975	-2.378425	1.394876
101	1	0	-8.633483	-4.196709	-1.693726
102	1	0	-9.191793	-3.918180	-0.041412
103	1	0	-9.670621	-2.812153	-1.343396
104	1	0	2.554474	1.041793	5.492760
105	1	0	2.132720	2.502088	4.447451
106	1	0	-1.722101	-4.056167	1.619422
107	1	0	-1.204747	-3.190903	3.087454
108	1	0	-2.234059	-2.349359	1.862797
109	1	0	5.697007	5.502002	-1.087208
110	1	0	4.651245	6.369267	-2.244465
111	1	0	4.550246	6.733974	-0.494617
112	1	0	-1.283909	1.961065	4.572403
